# Mechanism of the extremely high duplex-forming ability of oligonucleotides modified with *N*-*tert*-butylguanidine- or *N*-*tert*-butyl-*N*′- methylguanidine-bridged nucleic acids

**DOI:** 10.1093/nar/gkad608

**Published:** 2023-07-18

**Authors:** Takao Yamaguchi, Naohiro Horie, Hiroshi Aoyama, Shinji Kumagai, Satoshi Obika

**Affiliations:** Graduate School of Pharmaceutical Sciences, Osaka University, 1-6 Yamadaoka, Suita, Osaka 565-0871, Japan; Graduate School of Pharmaceutical Sciences, Osaka University, 1-6 Yamadaoka, Suita, Osaka 565-0871, Japan; Graduate School of Pharmaceutical Sciences, Osaka University, 1-6 Yamadaoka, Suita, Osaka 565-0871, Japan; Sohyaku. Innovative Research Division, Mitsubishi Tanabe Pharma Corporation, Shonan Health Innovation Park, 2-26-1 Muraoka-Higashi, Fujisawa, Kanagawa 251-8555, Japan; Graduate School of Pharmaceutical Sciences, Osaka University, 1-6 Yamadaoka, Suita, Osaka 565-0871, Japan; National Institutes of Biomedical Innovation, Health and Nutrition (NIBIOHN), 7-6-8 Saito-Asagi, Ibaraki, Osaka 567-0085, Japan; Institute for Open and Transdisciplinary Research Initiatives (OTRI), Osaka University, 1-1 Yamadaoka, Suita, Osaka 565-0871, Japan

## Abstract

Antisense oligonucleotides (ASOs) are becoming a promising class of drugs for treating various diseases. Over the past few decades, many modified nucleic acids have been developed for application to ASOs, aiming to enhance their duplex-forming ability toward cognate mRNA and improve their stability against enzymatic degradations. Modulating the sugar conformation of nucleic acids by substituting an electron-withdrawing group at the 2′-position or incorporating a 2′,4′-bridging structure is a common approach for enhancing duplex-forming ability. Here, we report on incorporating an *N*-*tert*-butylguanidinium group at the 2′,4′-bridging structure, which greatly enhances duplex-forming ability because of its interactions with the minor groove. Our results indicated that hydrophobic substituents fitting the grooves of duplexes also have great potential to increase duplex-forming ability.

## Graphical Abstract

**Graphical Abstract:**
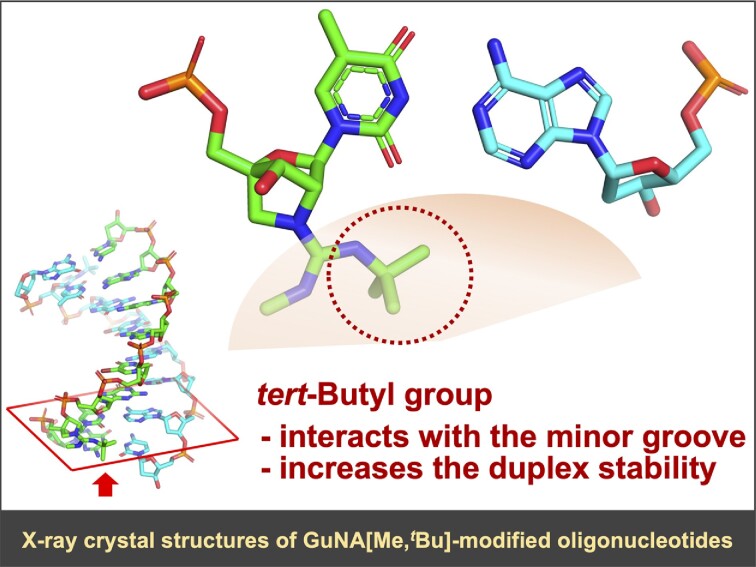

## INTRODUCTION

**Figure 1.**
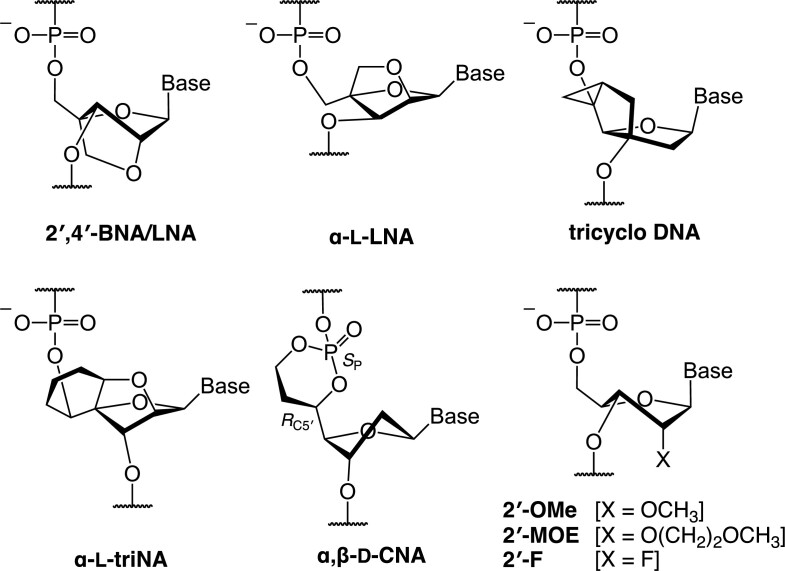
Structures of 2′,4′-BNA/LNA, α-l-LNA, tricyclo DNA, α-l-triNA, α,β-d-CNA, 2′-OMe, 2′-MOE and 2′-F.

Modified nucleic acids play important roles in developing effective and less-toxic therapeutic oligonucleotides (1). For an application to antisense oligonucleotides (ASOs), numerous modified nucleic acids have been designed to enhance duplex-forming ability toward target RNA and improve stability against enzymatic degradations. 2′-*O*,4′-*C*-Methylene-bridged nucleic acid (2′,4′-BNA) (2,3), which is also known as locked nucleic acid (LNA) (4,5), is now commonly used in ASO design because it dramatically enhances the RNA-binding affinity (Δ*T*_m_ = +5°C/mod.) (Figure 1) (6). 2′,4′-BNA/LNA has a restricted sugar conformation which is preferable for hybridizing to complementary single-stranded RNA (ssRNA). Similar to 2′,4′-BNA/LNA, restriction of the sugar pucker or phosphate backbone is a promising approach to increasing duplex-forming ability. The successful examples of which are α-l-LNA (Δ*T*_m_ = +5°C/mod.) (7,8), tricyclo-DNA (Δ*T*_m_ = +2 to + 4°C/mod.) (9–13), α-l-triNA (Δ*T*_m_ = +5 to + 8°C/mod.) (14), α,β-d-CNA (Δ*T*_m_ = +3°C/mod.) (15) and the like (16). Another approach employs the 2′-substitution with an electron-withdrawing group (EWG) such as methoxy (2′-OMe), 2-(methoxy)ethoxy (2′-MOE) (17), or fluorine (2′-F) (18). These EWGs modulate the sugar pucker to N-type (northern) through a gauche effect, but their effect is moderate in terms of enhancing the RNA-binding affinity (Δ*T*_m_ = +2°C/mod.). Nucleobase modification enhancing π–π stacking and/or hydrogen-bond formations (e.g. G-clamp) is also a powerful method to increase duplex-forming ability (19,20). Still, therapeutic applications are rarely seen, except for naturally occurring nucleobases such as 5-methylcytosine (^m^C), possibly due to safety concerns. It is also known that replacing the anionic phosphodiester linkage in nucleic acids with a cationic guanidinium group can greatly improve the DNA- and RNA-binding affinity (21–25). Charge neutralization of the anionic oligonucleotides by incorporation of cationic substituent groups (26–29) is an alternative approach to improve their ability to form duplexes. The effect of the introduction of cationic groups on RNA-binding affinity is generally moderate (Δ*T*_m_ < +2°C/mod.) and is easily affected by buffer conditions (salt concentration).

**Figure 2.**
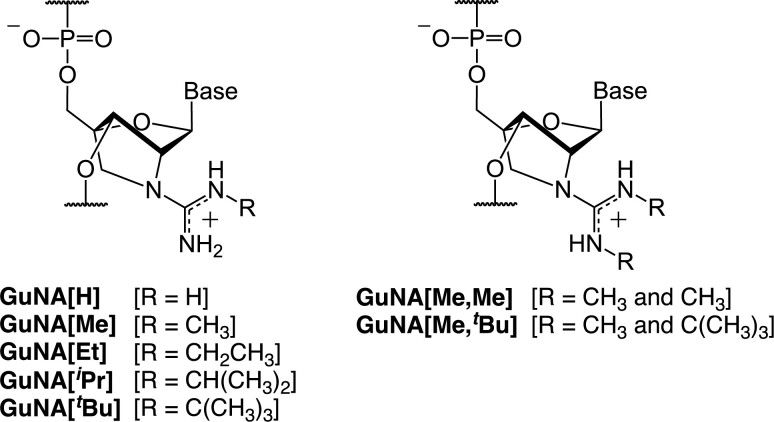
Structures of GuNA[H], GuNA[Me] and the newly designed *N*-substituted GuNA analogs (GuNA[Et], GuNA[*^i^*Pr], GuNA[*^t^*Bu], GuNA[Me,Me] and GuNA[Me,*^t^*Bu]).

We previously reported that oligonucleotides modified with guanidine- or *N*-methylguanidine-bridged nucleic acid (GuNA[H] and GuNA[Me], respectively; Figure 2) exhibited higher stability against 3′-exonuclease than those modified with 2′,4′-BNA/LNA (30–32). However, the GuNA[H]- and GuNA[Me]-modified oligonucleotides, despite their cationic characters, had almost the same RNA-binding affinity (Δ*T*_m_ = +5°C/mod.) with the 2′,4′-BNA/LNA-modified ones. We recently demonstrated that GuNA[H]-modified ASOs are well tolerated and have altered organ-specificity, as well as a preference for skeletal muscle, compared with their 2′,4′-BNA/LNA-modified counterparts (33). Moreover, GuNA[H]-modified anti-miRNA oligonucleotides were shown to have high activity, possibly due to the additional interactions between GuNA[H] and miRISC (34). Here, we report the synthesis and properties of oligonucleotides bearing other GuNA analogs, which have one or two alkyl groups at the guanidine moiety (GuNA[Et], GuNA[*^i^*Pr], GuNA[*^t^*Bu], GuNA[Me,Me] and GuNA[Me,*^t^*Bu] in Figure 2). We found that incorporation of GuNA[R] or GuNA[R,R] into the oligonucleotide significantly increased the RNA-binding affinity as the *N*-alkyl group became larger [*tert*-butyl (Δ*T*_m_ = +8°C/mod.) > isopropyl (Δ*T*_m_ = +7°C/mod.) > ethyl (Δ*T*_m_ = +6°C/mod.) > methyl and hydrogen (Δ*T*_m_ = +5°C/mod.)]. X-ray crystal structures of the self-complementary GuNA[Me,Me]- and GuNA[Me,*^t^*Bu]-modified oligonucleotides showed that interactions between the *N*-*tert*-butyl group and the minor groove greatly enhanced the duplex stability. Our results indicated that substituent groups fitting the grooves of DNA/DNA, DNA/RNA or RNA/RNA duplexes have much potential to increase the duplex-forming ability of therapeutic oligonucleotides.

## MATERIALS AND METHODS

### Synthesis of GuNA phosphoramidites

All moisture-sensitive reactions were carried out in well-dried glassware under N_2_ or Ar atmosphere. Dehydrated acetonitrile, 1,2-dichloroethane (DCE), dichloromethane, *N,N*-dimethylacetamide (DMA), *N,N*-dimethylformamide (DMF) and tetrahydrofuran (THF) were used as purchased. ^1^H, ^13^C and ^31^P NMR spectra were recorded on JEOL JNM-AL300, JNM-ECS400 and JNM-ECA500 spectrometers. Chemical shift values are expressed in δ values (ppm) relative to tetramethylsilane (δ = 0.00 ppm) as the internal standard, CHCl_3_ (δ = 7.26 ppm) or DMSO (δ = 2.49 ppm) for ^1^H NMR, CDCl_3_ (δ = 77.0 ppm) or DMSO (δ = 39.5 ppm) for ^13^C NMR, and 5% H_3_PO_4_ (δ = 0.00 ppm) for ^31^P NMR. A MALDI-TOF mass spectrometer (SpiralTOF JMS-S3000) was used to measure the mass spectra of all compounds. For manual chromatography, silica gels PSQ-60B (60 μm) and PSQ-100B (110 μm) (Fuji Silysia Chemical) were used. For automated flash chromatography, Hi-Flash™ column silica gel 40 μm (Yamazen) and Purif-Pack^®^-EX NH-50 μm (Shoko Science) were used at Smart Flash EPCLC W-Prep 2XY (Yamazen). The reaction progress was monitored by analytical thin-layer chromatography on glass plates (TLC Silica gel 60 F254) (Merck), and the products were visualized by UV light. For details on the synthetic procedure of GuNA phosphoramidites, see Supporting Information.

### Synthesis and purification of GuNA[R]-modified oligonucleotides (ODN1–ODN3, ODN8–ODN10)

Synthesis of oligonucleotides modified with GuNA[R] was performed using the nS-8 oligonucleotide synthesizer (GeneDesign, Inc.) and the standard phosphoramidite protocol. Custom Primer Support™ T 40s (GE Healthcare) was used as solid support in a 0.2 μmol scale. The amidite was dehydrated and solved in acetonitrile at 0.1 M. The standard synthesis cycle was used to assemble the reagents, except that the coupling time was extended from 25 sec to 16 min for GuNA[R]. We used 0.25 M 5-ethylthio-1*H*-tetrazole (ETT) in acetonitrile as an activator. The synthesis was carried out in trityl-on mode. The elongated oligonucleotides were treated with a 1:1 mixture of 7 N ammonia solution in methanol and 40% aq. methylamine at room temperature for 4 h to remove the solid support, and then the mixture was heated at 60°C for 10 h. After deprotection, oligonucleotides were purified using Sep-Pak^®^ Plus C18 Cartridge, and 5′-dimethoxytrityl group was removed with 2% aq. trifluoroacetic acid on the cartridge. Subsequently, desired oligonucleotides were further purified using reverse-phase HPLC with Waters XBridge™ C18 (4.6 × 50 mm analytical and 10 mm × 50 mm preparative) columns, with a linear gradient of acetonitrile in 0.1 M triethylammonium acetate buffer (pH 7.0). The purity and structure of the oligonucleotides were confirmed by HPLC and MALDI-TOF mass spectroscopy, respectively.

### Synthesis and purification of GuNA[R,R]-modified oligonucleotides (ODN4–ODN7, ODN11–ODN18 and ODN24–ODN33)

Synthesis of oligonucleotides modified with GuNA[R,R] was performed using the nS-8 oligonucleotide synthesizer (GeneDesign, Inc.) and the standard phosphoramidite protocol. Primer Support™ 5G T 350 (GE Healthcare) was used as solid support in a 1.0 μmol scale. The amidite was dehydrated and solved in dichloromethane at 0.125 M for GuNA[R,R]-T, or 0.1 M for GuNA[R,R]-A, -G and -^m^C. The standard synthesis cycle was used to assemble the reagents, except that the coupling time was extended from 25 sec to 20 min for GuNA[R,R]. We used 0.4 M 5-benzylthio-1*H*-tetrazole (BTT) in acetonitrile as an activator, and 50 g/l *tert*-butylphenoxyacetic anhydride (Tac_2_O) in tetrahydrofuran was used as a capping reagent. Oxidizing steps were performed in an iodide solution for 10 s (**ODN4**–**ODN7**, **ODN11**, **ODN12**, **ODN24**–**ODN29**) or 0.5 M (1*S*-+-10-camphorsulfonyl)-oxaziridine (CSO) in tetrahydrofuran for 3 min (**ODN13**–**ODN18**, **ODN30**–**ODN33**). The synthesis was carried out in the trityl-on mode. For **ODN4**–**ODN7**, **ODN11**–**ODN18** and **ODN24**–**ODN27**, the elongated oligonucleotides were treated with a 1:1 mixture of 38 wt.% methylamine solution in ethanol and 40% aq. methylamine at room temperature for 20 min to remove the solid support, and then the mixture was heated at 60°C for 4 h. For **ODN28**, **ODN30** and **ODN32**, the oligonucleotides were treated with 0.4 M sodium hydroxide solution in a 4:1 mixture of methanol and water at room temperature for 17 h, followed by 5% aq. acetic acid to stop the reaction. For **ODN29**, **ODN31** and **ODN33**, the oligonucleotides were treated with a 1:1 mixture of 7 M methylamine solution in methanol and 28% aq. ammonium hydroxide at room temperature for 4 h to remove the solid support, and then the mixture was heated at 60°C for 8 h. After deprotection, crude oligonucleotides were purified using Sep-Pak^®^ Plus C18 Cartridge, and the 5′-dimethoxytrityl group was removed with 2% aq. trifluoroacetic acid on the cartridge. Subsequently, desired oligonucleotides were further purified using reverse-phase HPLC with Waters XBridge™ C18 (4.6 × 50 mm analytical and 10 mm × 50 mm preparative) columns, with a linear gradient of acetonitrile in 0.1 M triethylammonium acetate buffer (pH 7.0). The purity and structure of the oligonucleotides were confirmed by HPLC and MALDI-TOF mass spectroscopy, respectively. The following oligonucleotides were synthesized by GeneDesign, Inc.: **ODN19**, **ODN21**–**ODN23** and **ODN34**. The following oligonucleotides were prepared previously by the authors of this paper: **ODN20**, **ODN35** and **ODN36**.

### UV melting experiments

The UV melting experiments were carried out using SHIMADZU UV-1650PC and SHIMADZU UV-1800 spectrometers equipped with a *T*_m_ analysis accessory. Equimolecular amounts of the target ssRNAs or ssDNAs and oligonucleotides were dissolved in 10 mM sodium phosphate buffer (pH 7.2) containing 100 mM NaCl to achieve a final strand concentration of 4 μM each. The samples were annealed by heating at 100°C followed by slow cooling to 5°C. The melting profile was recorded at 260 nm from 5 to 90°C at a scan rate of 0.5°C/min. *T*_m_ values were taken as the temperatures at which the formed duplexes were half dissociated, determined by the sigmoidal melting curves.

### Thermodynamic analysis

The UV melting experiments were carried out using SHIMADZU UV-1650PC and SHIMADZU UV-1800 spectrometers equipped with a *T*_m_ analysis accessory. Equimolecular amounts of the target ssRNAs or ssDNAs and oligonucleotides were dissolved in 10 mM sodium phosphate buffer (pH 7.2) containing 100 mM NaCl to achieve final strand concentrations of 0.86, 1.47, 2.43, 4.00, 6.60 and 10.9 μM. The samples were annealed by heating at 100°C followed by slow cooling to room temperature. The melting profile was recorded using the method described in ‘UV melting experiments’. The van’t Hoff plots were prepared using the *T*_m_ values in each concentration. For the calculation of Δ*H*°, Δ*S*° and Δ*G*°, the temperature was assumed to be 25°C (298.15 K).

### Crystallization

#### Preparation of crystals

Crystallization conditions were screened with the Nucleic Acid Mini Screen™ kit (Hampton Research) using the hanging drop vapor diffusion technique. Droplets consisting of a mixture of 1 mM oligonucleotide solution and the mini-screen buffer (1:1 or 1:2 v/v) were equilibrated against 1 ml of 35% v/v 2-methyl-2,4-pentanediol (MPD) aqueous solution. A crystal suitable for the diffraction experiment was obtained from a droplet containing 1 μl of 1 mM oligonucleotide and 1 μl of buffer #12, #14 and #10 for **ODN25**, **ODN28** and **ODN29**, respectively. The crystal was mounted in a nylon loop and frozen in liquid nitrogen with the reservoir solution as a cryoprotectant.

#### X-ray data collection and refinement

For **ODN25**, the crystal was diffracted in a Rigaku MicroMax-007HF with R-axis IV++ using copper radiation. The diffraction data were processed with Mosflm and scaled with Aimless. The initial structure was determined by the molecular replacement method using the single-stranded 10 mer nucleotide containing 2′,4′-BNA/LNA modification (PDB ID: 1I5W) (35) as a template model. Rotation and translation searches for molecular replacement were performed by PHASER. Model refinement was performed with REFMAC. For **ODN28** and **ODN29**, X-ray diffraction data were collected at SPring-8 (Hyogo, Japan) with a beamline BL44XU equipped with an EIGER X 16M. One of the crystals was scanned to find the one with strong anomalous scattering at the K-edge absorption of bromine. The XDS software was used for data processing. The initial phases were determined with single-wavelength anomalous dispersion using the hkl2map program of SHELX. The atomic models were built using the molecular graphics program COOT and refined with Phenix. Geometric restraint library files of GuNA[Me,*^t^*Bu] and GuNA[Me,Me] monomers were produced with the CCP4 programs Monomer Library Sketcher and Libcheck. The statistics for data collection and refinement are summarized in Supplementary Tables S6–S8.

### Nuclease resistance study

The sample solutions were prepared by dissolving 0.75 nmol of oligonucleotides in 50 mM Tris-HCl buffer (pH 8.0) containing 10 mM MgCl_2_ (sample volume: 100 μL). In each sample solution, same amount (0.03 or 0.12 μg) of phosphodiesterase I from *Crotalus adamanteus* venom (svPDE) was added, and the cleavage reaction was carried out at 37°C. A portion of each reaction mixture was removed at timed intervals and heated to 90°C for 2 min to deactivate the nuclease. Aliquots of the timed samples were analyzed by RP-HPLC to evaluate the amounts of intact oligonucleotides remaining. The percentage of intact oligonucleotides in each sample was calculated and plotted against the digestion time to obtain a degradation curve with time.

## RESULTS AND DISCUSSION

### Synthesis of the amidite blocks of GuNA analogs

**Scheme 1.**
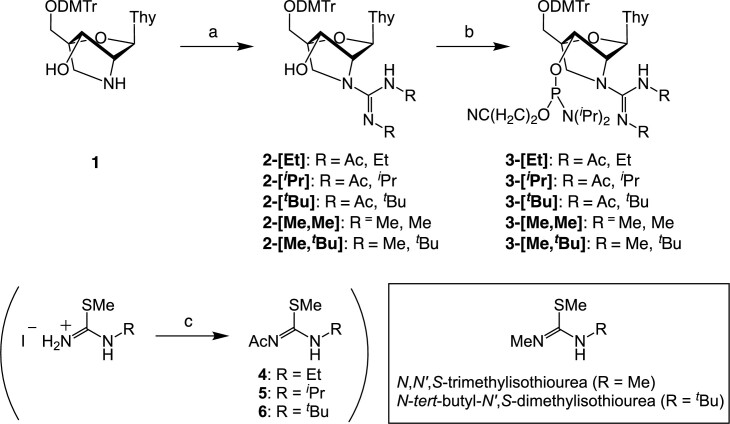
Synthesis of GuNA[R]-T and GuNA[R,R]-T phosphoramidites. Reagents and conditions: (a) *N*-ethyl-*N′*-acetyl-*S*-methylisothiourea (**4**), AgOTf, DIPEA, THF, rt, 89% for **2-[Et]**; *N*-isopropyl-*N*′-acetyl-*S*-methylisothiourea (**5**), AgOTf, DIPEA, THF, rt, 96% for **2-[*^i^*Pr]**; *N*-*tert*-butyl-*N*′-acetyl-*S*-methylisothiourea (**6**), AgOTf, DIPEA, THF, rt, 87% for **2-[*^t^*Bu]**; *N,N′,S*-trimethylisothiourea, AgOTf, DIPEA, DMA, 90°C, 83% for **2-[Me,Me]**; *N*-*tert*-butyl-*N*′*,S*-dimethylisothiourea, AgOTf, DIPEA, DMA, 90°C, 98% for **2-[Me,*^t^*Bu]**; (b) 2-cyanoethyl *N,N*-diisopropylchlorophosphoramidite, DIPEA, CH_2_Cl_2_, rt, 83% for **3-[Et]**, 71% for **3-[*^i^*Pr]**, 48% for **3-[*^t^*Bu]**, 98% for **3-[Me,Me]**, 86% for **3-[Me,*^t^*Bu]**; (c) Ac_2_O, TEA, CH_2_Cl_2_, rt, 84% for **4**, 93% for **5**, 97% for **6**; T = thymin-1-yl.

Five GuNA[R] and GuNA[R,R] phosphoramidites (**3-[Et]**, **3-[*^i^*Pr]**, **3-[*^t^*Bu]**, **3-[Me,Me]** and **3-[Me,*^t^*Bu]**) bearing a thymine (T) nucleobase, shown in Scheme 1, were newly designed for incorporation into oligonucleotides. Before the synthesis of GuNA[R] and GuNA[R,R] phosphoramidites, guanidinylation reagents **4**–**6** were synthesized from the corresponding *N*-alkylated isothioureas (36–38). Starting from 5′-*O*-(4,4′-dimethoxytrityl)-2′-amino-LNA-T (**1**), a common precursor for GuNA analogs (31,32,39,40), the synthesis of GuNA[R]-T phosphoramidites (**3-[Et]**, **3-[*^i^*Pr]** and **3-[*^t^*Bu]**) was accomplished in two steps. At first, the 2′-amino group of compound **1** was converted into *N*-alkylated guanidines by using guanidinylation reagents **4**–**6**. The remaining 3′-hydroxyl groups of **2-[Et]**, **2-[*^i^*Pr]** and **2-[*^t^*Bu]** were then phosphitylated with 2-cyanoethyl *N*,*N*-diisopropylchlorophosphoramidite to afford the desired products. The GuNA[R,R]-T phosphoramidites, **3-[Me,Me]** and **3-[Me,*^t^*Bu]**, were similarly synthesized. By treatment with *N,N′,S*-trimethylisothiourea (41) or *N*-*tert*-butyl-*N′,S*-dimethylisothiourea (42) in DMA, the 2′-amino group of **1** was converted into the corresponding *N,N′*-dialkylguanidine moiety. Subsequent phosphitylation of the 3′-hydroxyl group with 2-cyanoethyl *N,N*-diisopropylchlorophosphoramidite yielded the desired phosphoramidites. Since there were no remarkable side reactions in the phosphitylation step of **2-[Me,Me]** and **2-[Me,*^t^*Bu]**, their *N,N′*-dialkylguanidine moieties were unprotected.

### Synthesis of GuNA[R]- and GuNA[R,R]-modified oligonucleotides

Using an automated DNA synthesizer (solid-phase synthesis), the given phosphoramidites were incorporated into oligonucleotides in the same manner as the previous method (32) (Table 1). After elongation reactions, the oligonucleotides were cleaved from the solid support. At the same time, the protective groups at the nucleobases and the phosphate backbone were removed by treatment with an ammonia/methylamine solution (7 M ammonium solution in methanol/40% aqueous methylamine solution = 50:50 v/v) at 60°C for 10 h. This method provided GuNA[Et]- and GuNA[*^i^*Pr]-modified oligonucleotides (**ODN1** and **ODN2**) at 13% and 22%, respectively. However, GuNA[*^t^*Bu]-modified **ODN3** was isolated at a low yield (2%). The remaining acetyl group at the guanidine moiety was observed by LC-MS analysis of the crude material (see Supplementary Figure S1 in Supporting Information), which indicated that the nearby *tert*-butyl group sterically inhibited the deprotection of the acetyl group. In the preparation of GuNA[R,R]-modified oligonucleotides (**ODN4**–**ODN7**), condition optimization was required because the guanidine moieties of **3-[Me,Me]** and **3-[Me,*^t^*Bu]** monomers were unprotected. We observed acetylation of the dialkylguanidine moiety by acetic anhydride, the capping reagent used in the solid-phase synthesis, and removing the acetyl group was quite difficult (see Supplementary Figure S2 in Supporting Information). Therefore, we used 4-*tert*-butylphenoxyacetic anhydride (Tac_2_O) as a labile capping reagent. This successfully reduced the production of guanidine-acylated oligonucleotides (see Supplementary Figure S3 in Supporting Information). In addition, 5-(ethylthio)-*1H*-tetrazole (ETT) and iodine, which could be affected by the basic guanidine moiety, were replaced with BTT and CSO (see Supplementary Tables S1 and S2 in Supporting Information). These changes successfully enabled us to obtain enough GuNA[R,R]-modified oligonucleotides for the following evaluations. The isolated yields of **ODN4**–**ODN7** were 2–24%, as shown in Table 1.

**Table 1.** Synthetic yields and MALDI-TOF mass data of oligonucleotides **ODN1**–**ODN7**

				MALDI-TOF mass [M–H]^–^	
Name	Oligonucleotides (5′–3′)	Modification (T = )	Yields [%]	Calcd.	Found
**ODN1**	d(GCGTTTTTTGCT)	GuNA[Et]	13	3729.5	3729.6
**ODN2**		GuNA[*^i^*Pr]	22	3743.5	3743.7
**ODN3**		GuNA[*^t^*Bu]	2	3757.5	3758.1
**ODN4**		GuNA[Me,Me]	24	3729.5	3728.6
**ODN5**		GuNA[Me,*^t^*Bu]	22	3771.6	3771.4
**ODN6**	d(GCGTTTTTTGCT)	GuNA[Me,Me]	5	3923.7	3923.6
**ODN7**		GuNA[Me,*^t^*Bu]	2	4050.0	4050.7

### Duplex-forming ability of GuNA[R]- and GuNA[R,R]-modified oligonucleotides

We next evaluated the duplex-forming ability of the GuNA[R]- and GuNA[R,R]-modified oligonucleotides toward the complementary RNA or DNA strand (Table 2). As mentioned in the Introduction section, GuNA[Me] modification increased the duplex stability with the Δ*T*_m_ (deference in melting temperature compared to the control oligodeoxynucleotide, **ODN19**) by + 5°C (see the results of **ODN20**). Notably, we observed significantly increased *T*_m_ values of the GuNA[*^t^*Bu]- and GuNA[Me,*^t^*Bu]-modified oligonucleotides (**ODN3** and **ODN5**) for both RNA and DNA strands. Moreover, the *T*_m_ values increased significantly with the increasing size of the alkyl group at the guanidine moiety (see **ODN20**, **ODN1**, **ODN2** and **ODN3** toward ssRNA). Furthermore, tandem modification with GuNA[Me,*^t^*Bu] greatly increased the *T*_m_ compared to GuNA[Me,Me] (**ODN6** versus **ODN7**). These results indicated that the bulky alkyl group on the guanidine moiety played an essential role in increasing duplex stability, and the tandem incorporation of a bulky substituent did not reduce the duplex stability.

**Table 2.** *T*_m_ Values of the duplexes formed between GuNA[R]- or GuNA[R,R]-modified oligonucleotides and the complementary strands^a^

			*T*_m_ (Δ*T*_m_/mod.) [°C]			
Name	Oligonucleotides (5′–3′)	Modification (T = )	vs. ssRNA		vs. ssDNA	
**ODN19**	d(GCGTTTTTTGCT)	–	47		51	
**ODN20** ^b^	d(GCGTTTTTTGCT)	GuNA[Me]	52	(+5)	56	(+5)
**ODN1**		GuNA[Et]	53	(+6)	N.D.	
**ODN2**		GuNA[*^i^*Pr]	54	(+7)	N.D.	
**ODN3**		GuNA[*^t^*Bu]	55	(+8)	59	(+8)
**ODN4**		GuNA[Me,Me]	51	(+4)	55	(+4)
**ODN5**		GuNA[Me,*^t^*Bu]	56	(+9)	60	(+9)
**ODN6**	d(GCGTTTTTTGCT)	GuNA[Me,Me]	58	(+3.7)	59	(+2.7)
**ODN7**		GuNA[Me,*^t^*Bu]	70	(+7.7)	74	(+7.7)

^a^Conditions: 10 mM sodium phosphate buffer (pH 7.2), 100 mM NaCl, 4 μM each oligonucleotide, 0.5°C/min at 260 nm. Target strands: 5′-r(AGCAAAAAACGC)-3′ and 5′-d(AGCAAAAAACGC)-3′. The *T*_m_ values reflect the average of three measurements. Δ*T*_m_/mod.: change in *T*_m_ value (Δ*T*_m_) per modification compared to the unmodified oligonucleotide (**ODN19**). ^b^Reference 32. N.D. means not determined.

We further evaluated the thermodynamic parameters of the duplexes formed between the GuNA-modified oligonucleotides and the complementary ssRNA (Table 3, see also Supplementary Figure S4 for van’t Hoff plots). Compared to the GuNA[Me]-modified oligonucleotide (**ODN20**), **ODN2**, **ODN3** and **ODN5** bearing bulkier substituents were found to stabilize the duplex enthalpically, suggesting a positive energy gain by increased nucleobase stacking, hydrogen-bond formation, and/or other electrostatic interactions.

**Table 3.** Thermodynamic parameters of duplex formation between GuNA[R]- or GuNA[R,R]-modified oligonucleotides and the complementally ssRNA^a^

Name	Oligonucleotides (5′–3′)	Modification (T = )	Δ*H*° [kcal•mol^−1^]	Δ*S*° [cal•(K•mol)^–1^]	Δ*G*° [kcal•mol^−1^]
**ODN20** ^b^	d(GCGTTTTTTGCT)	GuNA[Me]	–70.8	–191.3	–13.8
**ODN2**		GuNA[*^i^*Pr]	–77.2	–209.7	–14.7
**ODN3**		GuNA[*^t^*Bu]	–104.3	–291.4	–17.4
**ODN5**		GuNA[Me,*^t^*Bu]	–104.1	–290.5	–17.4

^a^Conditions: 10 mM sodium phosphate buffer (pH 7.2), 100 mM NaCl, 0.89–10.9 μM each oligonucleotide, 0.5°C/min at 260 nm. Target strands: 5′-r(AGCAAAAAACGC)-3′. These values were determined by van’t Hoff plots with six data points. The Δ*G*° value was calculated at 298.15 K (25°C). ^b^Reference 32.

### X-ray crystallographic analysis of the duplexes formed by GuNA[R,R]-modified oligonucleotides

**Figure 3.**
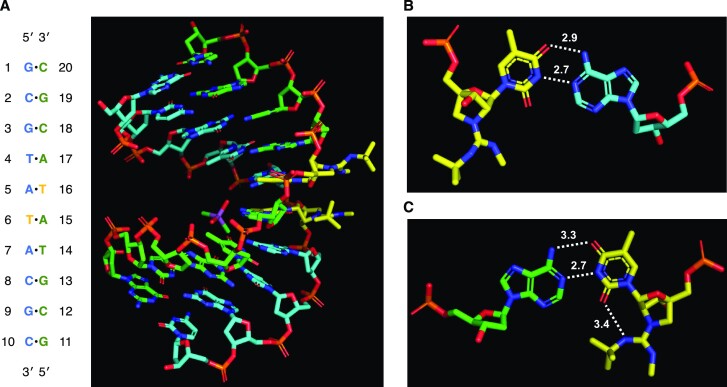
X-ray crystal structure of antiparallel 10 mer oligonucleotide duplex having GuNA[Me,*^t^*Bu] (**ODN25**). (**A**) Overall structure of the duplex. Carbon atoms of sense and antisense strands are shown in cyan and green, respectively. Carbon atoms of GuNA[Me,*^t^*Bu] are shown in yellow. Arsenic and carbon atoms of cacodylic acid are shown in purple and green, respectively. (B, C) Enlarged views of the GuNA[Me,*^t^*Bu]-modified positions. Base pairs containing GuNA[Me,*^t^*Bu]-T, GuNA[Me,*^t^*Bu]-T16•dA5 (**B**) and GuNA[Me,*^t^*Bu]-T6•dA15 (**C**), are shown with hydrogen bonds. The hydrogen bond (*N*–*O* and *N*–*N*) distances are shown by the angstrom unit (Å) with dotted lines.

**Figure 4.**
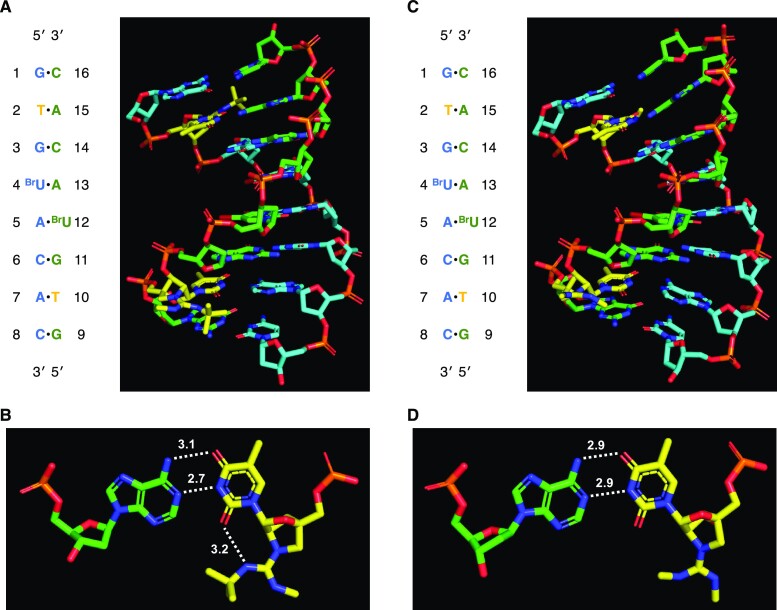
X-ray crystal structures of antiparallel 8 mer oligonucleotide duplexes having either GuNA[Me,*^t^*Bu] (**ODN29**) or GuNA[Me,Me] (**ODN28**). (**A**) Overall structure of the duplex having GuNA[Me,*^t^*Bu]. (**B**) Enlarged view of the GuNA[Me,*^t^*Bu]-T2•dA15 base pair. The hydrogen bond (*N*–*O* and *N*–*N*) distances are shown by the angstrom unit (Å) with dotted lines. (**C**) Overall structure of the duplex having GuNA[Me,Me]. (**D**) Enlarged view of the GuNA[Me,Me]-T2•dA15 base pair. Carbon atoms of sense and antisense strands are shown in cyan and green, respectively. Carbon atoms of GuNA[R,R] are shown in yellow.

Based on the above results, we speculated that the spatial arrangement of the *tert*-butyl group at the guanidine moiety in the duplex structure is crucial for increased duplex stability. To clarify this, we conducted an X-ray crystallographic analysis of GuNA[R,R]-modified oligonucleotides. Two self-complementary sequences were selected; (1) a 10 mer oligodeoxynucleotide (5′-GCGTATACGC-3′)(35) and (2) an 8 mer oligodeoxynucleotide (5′-GTG^Br^UACAC-3′), where ^Br^U represents a 5-bromourasil nucleobase(43)—these sequences have been used previously for crystallographic analysis. We prepared ten oligonucleotides bearing either GuNA[Me,Me] or GuNA[Me,*^t^*Bu] modifications at different positions (see Supplementary Table S3 in Supporting Information). Among them, three oligonucleotides, **ODN25**, **ODN28** and **ODN29** (Table 4), formed good crystals for X-ray crystallographic analysis, and their crystal structures were successfully determined at the resolutions of 2.01, 0.95 and 0.93 Å, respectively (Figures 3 and 4). All three crystal structures were found to adopt the A-form duplex.

**Table 4.** Oligonucleotides used for X-ray crystallographic analysis^a^

Name	Oligonucleotides (5′–3′)	Modification (T = )
**ODN25**	d(GCGTATACGC)	GuNA[Me,*^t^*Bu]
**ODN28**	d(GTG^Br^UACAC)	GuNA[Me,Me]
**ODN29**	d(GTG^Br^UACAC)	GuNA[Me,*^t^*Bu]

^a^Underlined text in the sequence indicates modified nucleic acid.

Intriguingly, two GuNA[Me,*^t^*Bu]s in the duplexed **ODN25** formed different structures from each other (Figure 3). The *tert*-butyl group of one was located at the minor groove side (see the GuNA[Me,*^t^*Bu]-T6•dA15 base pair in Figure 3A, and C), whereas that of the other one was located outside of the duplex (see the GuNA[Me,*^t^*Bu]-T16•dA5 base pair in Figure 3A and B). The results could be explained by a steric repulsion of the two *tert*-butyl groups because the GuNA[Me,*^t^*Bu]s were located close together in the duplexed **ODN25**. This means that one of the *tert*-butyl groups at the minor groove exposed another *tert*-butyl group to the outside. On the other hand, two *tert*-butyl groups of the duplexed **ODN29** were located on the minor groove side, and two GuNA[Me,*^t^*Bu]s were found to have similar structures (see Figure 4A and B). Notably, we found that the *tert*-butyl-substituted guanidine moiety and the 2-carbonyl group of the thymine nucleobase formed a hydrogen bond (3.4 Å for **ODN25** in Figure 3C and 3.2 Å for **ODN29** in Figure 4B), suggesting that this additional hydrogen-bond contact, as well as the hydrophobic interactions between the *tert*-butyl group and the minor groove, contributed greatly to duplex stability. In contrast, additional hydrogen-bond contacts were not observed in the duplex formed by the GuNA[Me,Me]-modified **ODN28** (Figure 4C and D). In these three X-ray crystal structures, no counter anion was observed around the guanidium part.

We further evaluated the *T*_m_ values of the crystalized self-complementary oligonucleotides, **ODN25**, **ODN28** and **ODN29** (Table 5). Here, *T*_m_ values of the related oligonucleotides (**ODN34**, **ODN24**, **ODN26** and **ODN27**) are also shown. Compared to the GuNA[Me,Me]-modified **ODN24**, GuNA[Me,*^t^*Bu]-modified **ODN25**—in which one *tert*-butyl group flipped to the outside of the duplex—showed only a one-degree increase in the *T*_m_ value. On the other hand, **ODN27** containing GuNA[Me,*^t^*Bu] at a different position on the same sequence showed a much higher *T*_m_ than the corresponding GuNA[Me,Me]-modified **ODN26**. We propose that, in the case of **ODN27**, both *tert*-butyl groups were placed at the minor groove because they were separated, resulting in noticeable stabilization of the duplex (Δ*T*_m_/mod. = +24.5°C). Similarly, **ODN29** bearing GuNA[Me,*^t^*Bu] at the second position from the 5′-end exhibited a much higher *T*_m_ (55°C) than the corresponding GuNA[Me,Me]-modified oligonucleotide (44°C for **ODN28**).

**Table 5.** *T*_m_ values of the duplexes formed between GuNA[R,R]-modified oligonucleotides and the complementary ssRNA^a^

Name	Oligonucleotides (5′–3′)	Modification (T = )	*T*_m_ (Δ*T*_m_/mod.) [°C]	
**ODN34**	d(GCGTATACGC)	–	27	
**ODN24**	d(GCGTATACGC)	GuNA[Me,Me]	50	(+11.5)
**ODN25**		GuNA[Me,*^t^*Bu]	51	(+12.0)
**ODN26**	d(GCGTATACGC)	GuNA[Me,Me]	63	(+18.0)
**ODN27**		GuNA[Me,*^t^*Bu]	76	(+24.5)
**ODN28**	d(GTG^Br^UACAC)	GuNA[Me,Me]	44	
**ODN29**		GuNA[Me,*^t^*Bu]	55	

^a^Conditions: 10 mM sodium phosphate buffer (pH 7.2), 100 mM NaCl, 4 μM each oligonucleotide, 0.5°C/min at 260 nm. The *T*_m_ values reflect the average of three measurements. Δ*T*_m_/mod.: change in *T*_m_ value (Δ*T*_m_) per modification compared to the unmodified oligonucleotide (**ODN34**). ^Br^U = 5-bromo-2′-deoxyuridine.

The extra hydrogen bond, observable in **ODN25** and **ODN28**, would indirectly affect the strengths of the hydrogen bonds of base pairing and base stacking. GuNA[Me,Me] and GuNA[Me,*^t^*Bu] had different hydrogen-bond distances in their T•A base pairs (N3(T)–N1(A): 2.9 Å for GuNA[Me,Me] and 2.7 Å for GuNA[Me,*^t^*Bu]; O4(T)–N6(A): 2.9 Å for GuNA[Me,Me] and 3.1 Å for GuNA[Me,*^t^*Bu]). Shortened N3(T)–N1(A) bonds were observed when the *tert*-butyl group was tightly bound to the minor groove (Figures 3C and 4B). As evaluated in Table 3, GuNA[*^t^*Bu] and GuNA[Me,*^t^*Bu] stabilized the duplex enthalpically. Taken together, the increased duplex stability can be understood by the following three processes: (i) the *tert*-butyl group of GuNA[*^t^*Bu] or GuNA[Me,*^t^*Bu] bound tightly to the minor groove, (ii) an additional hydrogen bond formed, and (iii) the nearby T•A base-pair strengthened. In addition, we compared the X-ray crystal structures of **ODN25** and **ODN29**, containing GuNA[Me,*^t^*Bu], with those of the original sequences (PDB IDs: 1I5W and 8HU5) (35,43) and have confirmed that the overall structures of duplexes containing GuNA[Me,*^t^*Bu] are quite similar to those of the original LNA-modified and unmodified DNA duplexes (see Supplementary Figures S5 and S6 in Supporting Information). Although the X-ray crystal structure is a snapshot, the GuNA[Me,*^t^*Bu] modification is expected to have little effect on the overall duplex structure and the stacking geometries. Using space-filling models, we confirmed that the *tert*-butyl groups fit tight into the minor groove (see Supplementary Figures S5B and S6B in Supporting Information).

We also synthesized GuNA[Me,Me]-A, GuNA[Me,*^t^*Bu]-A, GuNA[Me,Me]-G, GuNA[Me,*^t^*Bu]-G, GuNA[Me,Me]-^m^C and GuNA[Me,*^t^*Bu]-^m^C phosphoramidites (see **Scheme S1** and **Scheme S2** in Supporting Information). Among them, GuNA[Me,Me]-A, GuNA[Me,*^t^*Bu]-A, GuNA[Me,Me]-^m^C and GuNA[Me,*^t^*Bu]-^m^C were successfully incorporated into the designed sequences in the required amounts for evaluation (see Supplementary Table S4 in Supporting Information) and their *T*_m_ values were determined (Table 6). Again, we observed increased *T*_m_ values for GuNA[Me,*^t^*Bu]-A- and GuNA[Me,*^t^*Bu]-^m^C-modified oligonucleotides (**ODN14** and **ODN18**) compared to their GuNA[Me,Me]-modified counterparts (**ODN13** and **ODN17**). However, the duplex stabilizing effect of the *tert*-butyl groups of GuNA[Me,*^t^*Bu]-A and GuNA[Me,*^t^*Bu]-^m^C seemed to be less than that of GuNA[Me,*^t^*Bu]-T (see **ODN5** in Table 2), possibly due to no hydrogen-bond formation between the guanidine moiety and the nucleobase in the GuNA[Me,*^t^*Bu]-A and GuNA[Me,*^t^*Bu]-^m^C nucleosides.

**Table 6.** *T*_m_ Values of the duplexes formed between GuNA[R,R]-modified oligonucleotides and the complementary strands^a^

			*T*_m_ (Δ*T*_m_/mod.) [°C]			
Name	Oligonucleotides (5′–3′)	Modification (A or ^m^C = )	vs. ssRNA		vs. ssDNA	
**ODN22**	d(GCGTTATTTGCT)	–	45		49	
**ODN13**	d(GCGTTATTTGCT)	GuNA[Me,Me]	50	(+5)	53	(+4)
**ODN14**		GuNA[Me,*^t^*Bu]	51	(+6)	54	(+5)
**ODN23**	d(GCGTTCTTTGCT)	–	52		53	
**ODN17**	d(GCGTT^m^CTTTGCT)	GuNA[Me,Me]	59	(+7)	60	(+7)
**ODN18**		GuNA[Me,*^t^*Bu]	60	(+8)	61	(+8)

^a^Conditions: 10 mM sodium phosphate buffer (pH 7.2), 100 mM NaCl, 4 μM each oligonucleotide, 0.5°C/min at 260 nm. Target strands: 5′-r(AGCAAAAAACGC)-3′ and 5′-d(AGCAAAAAACGC)-3′. The *T*_m_ values reflect the average of three measurements. Δ*T*_m_/mod.: change in *T*_m_ value (Δ*T*_m_) per modification compared to the unmodified oligonucleotide (**ODN22** or **ODN23**).

### Mismatch discrimination ability of GuNA[R]- or GuNA[R,R]-modified oligonucleotides

**Figure 5.**
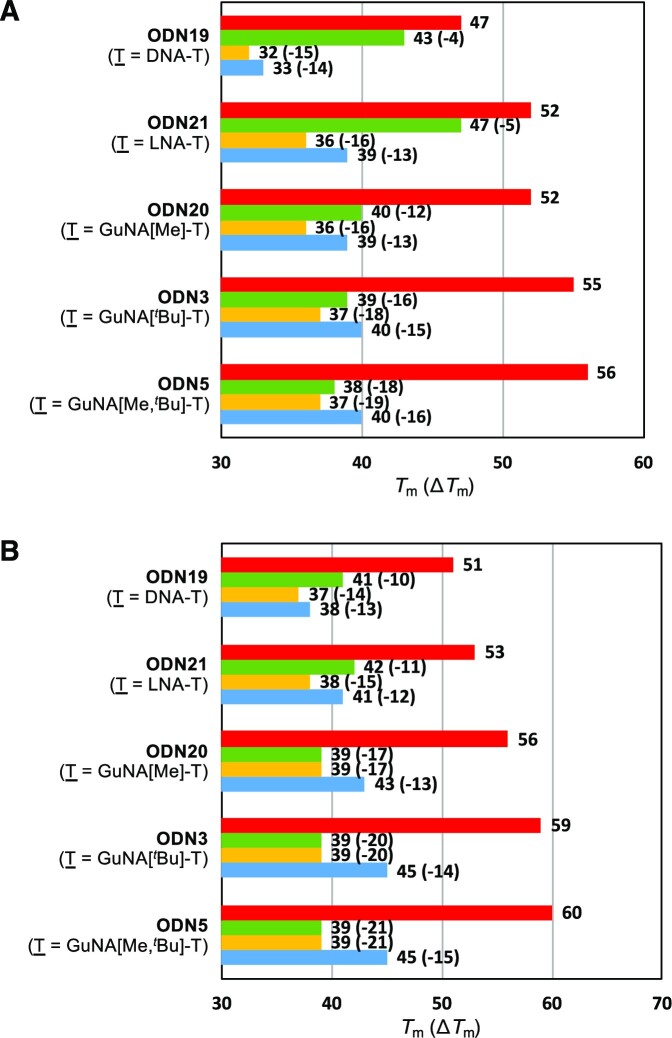
Mismatch discrimination ability of GuNA-modified oligonucleotides toward (**A**) ssRNA and (**B**) ssDNA. The values shown in parentheses are Δ*T*_m_ (*T*_m_ [mismatch] – *T*_m_ [match]). Conditions: 10 mM sodium phosphate buffer (pH 7.2), 100 mM NaCl and 4 μM each oligonucleotide. The sequence of modified oligonucleotides is 5′-d(GCGTTTTTTGCT)-3′. The target RNA and DNA sequences are 5′-r(AGCAAAYAACGC)-3′ and 5′-d(AGCAAAYAACGC)-3′, respectively. Adenine (red bar), guanine (green bar), cytosine (yellow bar) and thymine/uracil (blue bar) nucleobases were incorporated into oligonucleotides at the Y position of the sequence. The *T*_m_ values reflect the average of three measurements. The *T*_m_ values of **ODN19** and **ODN21** were referred from references 44 and 45.

**Figure 6.**
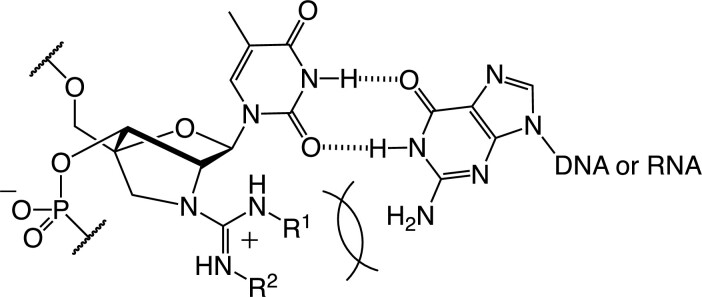
Plausible mechanism for the destabilization of the T•G mismatched wobble base pair formed by GuNA[R] or GuNA[R,R].

Since the *tert*-butyl groups of GuNA[*^t^*Bu] and GuNA[Me,*^t^*Bu] exist at the minor groove in the duplex, the mismatch discrimination ability of these modified nucleic acids should be altered from the other GuNA derivatives. The most common mismatched base pair is the T•G wobble base pair where the 2-amino group of the guanine nucleobase exists at the minor group. We thought that T•G mismatch discrimination would be improved by incorporation of GuNA[*^t^*Bu] and GuNA[Me,*^t^*Bu]. Therefore, we evaluated the mismatch discrimination ability of GuNA[Me]-T-, GuNA[*^t^*Bu]-T- and GuNA[Me,*^t^*Bu]-T-modified oligonucleotides (Figure 5). Here, 2′,4′-BNA/LNA-T-modified **ODN21** was also used as a control, having the bridging structure. As shown in Figure 3, GuNA[Me]-T-modified **ODN20** exhibited a high mismatch discrimination ability compared to the natural **ODN19** (44) and the 2′,4′-BNA/LNA-T-modified **ODN21** (45). The strong destabilization of the T•G mismatched wobble base pair by GuNA[Me] was observed. However, as we had expected, the T•G mismatch discrimination ability was much more significant when the substituent groups at the guanidine moiety became larger (see **ODN3** and **ODN5** in Figure 5); here, the steric repulsion between the 2-amino group of the facing guanine nucleobase and the substituent groups at the guanidine moiety (shown as R^1^ and R^2^ in Figure 6) occurred. Among the modified oligonucleotides evaluated in Figure 5, GuNA[Me,*^t^*Bu]-modified **ODN5** had the best results, having the highest binding affinity toward the full complementary strand (Y = A) and the highest recognition ability toward the mismatched sequences (see Δ*T*_m_ for Y = G, C, T or U).

### Nuclease stability of GuNA[R]- and GuNA[R,R]-modified oligonucleotides

**Figure 7.**
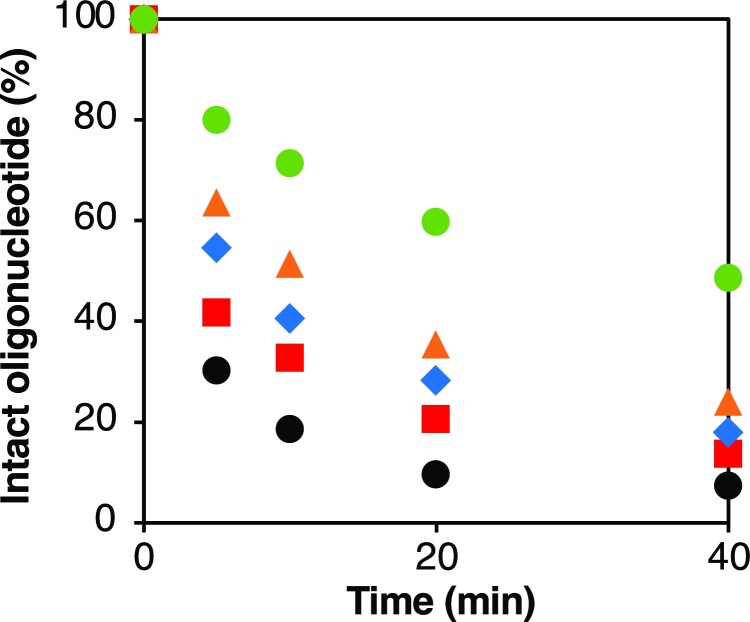
Stability of modified oligonucleotides against 3′-exonuclease. Conditions: 0.03 μg phosphodiesterase I from *Crotalus adamanteus* venom (svPDE), 10 mM MgCl_2_, 50 mM Tris-HCl buffer (pH 8.0), 7.5 μM each oligonucleotide at 37°C (total volume 100 μl). Data points are averages of triplicate experiments. The sequence used in this study was 5′-d(TTTTTTTTTT)-3′. T = GuNA[H] (black circle, **ODN36**), GuNA[Me] (red square, **ODN35**), GuNA[Et] (blue diamond, **ODN8**), GuNA[*^i^*Pr] (orange triangle, **ODN9**) or GuNA[*^t^*Bu] (green circle, **ODN10**).

For application to therapeutic oligonucleotides, the nuclease stability of modified nucleic acids is essential. Therefore, we also evaluated the nuclease stability of GuNA[R]-modified oligonucleotides (Figure 7). Poly T oligonucleotides modified at the second position from the 3′-end were synthesized (Supplementary Table S5 in Supporting Information) and subjected to the stability assay against 3′-exonuclease (svPDE: phosphodiesterase I from *Crotalus adamanteus* venom). As anticipated, oligonucleotides bearing bulkier alkyl groups were found to have higher enzymatic stability (*tert*-butyl > isopropyl > ethyl > methyl > hydrogen). Understandably, the bulkier alkyl groups blocked the access of nuclease more efficiently. We observed similar trends in the GuNA[R,R]-modified oligonucleotides (see Supplementary Figure S7 in Supporting Information), and all GuNA analogs synthesized here were found to increase nuclease stability more than 2′,4′-BNA/LNA.

## CONCLUSION

In conclusion, we synthesized and evaluated a series of GuNA derivatives. We found that the substitution of the guanidine moiety by bulky alkyl groups, such as isopropyl and *tert*-butyl, greatly increased the stability of the duplex formed by GuNA-modified oligonucleotides and the DNA or RNA complement. We also conducted an X-ray crystallographic analysis of GuNA[Me,*^t^*Bu]-modified oligonucleotides, and the given data showed that the *tert*-butyl group bound to the minor groove, and an additional hydrogen bond was formed. These are important factors for the significantly increased duplex-forming ability of GuNA[*^t^*Bu]- and GuNA[Me,*^t^*Bu]-modified oligonucleotides. We believe that the data shown here have strong implications for developing novel types of artificial nucleic acids in the future. We also demonstrated that GuNA[*^t^*Bu]-T- and GuNA[Me,*^t^*Bu]-T-modified oligonucleotides have high mismatch discrimination ability because the *tert*-butyl group destabilizes the generally stable T•G mismatched wobble base pair. Furthermore, GuNA[*^t^*Bu]- and GuNA[Me,*^t^*Bu]-modified oligonucleotides were found to have high nuclease stability. Therefore, GuNA[*^t^*Bu] and GuNA[Me,*^t^*Bu] are good candidates for use in therapeutic oligonucleotides.

## Supplementary Material

gkad608_Supplemental_File

## Data Availability

All relevant datasets that support the findings of this study, including the procedures for monomer synthesis, as well as the compound characterization data (^1^H, ^13^C and ^31^P NMR spectra for all synthesized monomers, the HPLC and MS data for the synthesized oligomers), UV-melting curves and X-ray crystallography data (Supplementary Tables S6–S8) are available in the Supplementary Information. Atomic coordinates and structure factors for the reported crystal structures have been deposited in the Protein Data bank under accession numbers of 8HIS for duplexed **ODN25**, 8I50 for duplexed **ODN28** and 8HU5 for duplexed **ODN29**.
