# Supplementary material for: Mechanism of the extremely high duplex-forming ability of oligonucleotides modified with N-tert-butylguanidine- or N-tert-butyl-N′- methylguanidine-bridged nucleic acids
Source: Nucleic Acids Res. 2023 Jul 18;51(15):7749–61. doi: 10.1093/nar/gkad608 (PMC10450189; doi:10.1093/nar/gkad608)
Supplement: gkad608_Supplemental_File [file gkad608_supplemental_file.pdf]

Supplementary Information

Mechanism of the extremely high duplex-forming ability of oligonucleotides modified with *N-tert*-butylguanidine- or *N-tert*-butyl-*N′*-methylguanidine-bridged nucleic acids

Takao Yamaguchi[1,*], Naohiro Horie[1], Hiroshi Aoyama[1], Shinji Kumagai[2] and Satoshi Obika[1,3,4,*]

[1]Graduate School of Pharmaceutical Sciences, Osaka University, 1-6 Yamadaoka, Suita, Osaka 565-0871, Japan.
[2]Sohyaku. Innovation Research Division, Mitsubishi Tanabe Pharma Corporation, Shonan Health Innovation Park, 2-26-1 Muraoka-Higashi, Fujisawa, Kanagawa 251-8555, Japan.
[3]National Institutes of Biomedical Innovation, Health and Nutrition (NIBIOHN), 7-6-8 Saito-Asagi, Ibaraki, Osaka 567-0085, Japan.
[4]Institute for Open and Transdisciplinary Research Initiatives (OTRI), Osaka University, 1-1 Yamadaoka, Suita, Osaka 565-0871, Japan.

*E-mail: yamaguchi-ta@phs.osaka-u.ac.jp (TY), obika@phs.osaka-u.ac.jp (SO)

Table of Contents

1. Supplementary data
2. Procedures for phosphoramidite synthesis
3. ^{1}H NMR, ^{13}C NMR and ^{31}P NMR spectra of new compounds
4. Characterization data (HPLC and mass data) of synthesized oligonucleotides
5. UV-melting curves
6. X-ray crystallography data

1. Supplementary data

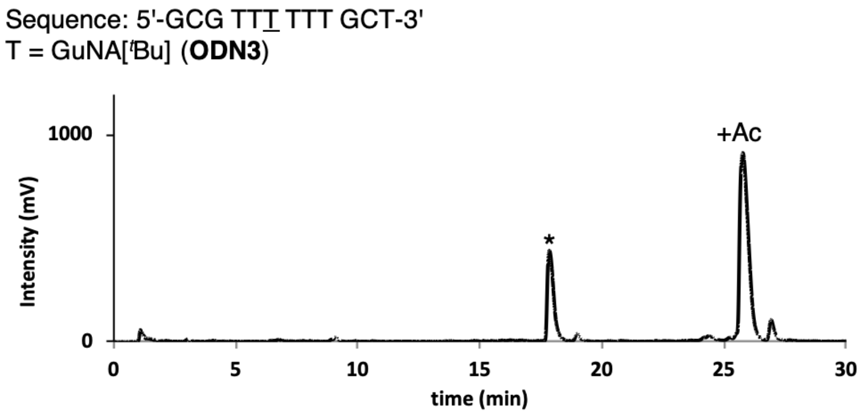

Figure S1. Synthesis of GuNA[^{t}Bu]-modified oligonucleotide (**ODN3**). Synthesized oligonucleotide (crude) was analyzed by reverse-phase UPLC-MS. Asterisk in chart indicates the desired oligonucleotide, and +Ac indicates an acetylated product (at the guanidine). HPLC conditions: Reverse-phase HPLC (Waters XBridge™ $OTSC_{18}$ column, 10 x 50 mm) with linear gradient of acetonitrile (5 to 10% over 30 min) in 0.1 M triethylamine-acetic acid (TEAA) buffer (pH 7.0) as mobile phase (4.0 mL/min).

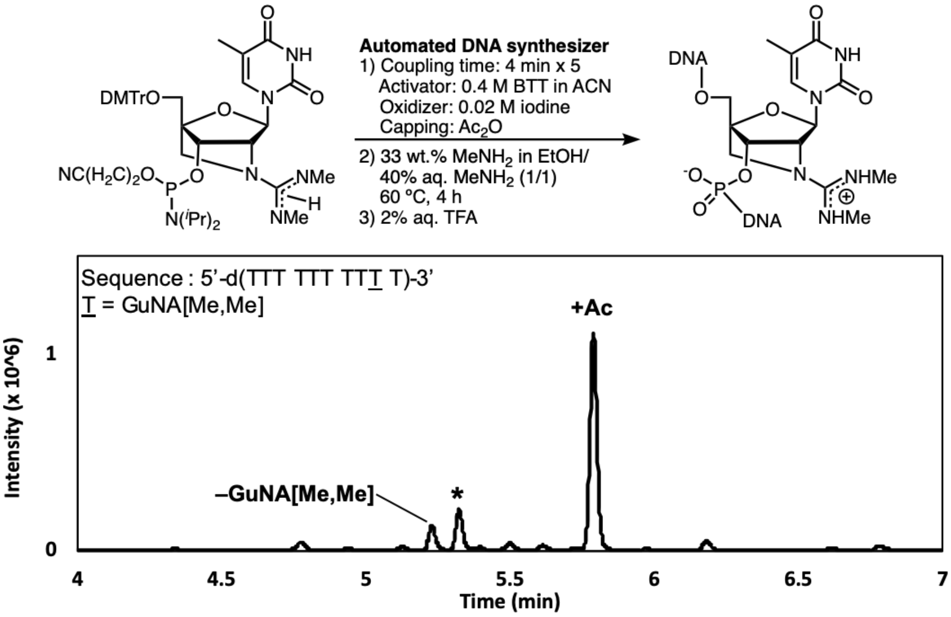

Figure S2. Synthesis of GuNA[Me,Me]-modified oligonucleotide under Ac_2O capping and $MeNH_2$ deprotection conditions. Synthesized oligonucleotide (crude) was analyzed by reverse-phase UPLC-MS. Asterisk in chart indicates the desired oligonucleotide, +Ac indicates an acetylated product (at the guanidine), and –GuNA[Me,Me] indicates n-1 mer oligonucleotide (T9-mer). UPLC conditions: Reversed-phase UHPLC (ACQUITY UPLC® Oligonucleotide BEH C18 Column, 130 Å, 1.7 µm, 2.1 mm x 100 mm, 1/pkg) with linear gradient of methanol (5 to 40% over 9 min) in 1% HFIP-0.1% TEA dissolved in water as mobile phase (0.3 mL/min).

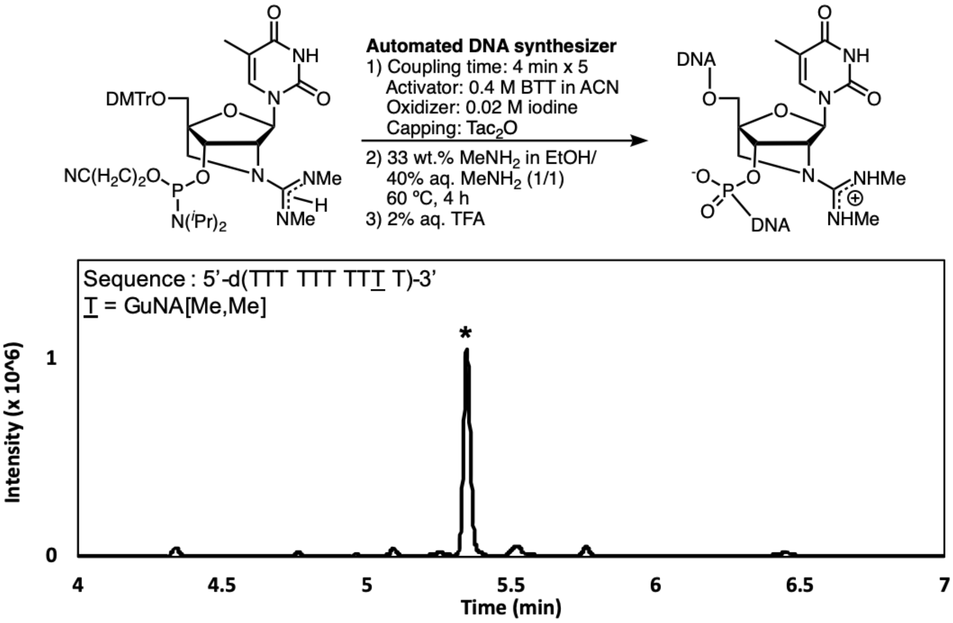

Figure S3. Synthesis of GuNA[Me,Me]-modified oligonucleotide under Tac$_2$O capping and MeNH$_2$ deprotection conditions. Synthesized oligonucleotide (crude) was analyzed by reverse-phase UPLC-MS. Asterisk in chart indicates the desired oligonucleotide. UPLC conditions: Reversed-phase UHPLC (ACQUITY UPLC® Oligonucleotide BEH C18 Column, 130 Å, 1.7 µm, 2.1 mm x 100 mm, 1/pkg) with linear gradient of methanol (5 to 40% over 9 min) in 1% HFIP-0.1% TEA dissolved in water as mobile phase (0.3 mL/min).

Table S1. First optimization of the GuNA[Me,Me]-modified oligonucleotide synthesis[a]

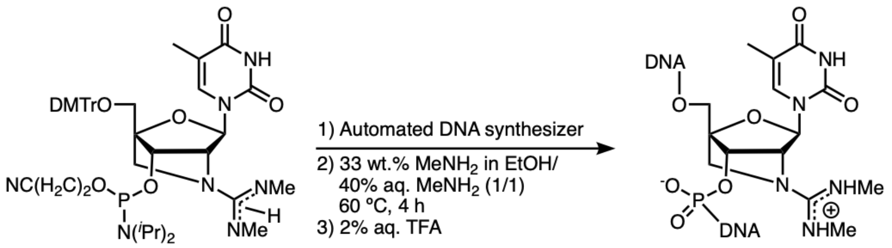

Entry	Oligonucleotide[b] 5'–3'	Activator[c]	Oxidizer[d]	Capping reagent	Yield [%]
1	d(TTT TTT TTT T)	0.25 M DCI	0.02 M iodine	Tac$_2$O	0.8
2		0.25 M ETT			10
3		0.25 M BTT			24
4		0.4 M BTT			33

[*a*] All oligonucleotides were synthesized with a 1 µmol scale cartridge. [*b*] T = GuNA[Me,Me]. [*c*] All activators were dissolved in MeCN. Coupling time for the GuNA[Me,Me] amidite was prolonged to 4 min x 5. [*d*] 0.02 M iodine = 0.02 M iodine/pyridine/H$_2$O/THF, 10 sec.

Table S2. Second optimization of the GuNA[Me,Me]-modified oligonucleotide synthesis[a]

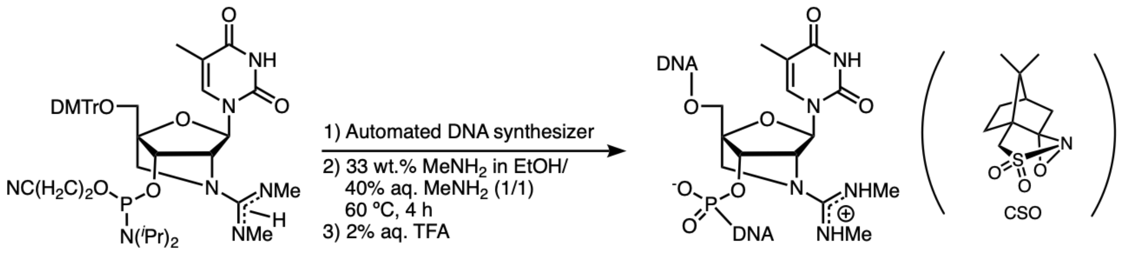

Entry	Oligonucleotide[b] 5'–3'	Activator[c]	Oxidizer[d]	Capping reagent	Yield [%]
1	d(TTT TTT TTT T)	0.4 M BTT	0.02 M iodine	Tac_2O	0.8
2			10% TBHP		10
3			0.5 M CSO		24

[a] All ODNs were synthesized with a 1 µmol scale cartridge. [b] T = GuNA[Me,Me]. [c] BTT was dissolved in MeCN. Coupling time for the GuNA[Me,Me] amidite was prolonged to 4 min x 5. [d] 0.02 M iodine = 0.02 M iodine/pyridine/H_2O/THF, 10 sec. 10% TBHP = 10% TBHP/H_2O/THF, 10 min. 0.5 M CSO = 0.5 M CSO/THF, 3 min.

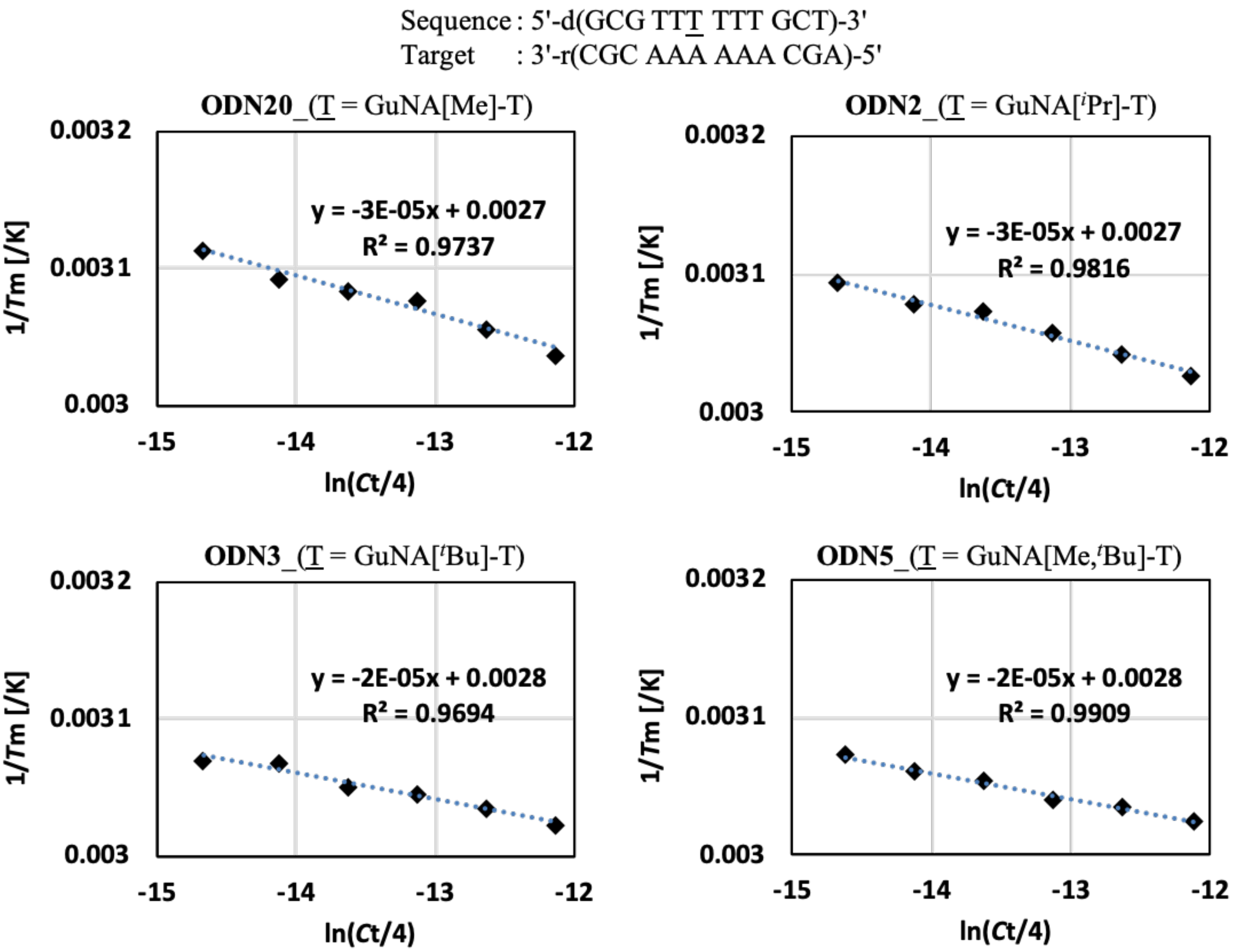

Figure S4. Van't Hoff plots of DNA-RNA duplexes having GuNA analogs.

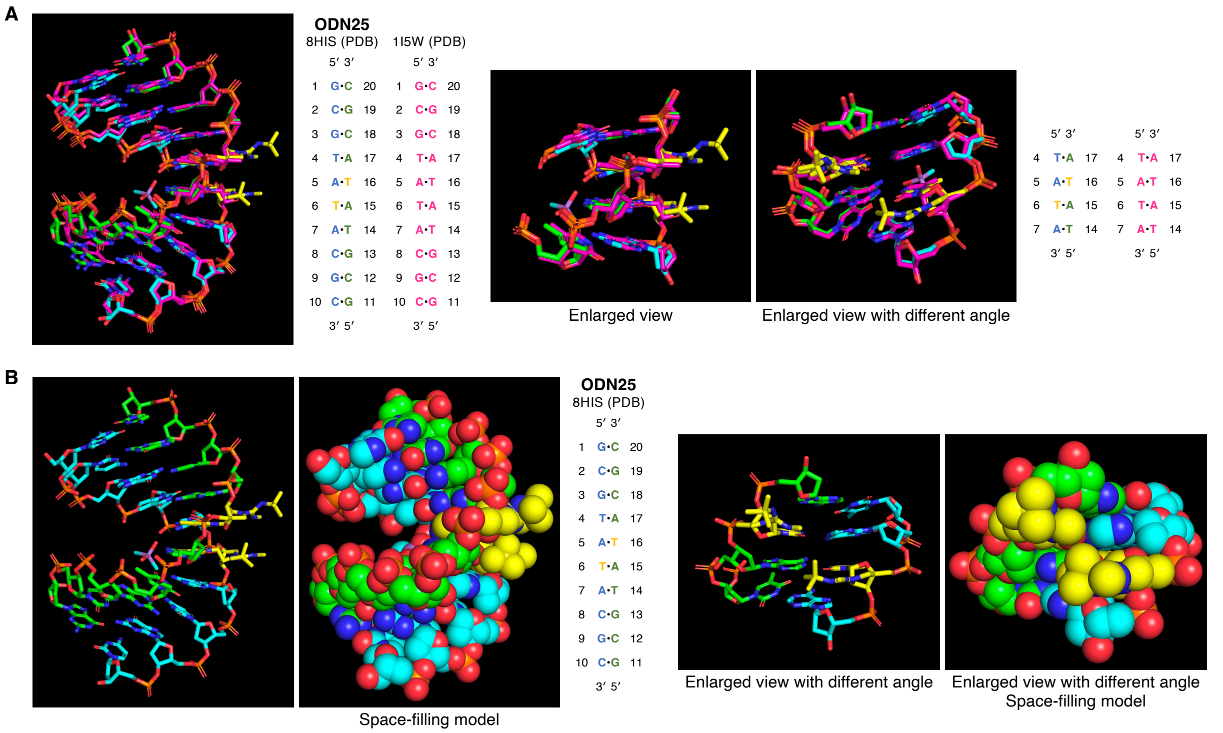

Figure S5. X-ray crystal structure of the antiparallel 10 mer oligonucleotide duplex harboring GuNA[Me,^{t}Bu] (**ODN25**) (PDB ID: 8HIS). (A) Merged images with the X-ray crystal structure of the original LNA-modified DNA sequence (PDB ID: 1I5W) (Egli,M., Minasov,G., Teplova,M., Kumar,R. and Wengel,J. (2001) X-Ray crystal structure of a locked nucleic acid (LNA) duplex composed of a palindromic 10-mer DNA strand containing one LNA thymine monomer. *Chem. Commun.*, 651–652). LNA modifications exist in exactly the same positions with the GuNA[Me,^{t}Bu] modifications (T6 and T16). (B) Space-filling models of the X-ray crystal structure of GuNA[Me,^{t}Bu]-modified **ODN25**.

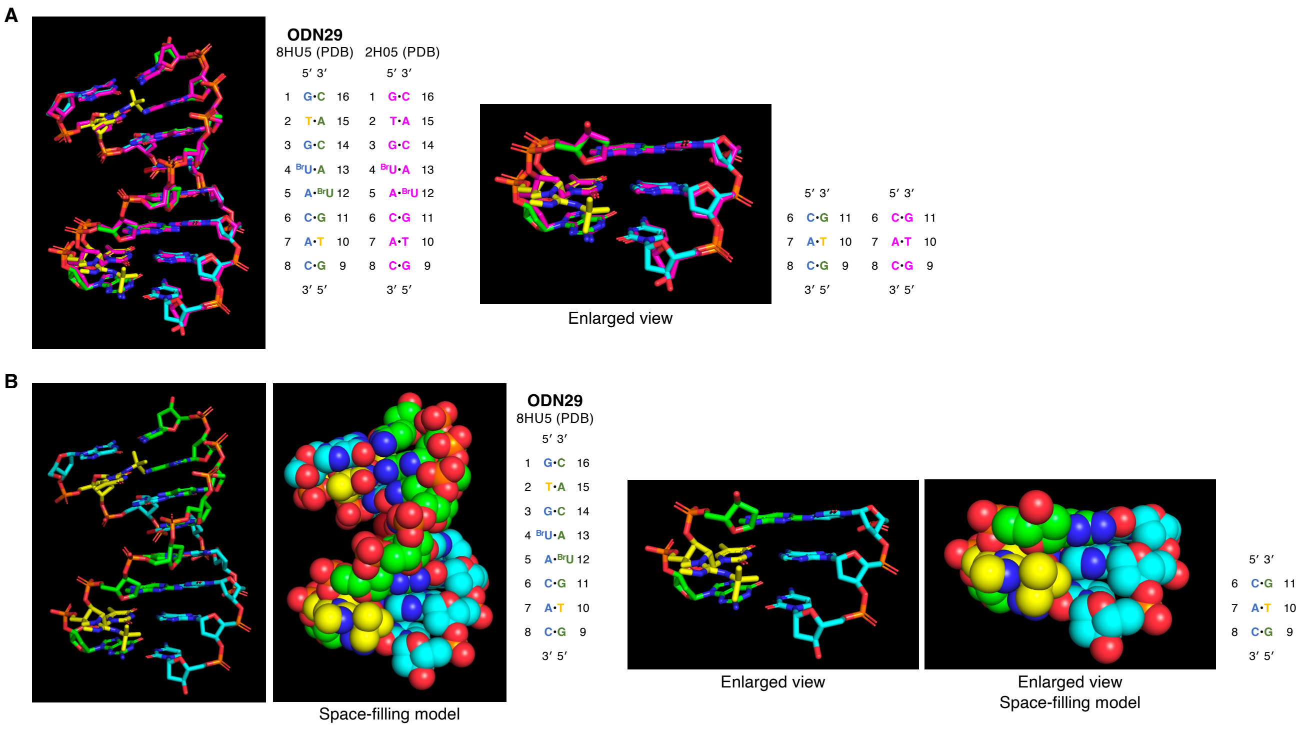

Figure S6. X-ray crystal structure of the antiparallel 8 mer oligonucleotide duplex harboring GuNA[Me,^{t}Bu] (**ODN29**) (PDB ID: 8HU5). (A) Merged images with the X-ray crystal structure of the original DNA sequence (PDB ID: 2H05) (Jiang,J., Sheng,J., Carrasco,N. and Huang,Z. (2007) Selenium derivatization of nucleic acids for crystallography. *Nucleic Acids Res.*, *35*, 477–485). (B) Space-filling models of the X-ray crystal structure of GuNA[Me,^{t}Bu]-modified **ODN29**.

Table S3. Characterization data of oligonucleotides used for crystallization studies[a]

Name	Oligonucleotide 5'–3'	T/^{m}C/A	Yield [%]	MALDI-TOF mass [M–H]$^{-}$ Calcd.	Found
**ODN24**	d(GCG TAT ACG C)	GuNA[Me,Me]	11	3124.1	3124.0
**ODN25**		GuNA[Me,^{t}Bu]	9	3166.2	3165.4
**ODN26**	d(GCG TAT ACG C)	GuNA[Me,Me]	15	3124.1	3124.7
**ODN27**		GuNA[Me,^{t}Bu]	3.8	3166.2	3166.7
**ODN28**	d(GTG ^{Br}UAC AC)	GuNA[Me,Me]	15	2570.6	2571.7
**ODN29**		GuNA[Me,^{t}Bu]	10	2612.7	2612.8
**ODN30**	d(GTG ^{Br}UA^{m}C AC)	GuNA[Me,Me]	7.5	2584.6	2585.1
**ODN31**		GuNA[Me,^{t}Bu]	17	2626.7	2627.2
**ODN32**	d(GTG ^{Br}UAC AC)	GuNA[Me,Me]	10	2570.6	2571.4
**ODN33**		GuNA[Me,^{t}Bu]	9.7	2612.7	2613.5

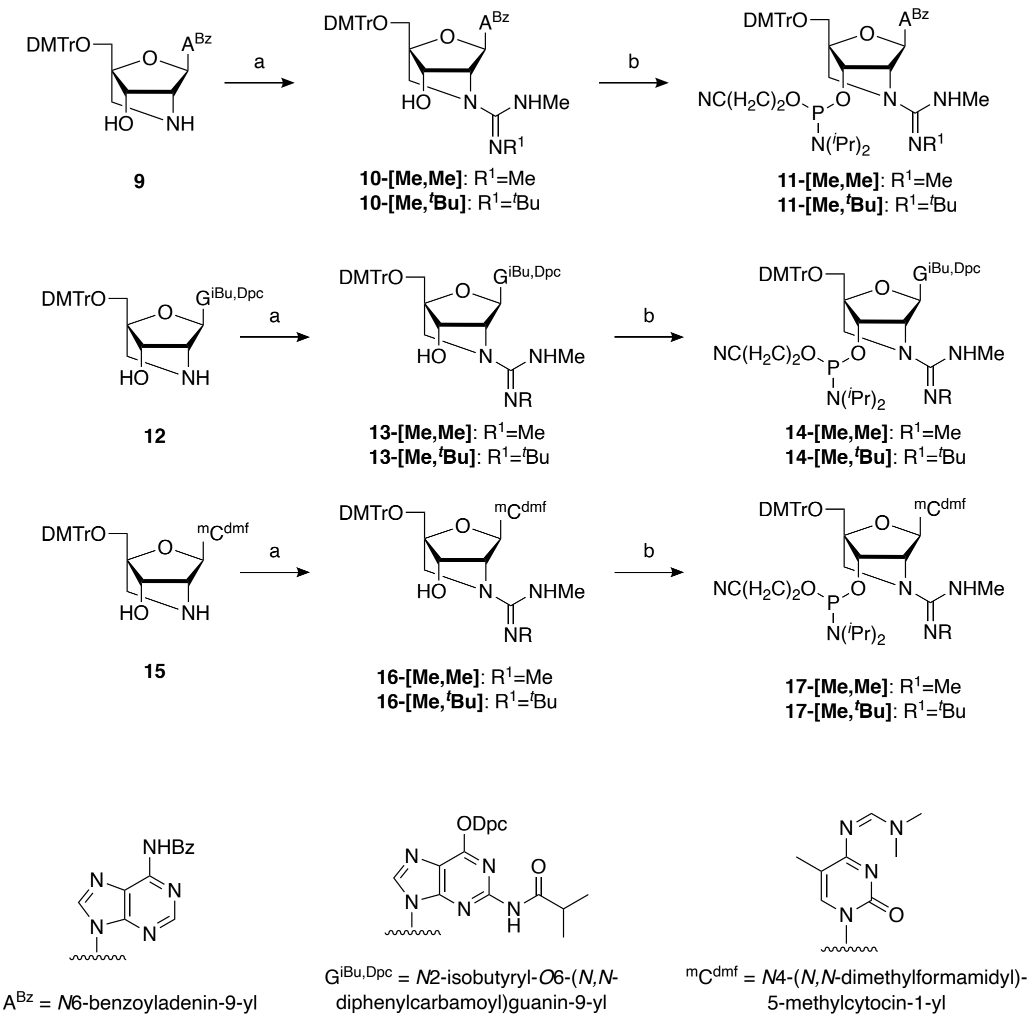

Scheme S1. Synthesis of GuNA[R,R]-A, -G, and -^{m}C phosphoramidites. Reagents and conditions: (a) *N,N',S*-trimethylisothiourea (**7**), AgOTf, DIPEA, DMA, 90 °C, 55% (for **10-[Me,Me]**), 23% (for **13-[Me,Me]**), 68% (for **16-[Me,Me]**); *N-tert*-butyl-*N',S*-dimethylisothiourea (**8**), AgOTf, DIPEA, DMA, 90 °C, 44% (for **10-[Me,^{t}Bu]**), 38% (for **13-[Me,^{t}Bu]**), 64% (for **16-[Me,^{t}Bu]**); (b) 2-cyanoethyl-*N,N*-diisopropylchlorophosphoramidite, DIPEA, CH_2Cl_2, rt., 68% (for **11-[Me,Me]**), 69% (for **11-[Me,^{t}Bu]**), 81% (for **14-[Me,Me]**), 81% (for **14-[Me,^{t}Bu]**), 67% (for **17-[Me,Me]**), 56% (for **17-[Me,^{t}Bu]**).

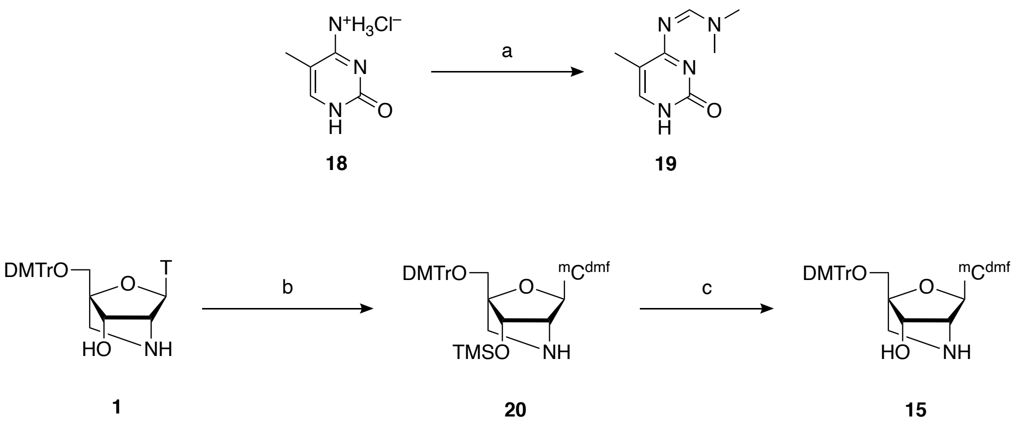

Scheme S2. Synthesis of a precursor **15** for GuNA[R,R]-^{m}C phosphoramidites. Reagents and conditions: (a) DMF-DMA, DMF, rt., quant.; (b) **19**, BSA, TMSOTf, DCE, 40 °C 67%; (c) TBAF, THF, rt., 96%.

Table S4. Synthesis result and characterization of oligonucleotides having GuNA[R,R]-A, -G, and -^{m}C analogs

Name	Oligonucleotide 5'–3'	A/G/^{m}C	Yield [%]	MALDI-TOF mass [M–H]$^-$	
				Calcd.	Found
**ODN13**	d(GCG TTA TTT GCT)	GuNA[Me,Me]	1.2	3738.5	3739.2
**ODN14**		GuNA[Me,^{t}Bu]	2.7	3780.6	3781.8
**ODN15**	d(GCG TTG TTT GCT)	GuNA[Me,Me]	0.3	3754.5	3755.1
**ODN16**		GuNA[Me,^{t}Bu]	< 0.1	3796.6	3797.7
**ODN17**	d(GCG TT^{m}C TTT GCT)	GuNA[Me,Me]	7.2	3728.5	3729.5
**ODN18**		GuNA[Me,^{t}Bu]	0.4	3770.6	3771.8

Table S5. Synthesis result and characterization of oligonucleotides for nuclease resistance study

Name	Oligonucleotide 5'–3'	T	Yield [%]	MALDI-TOF mass [M–H]$^-$	
				Calcd.	Found
**ODN8**	d(TTT TTT TTT T)	GuNA[Et]	18	3076.1	3076.7
**ODN9**		GuNA[^{i}Pr]	31	3090.1	3090.3
**ODN10**		GuNA[^{t}Bu]	12	3104.1	3104.7
**ODN11**		GuNA[Me,Me]	14	3076.1	3076.0
**ODN12**		GuNA[Me,^{t}Bu]	19	3118.2	3119.2

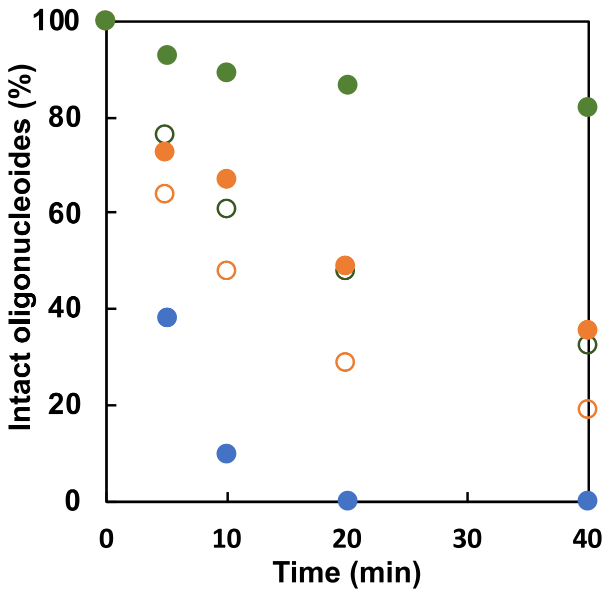

Figure S7. Stability of modified oligonucleotides against 3′-exonuclease. Conditions: 0.12 μg phosphodiesterase I from *Crotalus adamanteus* venom (svPDE), 10 mM $MgCl_2$, 50 mM Tris–HCl buffer (pH 8.0), 7.5 μM each oligonucleotide at 37 °C (total volume 100 μL). The sequence used in this study was 5′-d(TTTTTTTT<u>T</u>T)-3′. <u>T</u> = LNA (blue circle, **ODN34**), GuNA[Me] (orange open circle, **ODN35**), GuNA[*t*Bu] (green open circle, **ODN10**), GuNA[Me,Me] (orange closed circle, **ODN11**), or GuNA[Me,^{t}Bu] (green closed circle, **ODN12**).

2. Procedures for phosphoramidite synthesis

General

All moisture-sensitive reactions were carried out in well-dried glassware under N_2 or Ar atmosphere. Dehydrated acetonitrile, 1,2-dichloroethane (DCE), dichloromethane, *N,N*-dimethylacetamide (DMA), *N,N*-dimethylformamide (DMF), tetrahydrofuran (THF) were used as purchased. ^{1}H, ^{13}C and ^{31}P NMR spectra were recorded on JEOL JNM-AL300, JNM-ECS400, and JNM-ECA500 spectrometers. Chemical shift values are expressed in δ values (ppm) relative to tetramethylsilane (δ = 0.00 ppm) as internal standard, $CHCl_3$ (δ = 7.26 ppm) or DMSO (δ = 2.49 ppm) for ^{1}H NMR, $CDCl_3$ (δ = 77.0 ppm) or DMSO (δ = 39.5 ppm) for ^{13}C NMR, and 5% H_3PO_4 (δ = 0.00 ppm) for ^{31}P NMR. MALDI-TOF mass spectrometer (SpiralTOF JMS-S3000) was used to measure the mass spectra of all compounds. For manual chromatography, silica gels PSQ-60B (60 µm) and PSQ-100B (110 µM) (Fuji Silysia Chemical) were used. For automated flash chromatography, Hi-Flash™ column silica gel 40 µm (Yamazen) and Purif-Pack®-EX NH-50 µm (Shoko Science) were used at Smart Flash EPCLC W-Prep 2XY (Yamazen). The progress of reaction was monitored by analytical thin layer chromatography on glass plates (Merck, TLC Silica gel 60 F254), and the products were visualized by UV light.

Phosphoramidite synthesis

1-[5-O-(4,4'-Dimethoxytrityl)-2-(N'-acetyl-N-ethylguanydinyl)-4-C-methylene-β-D-ribopentofuranosyl]thymine (**2-[Et]**)

A solution of compound **1** (171 mg, 0.299 mmol) (see *Org. Biomol. Chem.* **2020**, *18*, 9461–9472) and **4** (55 mg, 0.34 mmol) in anhydrous THF (3 ml) was placed in an ice-bath. *N,N*-Diisopropylethylamine (78 µl, 0.45 mmol) and silver triflate (116 mg, 0.452 mmol) were added and the mixture was stirred at room temperature for 10 h. After completion of the reaction, mixture was diluted with ethyl acetate, and sat. aq. NaCl was added. After filtration, the product was extracted with ethyl acetate, washed with water and brine, dried using Na_2SO_4, and concentrated. The product was purified by column chromatography to afford **2-[Et]** (183 mg, 89%) as a white amorphous solid. ^{1}H NMR (DMSO-d_6, 80 ºC) δ: 1.10 (3H, t, J = 7.2 Hz), 1.59 (3H, s), 1.86 (3H, s), 3.10–3.49 (6H, m), 3.75 (6H, s), 4.16 (1H, s), 4.54 (1H, s), 5.52 (1H, s), 6.01 (1H, br), 6.90 (4H, d, J = 8.6 Hz), 7.15–7.33 (7H, m), 7.22–7.48 (3H, m), 7.67 (1H, br), 11.15 (1H, br); ^{13}C NMR (DMSO-d_6, 80 ºC) δ: 11.65, 14.50, 26.07, 37.00, 52.90, 54.78, 59.45, 63.34, 68.74, 85.62, 85.80, 87.35, 108.40, 113.01, 126.44, 127.45, 129.39, 129.43, 134.00, 134.93, 135.07, 144.18, 149.62, 158.02, 159.70, 163.22, 174.76; HRMS (MALDI): Calcd. for C37H42N5O8 $[M + H]^+$ 684.3028, Found 684.3037.

1-[5-O-(4,4'-Dimethoxytrityl)-2-(N'-acetyl-N-isopropylguanydinyl)-4-C-methylene-β-D-ribopentofuranosyl]-thymine (**2-[^{i}Pr]**)

A solution of compound **1** (171 mg, 0.299 mmol) and **5** (59 mg, 0.34 mmol) in anhydrous THF (3 ml) was

placed in an ice-bath. *N,N*-Diisopropylethylamine (78 µl, 0.45 mmol) and silver triflate (116 mg, 0.452 mmol) were added and the mixture was stirred at room temperature for 4 h. After completion of the reaction, mixture was diluted with ethyl acetate and sat. aq. NaCl was added. After filtration, the product was extracted with ethyl acetate, washed with water and brine, dried using Na_2SO_4, and concentrated. The product was purified by column chromatography to afford **2-[^{i}Pr]** (202 mg, 96%) as a white amorphous solid. ^{1}H NMR (DMSO-d_6, 80 ºC) δ: 1.12 (3H, d, *J* = 3.8 Hz), 1.14 (3H, *J* = 3.8 Hz), 1.59 (3H, s), 1.86 (3H, s), 3.09 (1H, s), 3.35 (1H, d, *J* = 10.3 Hz), 3.36 (1H, d, *J* = 11.0 Hz), 3.46 (1H, d, *J* = 10.3 Hz), 3.47 (1H, d, *J* = 10.7), 3.76 (6H, s), 3.91 (1H, br), 4.16 (1H, s), 4.49 (1H, s), 5.51 (1H, s), 6.06 (1H, br), 6.90 (4H, d, *J* = 8.6 Hz), 7.25–7.35 (7H, m), 7.42–7.48 (3H, m), 11.18 (1H, br); ^{13}C NMR (DMSO-d_6, 80 ºC) δ: 11.66, 22.23, 22.42, 26.03, 43.93, 53.11, 54.78, 59.42, 63.33, 68.71, 85.64, 87.41, 108.46, 113.01, 126.45, 127.45, 129.39, 129.43, 133.90, 134.93, 135.06, 144.19, 149.73, 158.03, 158.91, 163.21, 174.57; HRMS (MALDI): Calcd. for C38H44N5O8 $[M + H]^+$ 698.3184, Found 698.3188.

1-[5-O-(4,4'-Dimethoxytrityl)-2-(N'-acetyl-N-tert-butylguanydinyl)-4-C-methylene-β-D-ribopentofuranosyl]-thymine (**2-[^{t}Bu]**)

A solution of compound **1** (171 mg, 0.299 mmol) and **6** (66 mg, 0.35 mmol) in anhydrous THF (3 ml) was placed in an ice-bath. *N,N*-Diisopropylethylamine (78 µl, 0.45 mmol) and silver triflate (116 mg, 0.452 mmol) were added and the mixture was stirred at room temperature for 4 h. After completion of the reaction, mixture was diluted with ethyl acetate and sat. aq. NaCl was added. After filtration, the product was extracted with ethyl acetate, washed with water and brine, dried using Na_2SO_4, and concentrated. The product was purified by column chromatography to afford **2-[^{t}Bu]** (185 mg, 87%) as a white amorphous solid. ^{1}H NMR ($CDCl_3$) *δ* : 1.36 (9H, s), 1.66 (3H, s), 2.02 (3H, s), 3.27 (1H, d, *J* = 10.3 Hz), 3.44 (1H, d, *J* = 11.0 Hz), 3.51 (1H, d, *J* = 11.0 Hz), 3.57 (1H, d, *J* = 10.3 Hz), 3.76 (3H, s), 3.76 (3H, s), 4.23 (1H, s), 4.36 (1H, s), 5.49 (1H, s), 5.79 (1H, br), 6.31 (1H, br), 6.81 (4H, d, *J* = 7.9 Hz), 7.17–7.34 (7H, m), 7.43 (2H, d, *J* = 7.2 Hz), 7.53 (1H, d, *J* = 0.7 Hz), 9.78 (1H, br); ^{13}C NMR ($CDCl_3$) δ: 12.51, 26.35, 29.18, 53.43, 53.66, 55.12, 59.24, 63.62, 70.19, 85.40, 86.53, 88.73, 110.63, 113.17, 126.95, 127.90, 128.04, 129.98, 130.05, 134.14, 135.18, 135.44, 144.28, 150.25, 158.51, 159.16, 164.13, 175.66; HRMS (MALDI): Calcd. for C39H46N5O8 $[M + H]^+$ 712.3341, Found 712.3346.

1-[5-O-(4,4'-Dimethoxytrityl)-2-(N,N'-dimethylguanydinyl)-4-C-methylene-β-D-ribopentofuranosyl]-thymine (**2-[Me,Me]**)

A solution of compound **1** (173 mg, 0.303 mmol) and *N,N',S*-trimethylisothiourea (42 mg, 0.36 mmol) in anhydrous *N,N*-dimethylacetamide (3 ml) was placed in an ice-bath. *N,N*-Diisopropylethylamine (79 µl, 0.45 mmol) and silver triflate (122 mg, 0.475 mmol) were added and the mixture was stirred at 90 ºC for 1 h. Then, *N,N',S*-trimethylisothiourea (10 mg, 0.085 mmol), *N,N*-diisopropylethylamine (21 µl, 0.12 mmol) and silver

triflate (31 mg, 0.12 mmol) were added again and the mixture was stirred at 90 ºC for 2 h. After completion of the reaction, mixture was diluted with ethyl acetate and sat. aq. NaCl was added. After filtration, the product was extracted with ethyl acetate, washed with water and brine, dried using Na_2SO_4, and concentrated. The product was purified by column chromatography using amino silica gel to afford **2-[Me,Me]** (161.1 mg, 83%) as a yellow amorphous solid. ^{1}H NMR (DMSO-d_6) δ : 1.60 (3H, s), 2.81 (6H, s), 3.26 (1H, d, J = 10.5 Hz), 3.29 (1H, d, J = 11.5 Hz), 3.36 (1H, d, J = 11.0 Hz), 3.49 (1H, d, J = 10.1 Hz), 3.73 (6H, s), 3.95 (1H, s), 4.75 (1H, s), 5.59 (1H, s), 6.91 (4H, d, J = 8.6 Hz), 7.22–7.46 (10H, m); ^{13}C NMR (DMSO-d_6) δ: 12.54, 30.30, 55.09, 59.29, 64.55, 69.00, 85.83, 85.92, 87.34, 108.90, 113.31, 126.91, 127.65, 128.00, 129.75, 129.83, 133.78, 134.96, 135.19, 144.59, 158.23; HRMS (MALDI): Calcd. for C35H40N5O7 $[M + H]^+$ 642.2922, Found 642.2920.

1-[5-O-(4,4'-Dimethoxytrityl)-2-(N-tert-butyl-N'-methylguanydi-nyl)-4-C-methylene-β-D-ribopentofuranosyl]-thymine (**2-[Me,^{t}Bu]**)

A solution of compound **1** (3.0 g, 5.3 mmol) and *N-tert*-butyl-*N',S*-dimethylisothiourea (1.1 g, 7.1 mmol) in anhydrous *N,N*-dimethylacetamide (53 ml) was placed in an ice-bath. *N,N*-Diisopropylethylamine (0.98 ml, 5.6 mmol) and silver triflate (1.5 g, 5.6 mmol) were added and the mixture was stirred at 90 ºC for 2.5 h. Then, *N-tert*-butyl-*N',S*-dimethylisothiourea (1.1 g, 6.6 mmol), *N,N*-diisopropylethylamine (0.98 ml, 5.6 mmol) and silver triflate (1.4 g, 5.4 mmol) were added again and the mixture was stirred at 90 ºC for 2 h. After completion of the reaction, mixture was filtrated and concentrated. Then, the product was diluted with ethyl acetate, washed with sat. aq. $NaHCO_3$, sat. aq. $Na_2S_2O_3$ and brine, dried using Na_2SO_4, and concentrated. The product was purified by column chromatography using amino silica gel to afford **2-[Me,^{t}Bu]** (3.58 g, 98%) as an orange amorphous solid. ^{1}H NMR ($CDCl_3$) δ : 1.35 (9H, s), 1.66 (3H, s), 2.78 (3H, s), 3.20 (1H, d, J = 9.6 Hz), 3.30 (1H, d, J = 9.2 Hz), 3.43 (2H, s), 3.78 (3H, s), 3.79 (3H, s), 4.13 (1H, s), 4.57 (1H, s), 5.63 (1H, s), 6.82 (4H, dd, J = 2.1, 9.0 Hz), 7.18–7.37 (7H, m), 7.42–7.49 (2H, m), 7.56 (1H, s); ^{13}C NMR ($CDCl_3$) δ: 13.44, 30.23, 54.13, 55.22, 59.68, 64.55, 69.10, 86.48, 87.41, 88.78, 109.49, 113.22, 127.00, 127.94, 128.29, 130.12, 133.91, 135.46, 135.56, 144.40, 158.52, 158.55; HRMS (MALDI): Calcd. for C38H46N5O7 $[M + H]^+$ 684.33918, Found 684.33913.

1-[3-O-{2-Cyanoethoxy(diisopropylamino)phospino}-5-O-(4,4'-dimethoxytrityl)-2-(N'-acetyl-N-ethylguanydinyl)-4-C-methylene-β-D-ribopentofuranosyl]thymine (**3-[Et]**)

N,N-Diisopropylethylamine (190 µL, 1.1 mmol) and 2-cyanoethyl-*N,N*-diisopropyl-chlorophosphoramidite (120 µL, 0.54 mmol) were added to a solution of compound **2-[Et]** (183 mg, 0.27 mmol) in dichloromethane (3 mL), and the mixture was stirred at room temperature for 5 h. After completion of the reaction, sat. aq. $NaHCO_3$ was added and the product was extracted with chloroform. The organic phase was washed with water and brine, dried using Na_2SO_4, and concentrated. The product was purified by column chromatography to afford **3-[Et]**

(198 mg, 83%) as a white amorphous solid. ^{1}H NMR (CDCl$_3$) δ: 0.97 (18/5H, d, *J* = 6.9 Hz), 1.04 (12/5H, d, *J* = 6.9 Hz), 1.10 (18/5H, d, *J* = 6.9 Hz), 1.13 (12/5H, d, *J* = 6.9 Hz), 1.22 (6/5H, t, *J* = 7.3 Hz), 1.25 (9/5H, t, *J* = 7.3 Hz), 1.58 (3H, s), 2.02 (6/5H, s), 2.03 (9/5H, s), 2.39 (4/5H, t, *J* = 6.0 Hz), 2.50–2.64 (6/5H, m), 3.24 (2/5H, d, *J* = 10.1 Hz), 3.27 (2/5H, d, *J* = 10.1 Hz), 3.35–3.84 (46/5H, m), 3.79 (9/5H, s), 3.79 (9/5H, s), 3.80 (6/5H, s), 3.80 (6/5H, s), 4.38 (3/5H, d, *J* = 6.4 Hz), 4.47 (2/5H, d, *J* = 8.7 Hz), 4.74 (3/5H, s), 4.76 (2/5H, s), 5.67 (1H, s), 6.80–6.88 (4H, m), 7.22–7.37 (7H, m), 7.40–7.47 (2H, m), 7.73 (3/5H, d, *J* = 0.9 Hz), 7.74 (2/5H, d, *J* = 1.4 Hz), 8.24–9.20 (1H, br); ^{31}P NMR (CDCl$_3$) δ: 148.49, 148.56. HRMS (MALDI): Calcd. for C46H58N7O9NaP [M + Na]$^+$ 906.3926, Found 906.3910.

1-[3-O-{2-Cyanoethoxy(diisopropylamino)phospino}-5-O-(4,4'-dimethoxytrityl)-2-(N'-acetyl-N-isopropylguanydinyl)-4-C-methylene-β-D-ribopentofuranosyl]thymine (**3-[^{i}Pr]**)

N,N-Diisopropylethylamine (200 µL, 1.2 mmol) and 2-cyanoethyl-*N,N*-diisopropyl-chlorophosphoramidite (130 µL, 0.58 mmol) were added to a solution of compound **2-[^{i}Pr]** (200 mg, 0.29 mmol) in dichloromethane (3 mL), and the mixture was stirred at room temperature for 5 h. After completion of the reaction, sat. aq. NaHCO$_3$ was added and the product was extracted with chloroform. The organic phase was washed with water and brine, dried using Na$_2$SO$_4$, and concentrated. The product was purified by column chromatography to afford **3-[^{i}Pr]** (185 mg, 71%) as a white amorphous solid. ^{1}H NMR (CDCl$_3$) δ: 0.97 (3H, d, *J* = 6.9 Hz), 1.06 (3H, d, *J* = 6.9 Hz), 1.11 (3H, d, *J* = 6.9 Hz), 1.13 (3H, d, *J* = 6.9 Hz), 1.17 (3/2H, d, *J* = 6.5 Hz), 1.21 (3/2H, d, *J* = 6.5 Hz), 1.24 (3/2H, d, *J* = 6.2 Hz), 1.26 (3/2H, d, *J* = 6.2 Hz), 1.59 (3H, s), 2.03 (3H, s), 2.38 (1H, t, *J* = 6.2 Hz), 2.57 (1H, dt, *J* = 2.1, 5.9 Hz), 3.21 (1/2H, d, *J* = 10.7 Hz), 3.21 (1/2H, d, *J* = 10.7 Hz), 3.36 (1H, d, *J* = 10.7 Hz), 3.42–3.91 (6H, m), 3.80 (3H, s), 3.80 (3/2H, s), 3.80 (3/2H, s), 3.96–4.12 (1H, m), 4.37 (1/2H, d, *J* = 6.5 Hz), 4.47 (1/2H, d, *J* = 8.9 Hz), 4.65 (1/2H, s), 4.68 (1/2H, s), 5.64 (1H, s), 6.81–6.87 (4H, m), 7.21–7.35 (7H, m), 7.40–7.46 (2H, m), 7.71 (1/2H, d, *J* = 1.0 Hz), 7.73 (1/2H, d, *J* = 1.0 Hz), 8.59 (1H, br); ^{31}P NMR (CDCl$_3$) δ: 148.45, 148.88; HRMS (MALDI): Calcd. for C47H60N7O9NaP [M + Na]$^+$ 920.4082, Found 920.4090.

1-[3-O-{2-Cyanoethoxy(diisopropylamino)phospino}-5-O-(4,4'-dimethoxytrityl)-2-(N'-acetyl-N-tert-butyl-guanydinyl)-4-C-methylene-β-D-ribopentofuranosyl]thymine (**3-[^{t}Bu]**)

N,N-Diisopropylethylamine (180 µL, 1.0 mmol) and 2-cyanoethyl-*N,N*-diisopropyl-chlorophosphoramidite (120 µL, 0.52 mmol) were added to the solution of compound **2-[^{t}Bu]** (185 mg, 0.26 mmol) in dichloromethane (3 mL), and the mixture was stirred at room temperature for 5 h. After completion of the reaction, sat. aq. NaHCO$_3$ was added and the product was extracted with chloroform. The organic phase was washed with water and brine, dried using Na$_2$SO$_4$, and concentrated. The product was purified by column chromatography to afford **3-[^{t}Bu]** (115 mg, 48%) as a white amorphous solid. ^{1}H NMR (CDCl$_3$) δ: 0.97 (18/5H, d, *J* = 6.9 Hz), 1.09 (12/5H, d, *J* = 6.3 Hz), 1.12 (18/5H, d, *J* = 6.3 Hz), 1.15 (12/5H, d, *J* = 6.9 Hz), 1.41 (18/5H, s), 1.43 (27/5H, s), 1.64 (3H, s), 2.02 (3H, s), 2.34–2.45 (4/5H, m), 2.53–2.63 (6/5H, m), 3.15 (2/5H, d, *J* = 11.5 Hz), 3.17 (3/5H,

d, J = 10.9 Hz), 3.30 (2/5H, d, J = 10.9 Hz), 3.33 (3/5H, d, J = 10.9 Hz), 3.45–3.58 (4H, m), 3.64 (3/5H, d, J = 10.9 Hz), 3.65 (2/5H, d, J = 10.9 Hz), 3.77–3.83 (2/5H, m), 3.80 (18/5H, s), 3.80 (6/5H, s), 3.81 (6/5H, s), 3.87–3.95 (3/5H, m), 4.31 (3/5H, d, J = 6.3 Hz), 4.40 (2/5H, s), 4.46 (2/5H, d, J = 8.6 Hz), 4.55 (3/5H, s), 5.52 (2/5H, s), 5.55 (3/5H, s), 5.64 (2/5H, s), 5.70 (3/5H, s), 6.81–6.86 (4H, m), 7.22–7.32 (7H, m), 7.40–7.44 (2H, m), 7.66 (3/5H, d, J = 1.2 Hz), 7.69 (2/5H, d, J = 1.2 Hz), 8.39 (1H, br); ^{31}P NMR ($CDCl_3$) δ: 147.79, 149.30; HRMS (MALDI): Calcd. for C48H62N7O9NaP $[M + Na]^+$ 934.4239, Found 934.4239.

1-[3-O-{2-Cyanoethoxy(diisopropylamino)phospino}-5-O-(4,4'-dimethoxytrityl)-2-(N,N'-dimethylguanydinyl)-4-C-methylene-β-D-ribopentofuranosyl]thymine (**3-[Me,Me]**)

N,N-Diisopropylethylamine (0.54 mL, 3.1 mmol) and 2-cyanoethyl-*N,N*-diisopropyl-chlorophosphoramidite (0.42 mL, 1.9 mmol) were added to the solution of compound **2-[Me,Me]** (0.99 g, 1.6 mmol) in dichloromethane (16 mL), and the mixture was stirred at room temperature for 2.5 h. Then, 2-cyanoethyl-*N,N*-diisopropylchlorophosphoramidite (70 μL, 0.3 mmol) were added again and the mixture was stirred at room temperature for 1 h. After completion of the reaction, sat. aq. $NaHCO_3$ was added and the product was extracted with chloroform. The organic phase was washed with water and brine, dried using Na_2SO_4, and concentrated. The product was purified by column chromatography using amino silica gel to afford **3-[Me,Me]** (1.27 g, 98%) as a yellow amorphous solid. ^{1}H NMR ($CDCl_3$) δ: 0.93 (12/5H, d, J = 6.9 Hz), 1.00 (18/5H, d, J = 6.4 Hz), 1.11 (12/5H, d, J = 6.4 Hz), 1.12 (18/5H, d, J = 6.9 Hz), 1.82 (9/5H, s), 1.83 (6/5H, s), 2.27–2.41 (6/5H, m), 2.53 (4/5H, t, J = 6.4 Hz), 3.10 (6H, s), 3.40–3.84 (8H, m), 3.78 (12/5H, s), 3.79 (18/5H, s), 4.20 (2/5H, d, J = 6.4 Hz), 4.27 (3/5H, s), 4.30 (2/5H, d, J = 9.6 Hz), 4.35 (2/5H, s), 5.62 (1H, s), 6.82–6.87 (4H, m), 7.20–7.50 (10H, m), 7.40–7.47 (2H, m), 7.73 (3/5H, d, J = 0.9 Hz), 7.74 (2/5H, d, J = 1.4 Hz), 8.24–9.20 (1H, br); ^{31}P NMR ($CDCl_3$) δ: 148.63, 150.94; HRMS (MALDI): Calcd. for C44H57N7O8P $[M + H]^+$ 842.4001, Found 842.3985.

1-[3-O-{2-Cyanoethoxy(diisopropylamino)phospino}-5-O-(4,4'-dimethoxytrityl)-2-(N-tert-butyl-N'-methylguanydinyl)-4-C-methylene-β-D-ribopentofuranosyl]thymine (**3-[Me,^{t}Bu]**)

N,N-Diisopropylethylamine (58 μL, 0.33mmol) and 2-cyanoethyl-*N,N*-diisopropyl-chlorophosphoramidite (74 μL, 0.33mmol) were added to the solution of compound **2-[Me,^{t}Bu]** (0.19 g, 0.28 mmol) in dichloromethane (2.8 mL), and the mixture was stirred at room temperature for 3 h. Then, *N,N*-diisopropylethylamine (14 μL, 0.080 mmol) and 2-cyanoethyl-*N,N*-diisopropylchlorophosphoramidite (18 μL, 0.081 mmol) were added again and the mixture was stirred at room temperature for 1 h. After completion of the reaction, sat. aq. $NaHCO_3$ was added and the product was extracted with chloroform. The organic phase was washed with water and brine, dried using Na_2SO_4, and concentrated. The product was purified by column chromatography using amino silica gel to afford **3-[Me,^{t}Bu]** (209.4 mg, 86%) as a yellow amorphous solid. ^{1}H NMR ($CDCl_3$) δ: 0.95 (12/5H, d, J = 6.9 Hz), 1.05 (18/5H, d, J = 6.9 Hz), 1.11 (12/5H, d, J = 6.9 Hz), 1.14 (18/5H, d, J = 6.9 Hz), 1.33 (27/5H, s), 1.34 (18/5H, s), 1.57 (6/5H, s), 1.59 (9/5H, s), 2.92 (9/5H, s), 2.93 (6/5H, s), 3.31–3.90 (8H, m), 3.79 (12/5H,

s), 3.80 (18/5H, s), 4.30 (2/5H, d, *J* = 7.3 Hz), 4.36 (2/5H, d, *J* = 9.6 Hz), 4.41 (3/5H, s), 4.52 (2/5H, s), 5.65 (3/5H, s), 5.67 (2/5H, s), 6.81–6.86 (4H, m), 7.21–7.36 (7H, m), 7.42–7.48 (2H, m), 7.68 (1H, s); ^{31}P NMR ($CDCl_3$) δ: 148.83, 149.65; HRMS (MALDI): Calcd. for C47H63N7O8P $[M + H]^+$ 884.44702, Found 884.44537.

N'-Acetyl-N-ethyl-S-methylisothiourea (**4**)

Triethylamine (2.8 mL, 20 mmol) and acetic anhydride (1.2 mL, 13 mmol) were added to a solution of *N*-ethyl-*S*-methylisothiourea iodate (2.46 g, 10.0 mmol) in dichloromethane (12 mL), and the mixture was stirred at room temperature for 19 h. After completion of the reaction, sat. aq. $NaHCO_3$ was added and the product was extracted with chloroform. The organic phase was washed with brine, dried using Na_2SO_4, and concentrated. The product was purified by column chromatography to afford **4** (1.34 g, 84%) as a yellow oil. ^{1}H NMR ($CDCl_3$) δ: 1.21 (3H, t, *J* = 7.2 Hz), 2.10 (3H, s), 2.42 (3H, s), 3.30 (2H, q, *J* = 7.2 Hz), 10.85 (1H, s); ^{13}C NMR ($CDCl_3$) δ: 13.48, 14.36, 28.09, 38.42, 172.64, 183.70; HRMS (MALDI): Calcd. for C6H13N2OS $[M + H]^+$ 161.0743, Found 161.0742.

N'-Acetyl-N-isopropyl-S-methylisothiourea (**5**)

Triethylamine (2.1 mL, 15 mmol) and acetic anhydride (1.0 mL, 11 mmol) were added to a solution of *N*-isopropyl-*S*-methylisothiourea iodate (2.60 g, 10.0 mmol) in dichloromethane (13 mL), and the mixture was stirred at room temperature for 18.5 h. After completion of the reaction, sat. aq. $NaHCO_3$ was added and the product was extracted with chloroform. The organic phase was washed with brine, dried using Na_2SO_4, and concentrated. The product was purified by column chromatography to afford **5** (1.62 g, 93%) as a yellow oil. ^{1}H NMR ($CDCl_3$) δ: 1.22 (6H, d, *J* = 6.2 Hz), 2.10 (3H, s), 2.42, (3H, s), 3.76–3.86 (1H, m), 10.88 (1H, s); ^{13}C NMR ($CDCl_3$) δ: 13.55, 22.79, 28.14, 46.11, 171.32, 183.67; HRMS (MALDI): Calcd. for C7H15N2OS $[M + H]^+$ 175.0900, Found 175.0901.

N'-Acetyl-S-methyl-N-tert-butylisothiourea (**6**)

Triethylamine (2.1 mL, 15 mmol) and acetic anhydride (1.0 mL, 11 mmol) were added to a solution of *S*-methyl-*N*-*tert*-butylisothiourea iodate (2.74 g, 10.0 mmol) in dichloromethane (14 mL), and the mixture was stirred at room temperature for 23 h. After completion of the reaction, sat. aq. $NaHCO_3$ was added and the product was extracted with chloroform. The organic phase was washed with brine, dried using Na_2SO_4, and concentrated. The product was purified by column chromatography to afford **6** (1.82 g, 97%) as a yellow oil. ^{1}H NMR ($CDCl_3$) δ: 1.41 (9H, s), 2.09 (3H, s), 2.44 (3H, s), 11.22 (1H, s); ^{13}C NMR ($CDCl_3$) δ: 14.19, 28.12, 29.13, 53.01, 170.87, 183.11; HRMS (MALDI): Calcd. for C8H17N2OS $[M + H]^+$ 189.1056, Found 189.1053.

6N-Benzoyl-1-[5-O-(4,4'-dimethoxytrityl)-2-(N,N'-dimethylguanydinyl)-4-C-methylene-β-D-ribopentofurano-

syl]adenine (**10-[Me,Me]**)

A solution of compound **9** (0.72 g, 1.1 mmol) and *N,N',S*-trimethylisothiourea (**7**) (0.19 mg, 1.6 mmol) in anhydrous *N,N*-dimethylacetamide (10 ml) was placed in an ice-bath. *N,N*-diisopropylethylamine (0.28 mL, 1.6 mmol) and silver triflate (0.40 g, 1.6 mmol) were added and the mixture was stirred at 90 ºC for 1.5 h. After completion of the reaction, mixture was diluted with ethyl acetate and sat. aq. NaCl was added. After filtration, the product was extracted with ethyl acetate, washed with water and brine, dried using Na_2SO_4, and concentrated. The product was purified by column chromatography using amino silica gel to afford **10-[Me,Me]** (438.9 mg, 55%) as an orange amorphous solid/ IR (KBr): 3058, 3003, 2935, 2358, 1606, 1579, 1509, 1447, 1415, 1376, 1328, 1295, 1252, 1225, 1177, 1156, 1035 cm^{-1}; ^1H NMR (DMSO-d_6) δ: 2.86 (6H, s), 3.28 (1H, d, J = 10.9 Hz), 3.50 (1H, d, J = 9.7 Hz). 3.52 (1H, d, J = 10.9 Hz), 3.67 (1H, d, J = 9.7 Hz), 3.72 (6H, s), 4.41 (1H, s), 4.86 (1H, s), 6.22 (1H, s), 6.89 (4H, dd, J = 1.7, 8.6 Hz), 7.21–7.45 (12H, m), 7.97 (2H, d, J = 7.5 Hz), 8.18 (1H, s), 8.48 (1H, s). ^{13}C NMR (DMSO-d_6) δ: 30.93, 54.77, 55.11, 60.57, 64.18, 70.61, 84.98, 85.61, 87.45, 113.31, 125.75, 126.87, 127.72, 127.92, 128.00, 128.57, 129.77, 129.85, 135.19, 135.49, 144.74, 152.07, 158.20, 158.43, 167.49. HRMS (MALDI): Calcd. for C42H43N8O6 $[M + H]^+$ 755.33001, Found 755.32910.

6N-Benzoyl-1-[5-O-(4,4'-dimethoxytrityl)-2-(N-methyl-N'-tert-butylguanydinyl)-4-C-methylene-β-D-ribopento-furanosyl]adenine (**10-[Me,^{t}Bu]**)

A solution of compound **9** (0.69 g, 1.0 mmol) and *N-tert*-butyl-*N',S*-dimethylisothiourea (**8**) (0.21 g, 1.3 mmol) in anhydrous *N,N*-dimethylacetamide (10 ml) was placed in an ice-bath. *N,N*-diisopropylethylamine (0.26 mL, 1.5 mmol) and silver triflate (0.39 g, 1.5 mmol) were added and the mixture was stirred at 90 ºC for 4 h. After completion of the reaction, mixture was diluted with ethyl acetate and sat. aq. NaCl was added. After filtration, the product was extracted with ethyl acetate, washed with water and brine, dried using Na_2SO_4, and concentrated. The product was purified by column chromatography to afford **10-[Me,^{t}Bu]** (354.9 mg, 44%) as a yellow amorphous solid. IR (KBr): 3305, 3069, 2976, 1701, 1609, 1509, 1455, 1411, 1376, 1283, 1176, 1103, 1050, 1031 cm^{-1}; ^1H NMR ($CDCl_3$) δ: 1.49 (9H, s), 3.12 (3H, d, J = 4.8 Hz), 3.51 (1H, d, J = 11.0 Hz), 3.61 (1H, d, J = 11.0 Hz), 3.69 (2H, s), 3.78 (6H, s), 4.55 (1H, s), 5.07 (1H, s), 5.52 (1H, br), 6.16 (1H, s), 6.47 (1H, d, J = 5.2 Hz), 6.71 (1H, s), 6.84 (4H, d, J = 8.9 Hz), 7.18–7.36 (7H, m), 7.41–7.46 (2H, m), 7.48–7.56 (2H, m), 7.58–7.65 (1H, m), 8.00 (2H, d, J = 7.2 Hz), 8.28 (1H, s), 8.72 (1H, s), 9.23 (1H, s). ^{13}C NMR ($CDCl_3$) δ: 29.63, 31.88, 55.20, 55.35, 56.06, 59.53, 64.89, 70.15, 85.71, 86.65, 88.56, 113.28, 123.21, 127.07, 127.90, 127.99, 128.86, 129.96, 130.00, 132.95, 133.24, 135.09, 135.34, 139.93, 144.15, 149.56, 150.55, 152.50, 157.61, 158.59, 164.96. HRMS (MALDI): Calcd. for C45H49N8O6 $[M + H]^+$ 797.37696, Found 797.37412.

6N-Benzoyl-1-[3-O-{2-cyanoethoxy(diisopropylamino)phospino}-5-O-(4,4'-dimethoxytrityl)-2-(N,N'-dimethyl-guanydinyl)-4-C-methylene-β-D-ribopentofuranosyl]adenine (**11-[Me,Me]**)

N,N-Diisopropylethylamine (85 μL, 0.49 mmol) and 2-cyanoethyl-*N,N*-diisopropylchlorophosphoramidite

(0.11 μL, 0.49 mmol) were added to the solution of **10-[Me,Me]** (0.30 g, 0.40 mmol) in dichloromethane (4 mL), and the mixture was stirred at room temperature for 6 h. Then, *N,N*-diisopropylethylamine (85 μL, 0.49 mmol) and 2-cyanoethyl-*N,N*-diisopropylchlorophosphoramidite (0.11 mL, 0.49 mmol) were added again and the mixture was stirred at room temperature for 1.5 h. After completion of the reaction, sat. aq. $NaHCO_3$ was added and the product was extracted with chloroform. The organic phase was washed with water and brine, dried using Na_2SO_4, and concentrated. The product was purified by column chromatography to afford **11-[Me,Me]** (261.3 mg, 68%) as a white amorphous solid. ^{1}H NMR ($CDCl_3$) *δ*: 0.92 (6H, d, *J* = 6.4 Hz), 1.08 (6H, d, *J* = 6.9 Hz), 2.29–2.42 (7/5H, m), 2.51 (3/5H, br), 3.15 (21/5H, s), 3.18 (9/5H, s), 3.32–3.76 (7H, m), 3.78 (9/5H, s), 3.78 (21/5H, s), 3.87 (3/10H, d, *J* = 9.6 Hz), 3.94 (7/10H, d, *J* = 10.1 Hz), 4.76 (3/10H, d, *J* = 6.0 Hz), 4.80 (7/10H, d, *J* = 8.8 Hz), 5.04 (7/10H, s), 5.12 (3/10H, s), 6.30 (3/10H, s), 6.38 (7/10H, s), 6.80-6.86 (4H, m), 7.20–7.35 (7H, m), 7.40–7.45 (2H, m), 7.50–7.55 (2H, m), 7.58–7.64 (1H, m), 8.03 (2H, d, *J* = 7.8 Hz), 8.24 (3/10H, br), 8.33 (7/10H, br), 8.37 (3/10H, s), 8.43 (7/10H, s), 8.76 (7/10H, s), 8.77 (3/10H, s). ^{31}P NMR ($CDCl_3$) δ: 149.76, 151.47. HRMS (MALDI): Calcd. for C51H60N10O7P $[M + H]^+$ 955.43786, Found 955.44167.

6N-Benzoyl-1-[3-O-{2-cyanoethoxy(diisopropylamino)phospino}-5-O-(4,4'-dimethoxytrityl)-2-(N-methyl-N'-tert-butylguanydinyl)-4-C-methylene-β-D-ribopentofuranosyl]adenine (**11-[Me,^{t}Bu]**)

N,N-Diisopropylethylamine (49 μL, 0.28 mmol) and 2-cyanoethyl-*N,N*-diisopropylchlorophosphoramidite (62 μL, 0.28 mmol) were added to the solution of **10-[Me,^{t}Bu]** (0.16 g, 0.23 mmol) in dichloromethane (2.3 mL), and the mixture was stirred at room temperature for 3 h. Then, *N,N*-diisopropylethylamine (25 μL, 0.14 mmol) and 2-cyanoethyl-*N,N*-diisopropylchlorophosphoramidite (30 μL, 0.13 mmol) were added again and the mixture was stirred at room temperature for 1 h. After completion of the reaction, sat. aq. $NaHCO_3$ was added and the product was extracted with chloroform. The organic phase was washed with water and brine, dried using Na_2SO_4, and concentrated. The product was purified by column chromatography to afford **11-[Me,^{t}Bu]** (158.5 mg, 69%) as a white amorphous solid. ^{1}H NMR ($CDCl_3$) *δ*: 0.91 (9/5H, d, *J* = 6.4 Hz), 0.92 (21/5H, d, *J* = 6.9 Hz), 1.09 (6H, d, *J* = 6.9 Hz), 1.63 (63/10H, s), 1.64 (27/10H, s), 2.23-2.47 (2H, m), 3.16 (21/10H, s), 3.19 (9/10H, s), 3.45-3.78 (7H, m), 3.79 (9/5H, s), 3.80 (21/5H, s), 3.87 (7/10H, d, *J* = 10.1 Hz), 3.89 (3/10H, d, *J* = 8.2 Hz), 4.71 (7/10H, d, *J* = 9.2 Hz), 4.73 (3/10H, d, *J* = 8.2 Hz), 4.85 (7/10H, s), 4.96 (3/10H, s), 6.12 (3/10H, br), 6.17 (7/10H, br), 6.29 (3/10H, s), 6.37 (7/10H, s), 7.18–7.36 (7H, m), 7.43–7.47 (2H, m), 7.52–7.56 (2H, m), 7.61–7.65 (1H, m), 8.32 (3/10H, s), 8.39 (7/10H, s), 8.72 (7/10H, s), 8.74 (3/10H, s), 9.16 (1H, br) . ^{31}P NMR ($CDCl_3$) δ: 149.83, 151.48. HRMS (MALDI): Calcd. for C54H66N10O7P $[M + H]^+$ 997.48481, Found 997.48171.

2N-Isobutyryl-6O-diphenylcalbamoyl-1-[5-O-(4,4'-dimethoxytrityl)-2-(N,N'-dimethylguanydinyl)-4-C-methylene-β-D-ribopentofuranosyl]guanine (**13-[Me,Me]**)

A solution of compound **12** (0.86 g, 1.0 mmol) and *N,N',S*-trimethylisothiourea (0.17 g, 1.5 mmol) in anhydrous

N,N-dimethylacetamide (10 ml) was placed in an ice-bath. *N,N*-diisopropylethylamine (0.27 mL, 1.5 mmol) and silver triflate (0.39 g, 1.5 mmol) were added and the mixture was stirred at 90 ºC for 1.5 h. After completion of the reaction, mixture was diluted with ethyl acetate and sat. aq. NaCl was added. After filtration, the product was extracted with ethyl acetate, washed with water and brine, dried using Na_2SO_4, and concentrated. The product was purified by column chromatography to afford **13-[Me,Me]** (161.1 mg, 23%) as an orange amorphous solid. IR (KBr): 3297, 3067, 2972, 2937, 1746, 1595, 1508, 1493, 1446, 1412, 1382, 1335, 1282, 1252, 1177, 1105, 1030, 1003 cm^{-1}; ^1H NMR ($CDCl_3$) δ: 1.19 (3H, d, *J* = 6.2 Hz), 1.21 (3H, d, *J* = 6.5 Hz), 2.57 (1H, m), 3.03 (6H, s), 3.52 (2H, s), 3.63 (1H, d, *J* = 9.6 Hz), 3.77 (6H, s), 3.87 (1H, d, *J* = 9.6 Hz), 4.40 (1H, s), 4.54 (1H, br), 5.06 (1H, s), 6.00 (1H, s), 6.70 (1H, br), 6.84 (4H, d, *J* = 8.6 Hz), 7.19–7.45 (19H, m), 8.14 (1H, s), 8.39 (1H, s). ^{13}C NMR ($CDCl_3$) δ: 19.06, 19.27, 31.15, 36.52, 55.19, 59.63, 63.90, 70.93, 85.74, 86.59, 87.97, 113.27, 118.53, 121.36, 121.71, 127.03, 127.97, 128.01, 129.22, 129.98, 135.12, 135.28, 141.32, 141.44, 150.45, 151.82, 153.09, 155.97, 158.22, 158.60, 175.49. HRMS (MALDI): Calcd. for C52H54N9O8 $[M + H]^+$ 932.40899, Found 932.40832.

2N-Isobutyryl-6O-diphenylcalbamoyl-1-[5-O-(4,4'-dimethoxytrityl)-2-(N-methyl-N'-tert-butylguanydinyl)-4-C-methy-lene-β-D-ribopentofuranosyl]guanine (**13-[Me,^{t}Bu]**)

A solution of compound **12** (0.86 g, 1.0 mmol) and *N-tert*-butyl-*N',S*-dimethylisothiourea (0.25 g, 1.5 mmol) in anhydrous *N,N*-dimethylacetamide (10 ml) was placed in an ice-bath. *N,N*-diisopropylethylamine (0.26 mL, 1.5 mmol) and silver triflate (0.40 g, 1.6 mmol) were added and the mixture was stirred at 90 ºC for 2.5 h. After completion of the reaction, mixture was diluted with ethyl acetate and sat. aq. NaCl was added. After filtration, the product was extracted with ethyl acetate, washed with water and brine, dried using Na_2SO_4, and concentrated. The product was purified by column chromatography to afford **13-[Me,^{t}Bu]** (369.3 mg, 38%) as a white amorphous solid. IR (KBr): 3324, 3060, 2974, 2935, 2876, 2837, 2251, 1741, 1593, 1508, 1494, 1445, 1411, 1380, 1336, 1253, 1176, 1100, 1032, 1004 cm^{-1}; ^1H NMR ($CDCl_3$) δ: 1.13 (3H, d, *J* = 6.9 Hz), 1.17 (3H, d, *J* = 6.9 Hz), 1.35 (9H, s), 2.62 (1H, m), 3.10 (3H, d, *J* = 4.6 Hz), 3.46 (1H, d, *J* = 10.9 Hz), 3.55 (1H, d, *J* = 10.9 Hz), 3.63 (1H, d, *J* = 9.7 Hz), 3.65 (1H, d, *J* = 9.7 Hz), 3.75 (3H, s), 3.76 (3H, s), 4.67 (1H, d, *J* = 2.3 Hz), 4.96 (1H, d, *J* = 2.3 Hz), 5.07 (1H, s), 6.13 (1H, s), 6.15 (1H, s), 6.20 (1H, d, *J* = 5.2 Hz), 6.82 (4H, dd, *J* = 1.2, 9.2 Hz), 7.18–7,45 (19H, m), 8.21 (1H, s), 8.57 (1H, s). ^{13}C NMR ($CDCl_3$) δ: 18.97, 19.25, 29.43, 32.45, 36.37, 55.10, 55.19, 55.41, 59.75, 64.94, 71.20, 85.96, 86.60, 87.90, 113.27, 118.59, 121.62, 121.77, 127.01, 127.97, 128.00, 129.20, 130.00, 135.15, 135.29, 141.59, 142.27, 144.25, 150.61, 151.83, 153.36, 156.01, 157.65, 158.59, 175.51. HRMS (MALDI): Calcd. for C55H60N9O8 $[M + H]^+$ 974.45594, Found 974.45233.

2N-Isobutyryl-6O-diphenylcalbamoyl-1-[3-O-{2-cyanoethoxy(diisopropylamino)phospino}-5-O-(4,4'-dimetho-xytrityl)-2-(N,N'-dimethylguanydinyl)-4-C-methylene-β-D-ribopentofuranosyl]guanine (**14-[Me,Me]**)

N,N-Diisopropylethylamine (32 μL, 0.18 mmol) and 2-cyanoethyl-*N,N*-diisopropylchlorophosphoramidite (40

μL, 0.18 mmol) were added to the solution of **13-[Me,Me]** (0.14 g, 0.15 mmol) in dichloromethane (1.5 mL), and the mixture was stirred at room temperature for 2 h. Then, *N,N*-diisopropylethylamine (32 μL, 0.18 mmol) and 2-cyanoethyl-*N,N*-diisopropylchlorophosphoramidite (40 μL, 0.18 mmol) were added again and the mixture was stirred at room temperature for 1 h. After completion of the reaction, sat. aq. $NaHCO_3$ was added and the product was extracted with chloroform. The organic phase was washed with water and brine, dried using Na_2SO_4, and concentrated. The product was purified by column chromatography to afford **14-[Me,Me]** (137.8 mg, 81%) as a white amorphous solid. ^{1}H NMR ($CDCl_3$) δ: 0.85 (18/5H, d, J = 6.9 Hz), 0.89 (12/5H, d, J = 6.9 Hz), 1.06 (18/5H, d, J = 6.9 Hz), 1.07 (12/5H, d, J = 6.9 Hz), 1.23 (9/5H, d, J = 6.9 Hz), 1.24 (6/5H, d, J = 7.3 Hz), 1.24 (9/5H, d, J = 6.9 Hz), 1.26 (6/5H, d, J = 6.9 Hz), 2.31 (6/5H, t, J = 6.0 Hz), 2.36–2.65 (9/5H, m), 3.19 (3H, s), 3.29-3.83 (7H, m), 3.78 (6/5H, s), 3.78 (6/5H, s), 3.79 (9/5H, s), 3.79 (9/5H, s), 3.90 (2/5H, d, J = 10.1 Hz), 4.00 (3/5H, d, J = 10.1 Hz), 4.39 (2/5H, d, J = 5.5 Hz), 4.43 (3/5H, d, J = 9.2 Hz), 5.19 (3/5H, s), 5.29 (2/5H, s), 6.02 (1H, s), 6.83–6.87 (4H, m), 7.18–7.45 (19H, m), 7.88 (1H, br), 8.21 (2/5H, s), 8.25 (3/5H, s), 8.34 (3/5H, s), 8.37 (2/5H, s). ^{31}P NMR ($CDCl_3$) δ: 150.19, 150.59. HRMS (MALDI): Calcd. for C61H71N11O9P $[M + H]^+$ 1132.51684, Found 1132.51757.

2N-Isobutyryl-6O-diphenylcalbamoyl-1-[3-O-{2-cyanoethoxy(diisopropylamino)phospino}-5-O-(4,4'-dimetho-xytrityl)-2-(N-methyl-N'-tert-butylguanydinyl)-4-C-methylene-β-D-ribopentofuranosyl]guanine (**14-[Me,*t*Bu]**)

N,N-Diisopropylethylamine (35 μL, 0.20 mmol) and 2-cyanoethyl-*N,N*-diisopropylchlorophosphoramidite (42 μL, 0.19 mmol) were added to the solution of **13-[Me,*t*Bu]** (0.15 g, 0.16 mmol) in dichloromethane (1.6 mL), and the mixture was stirred at room temperature for 3 h. After completion of the reaction, sat. aq. $NaHCO_3$ was added and the product was extracted with chloroform. The organic phase was washed with water and brine, dried using Na_2SO_4, and concentrated. The product was purified by column chromatography to afford **14-[Me,*t*Bu]** (149.5 mg, 81%) as a white amorphous solid. ^{1}H NMR ($CDCl_3$) δ: 0.87 (4H, d, J = 6.9 Hz), 0.87 (2H, d, J = 6.4 Hz), 1.05 (4H, d, J = 6.9 Hz), 1.07 (2H, d, J = 7.8 Hz), 1.19–1.25 (6H, m), 1.50 (6H, s), 1.51 (3H, s), 2.25–2.70 (3H, m), 3.22 (2H, s), 3.25 (1H, s), 3.30–3.99 (8H, m), 3.78 (2H, s), 3.78 (4H, s), 4.49 (1H, d, J = 8.2 Hz), 5.17 (2/3H, s), 5.28 (1/3H, s), 6.17 (1/3H, s), 6.29 (2/3H, s), 6.82–6.86 (4H, m), 7.17–7.45 (19H, m), 8.24 (1/3H, s), 8.31 (2/3H, s), 8.31 (1/3H, s), 8.46 (2/3H, s). ^{31}P NMR ($CDCl_3$) δ: 150.05, 153.07. HRMS (MALDI): Calcd. for C64H77N11O9P $[M + H]^+$ 1174.56379, Found 1174.56183.

4N-(N,N-Dimethylformamidyl)-1-[5-O-(4,4'-dimethoxytrityl)-2-(N,N'-dimethylguanydinyl)-4-C-methylene-β-D-ribopentofurano-syl]5-methylcytocine (**16-[Me,Me]**)

A solution of compound **15** (1.0 g, 1.6 mmol) and *N,N',S*-trimethylisothiourea (0.28 g, 2.4 mmol) in anhydrous *N,N*-dimethylacetamide (16 ml) was placed in an ice-bath. *N,N*-Diisopropylethylamine (0.42 mL, 2.4 mmol) and silver triflate (0.63 g, 2.5 mmol) were added and the mixture was stirred at 90 ºC for 4 h. After completion

of the reaction, mixture was diluted with ethyl acetate and sat. aq. NaCl was added. After filtration, the product was extracted with ethyl acetate, washed with water and brine, dried using Na_2SO_4, and concentrated. The product was purified by column chromatography using amino silica gel to afford **16-[Me,Me]** (757.9 mg, 68%) as an orange amorphous solid. IR (KBr): 3293, 3071, 2954, 2838, 1660, 1607, 1508, 1446, 1423, 1377, 1337, 1253, 1226, 1176, 1109, 1055, 1031 cm^{-1}; ^{1}H NMR ($CDCl_3$) δ: 1.95 (3H, d, J = 0.69 Hz), 3.00 (3H, s), 3.01 (3H, d, J = 1.7 Hz), 3.16 (3H, s), 3.17 (3H, s), 3.45 (1H, d, J = 10.0 Hz), 3.53 (2H, s), 3.79 (6H, s), 3.86 (1H, d, J = 10.0 Hz), 4.24 (1H, s), 4.50 (1H, br), 4.56 (1H, s), 5.56 (1H, s), 6.85 (4H, d, J = 8.6 Hz), 7.20–7.38 (7H, m), 7.43–7.49 (2H, m), 7.60 (1H, d, J = 1.0 Hz), 7.67 (1H, br), 8.73 (1H, s). ^{13}C NMR ($CDCl_3$) δ: 14.23, 30.58, 35.24, 41.43, 55.24, 55.68, 59.11, 63.98, 69.02, 86.34, 86.72, 88.55, 112.04, 113.34, 127.07, 128.00, 128.04, 130.04, 135.21, 135.31, 136.33, 144.34, 156.65, 158.17, 158.59, 158.66, 171.64. HRMS (MALDI): Calcd. for C38H46N7O6 $[M + H]^+$ 696.35041, Found 696.35084.

4N-(N,N-Dimethylformamidyl)-1-[5-O-(4,4'-dimethoxytrityl)-2-(N-methyl-N'-tert-butylguanydinyl)-4-C-methylene-β-D-ribopentofurano-syl]5-methylcytocine (**16-[Me,^{t}Bu]**)

A solution of compound **15** (1.0 g, 1.6 mmol) and *N*-*tert*-butyl-*N',S*-dimethylisothiourea (0.39 g, 2.4 mmol) in anhydrous *N,N*-dimethylacetamide (16 ml) was placed in an ice-bath. *N,N*-Diisopropylethylamine (0.42 mL, 2.4 mmol) and silver triflate (0.64 g, 2.5 mmol) were added and the mixture was stirred at 90 ºC for 4 h. After completion of the reaction, mixture was diluted with ethyl acetate and sat. aq. NaCl was added. After filtration, the product was extracted with ethyl acetate, washed with water and brine, dried using Na_2SO_4, and concentrated. The product was purified by column chromatography using amino silica gel to afford **16-[Me,^{t}Bu]** (555.9 mg, 64%) as an orange amorphous solid. IR (KBr): 3293, 3066, 3008, 2950, 1660, 1508, 1446, 1423, 1376, 1337, 1252, 1177, 1108, 1054, 1031 cm^{-1}; ^{1}H NMR ($CDCl_3$) δ: 1.46 (9H, s), 1.89 (3H, s), 2.92 (3H, s), 3.12 (3H, s), 3.14 (3H, s), 3.32 (1H, d, J = 9.7 Hz), 3.47 (1H, d, J = 10.9 Hz), 3.56 (1H, d, J = 10.9 Hz), 3.76 (6H, s), 3.78 (1H, d, J = 6.3 Hz), 4.30 (1H, s), 4.41 (1H, s), 5.52 (1H, s), 6.83 (4H, d, J = 8.6 Hz), 7.19–7.35 (7H, m), 7.45 (2H, d, J = 7.5 Hz), 7.57 (1H, s), 8.71 (1H, s). ^{13}C NMR ($CDCl_3$) δ: 14.18, 29.60, 31.43, 35.11, 41.28, 54.93, 55.17, 55.92, 59.26, 64.00, 68.97, 86.02, 86.63, 88.11, 111.62, 113.26, 118.74, 121.92, 126.96, 127.97, 127.99, 129.99, 130.02, 135.20, 135.35, 136.33, 144.31, 156.42, 156.69, 158.03, 158.56, 171.61. HRMS (MALDI): Calcd. for C41H52N7O6 $[M + H]^+$ 738.39736, Found 738.39915.

4N-(N,N-Dimethylformamidyl)-1-[3-O-{2-cyanoethoxy(diisopropylamino)phospino}-5-O-(4,4'-dimethoxy-trityl)-2-(N,N'-dimethylguanydinyl)-4-C-methylene-β-D-ribopentofuranosyl]5-methylcytocine (**17-[Me,Me]**)

N,N-Diisopropylethylamine (25 μL, 0.14 mmol) and 2-cyanoethyl-*N,N*-diisopropylchlorophosphoramidite (32 μL, 0.14 mmol) were added to the solution of **16-[Me,Me]** (83 mg, 0.12 mmol) in dichloromethane (1.2 mL), and the mixture was stirred at room temperature for 2 h. Then, *N,N*-diisopropylethylamine (25 μL, 0.14 mmol) and 2-cyanoethyl-*N,N*-diisopropylchlorophosphoramidite (32 μL, 0.14 mmol) were added again and the mixture

was stirred at room temperature for 2 h. After completion of the reaction, sat. aq. $NaHCO_3$ was added and the product was extracted with chloroform. The organic phase was washed with water and brine, dried using Na_2SO_4, and concentrated. The product was purified by column chromatography using amino silica gel to afford **17-[Me,Me]** (71.5 mg, 67%) as a yellow amorphous solid. ^{1}H NMR ($CDCl_3$) *δ*: 0.99 (6H, d, *J* = 6.9 Hz), 1.11 (6H, d, *J* = 6.9 Hz), 1.93 (3H, d, *J* = 0.9 Hz), 2.32 (2H, t, *J* = 6.0 Hz), 3.05 (6H, s), 3.19 (6H, s), 3.40–3.78 (8H, m), 3.80 (6H, s), 4.36 (1H, d, *J* = 8.7 Hz), 4.39 (1H, s), 5.64 (1H, s), 6.86 (4H, dd, *J* = 2.3, 9.2 Hz), 7.22–7.35 (7H, m), 7.41–7.46 (2H, m), 7.65 (1H, d, *J* = 0.9 Hz), 8.81 (1H, s). ^{31}P NMR ($CDCl_3$) δ: 150.59, 150.76. HRMS (MALDI): Calcd. for C47H63N9O7P $[M + H]^+$ 896.45826, Found 896.46128.

4N-(N,N-Dimethylformamidyl)-1-[3-O-{2-cyanoethoxy(diisopropylamino)phospino}-5-O-(4,4'-dimethoxy-trityl)-2-(N-methyl-N'-tert-butylguanydinyl)-4-C-methylene-β-D-ribopentofuranosyl]5-methylthymine (**17-[Me,^{t}Bu]**)

N,N-Diisopropylethylamine (0.16 mL, 0.92 mmol) and 2-cyanoethyl-*N,N*-diisopropylchlorophosphoramidite (0.20 mL, 0.90 mmol) were added to the solution of **16-[Me,^{t}Bu]** (0.56 g, 0.75 mmol) in dichloromethane (7.5 mL), and the mixture was stirred at room temperature for 1.5 h. Then, *N,N*-diisopropylethylamine (80 µL, 0.46 mmol) and 2-cyanoethyl-*N,N*-diisopropylchlorophosphoramidite (0.10 mL, 0.45 mmol) were added again and the mixture was stirred at room temperature for 30 min. After completion of the reaction, sat. aq. $NaHCO_3$ was added and the product was extracted with chloroform. The organic phase was washed with water and brine, dried using Na_2SO_4, and concentrated. The product was purified by column chromatography to afford **17-[Me,^{t}Bu]** (398.1 mg, 56%) as a yellow amorphous solid. ^{1}H NMR ($CDCl_3$) *δ*: 1.00 (6H, d, *J* = 6.9 Hz), 1.12 (6H, d, *J* = 6.9 Hz), 1.51 (9H, s), 1.85 (3H, s), 2.22–2.35 (2H, m), 2.97 (3H, s), 3.17 (6H, s), 3.34–3.77 (8H, m), 3.79 (6H, s), 4.42 (1H, d, *J* = 8.7 Hz), 4.46 (1H, s), 5.62 (1H, s), 6.86 (4H, dd, *J* = 3.2, 8.7 Hz), 7.22–7.34 (7H, m), 7.43–7.45 (2H, m), 7.70 (1H , s), 8.80 (1H, s). ^{31}P NMR ($CDCl_3$) δ: 150.98. HRMS (MALDI): Calcd. for C50H69N9O7P $[M + H]^+$ 938.50521, Found 938.50299.

4N-(N,N-Dimethylformamidyl)-5-methylcytocine (**19**)

A solution of compound **18** (3.1 g, 19 mmol) in anhydrous DMF (190 ml) was placed in an ice-bath. *N,N*-diisopropylethylamine (3.3 ml, 19 mmol) and *N,N*-dimethylformamide-dimethyl acetal (5.1 ml, 39 mmol) were added and the mixture was stirred at room temperature for 2 h. After completion of the reaction, a precipitate was filtrated, and the solution phase was concentrated. The residue was suspended with 1:1 mixture of ethyl acetate and tert-butyl methyl ether, filtrated again, and combined with before filtrate to afford **19** (3.89 mg, quant.) as a white powder. IR (KBr): 3161, 2960, 2924, 2781, 1665, 1607, 1488, 1466, 1426, 1390, 1373, 1336, 1262, 1175, 1113 cm^{-1}; ^{1}H NMR (DMSO-d_6) *δ*: 1.87 (3H, s), 3.04 (3H, s), 3.14 (3H, s), 7.33 (1H, s), 8.52 (1H, s), 10.68 (1H, s). ^{13}C NMR (DMSO-d_6) δ:13.55, 34.67, 40.81, 107.67, 141.16, 157.20, 157.28, 171.40. HRMS (MALDI): Calcd. for C8H13N4O $[M + H]^+$ 181.10839, Found 181.10845.

4N-(N,N-Dimethylformamidyl)-1-[5-O-(4,4'-dimethoxytrityl)-3-O-trimethylsilyl-4-C-methylene-β-D-ribopento-furanosyl]-5-methylcytocine (**20**)

A suspension of compound **1** (4.2 g, 7.4 mmol) and compound **19** (4.0 g, 22 mmol) in 1,2-dichloroethane (74 ml) was placed in an ice-bath under stirring. *N,O*-Bis(trimethylsilyl)acetamide (16 ml, 66 mmol) was added and the mixture was stirred at 40 ºC for 30 min. Then, the mixture was cooled in an ice-bath, and trimethylsilyl trifluoromethanesulfonate (0.20 mL, 1.1 mmol) was dropped into the mixture. The mixture was heated again to 50 ºC, and stirred for 4.5 h. After completion of the reaction, sat. aq. $NaHCO_3$ was added and the product was extracted with chloroform. The organic phase was washed with brine, dried using Na_2SO_4, and concentrated. The product was dissolved in ethyl acetate, washed again with water and brine, dried using Na_2SO_4, and concentrated. The product was purified by column chromatography to afford **20** (3.44 mg, 67%) as a white amorphous solid. IR (KBr): 2952, 1661, 1606, 1508, 1445, 1425, 1394, 1375, 1335, 1306, 1253, 1177, 1151, 1108, 1083, 1055 cm^{-1}; ^1H NMR ($CDCl_3$) *δ*: 0.01 (9H, s), 1.87 (3H, s), 2.82 (1H, d, *J* = 10.0 Hz), 2.95 (1H, d, *J* = 10.0 Hz), 3.16 (3H, s), 3.17 (3H, s), 3.19 (1H, d, *J* = 10.7 Hz), 3.50 (1H, d, *J* = 11.0 Hz), 3.71 (1H, s), 3.80 (6H, s) 4.24 (1H, s), 5.66 (1H, s), 6.84 (4H, dd, *J* = 2.4, 8.9 Hz), 7.20–7.41 (7H, m), 7.47–7.52 (2H, m), 8.01 (1H, s), 8.82 (1H, s). ^{13}C NMR ($CDCl_3$) δ: 0.03, 14.33, 34.93, 41.20, 50.68, 55.20, 59.45, 62.04, 70.32, 86.21, 88.30, 89.63, 109.73, 113.13, 113.19, 126.87, 127.90, 128.10, 129.99, 130.07, 135.76, 135.79, 138.27, 144.69, 156.15, 157.93, 158.50, 171.04. HRMS (MALDI): Calcd. for C38H47N5O6NaSi $[M + Na]^+$ 720.31878, Found 720.31767.

4N-(N,N-Dimethylformamidyl)-1-[5-O-(4,4'-dimethoxytrityl)-4-C-methylene-β-D-ribopento-furanosyl]-5-methylcytocine (**15**)

A solution of compound **20** (42 mg, 0.060 mmol) in anhydrous tetrahydrofuran (540 μL) was placed in an ice-bath. 1 M Tetrabutylammonium fluoride in tetrahydrofuran (60 μL, 0.060 mmol) was added and the mixture was stirred at 0 ºC for 30 min. After completion of the reaction, sat. aq. NaCl was added and the product was extracted with chloroform. The organic phase was dried using Na_2SO_4, and concentrated. The product was purified by column chromatography to afford **15** (35.9 mg, 96%) as a white amorphous solid. IR (KBr): 3263, 2934, 1660, 1605, 1508, 1444, 1376, 1335, 1305, 1252, 1176, 1153, 1116, 1053 cm^{-1}; ^1H NMR ($CDCl_3$) *δ*: 1.84 (3H, s), 2.93 (1H, d, *J* = 10.3 Hz), 2.97 (1H, d, *J* = 10.3 Hz), 3.14 (6H, s), 3.43 (1H, d, *J* = 11.0 Hz), 3.49 (1H, d, *J* = 11.0 Hz), 3.75 (1H, s), 3.79 (6H, s), 4.18 (1H, s), 5.65 (1H, s), 6.85 (4H, d, *J* = 8.3 Hz), 7.18–7.42 (7H, m), 7.47–7.52 (2H, m), 7.83 (1H, s), 8.75 (1H, s). ^{13}C NMR ($CDCl_3$) δ: 14.12, 34.91, 41.10, 49.87, 55.15, 59.88, 61.74, 69.77, 86.27, 88.22, 89.20, 110.01, 113.15, 126.78, 127.87, 128.12, 130.07, 135.70, 135.77, 138.21, 144.72, 156.20, 157.77, 158.43, 170.97. HRMS (MALDI): Calcd. for. C35H39N5O6Na $[M + Na]^+$ 648.27926, Found 648.28019.

3. ^{1}H NMR, ^{13}C NMR and ^{31}P NMR spectra of new compounds

Compound **2-[Et]** (^{1}H NMR, DMSO-d_6, 80 ºC, 301 MHz)

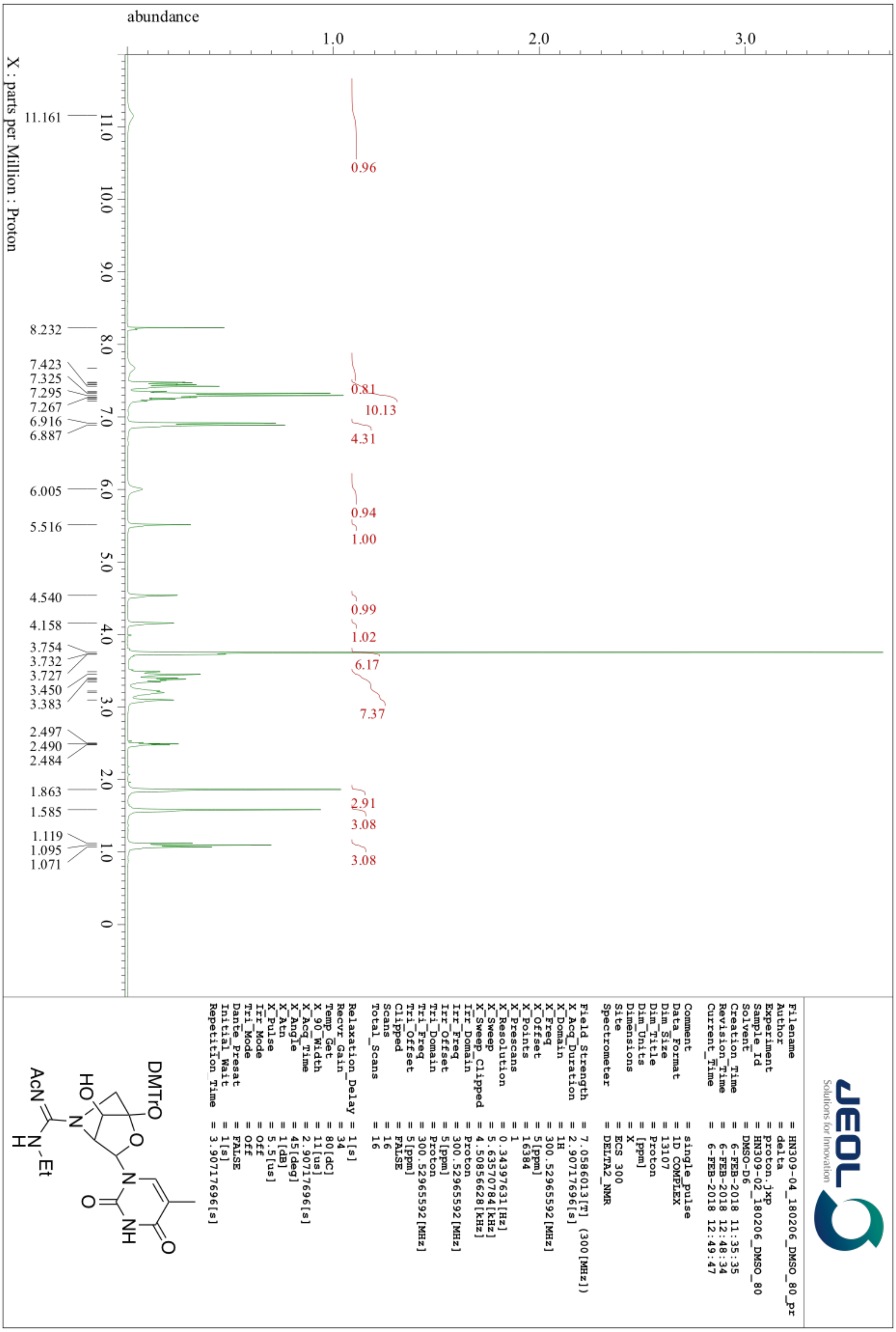

Compound **2-[Et]** (^{13}C NMR, $CDCl_3$, 80 ºC, 76 MHz)

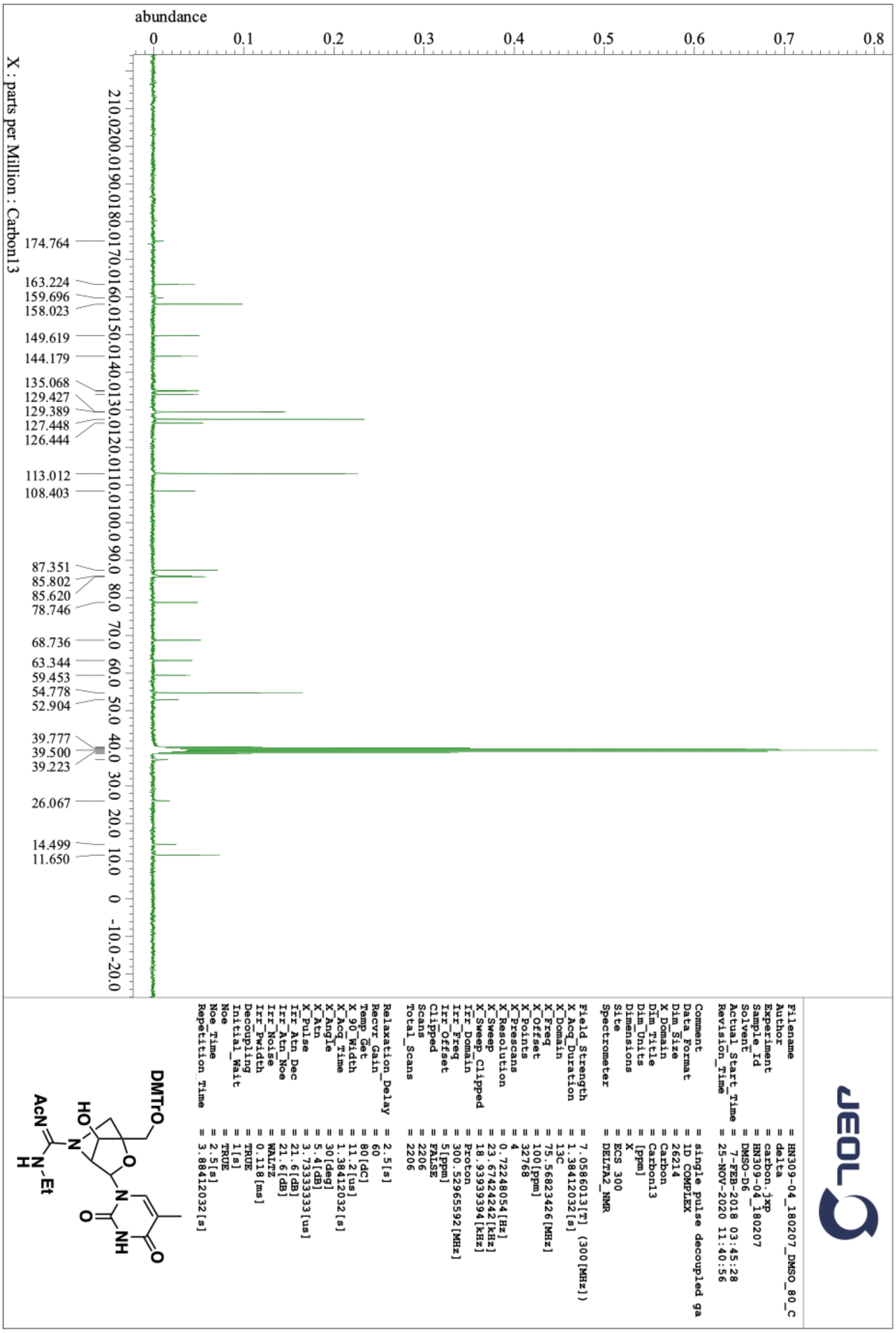

Compound **2-[^{i}Pr]** (^{1}H NMR, DMSO-d_6, 80 ºC, 301 MHz)

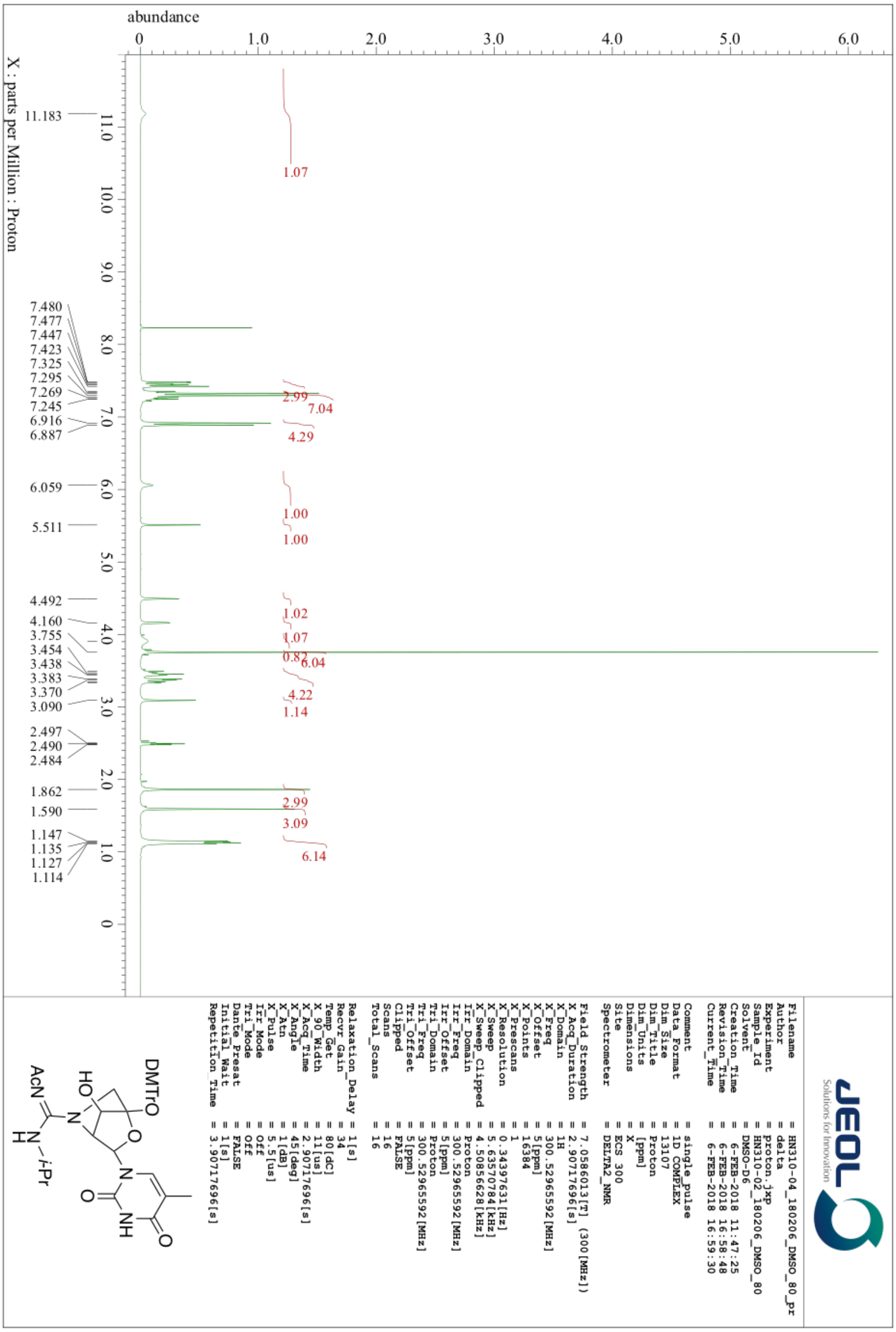

Compound **2-[^{i}Pr]** (^{13}C NMR, DMSO-d_6, 80 ºC, 76 MHz)

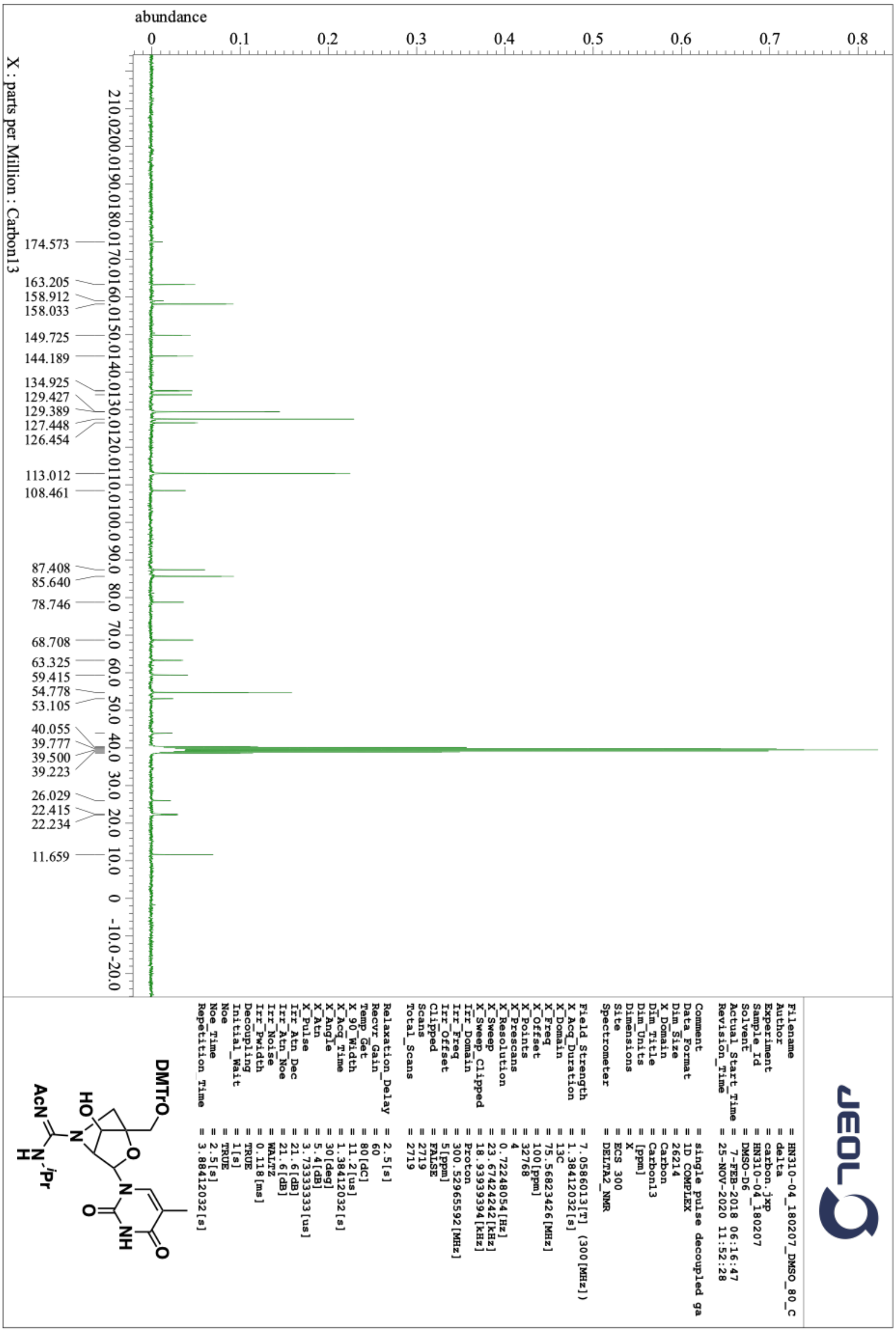

Compound **2-[^{t}Bu]** (^{1}H NMR, $CDCl_3$, 301 MHz)

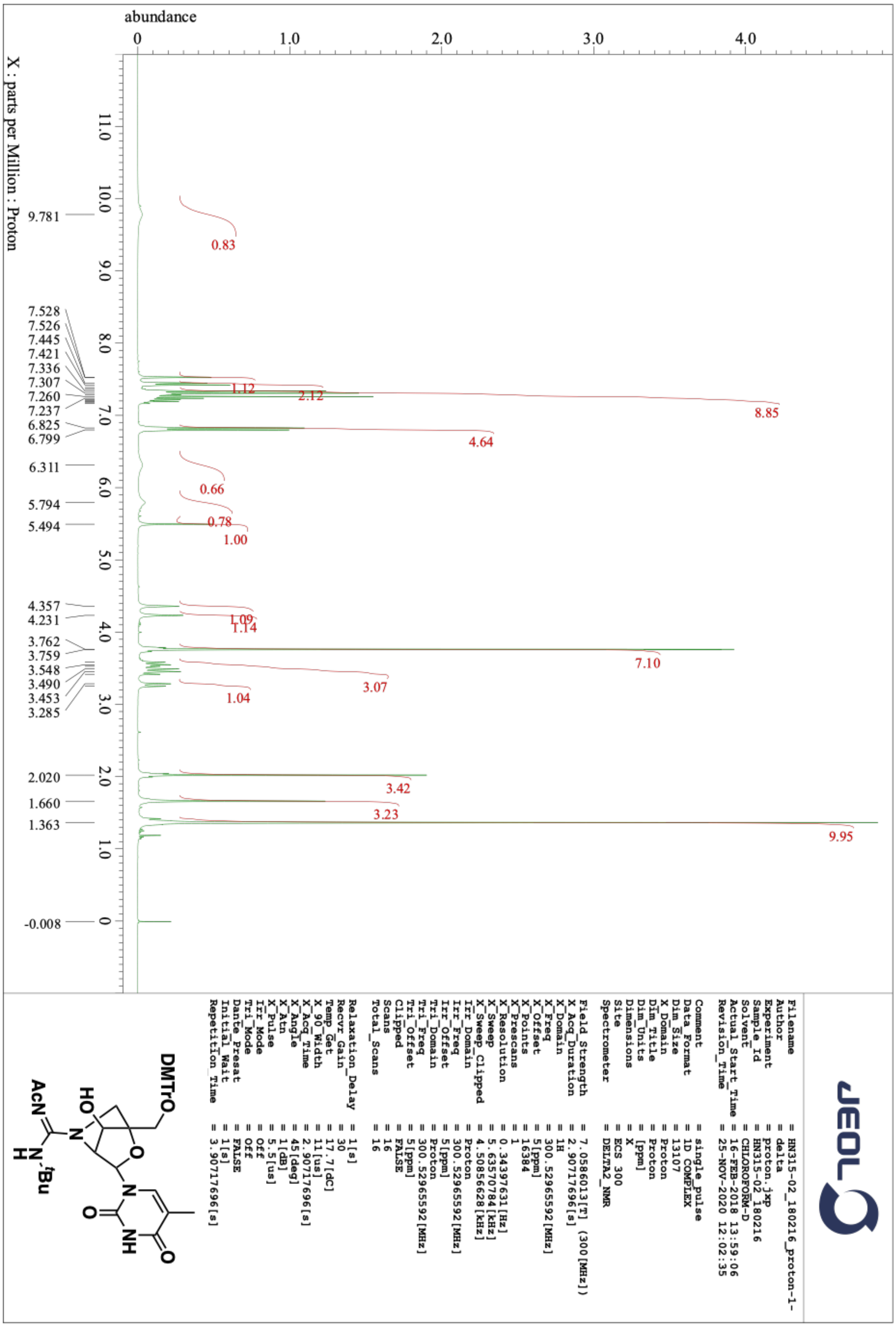

Compound **2-[^{t}Bu]** (^{13}C NMR, $CDCl_3$, 76 MHz)

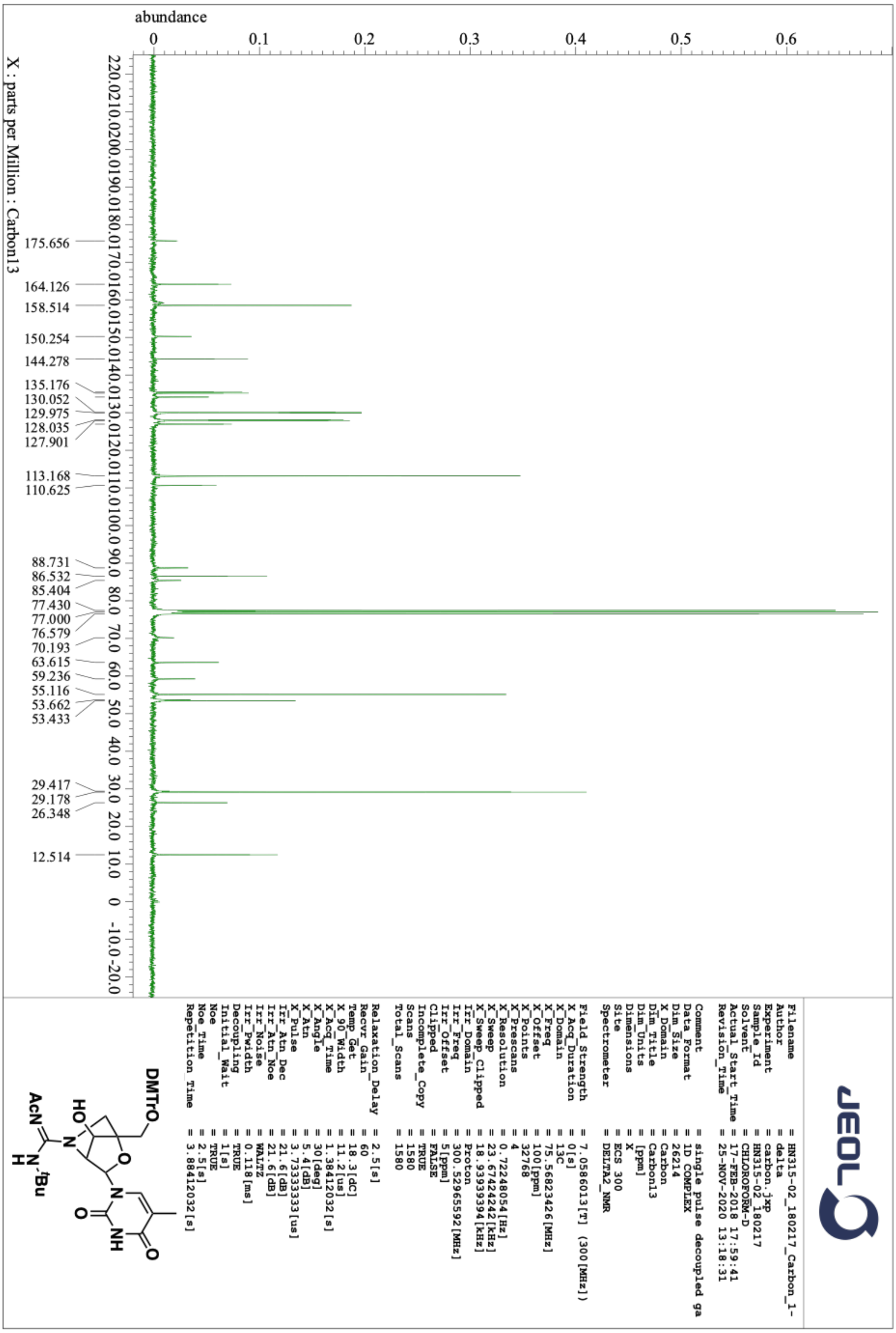

Compound **2-[Me,Me]** (^{1}H NMR, DMSO-d_6, 301 MHz)

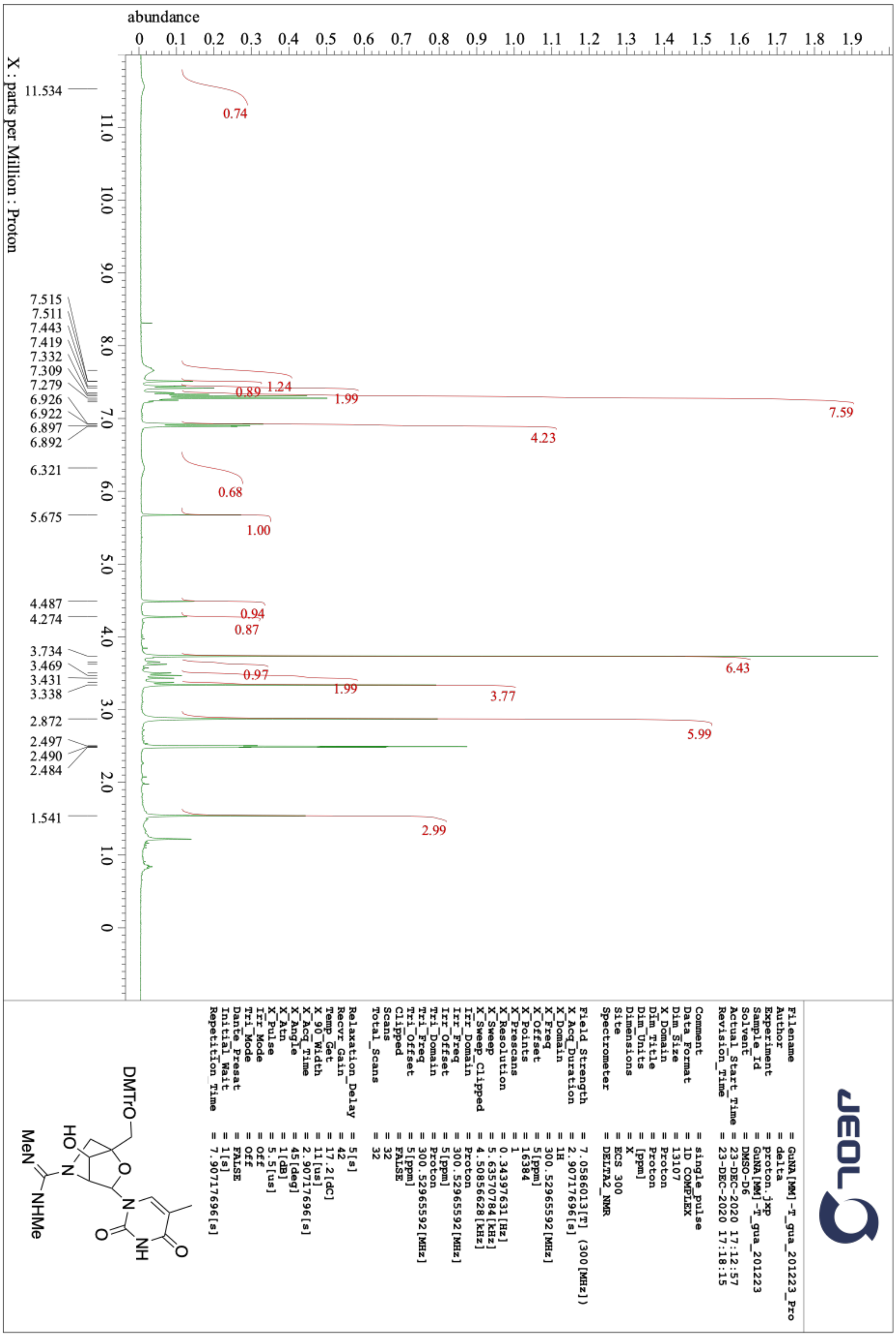

Compound **2-[Me,Me]** (^{13}C NMR, DMSO-d_6, 101 MHz)

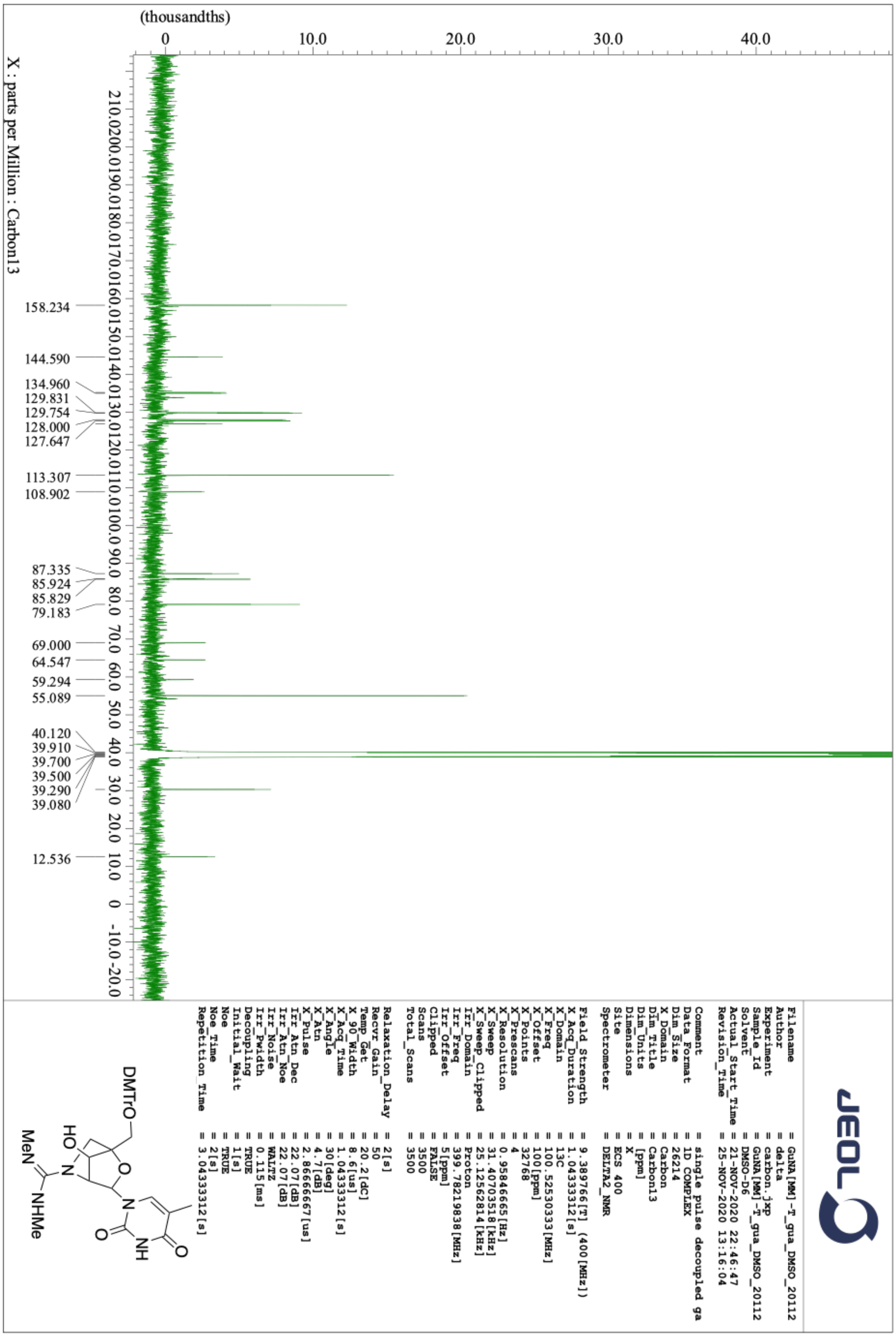

Compound **2-[Me,^{t}Bu]** (^{1}H NMR, $CDCl_3$, 301 MHz)

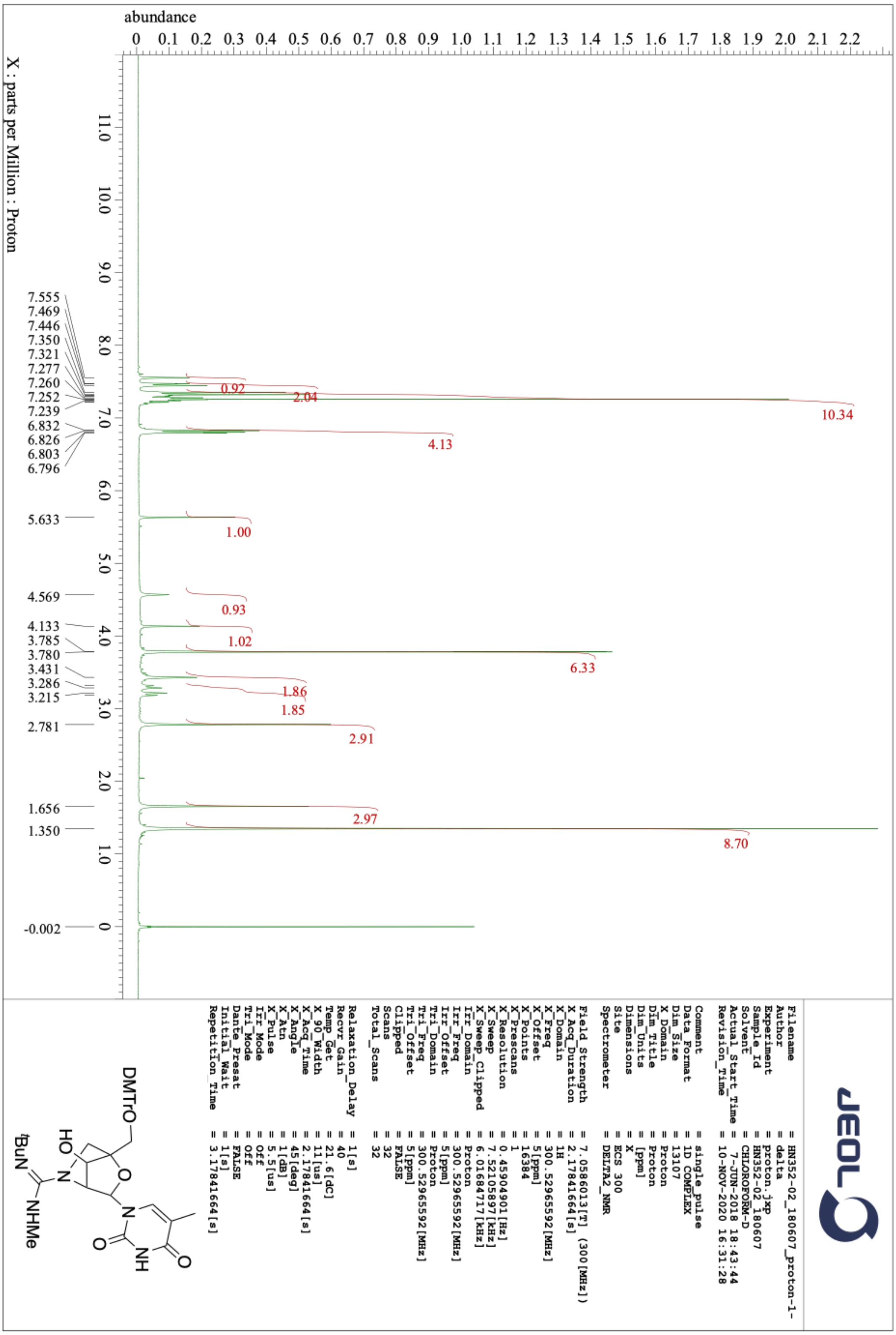

Compound **2-[Me,^{t}Bu]** (^{13}C NMR, $CDCl_3$, 101 MHz)

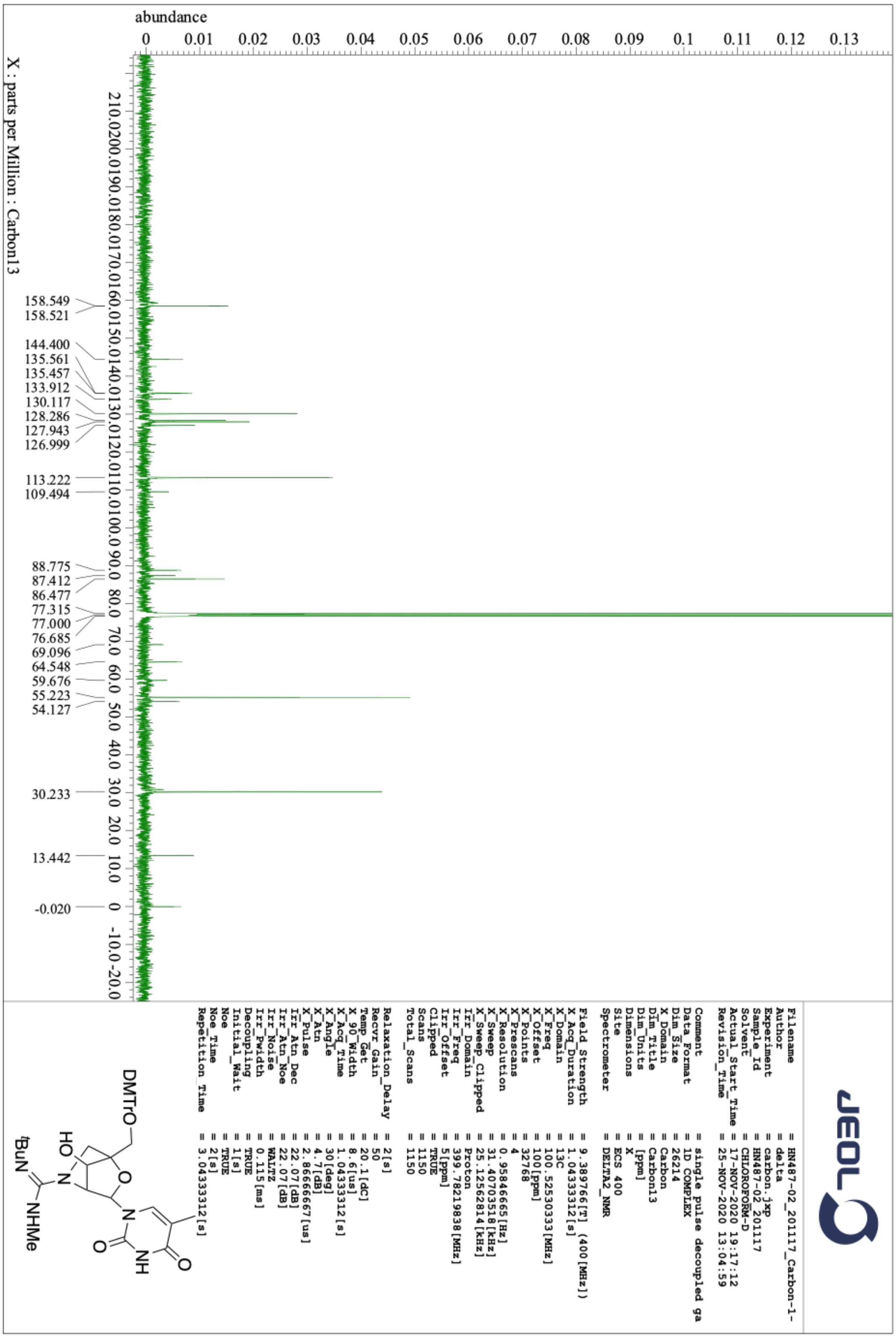

Compound **3-[Et]** (^{1}H NMR, $CDCl_3$, 400 MHz)

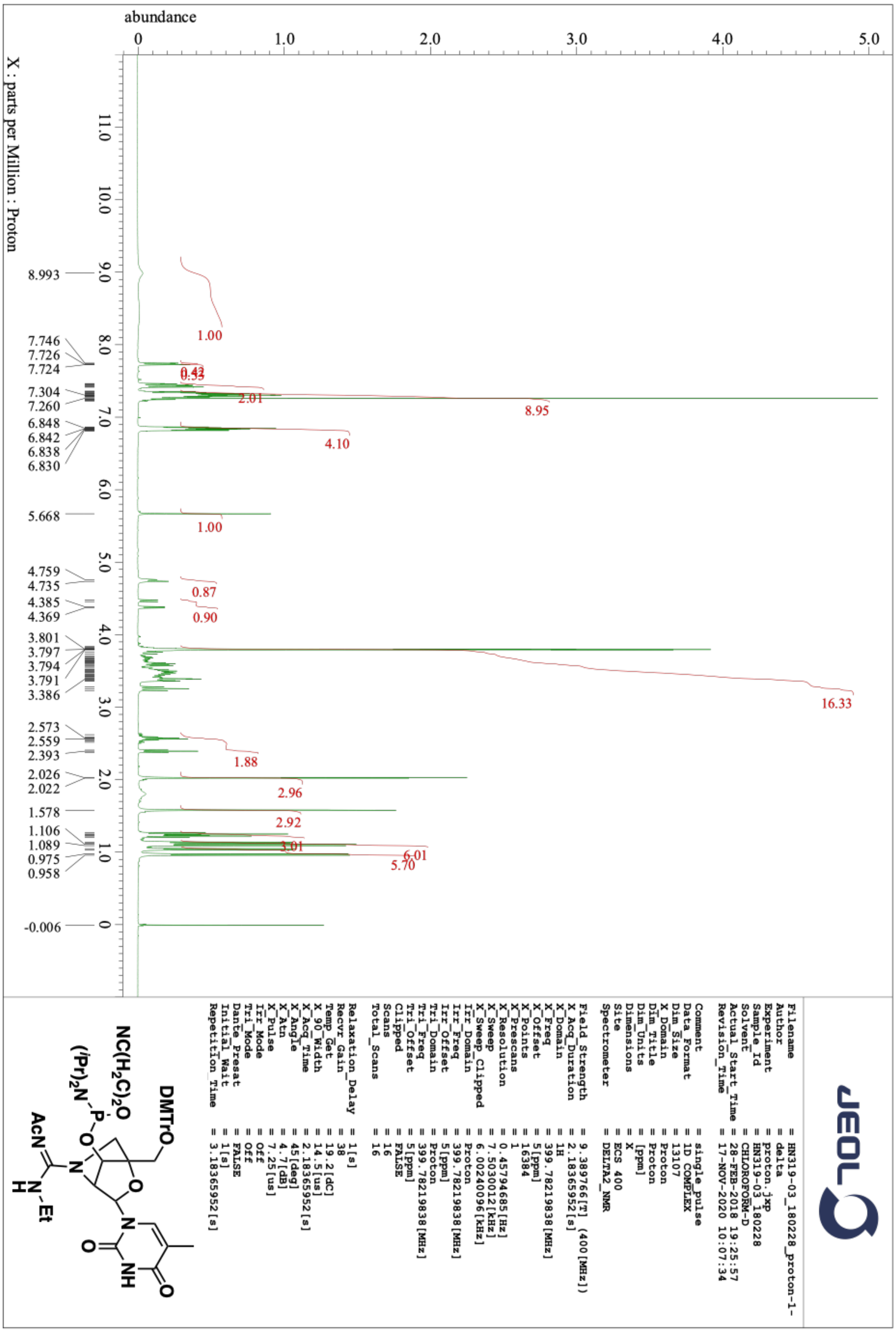

Compound **3-[Et]** (^{31}P NMR, $CDCl_3$, 122 MHz)

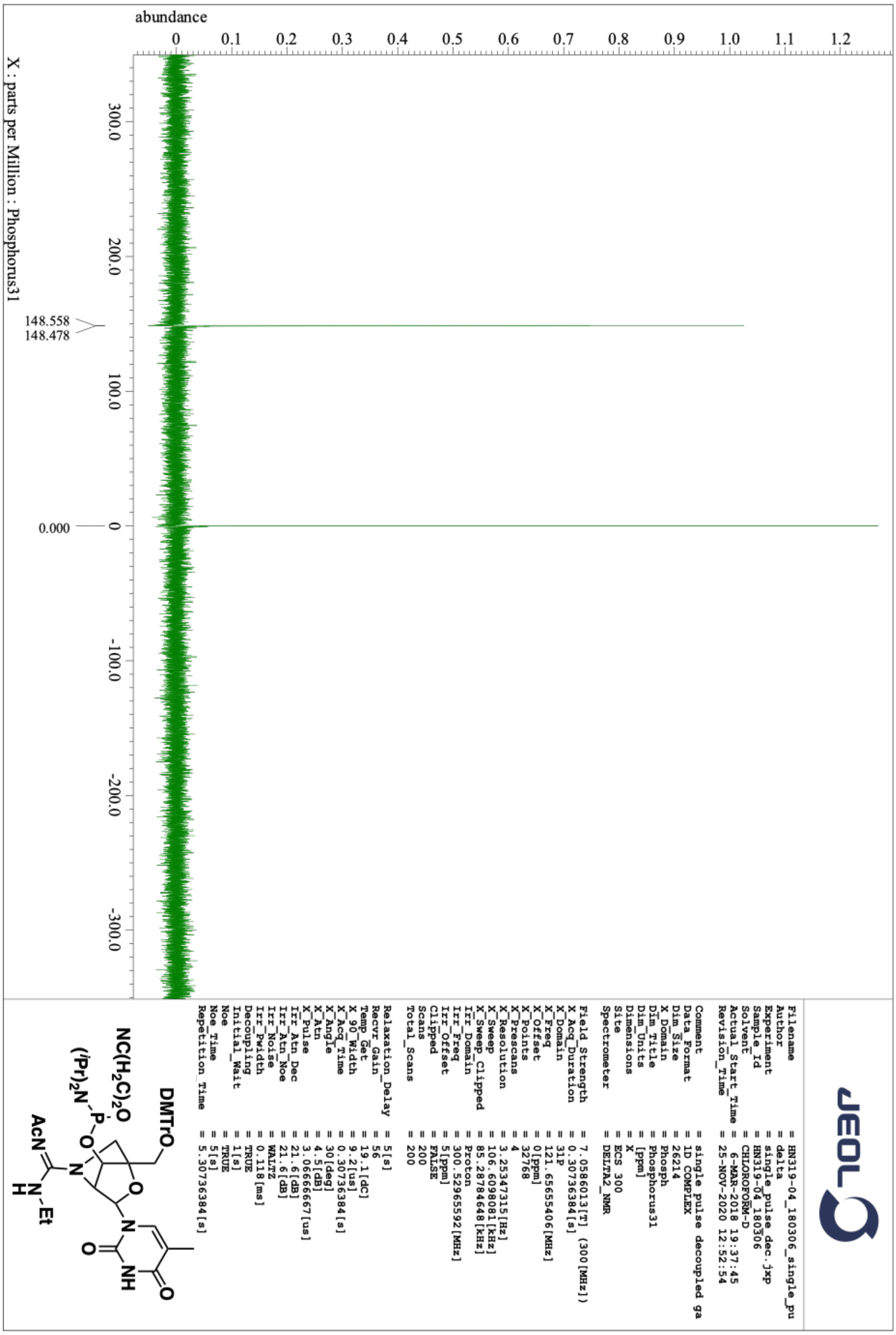

Compound **3-[^{i}Pr]** (^{1}H NMR, $CDCl_3$, 301 MHz)

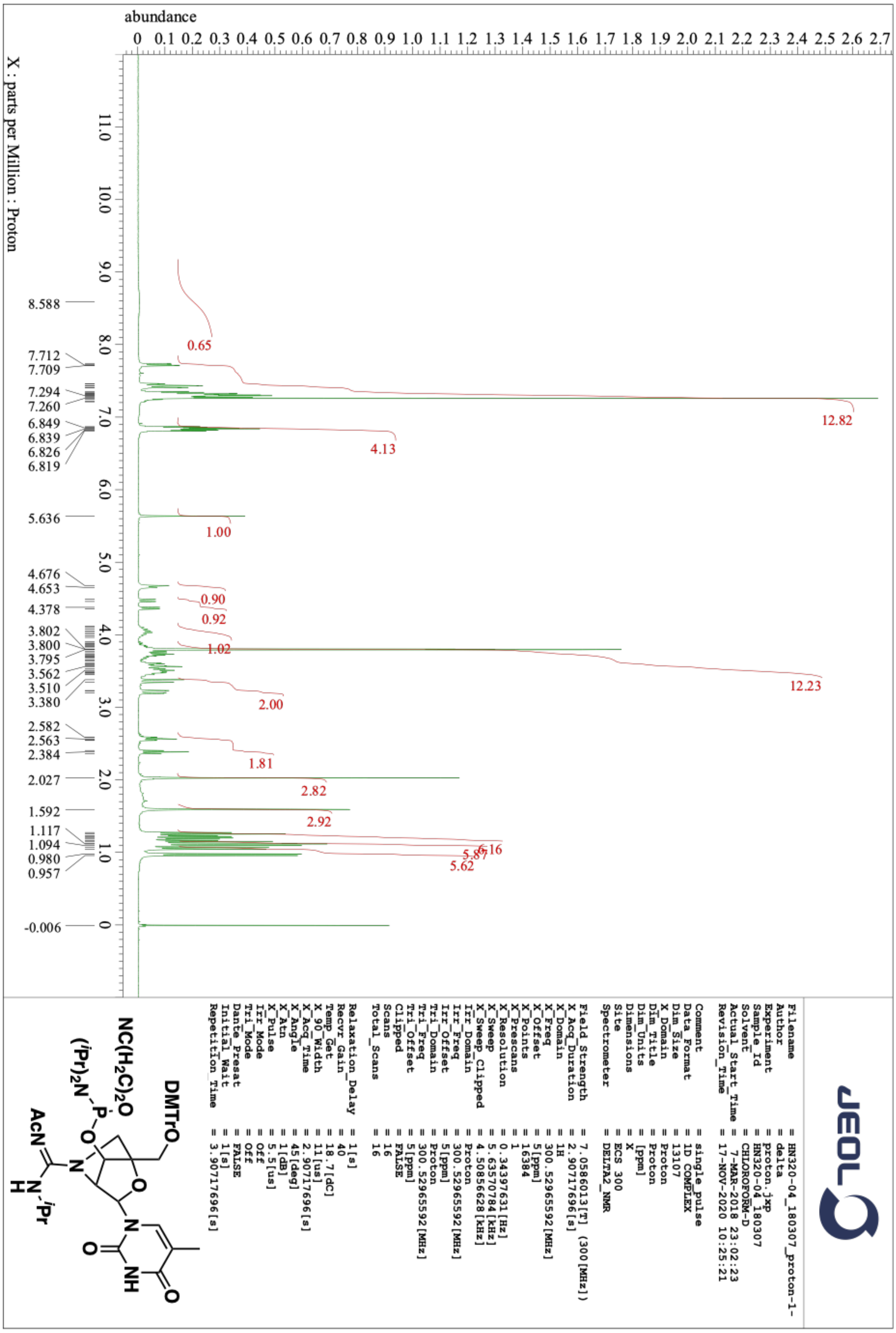

Compound **3-[^{i}Pr]** (^{31}P NMR, $CDCl_3$, 122 MHz)

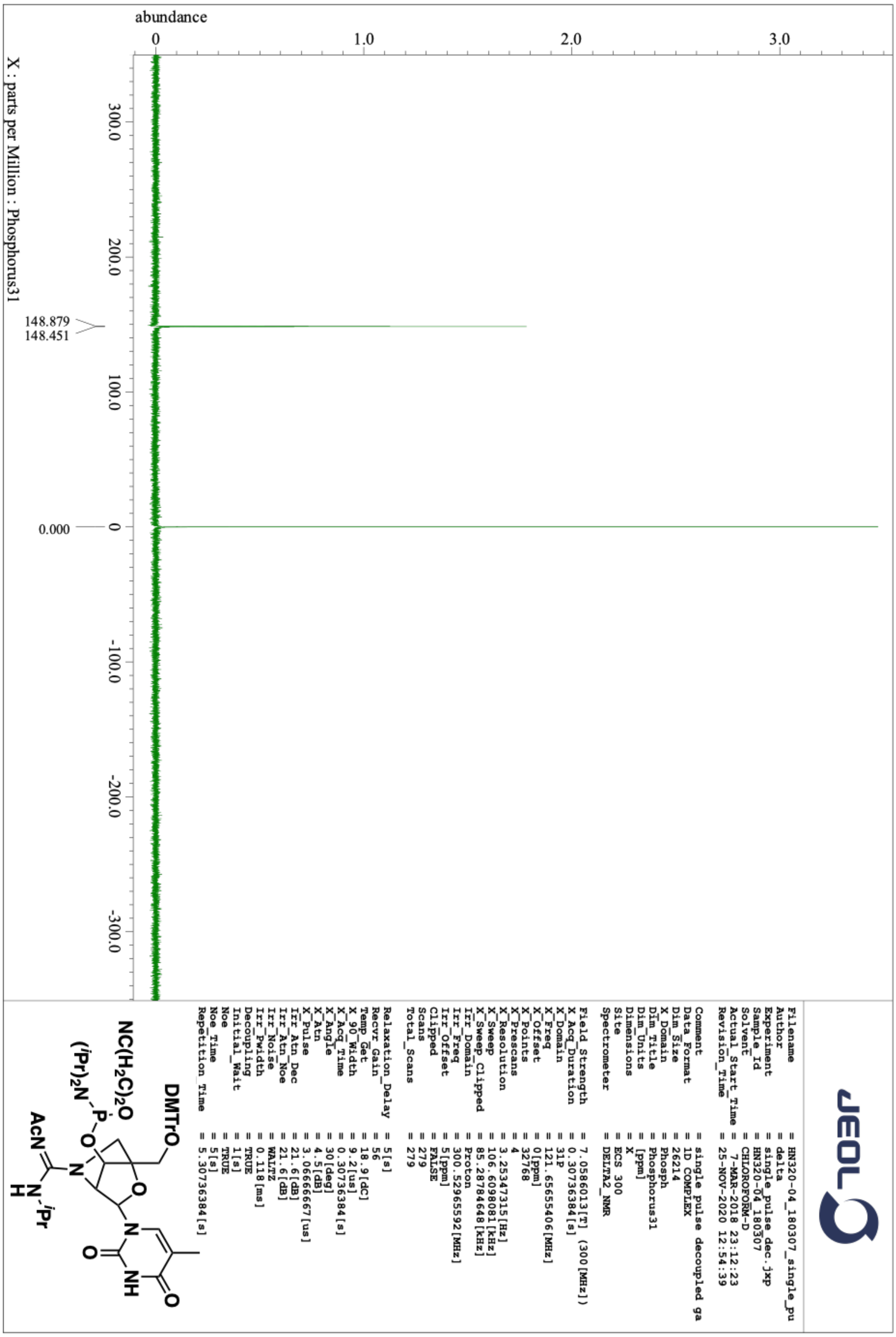

Compound **3-[^{t}Bu]** (^{1}H NMR, $CDCl_3$, 500 MHz)

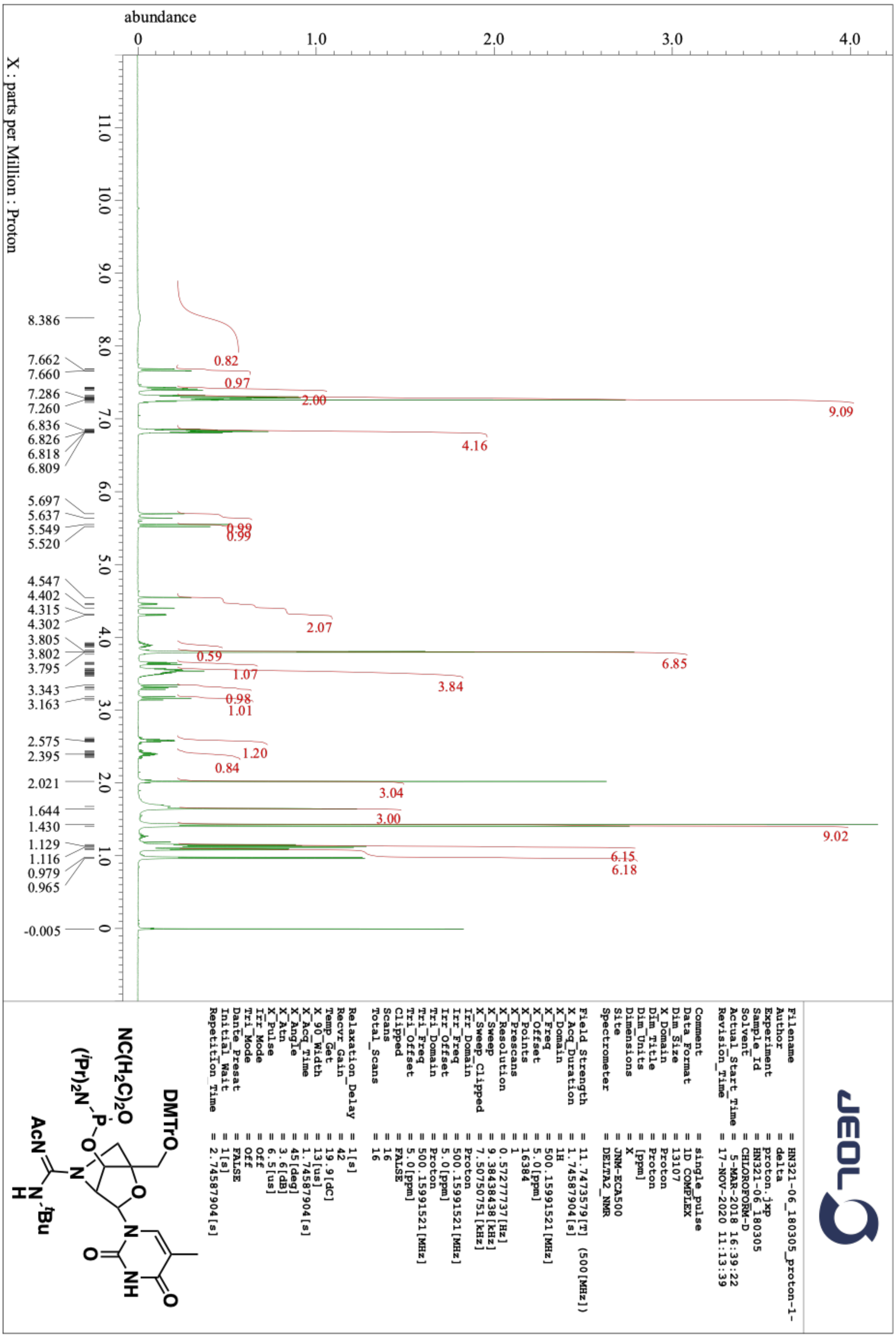

Compound **3-[^{t}Bu]** (^{31}P NMR, $CDCl_3$, 202 MHz)

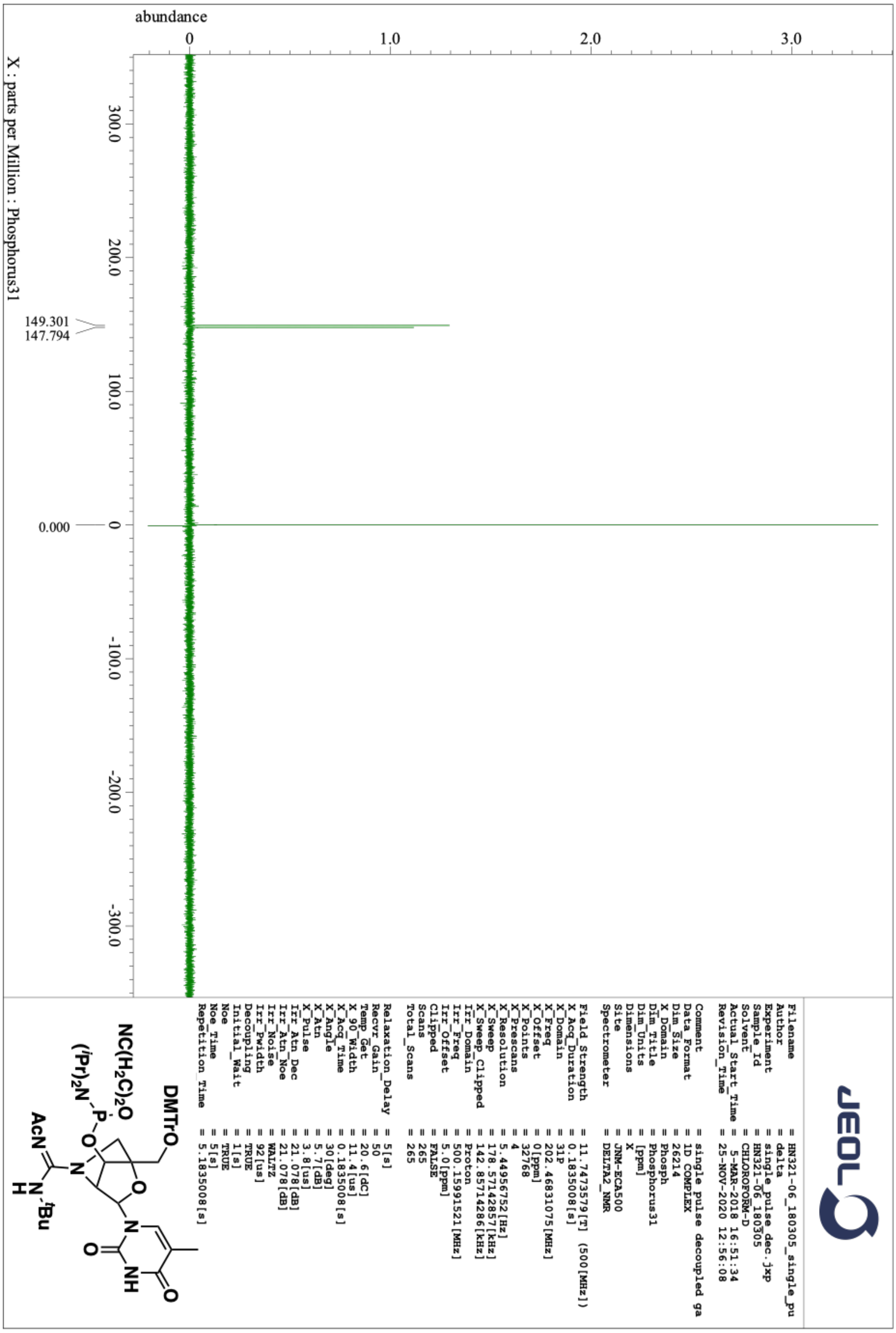

Compound **3-[Me,Me]** (^{1}H NMR, $CDCl_3$, 301 MHz)

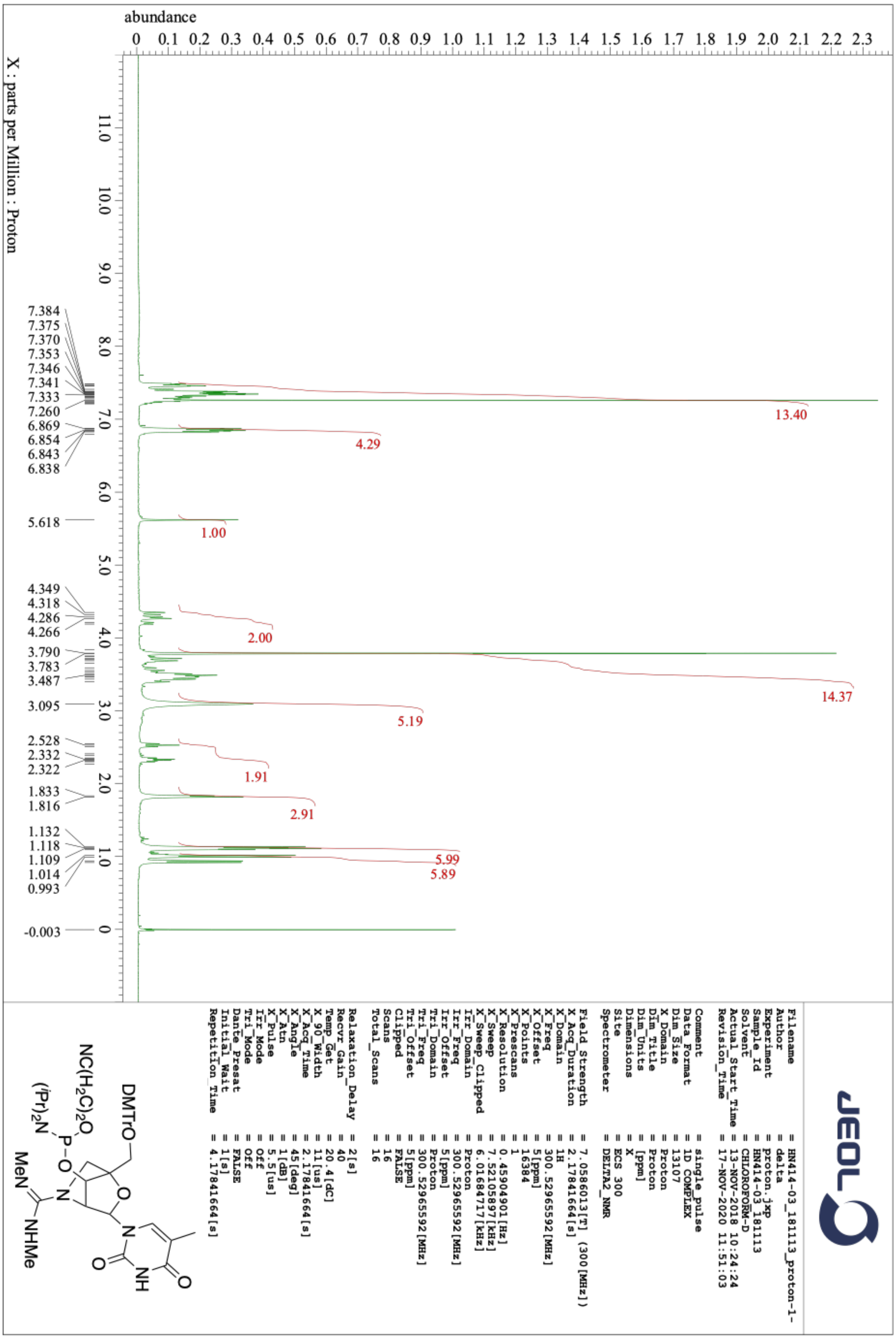

Compound **3-[Me,Me]** (^{31}P NMR, $CDCl_3$, 122 MHz)

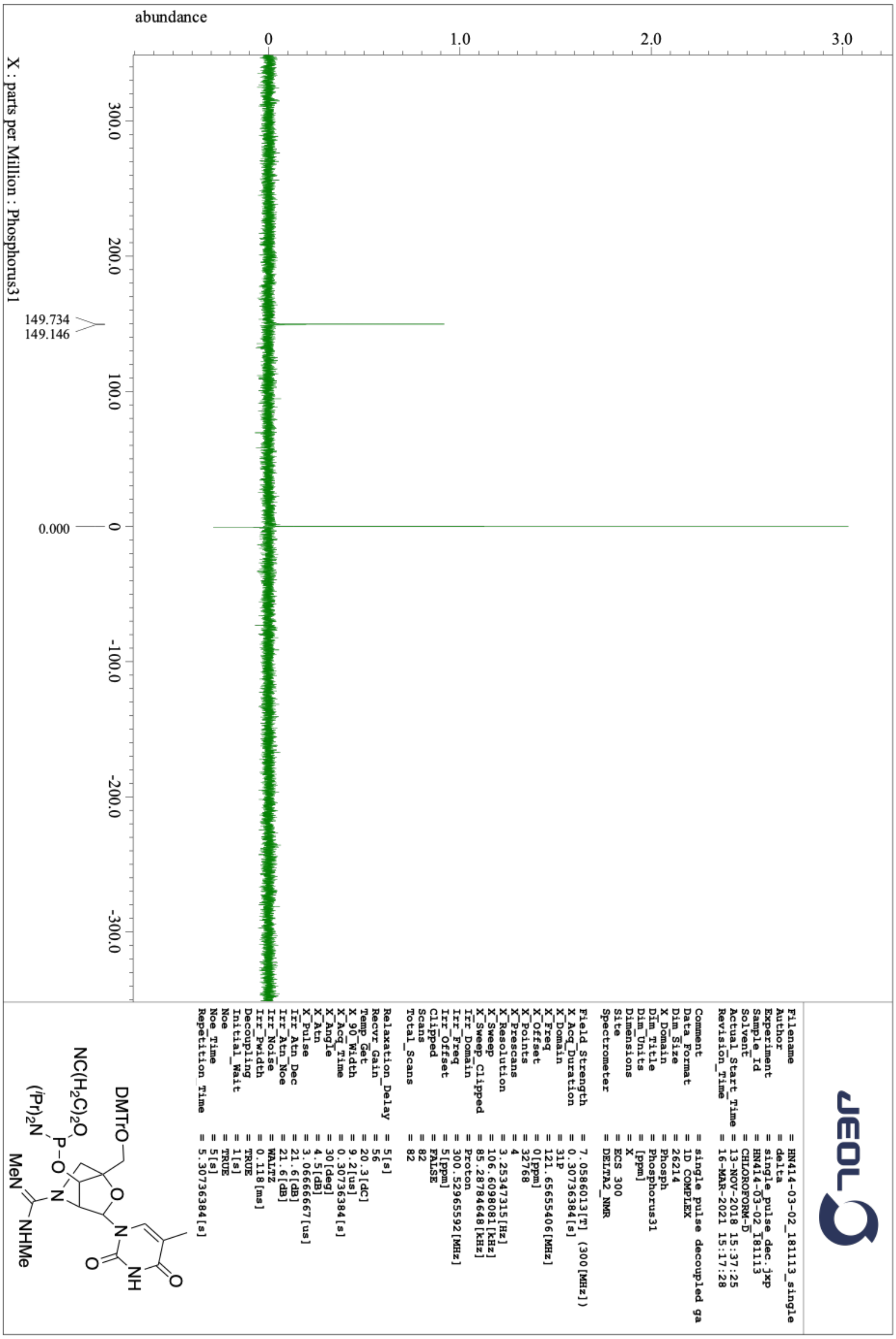

Compound **3-[Me,^{t}Bu]** (^{1}H NMR, $CDCl_3$, 301 MHz)

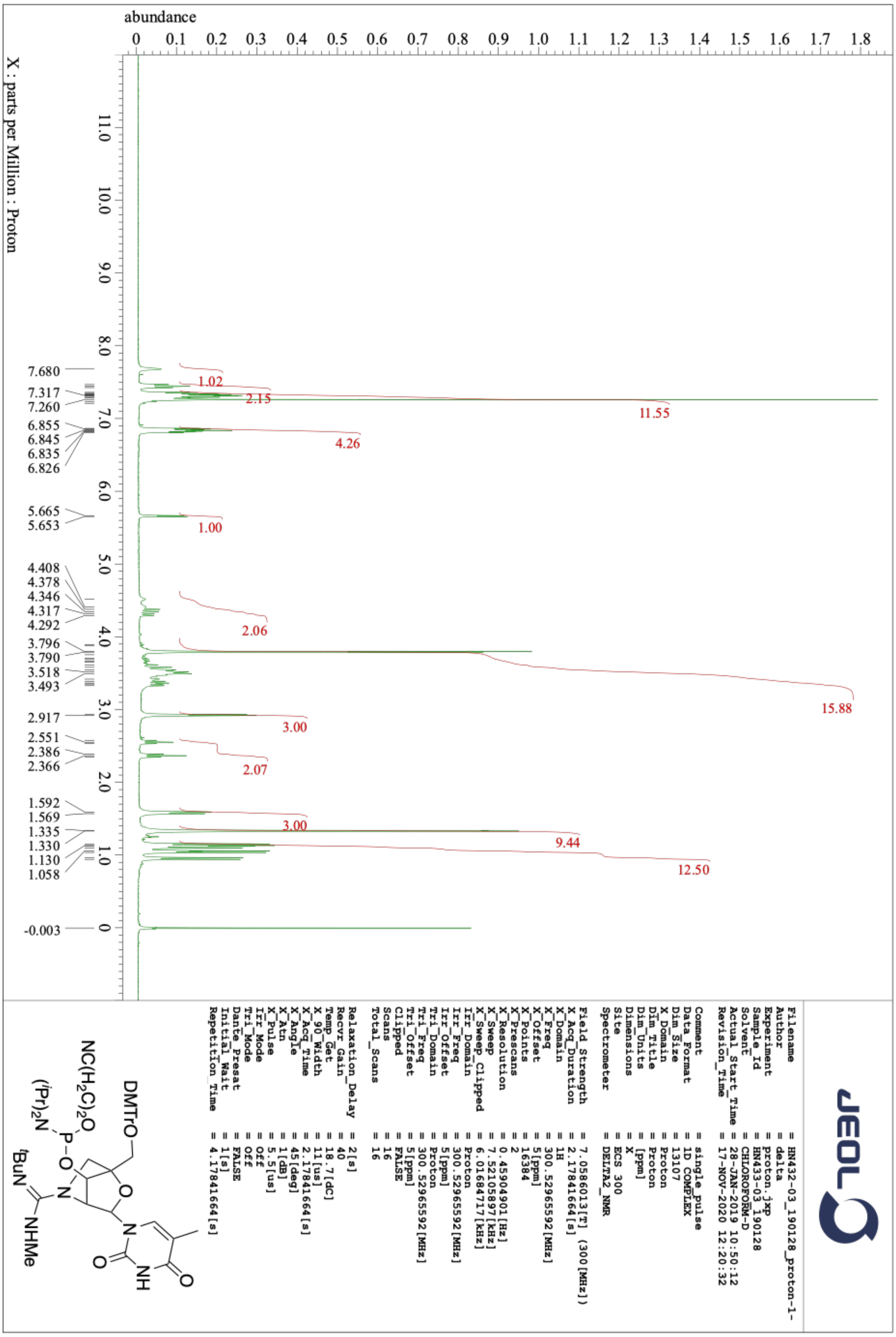

Compound **3-[Me,^{t}Bu]** (^{31}P NMR, $CDCl_3$, 122 MHz)

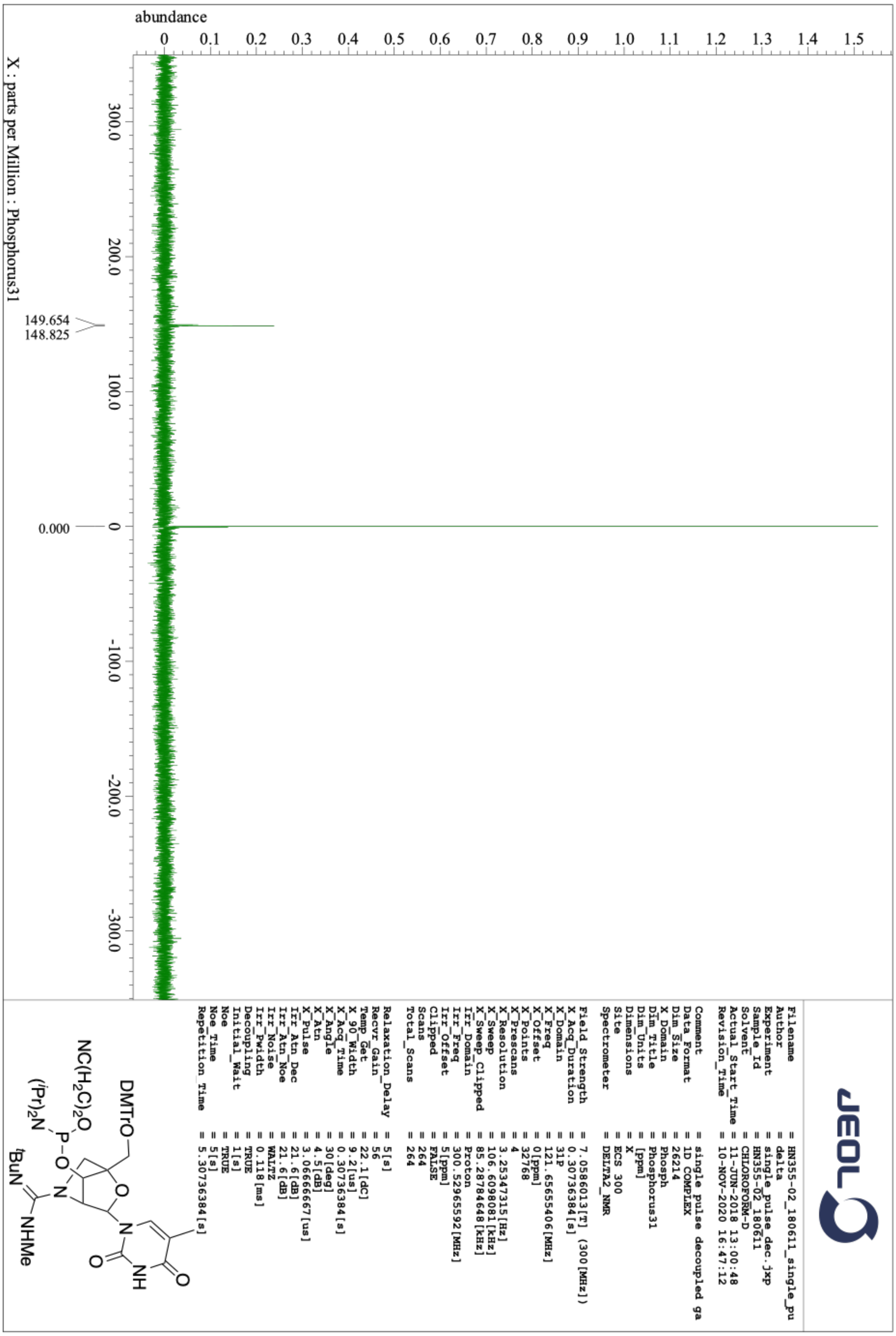

Compound **4** (^{1}H NMR, $CDCl_3$, 301 MHz)

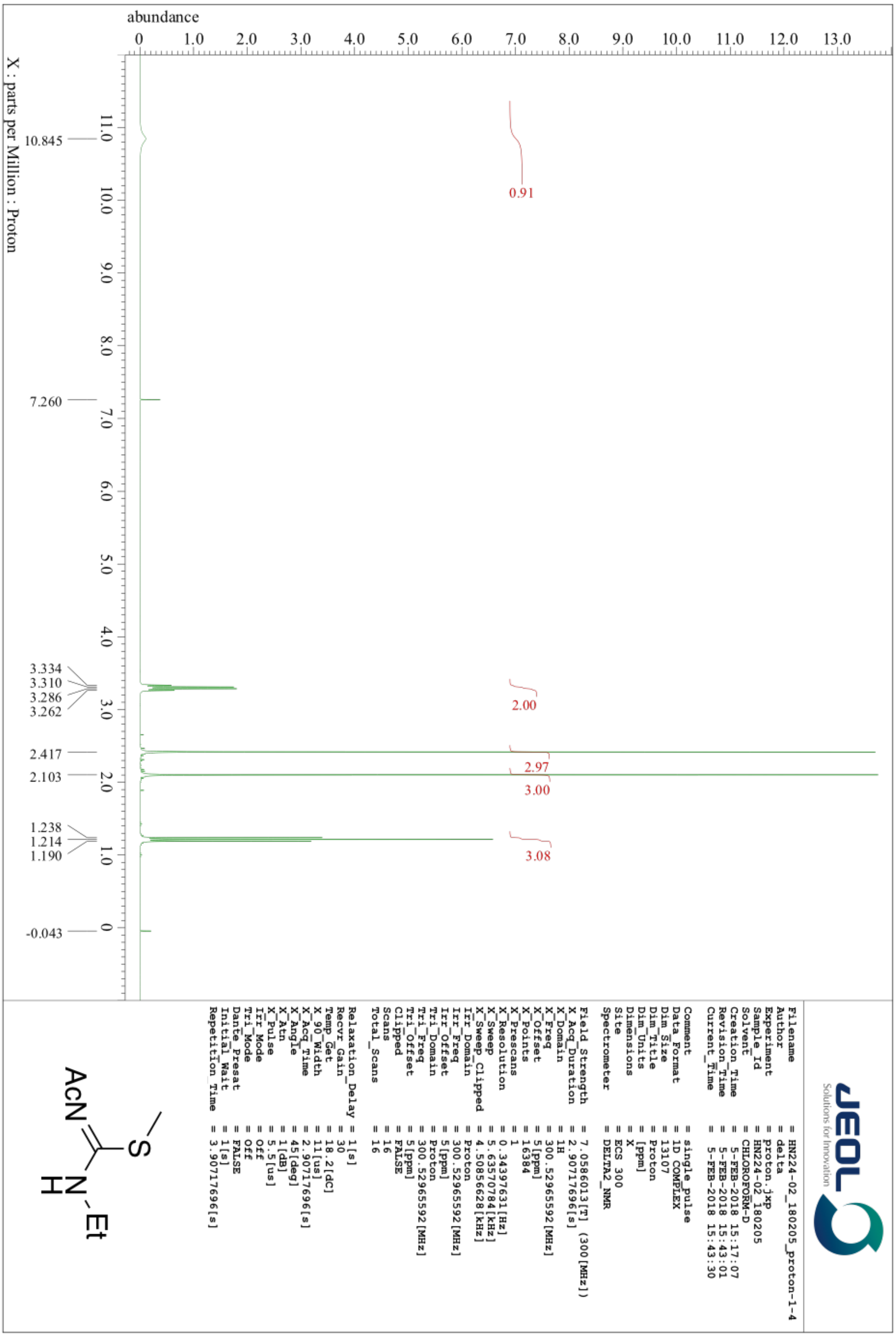

Compound **4** (^{13}C NMR, $CDCl_3$, 76 MHz)

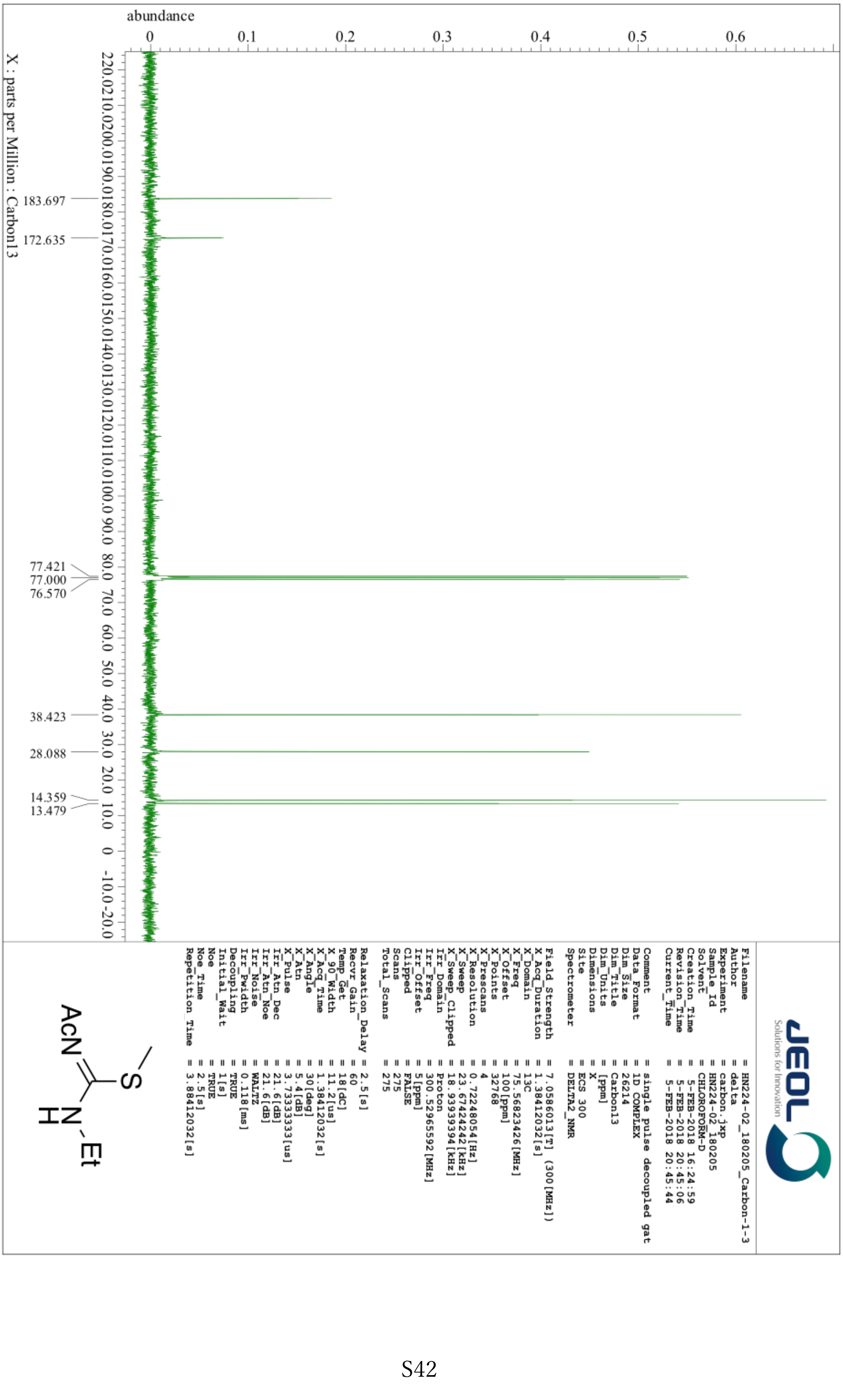

Compound **5** (^{1}H NMR, $CDCl_3$, 301 MHz)

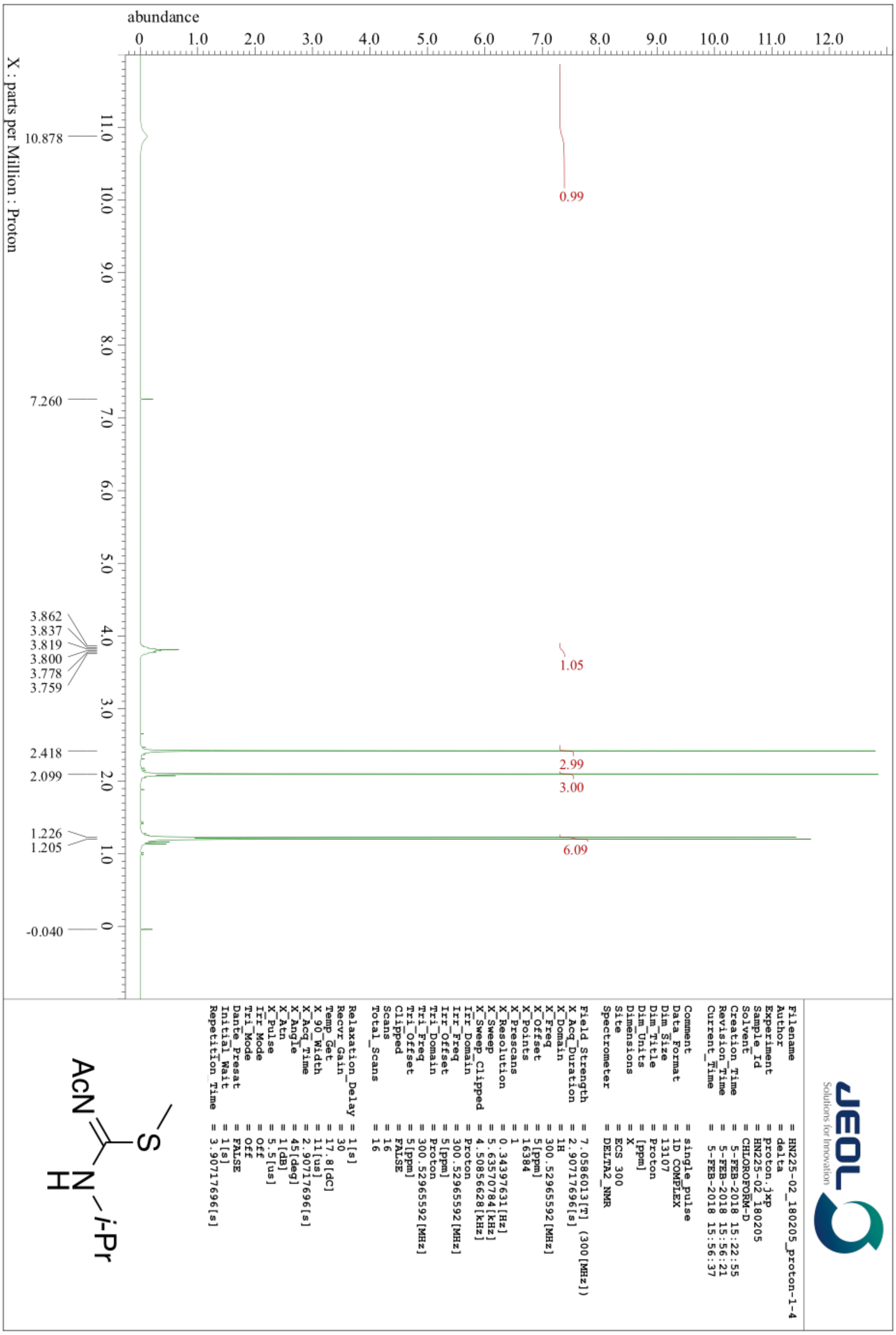

Compound **5** (^{13}C NMR, $CDCl_3$, 76 MHz)

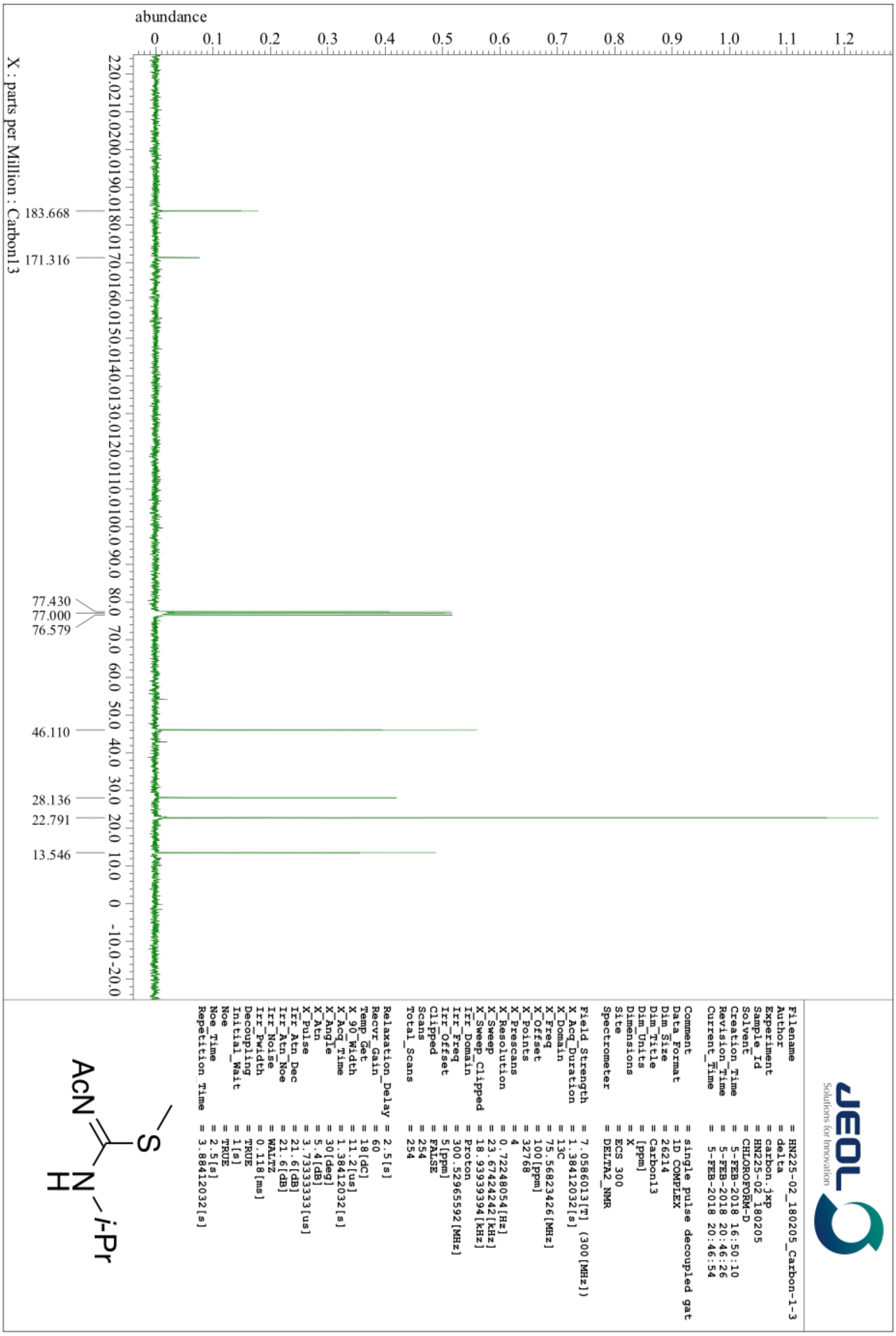

Compound **6** (^{1}H NMR, $CDCl_3$, 301 MHz)

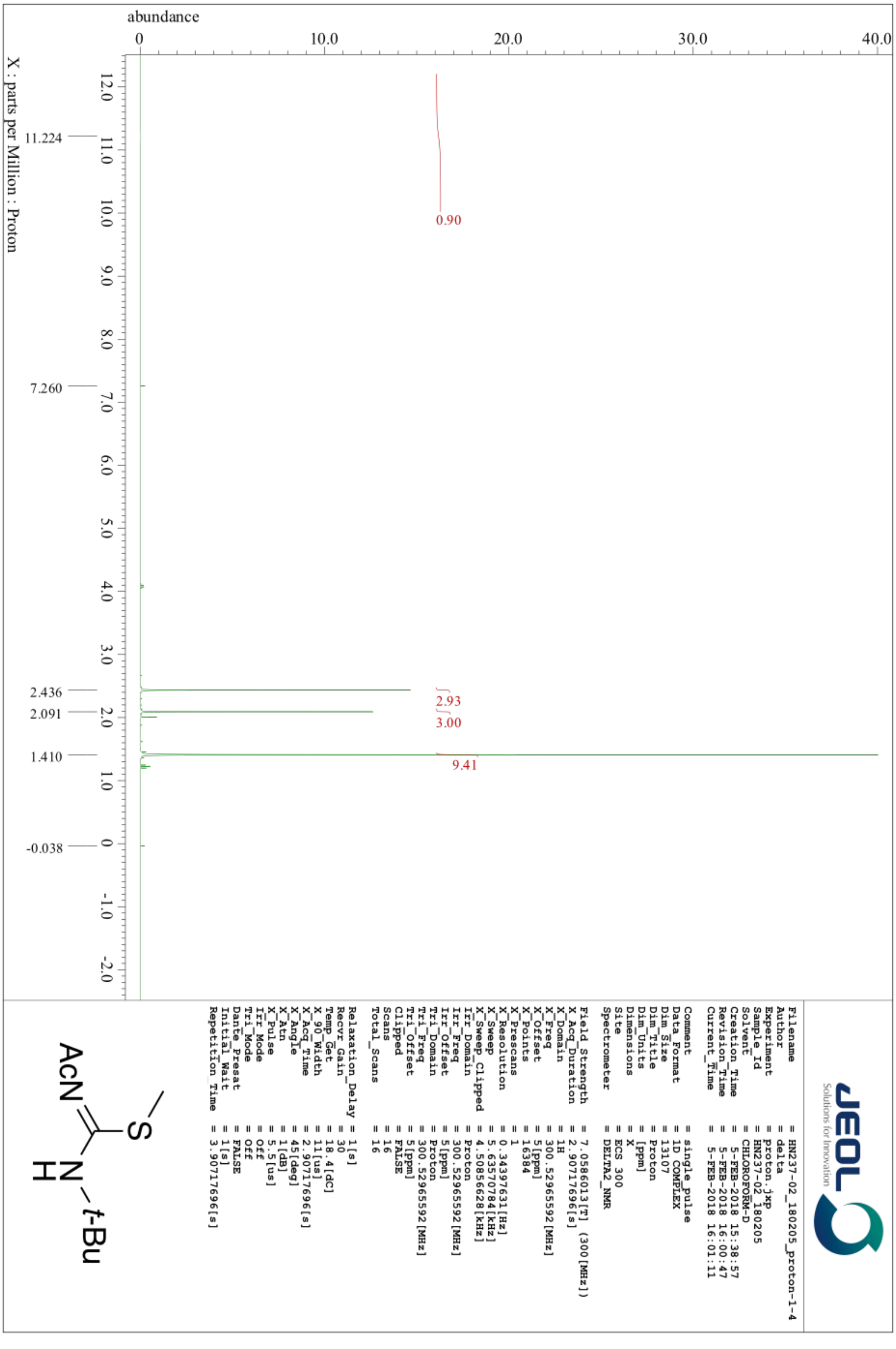

Compound **6** (^{13}C NMR, $CDCl_3$, 76 MHz)

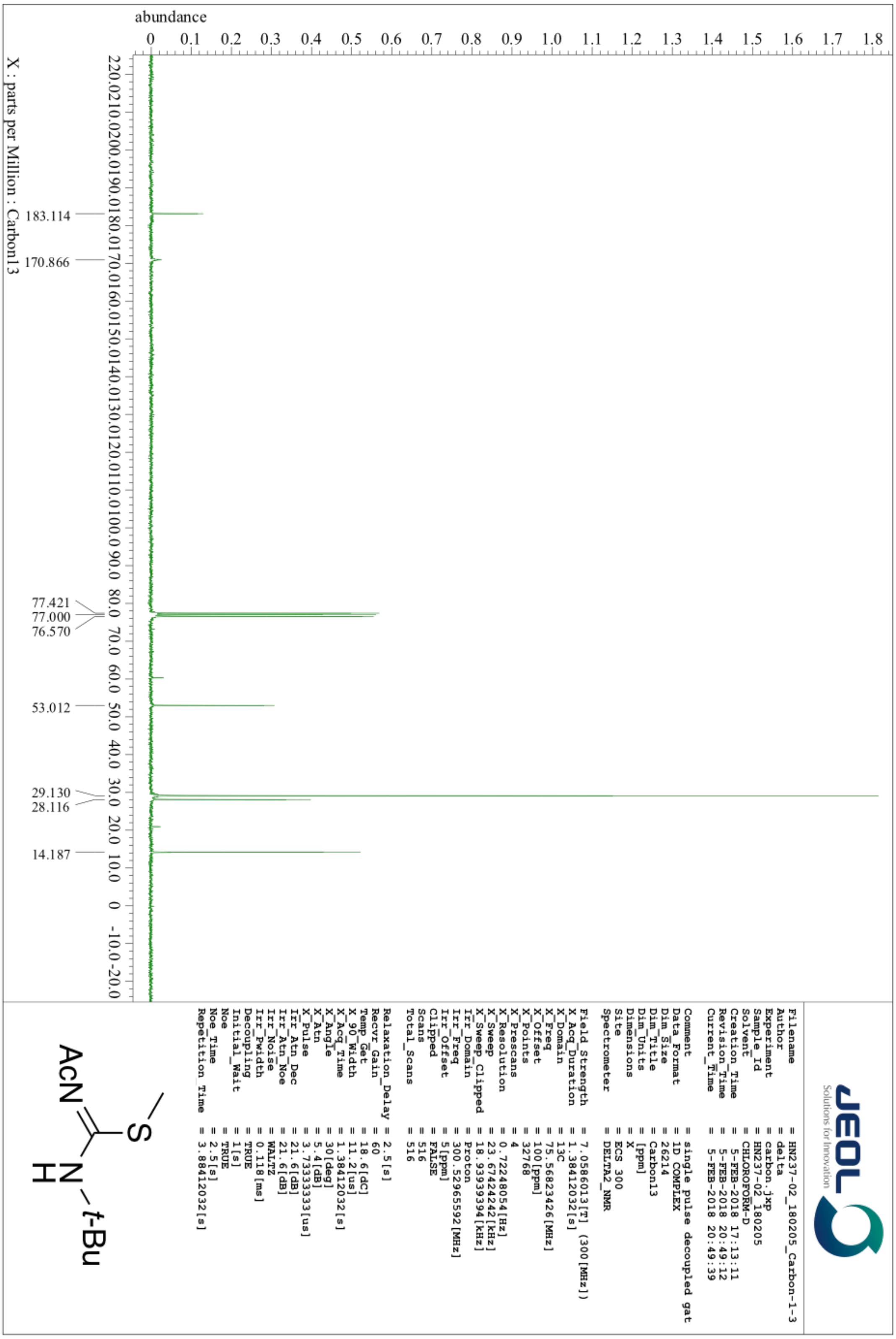

Compound **10-[Me,Me]** (^{1}H NMR, DMSO-d_6, 500 MHz)

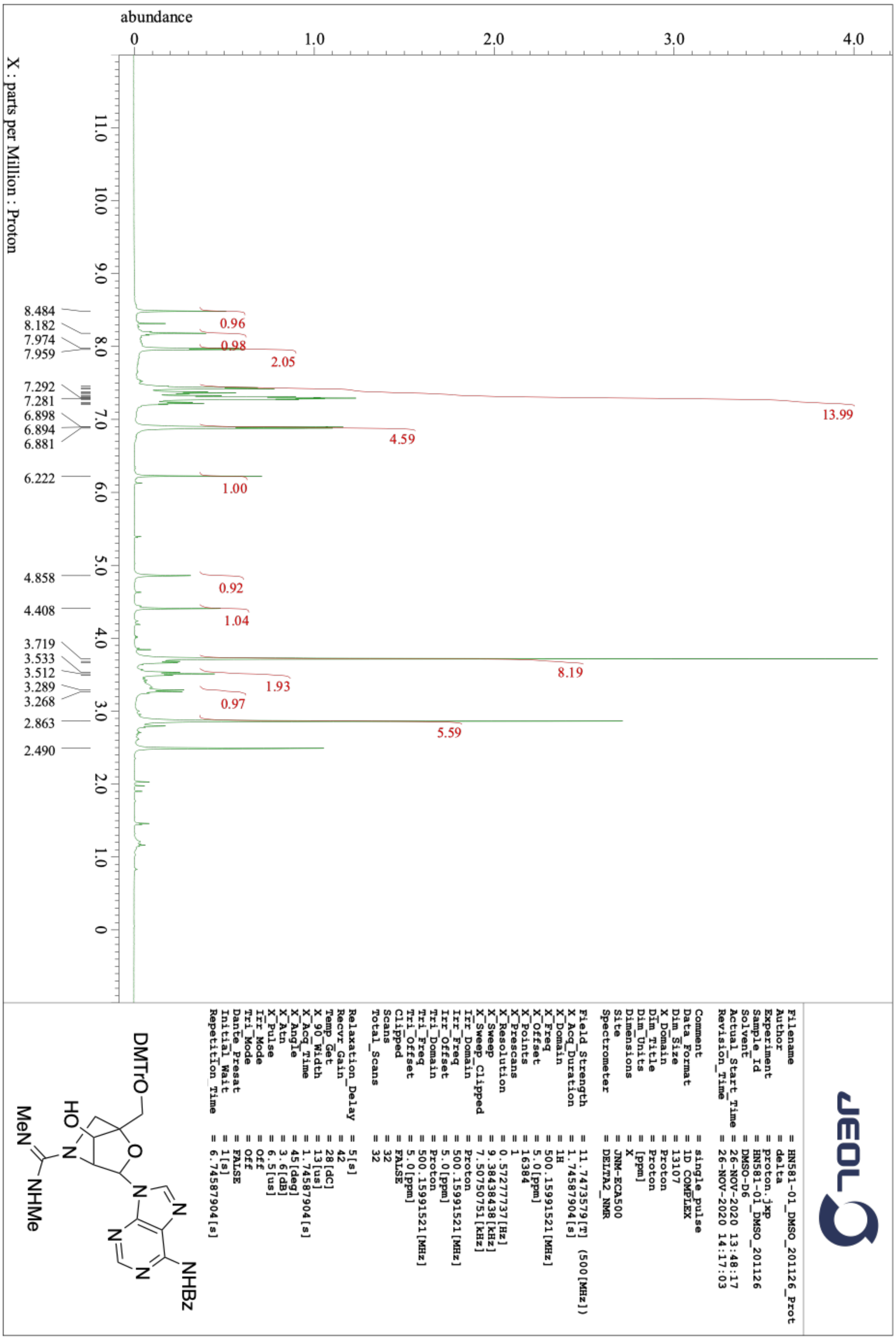

Compound **10-[Me,Me]** (^{13}C NMR, DMSO-d_6, 126 MHz)

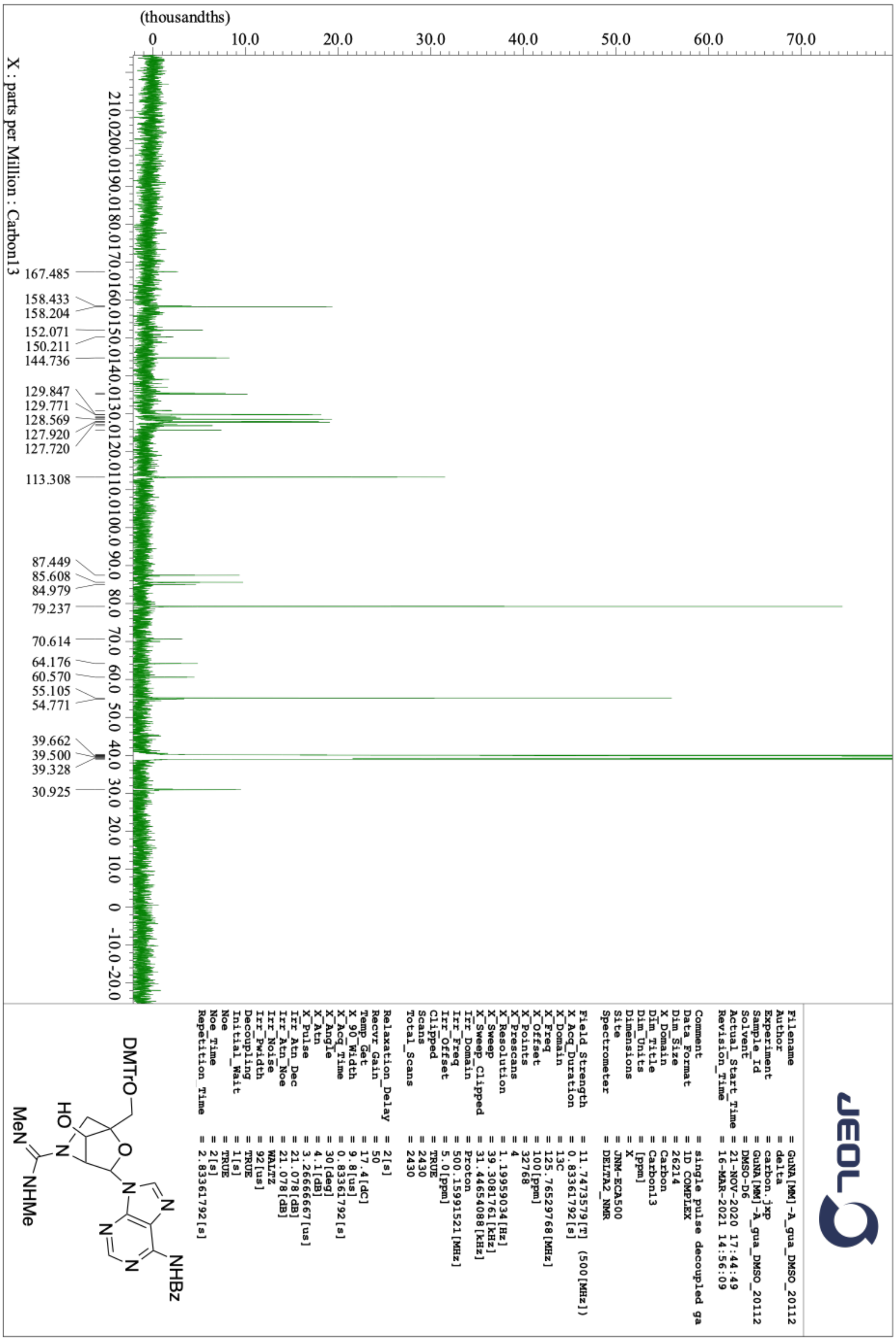

Compound **10-[Me,^{t}Bu]** (^{1}H NMR, $CDCl_3$, 301 MHz)

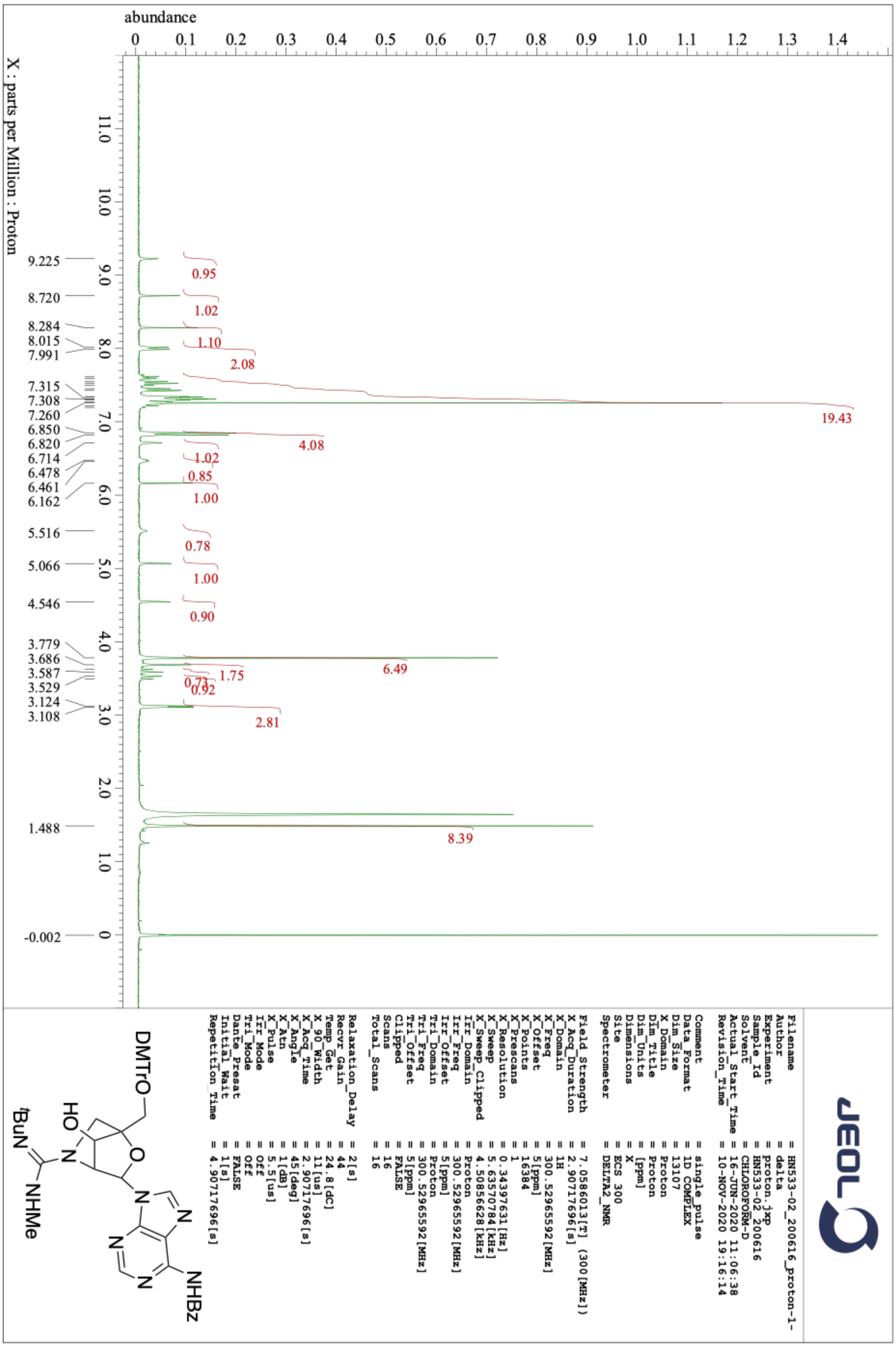

Compound **10-[Me,^{t}Bu]** (^{13}C NMR, $CDCl_3$, 101 MHz)

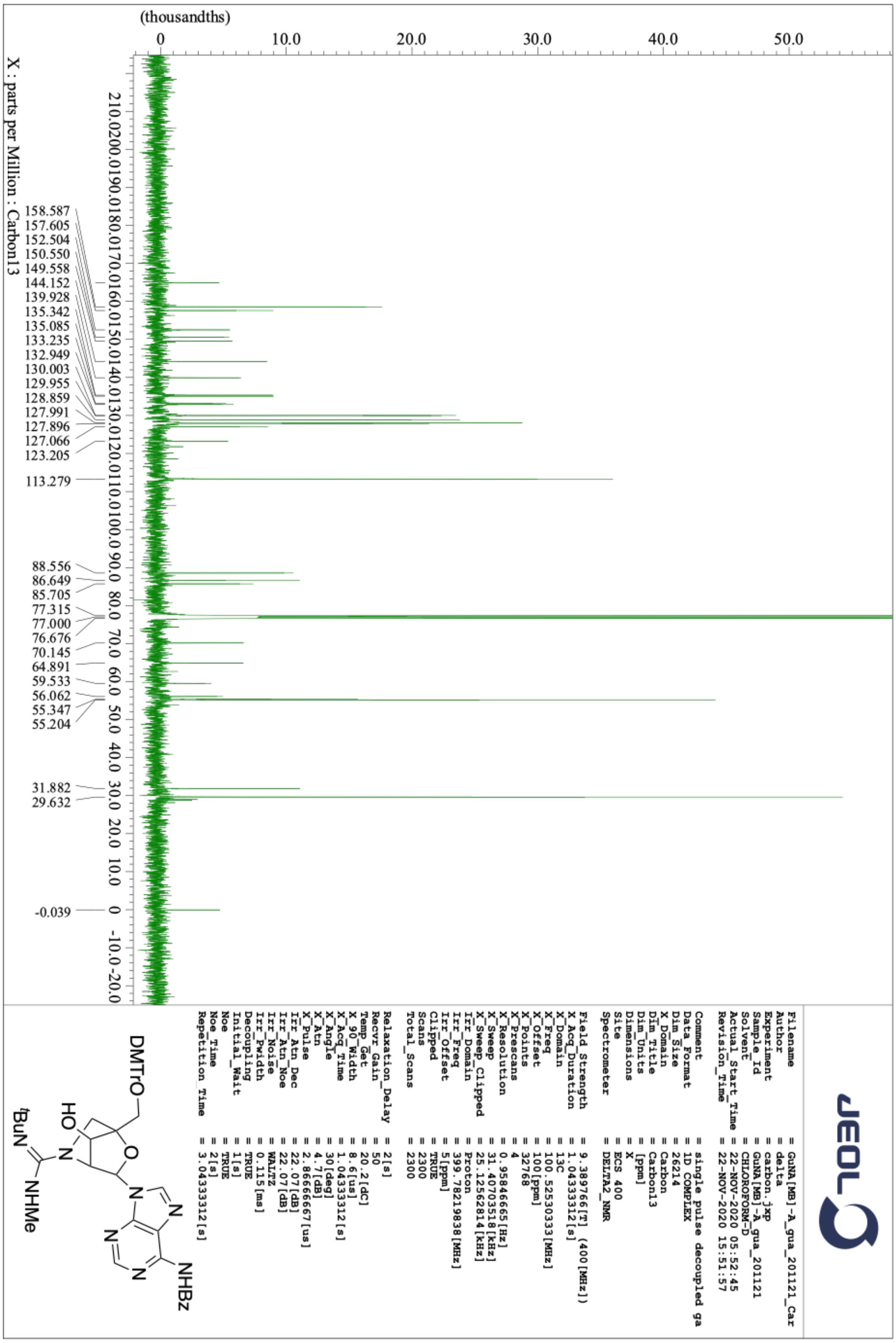

Compound **11-[Me,Me]** (^{1}H NMR, $CDCl_3$, 400 MHz)

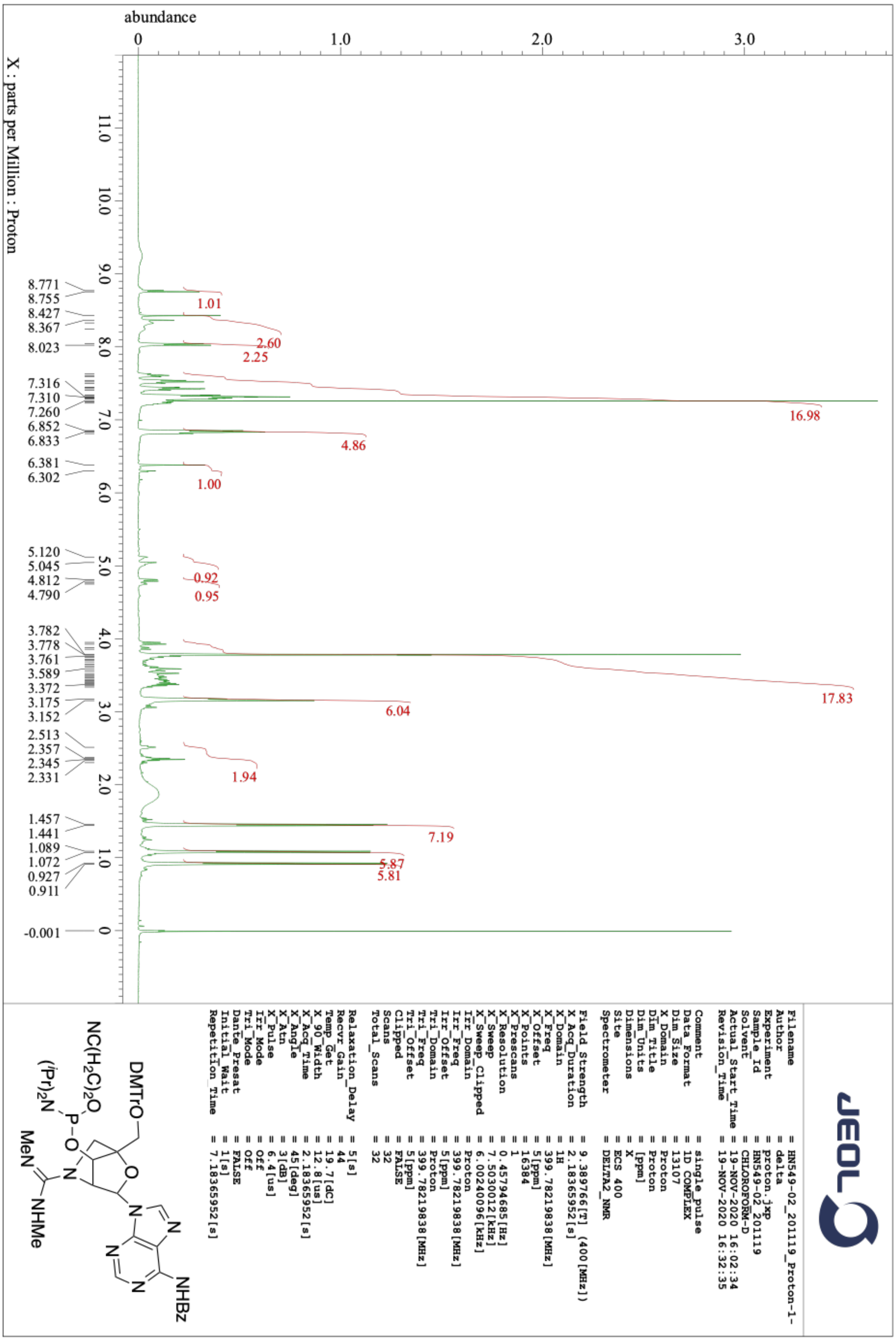

Compound **11-[Me,Me]** (^{31}P NMR, $CDCl_3$, 122 MHz)

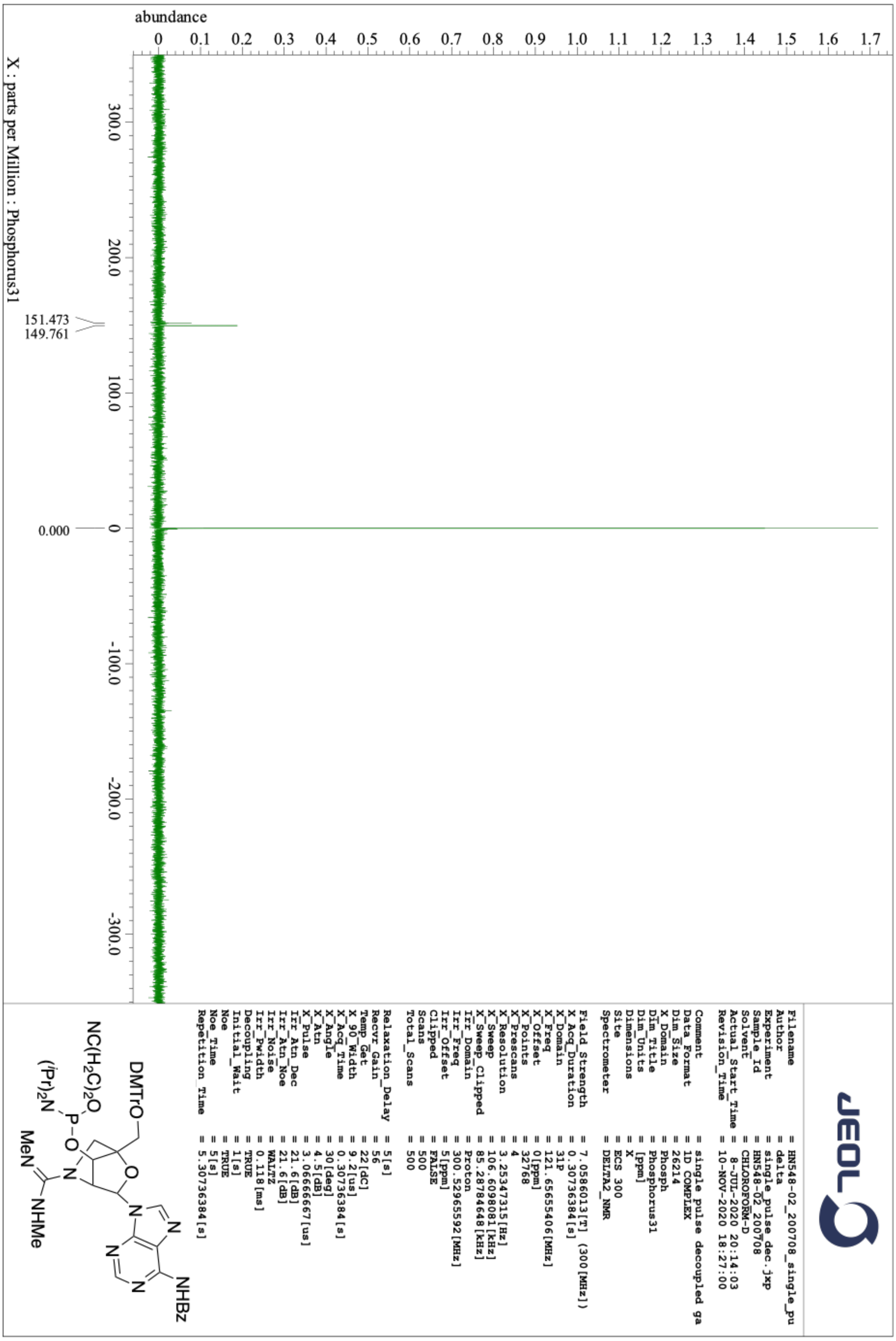

Compound **11-[Me,^{t}Bu]** (^{1}H NMR, $CDCl_3$, 400 MHz)

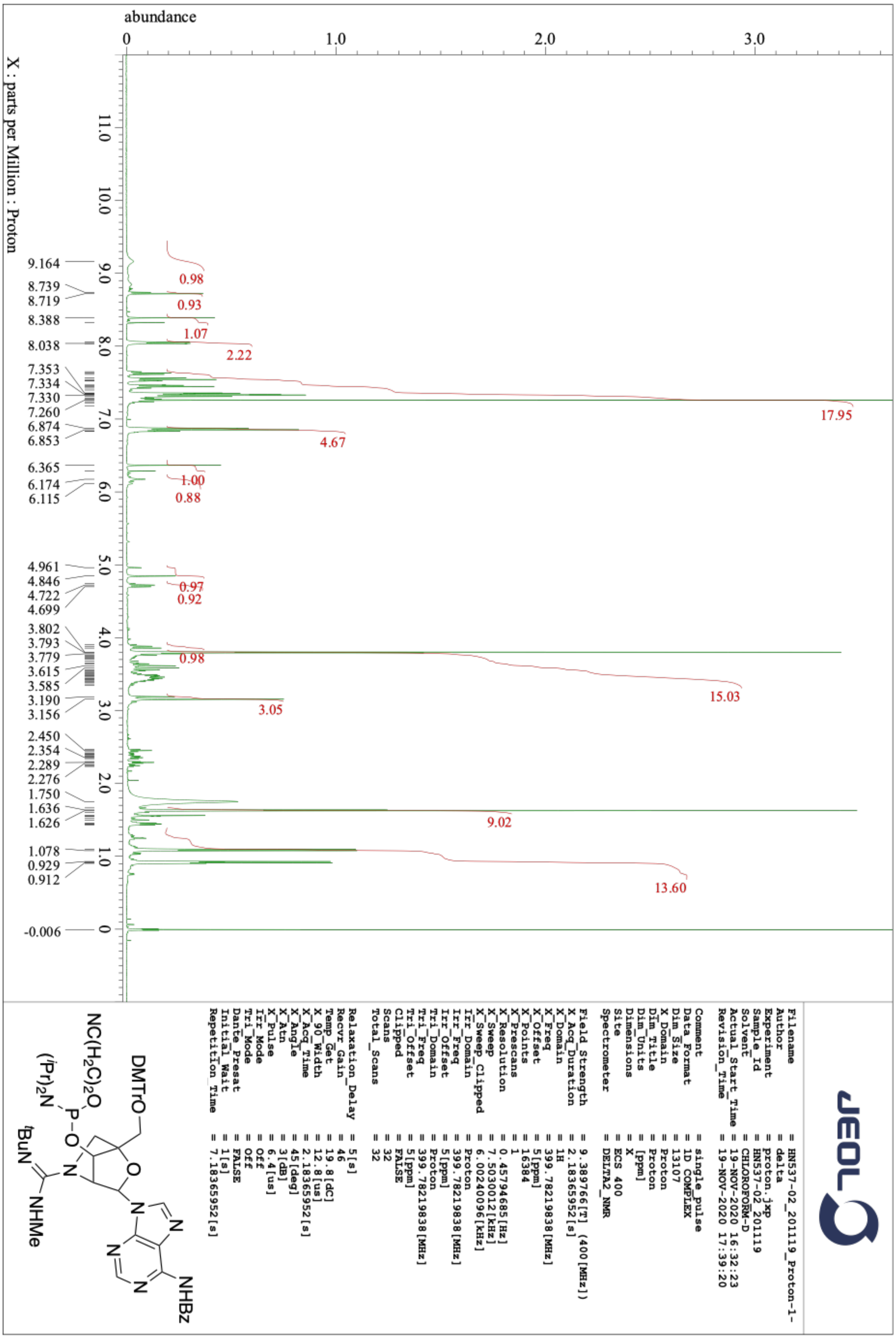

Compound **11-[Me,^{t}Bu]** (^{31}P NMR, $CDCl_3$, 162 MHz)

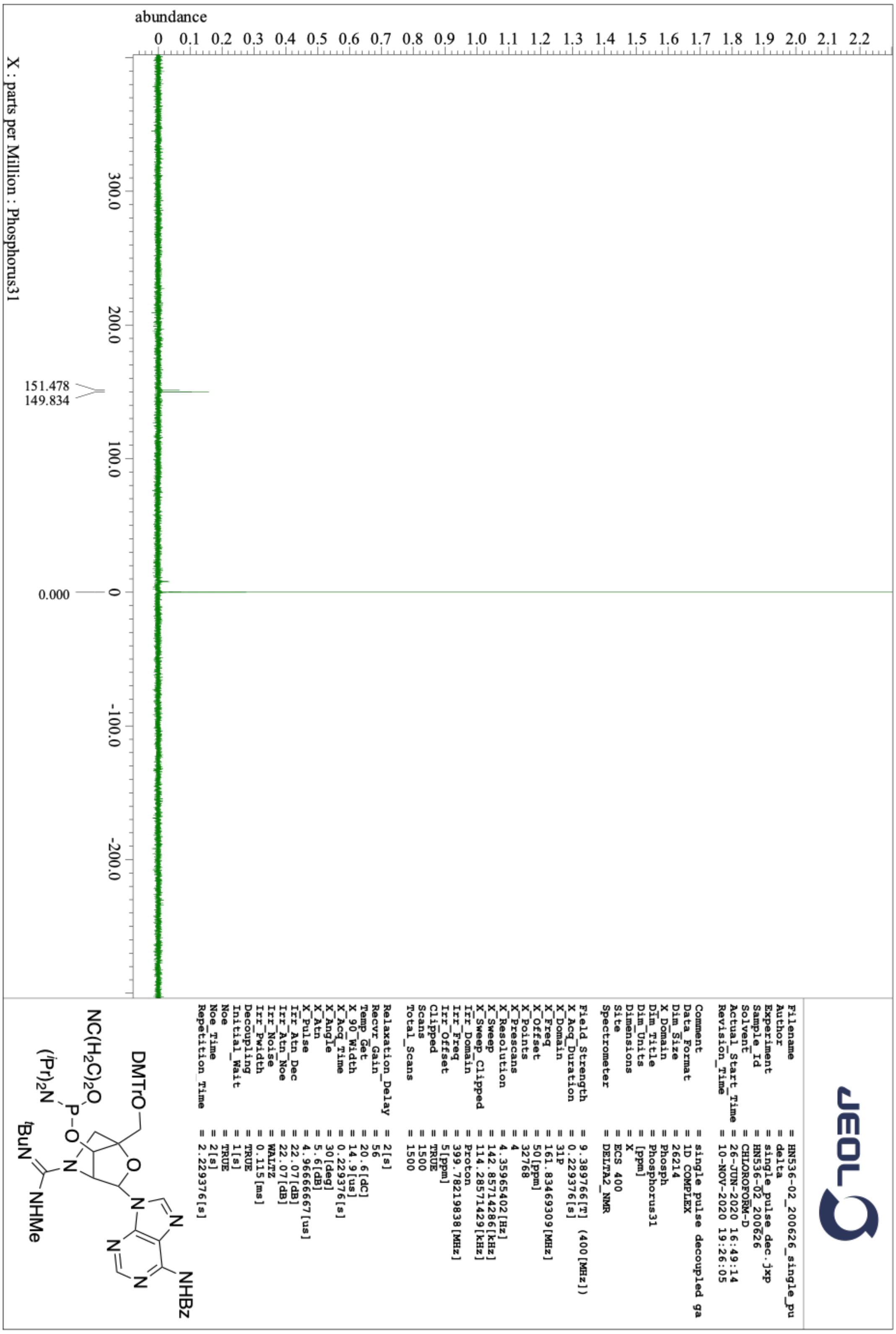

Compound **13-[Me,Me]** (^{1}H NMR, $CDCl_3$, 301 MHz)

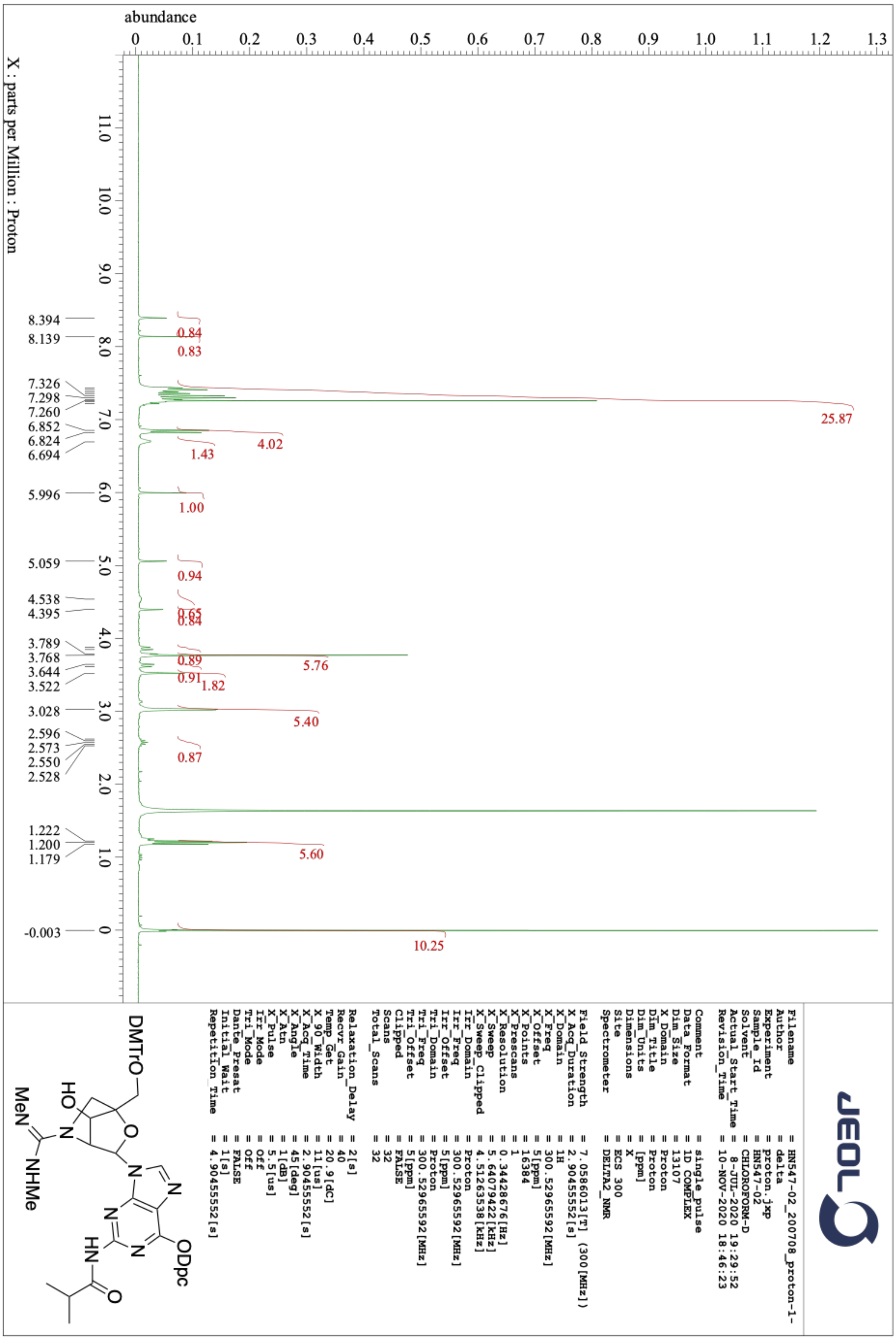

Compound **13-[Me,Me]** (^{13}C NMR, $CDCl_3$, 101 MHz)

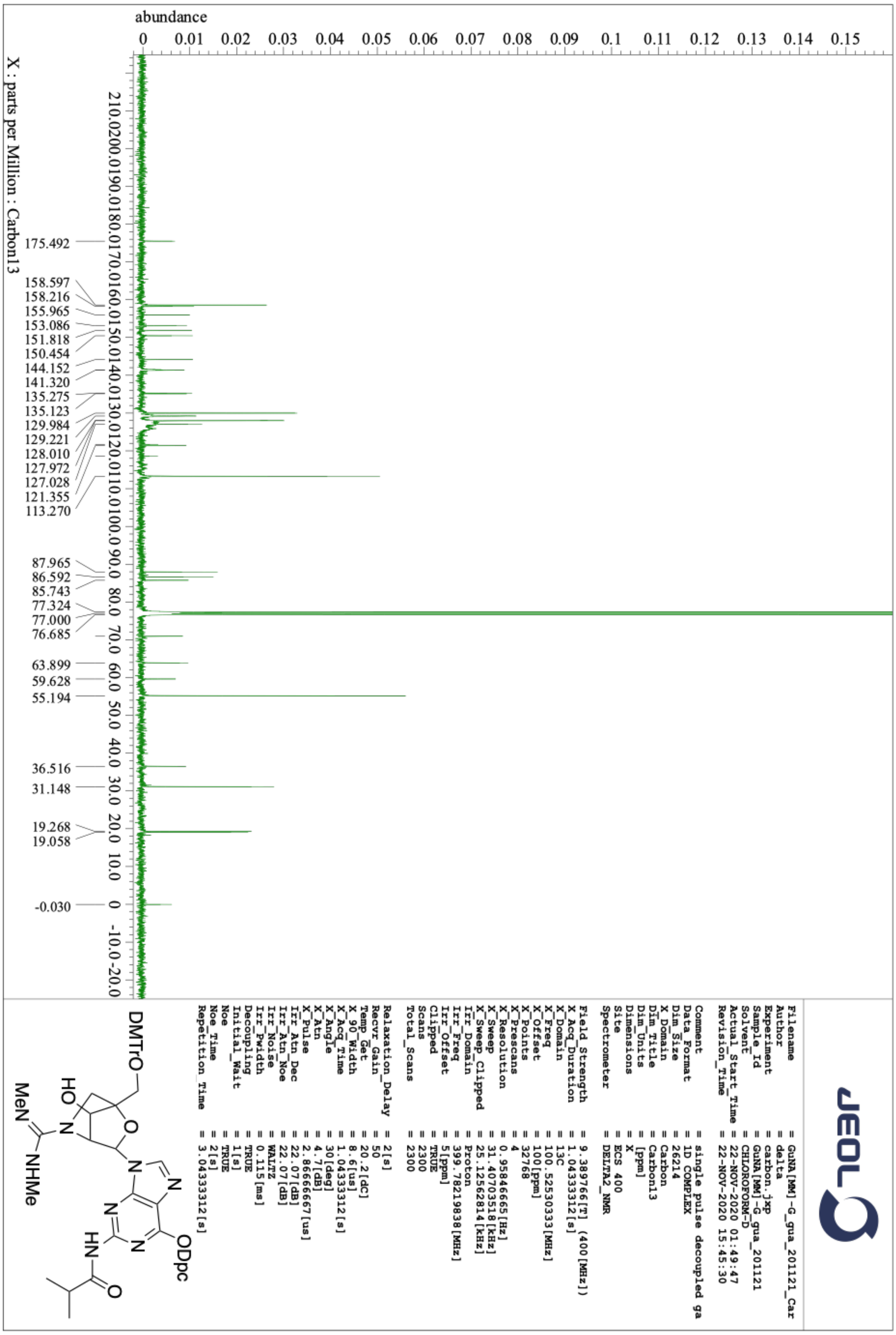

Compound **13-[Me,^{t}Bu]** (^{1}H NMR, $CDCl_3$, 301 MHz)

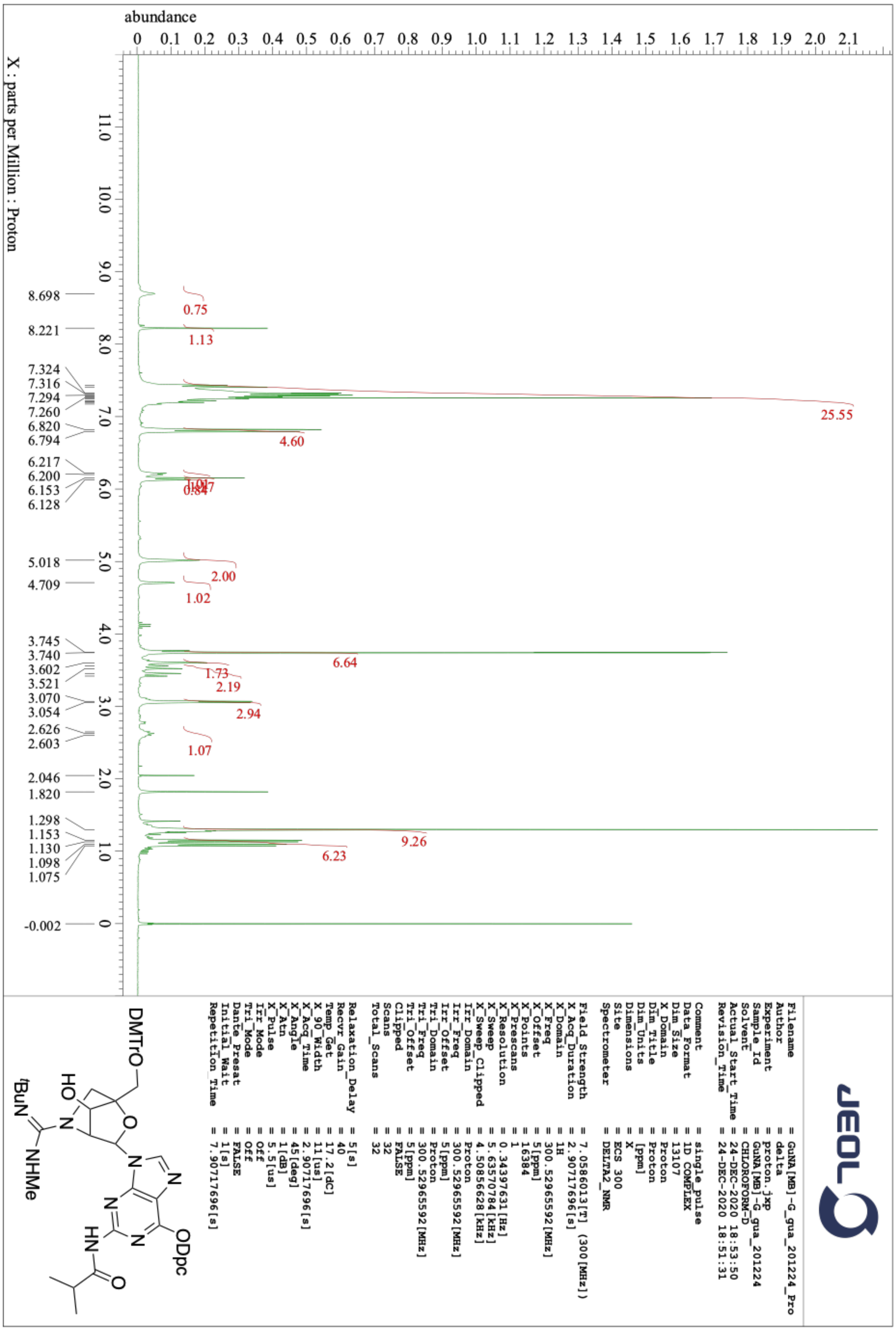

Compound **13-[Me,^{t}Bu]** (^{13}C NMR, $CDCl_3$, 101 MHz)

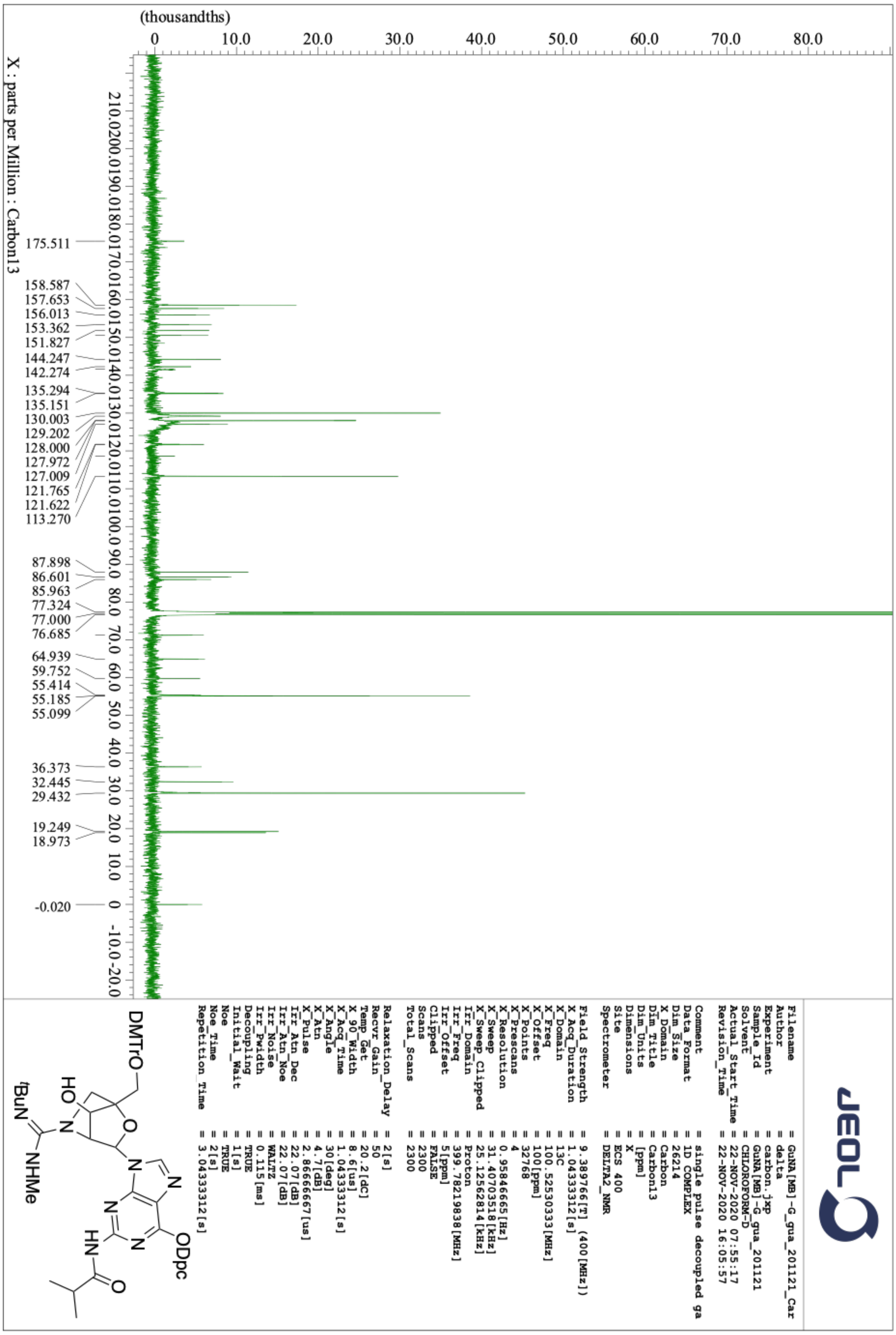

Compound **14-[Me,Me]** (^{1}H NMR, $CDCl_3$, 500 MHz)

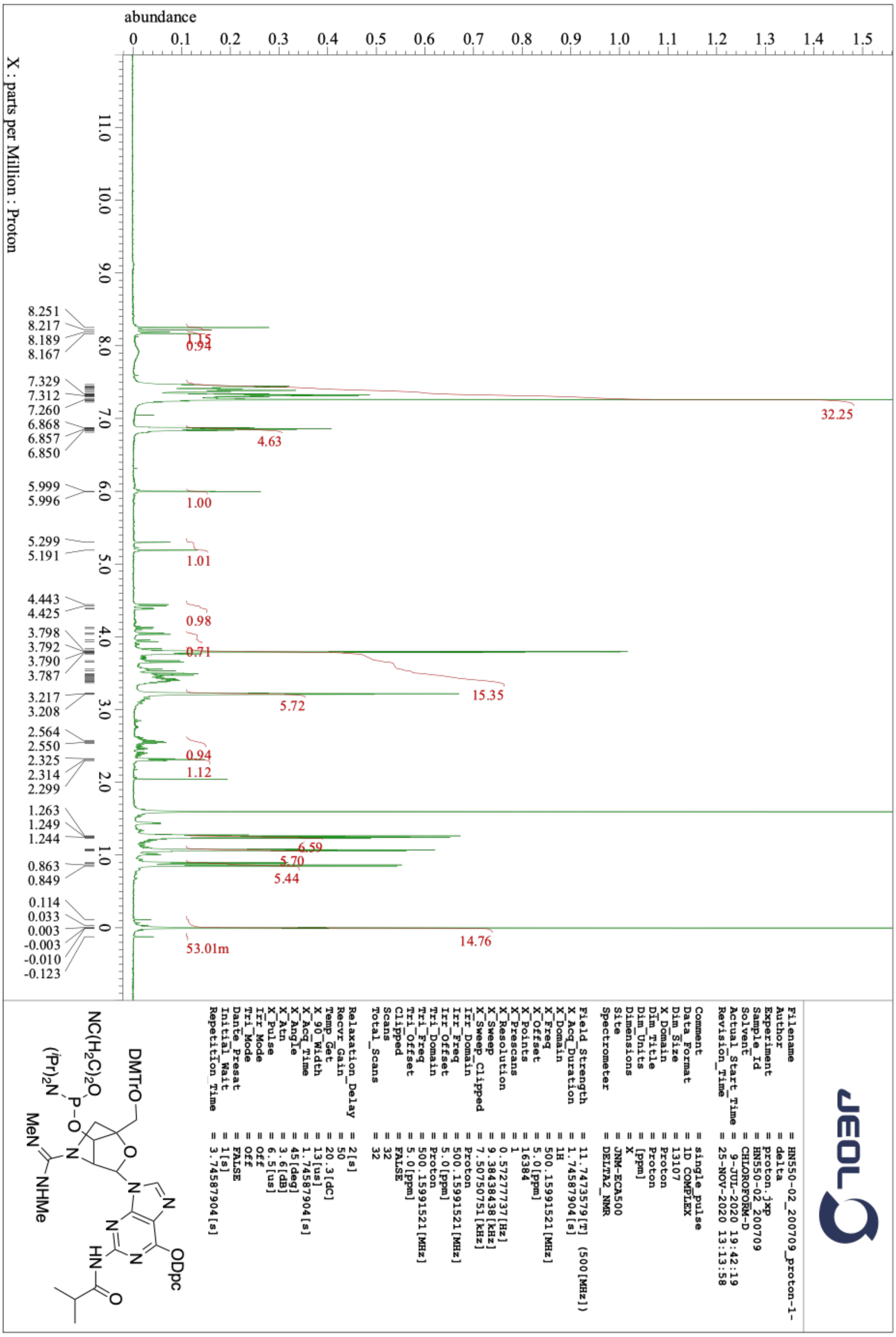

Compound **14-[Me,Me]** (^{31}P NMR, $CDCl_3$, 202 MHz)

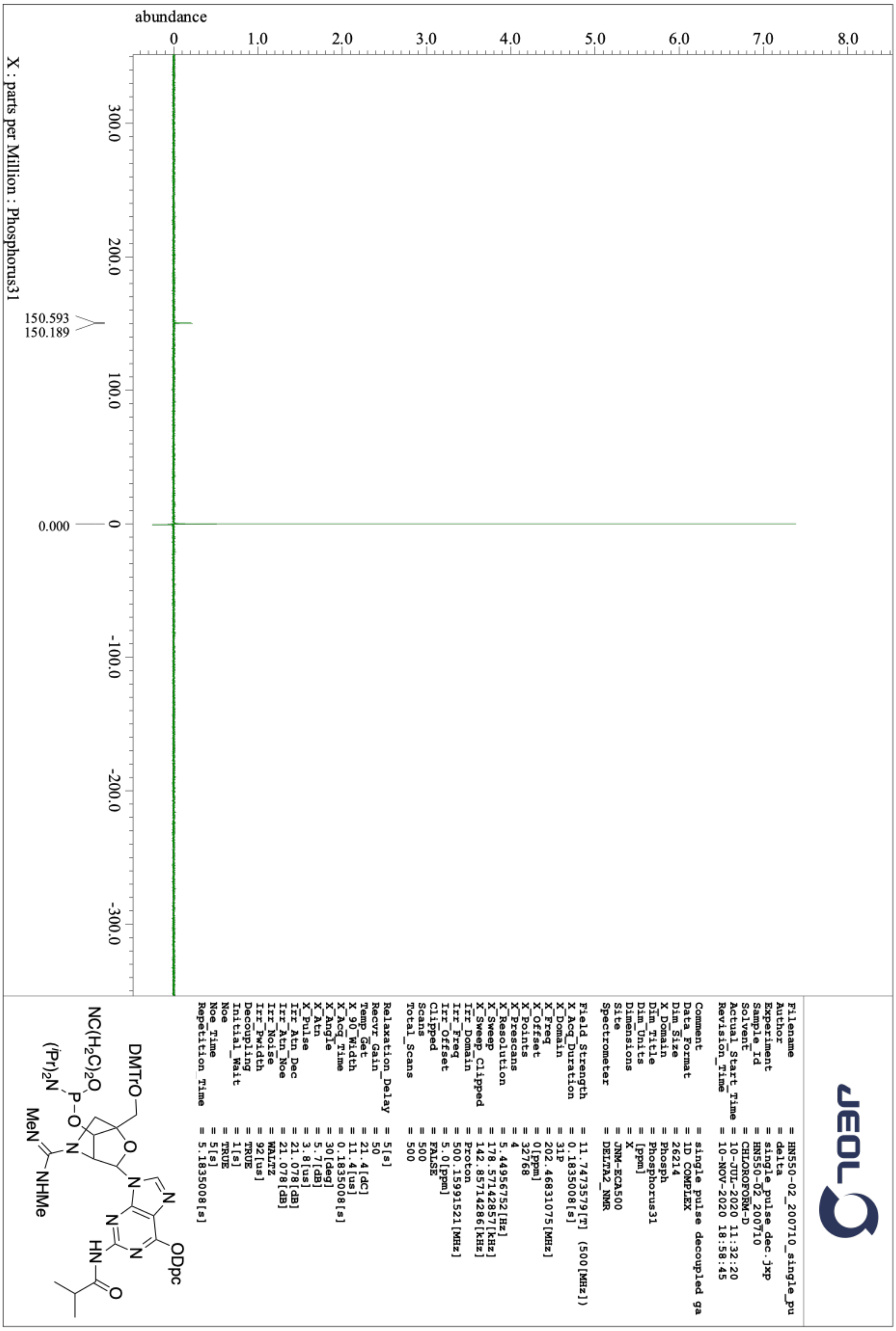

Compound **14-[Me,^{t}Bu]** (^{1}H NMR, $CDCl_3$, 400 MHz)

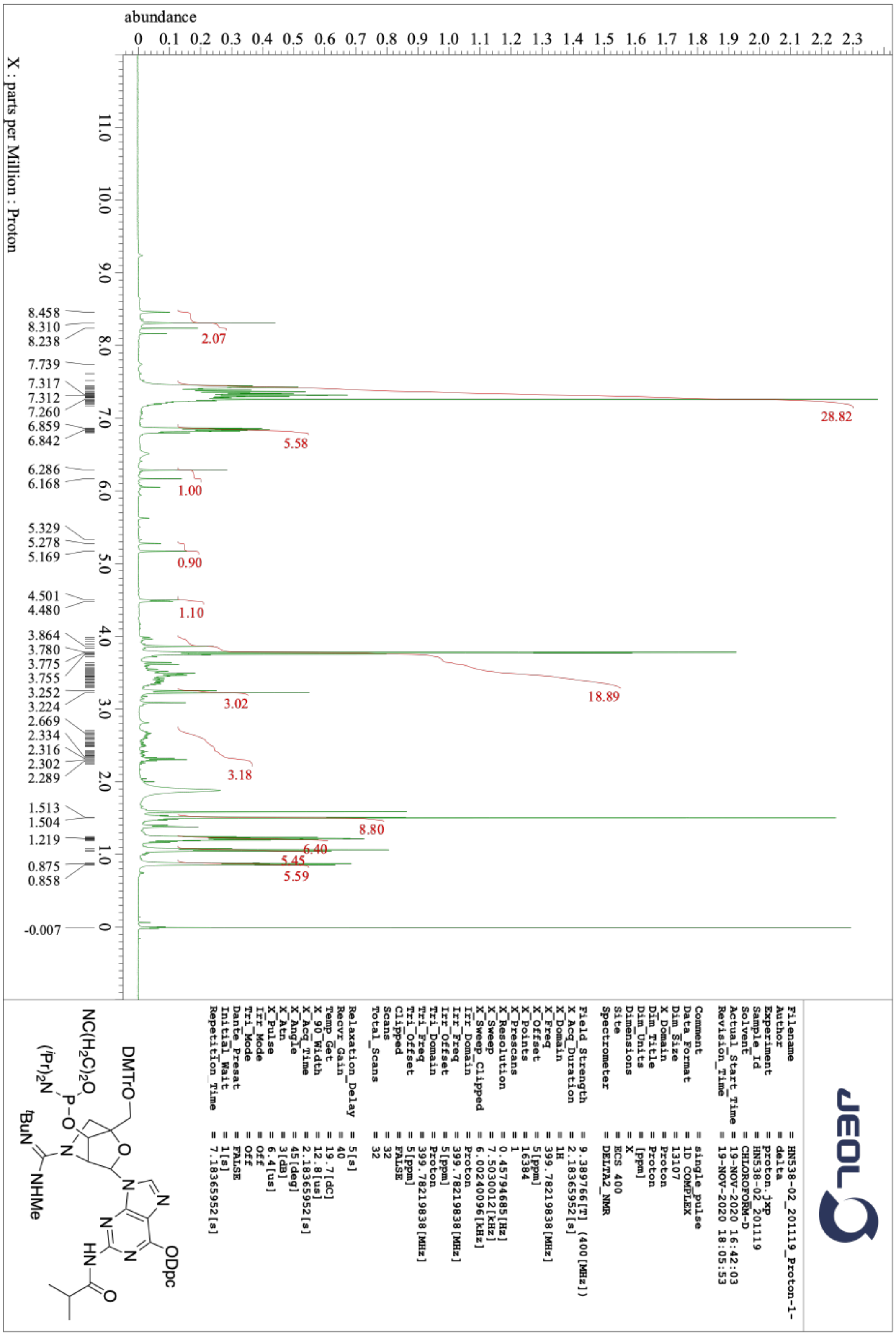

Compound **14-[Me,^{t}Bu]** (^{31}P NMR, $CDCl_3$, 162 MHz)

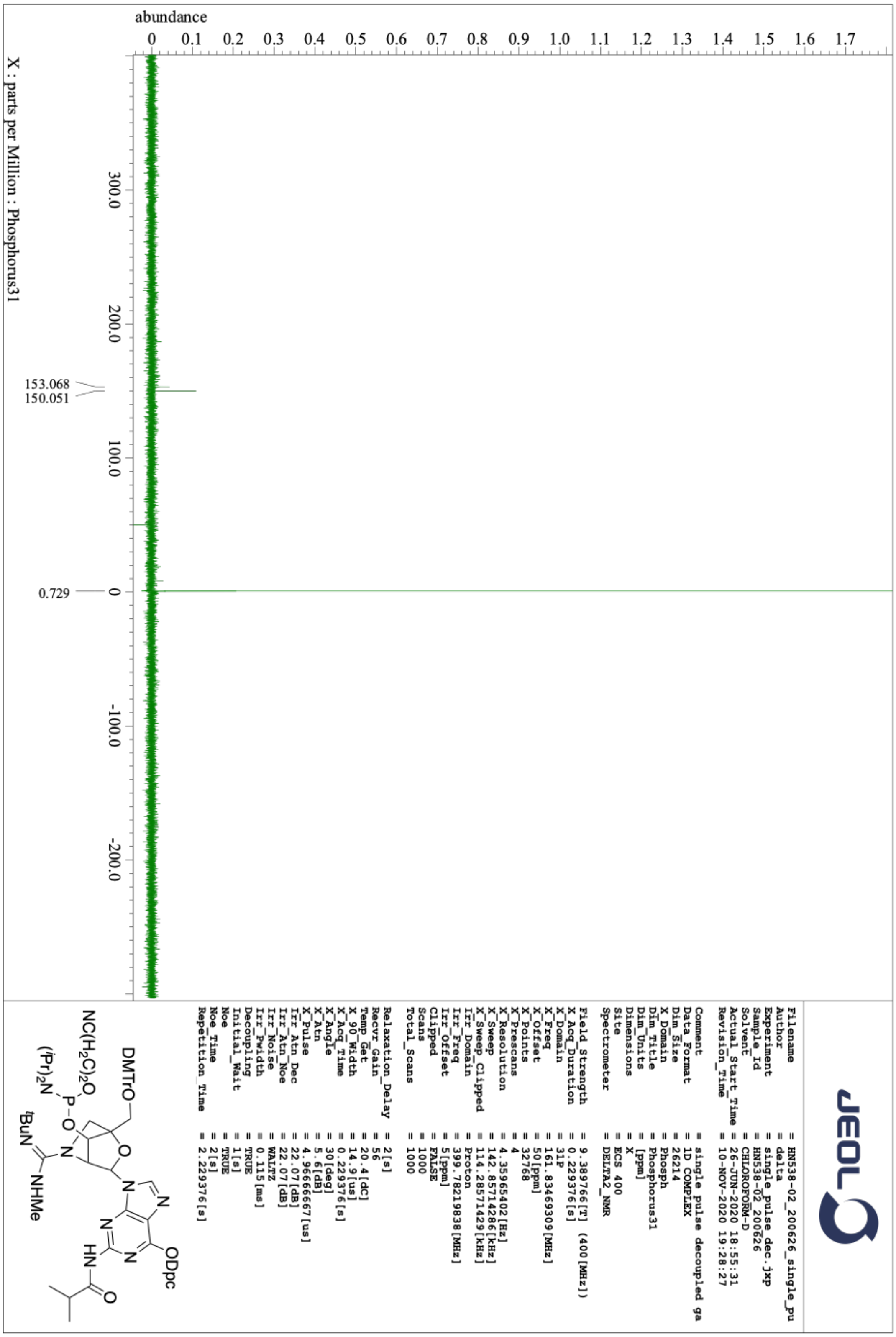

Compound **16-[Me,Me]** (^{1}H NMR, $CDCl_3$, 301 MHz)

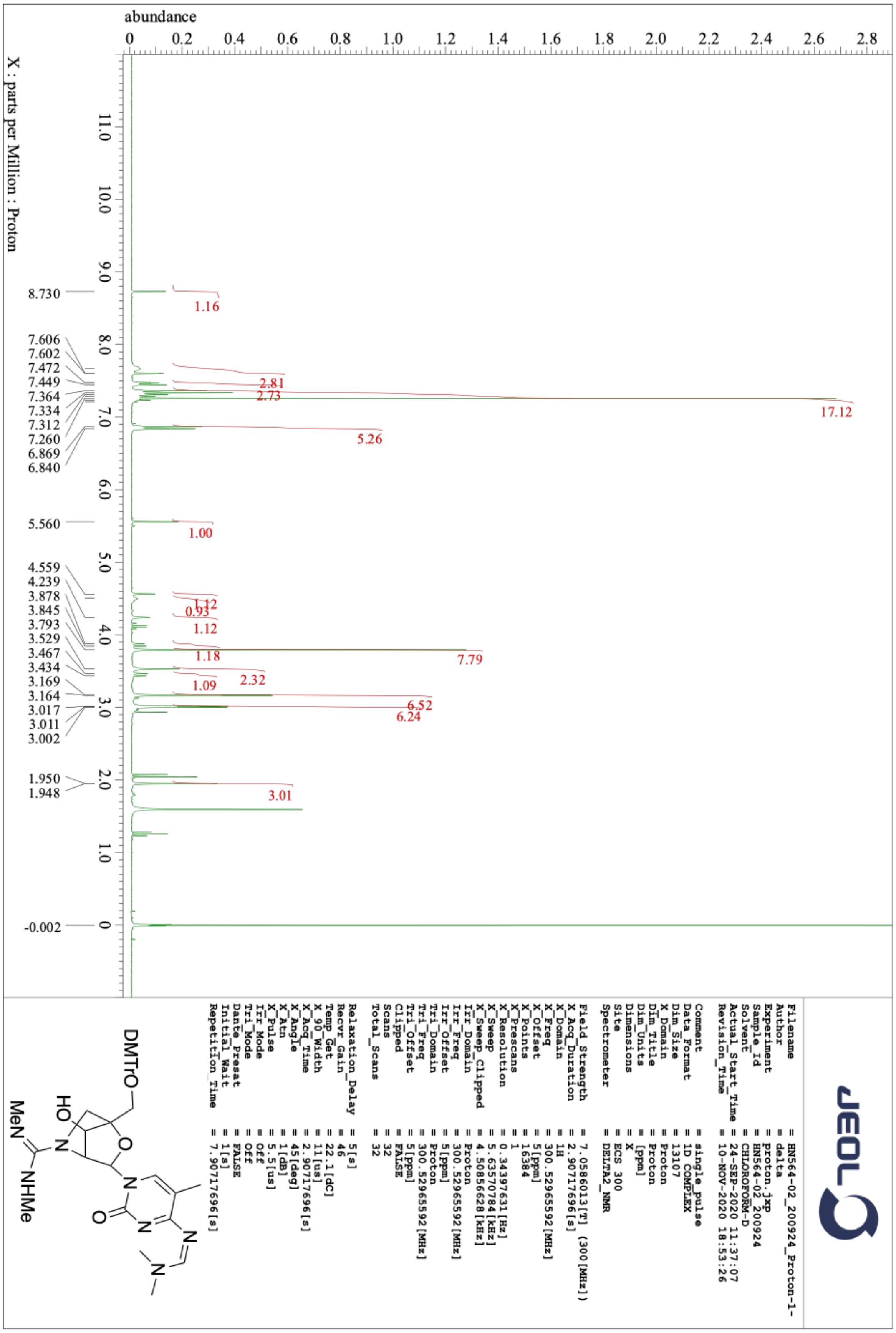

Compound **16-[Me,Me]** (^{13}C NMR, $CDCl_3$, 101 MHz)

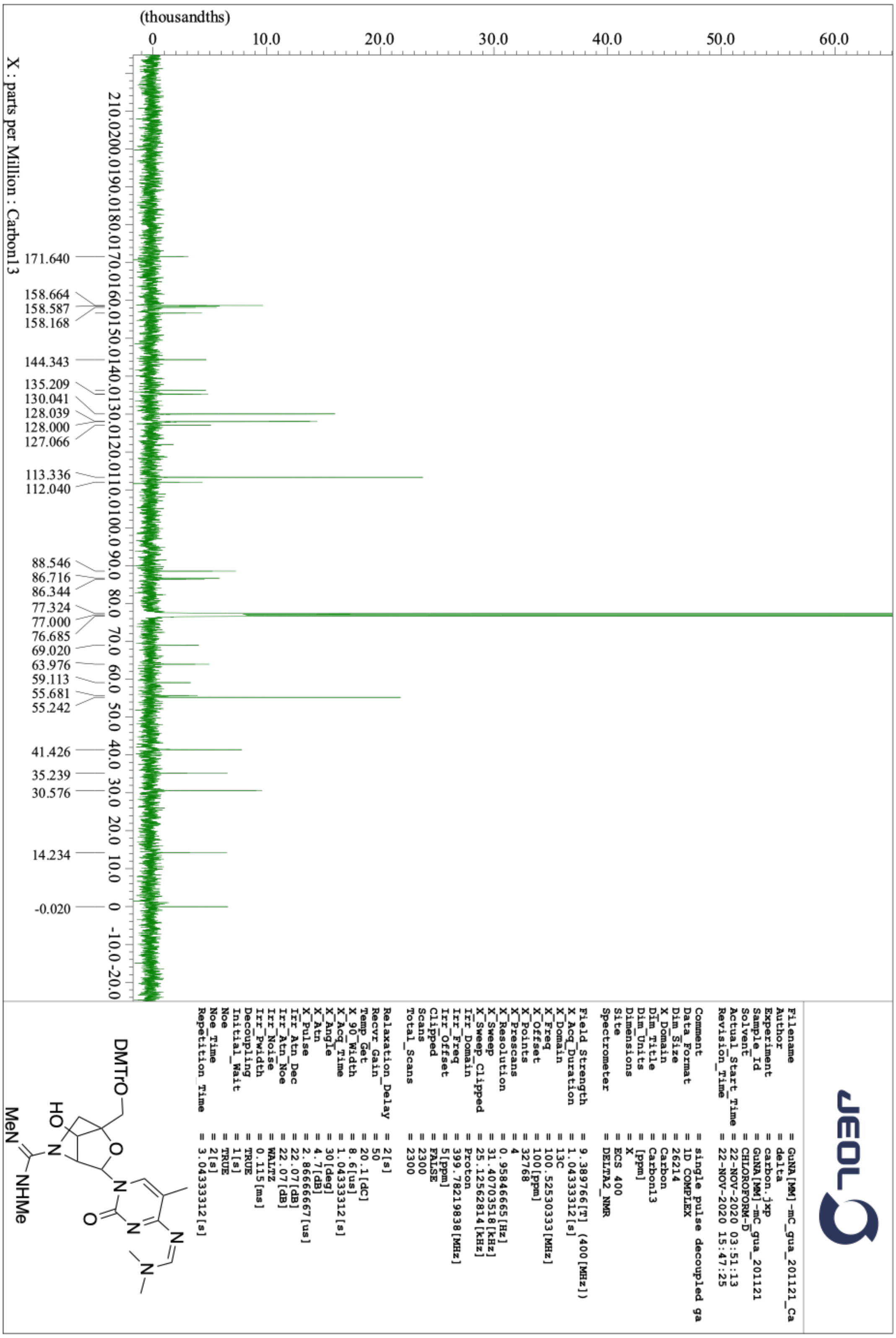

Compound **16-[Me,^{t}Bu]** (^{1}H NMR, $CDCl_3$, 500 MHz)

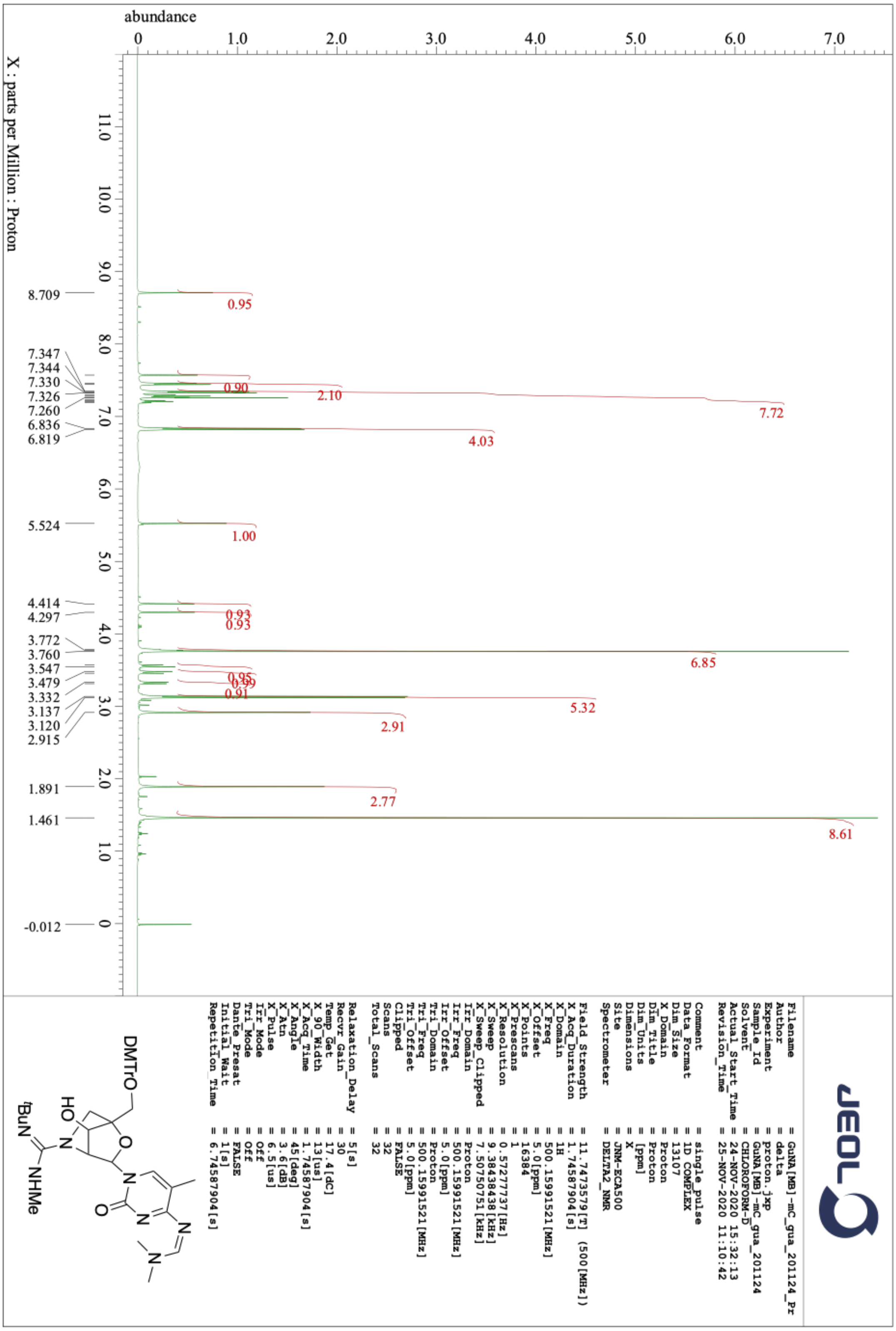

Compound **16-[Me,^{t}Bu]** (^{13}C NMR, $CDCl_3$, 101 MHz)

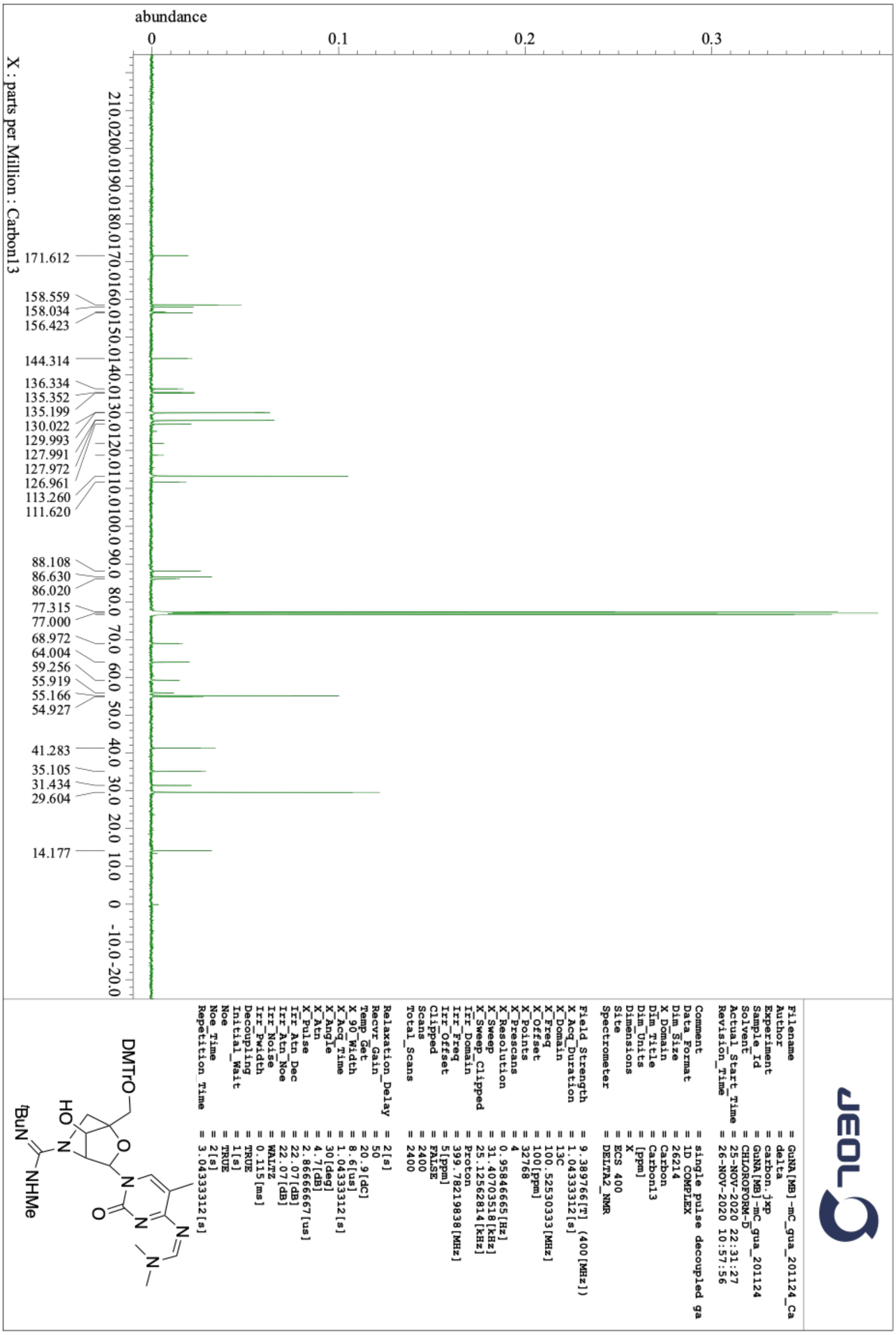

Compound **17-[Me,Me]** (^{1}H NMR, $CDCl_3$, 301 MHz)

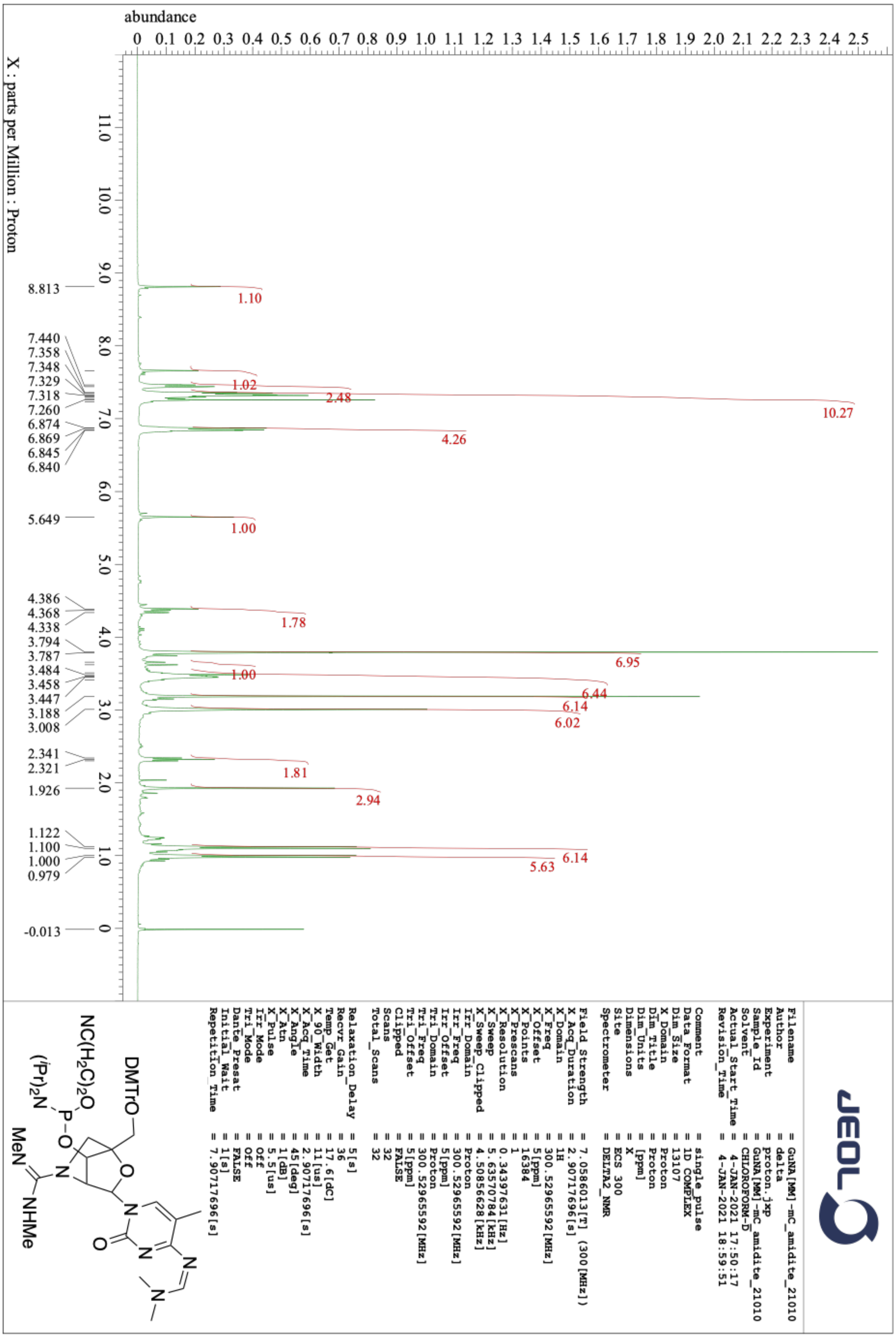

Compound **17-[Me,Me]** (^{31}P NMR, $CDCl_3$, 202 MHz)

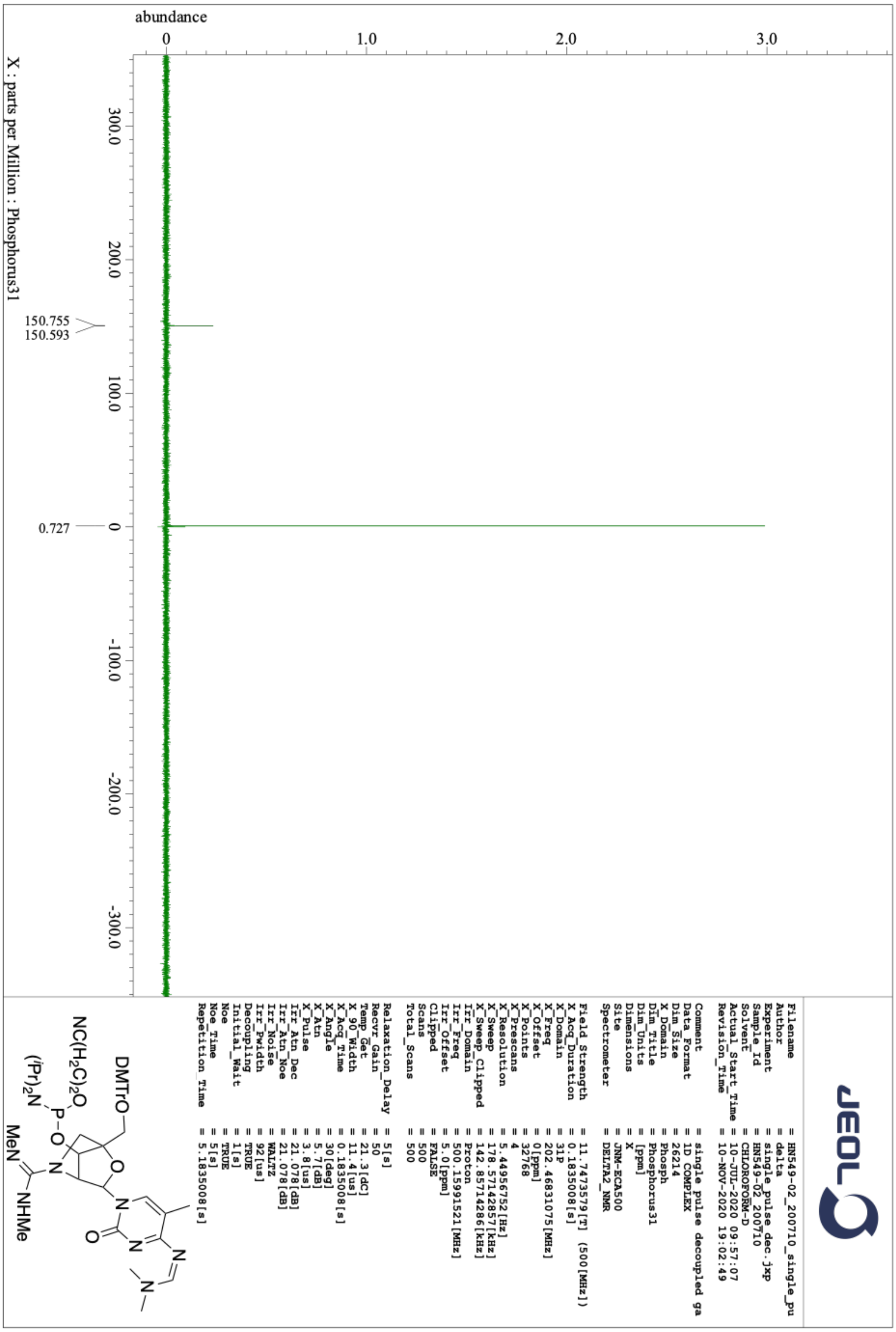

Compound **17-[Me,^{t}Bu]** (^{1}H NMR, $CDCl_3$, 500 MHz)

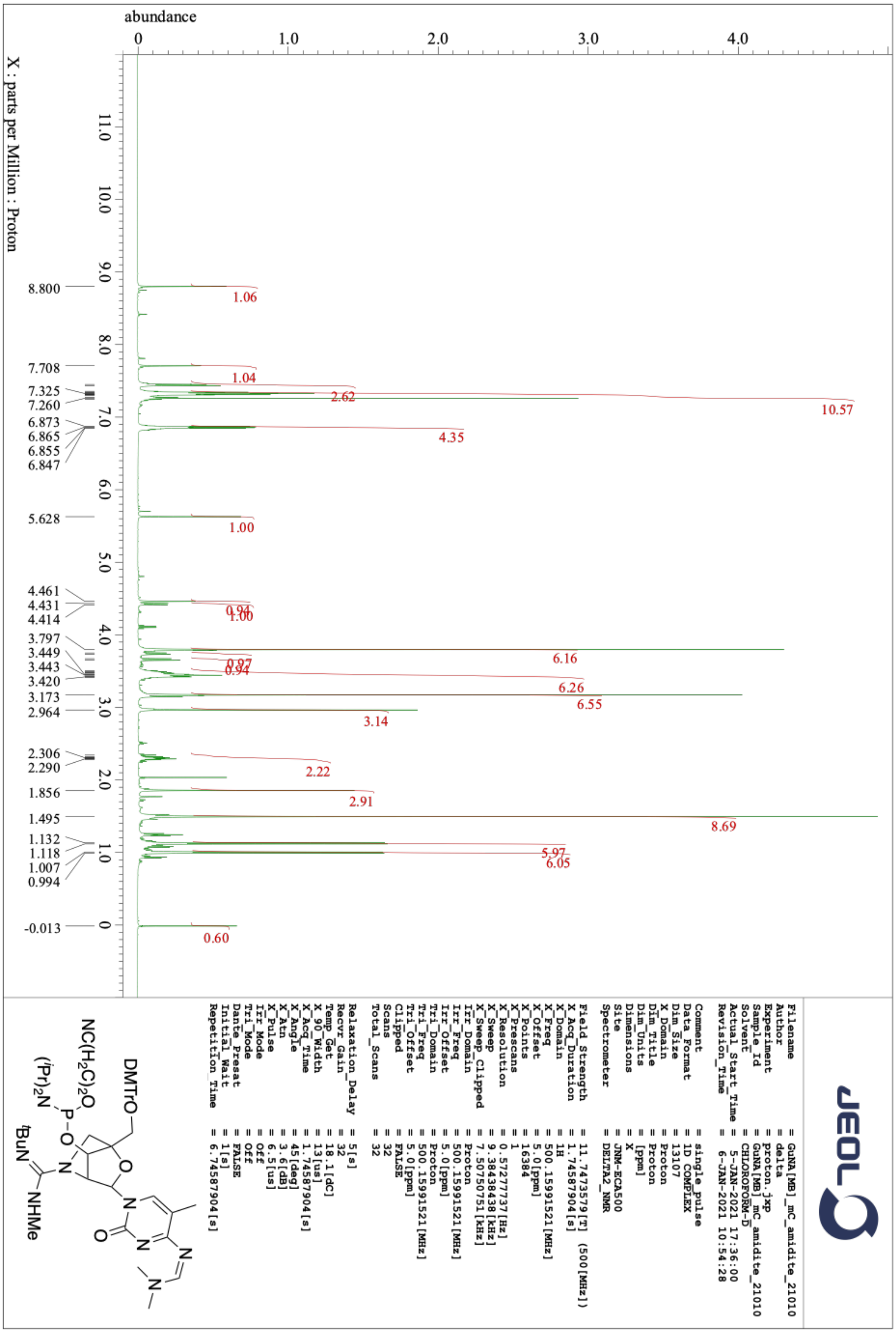

Compound **17-[Me,^{t}Bu]** (^{31}P NMR, $CDCl_3$, 162 MHz)

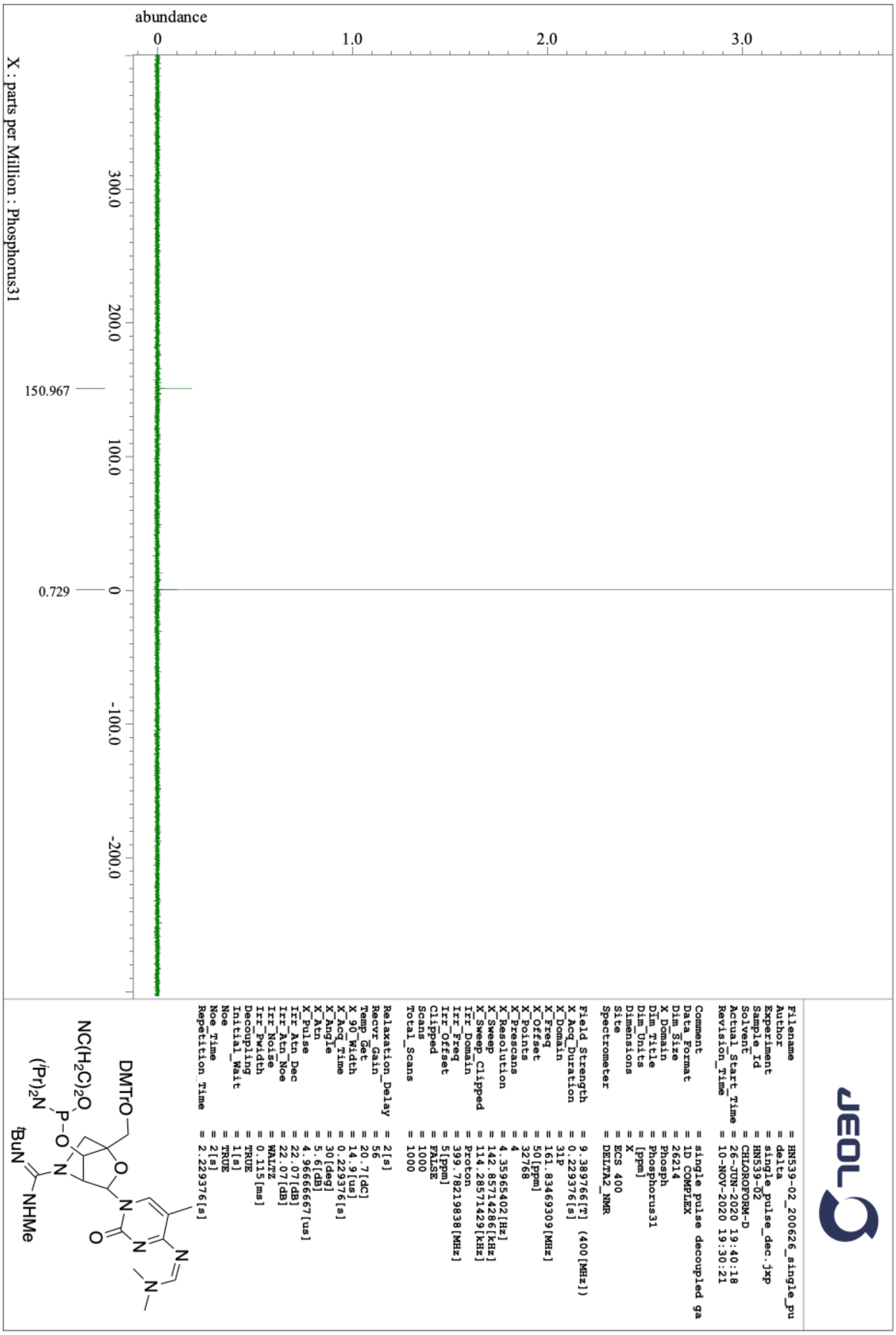

Compound **19** (^{1}H NMR, DMSO-d_6, 400 MHz)

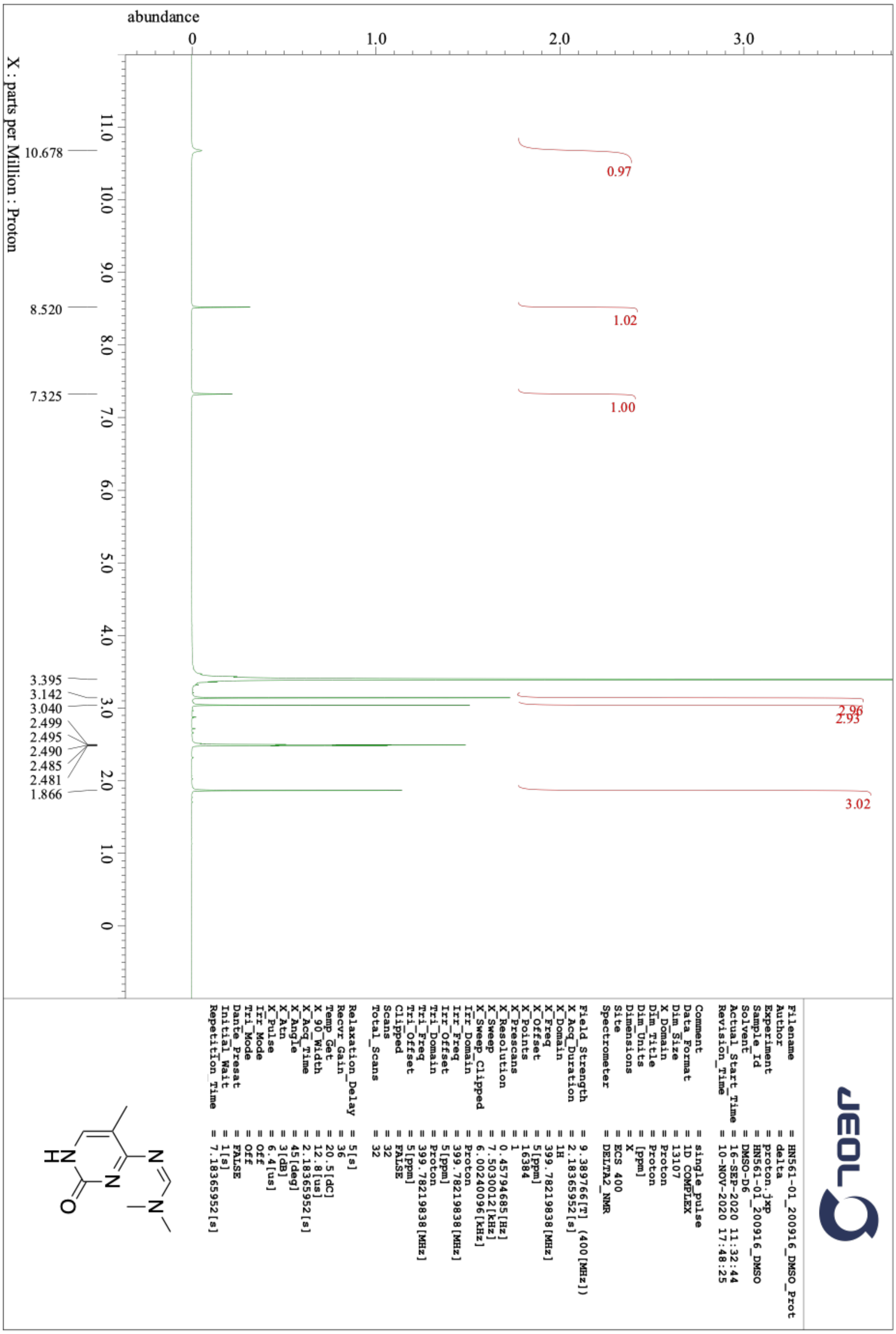

Compound **19** (^{13}C NMR, DMSO-d_6, 101 MHz)

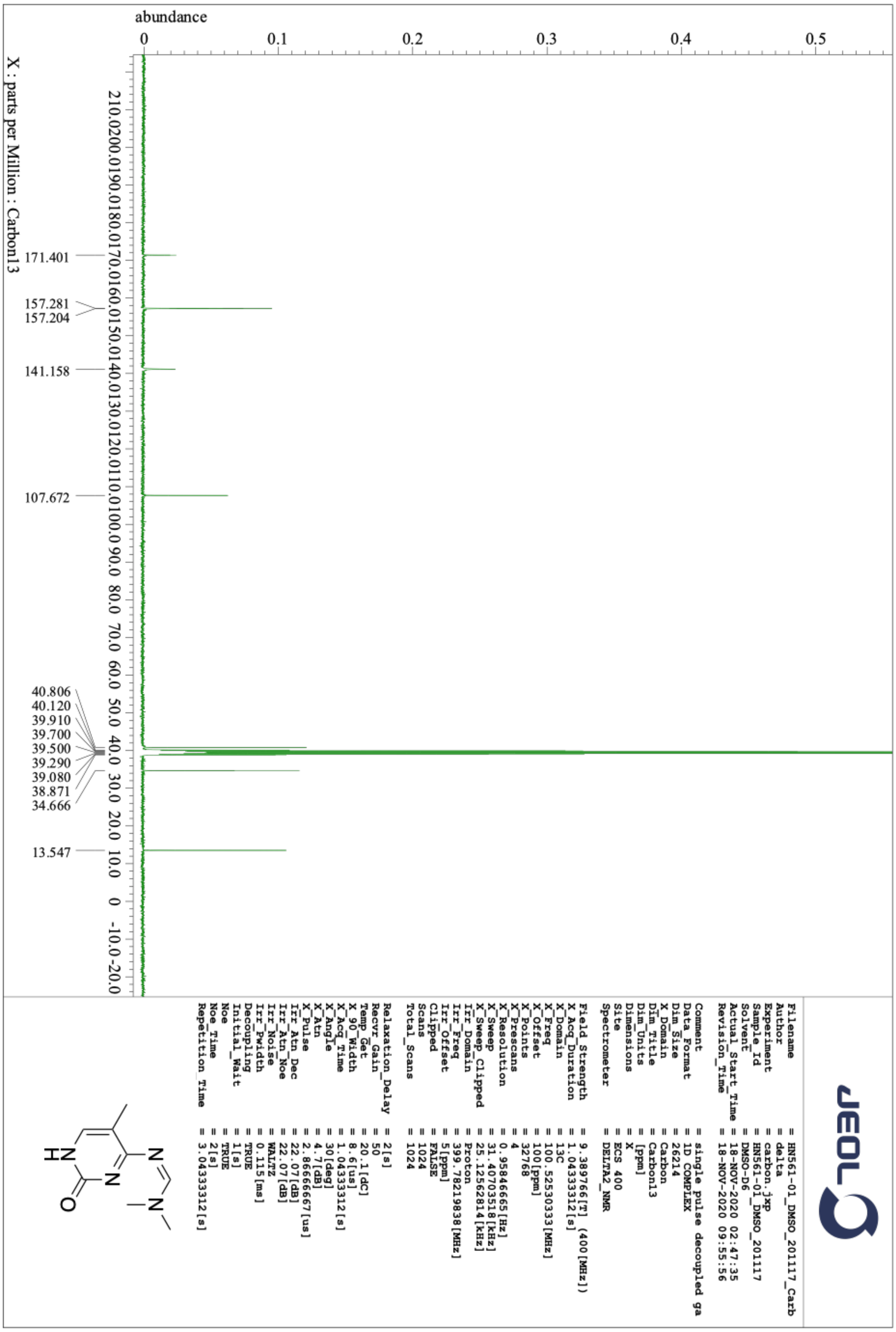

Compound **20** (^{1}H NMR, $CDCl_3$, 301 MHz)

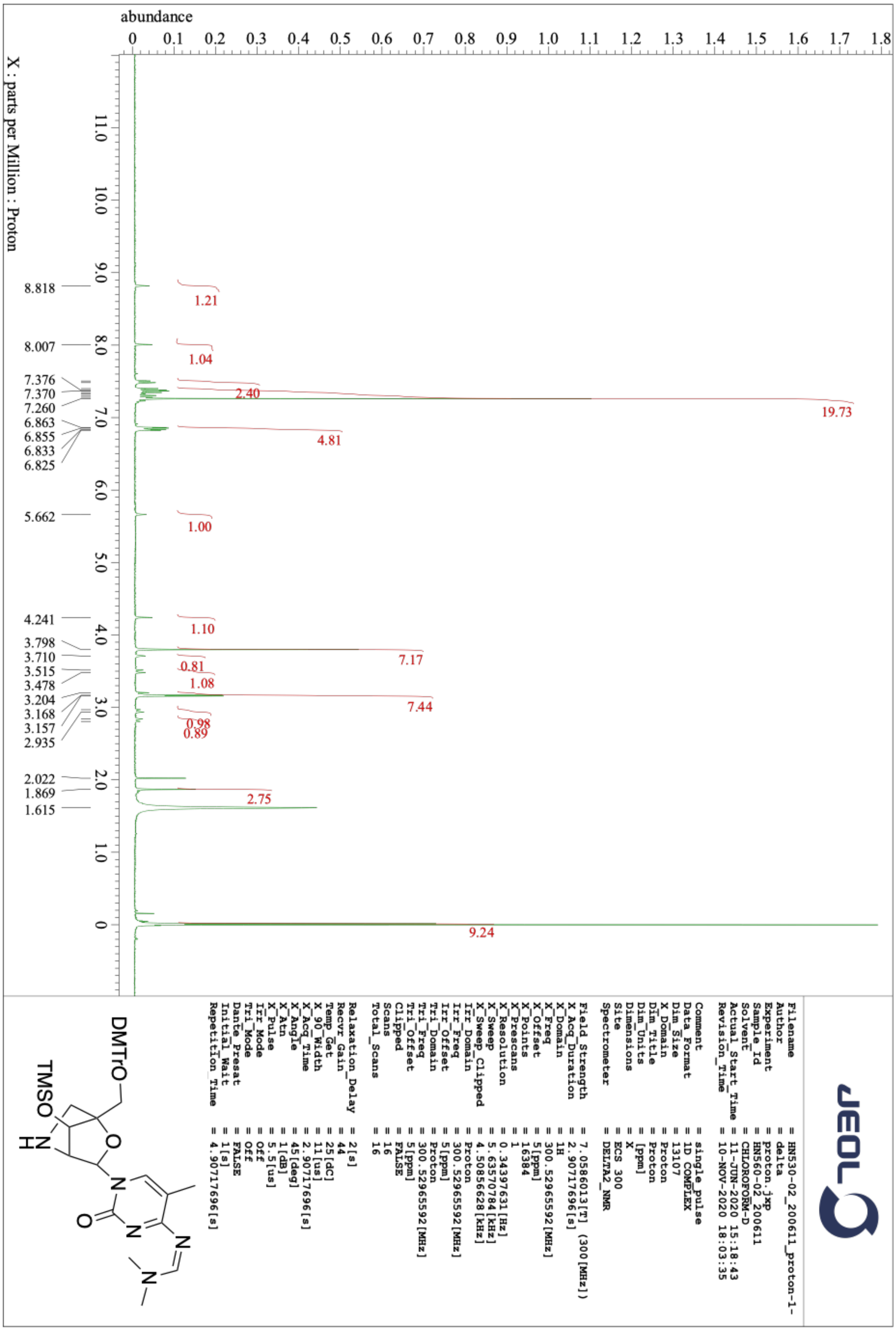

Compound **20** (^{13}C NMR, $CDCl_3$, 101 MHz)

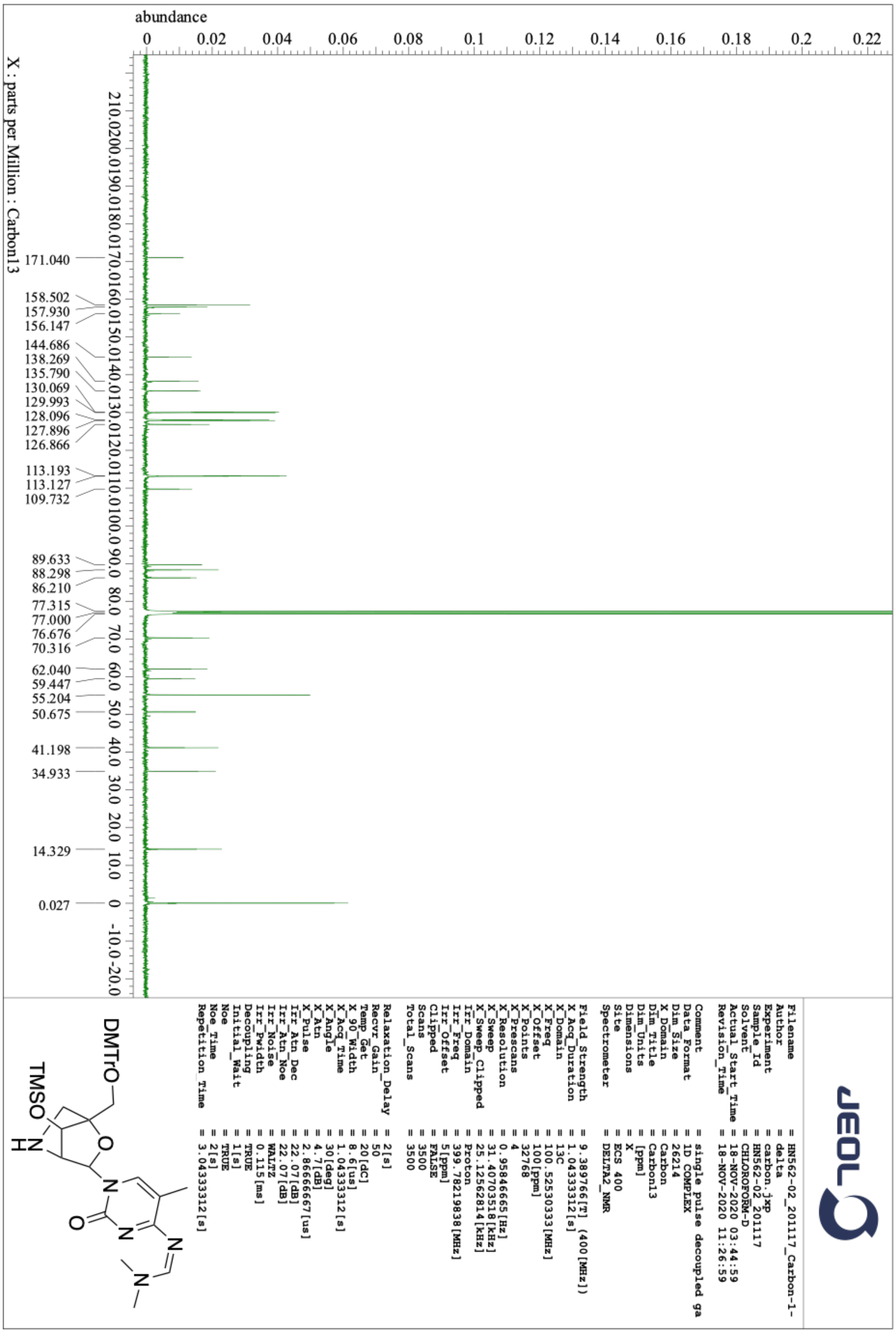

Compound **15** (^{1}H NMR, $CDCl_3$, 301 MHz)

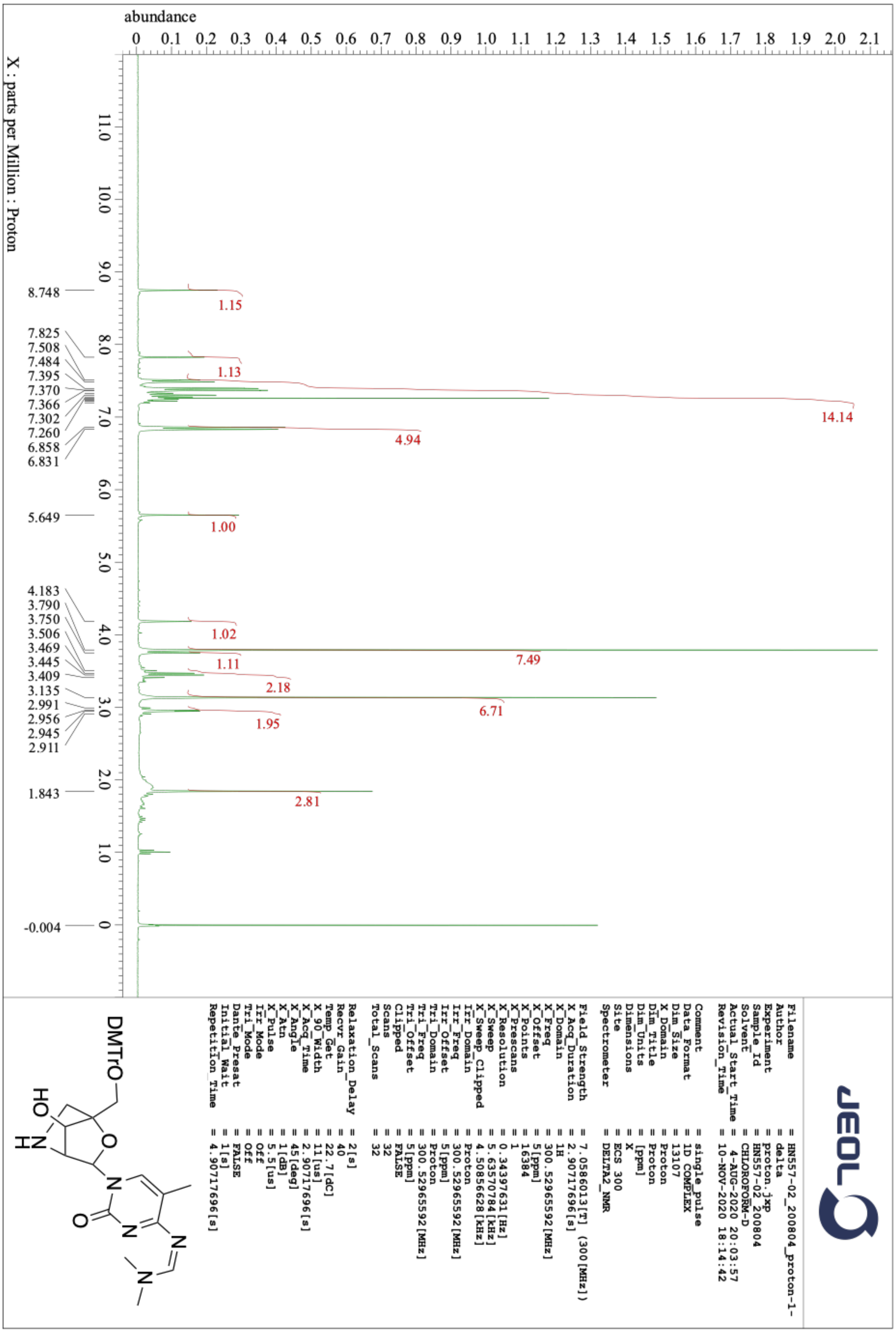

Compound **15** (^{13}C NMR, $CDCl_3$, 101 MHz)

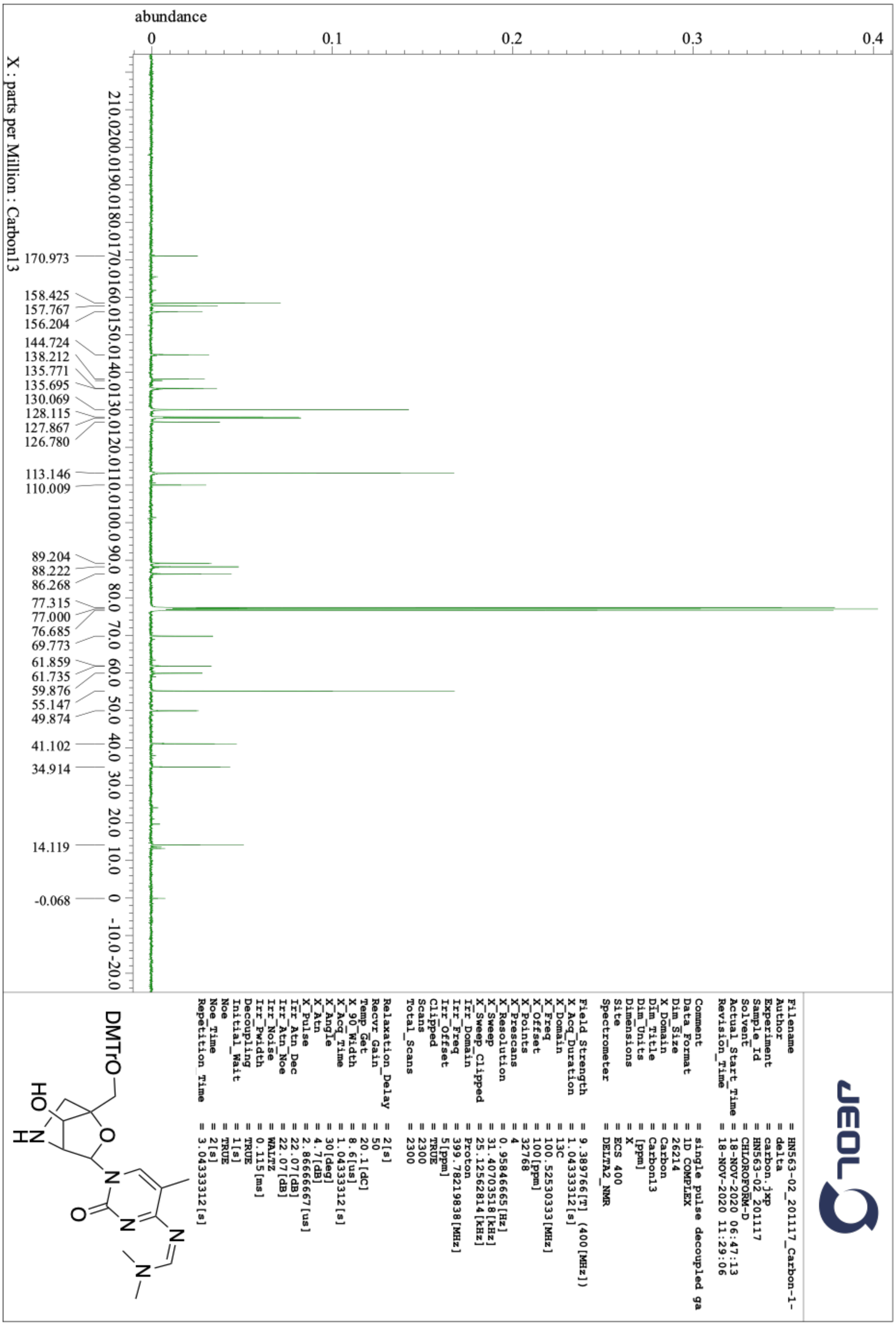

4. Characterization data (HPLC and mass data) of synthesized oligonucleotides

HPLC charts

ODN1 5'-d(GCG TTT TTT GCT)-3', T = GuNA[Et]-T

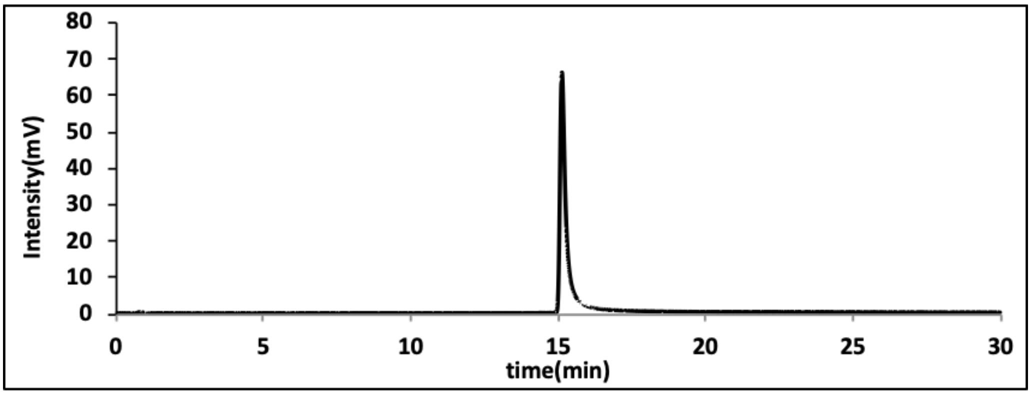

Linear gradient of acetonitrile (5 to 10% over 30 min) in 0.1 M TEAA buffer (pH 7.0) at 50 ºC.

ODN2 5'-d(GCG TTT TTT GCT)-3', T = GuNA[^{i}Pr]-T

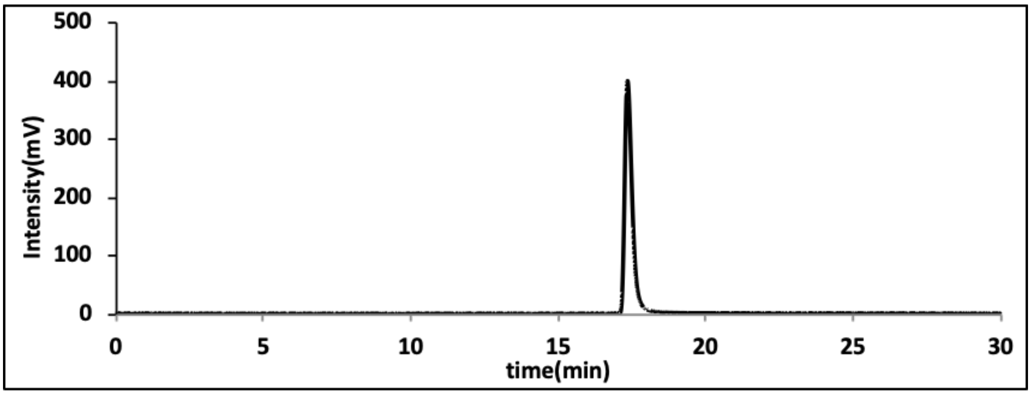

Linear gradient of acetonitrile (5 to 10% over 30 min) in 0.1 M TEAA buffer (pH 7.0) at 50 ºC.

ODN3 5'-d(GCG TTT TTT GCT)-3', T = GuNA[^{t}Bu]-T

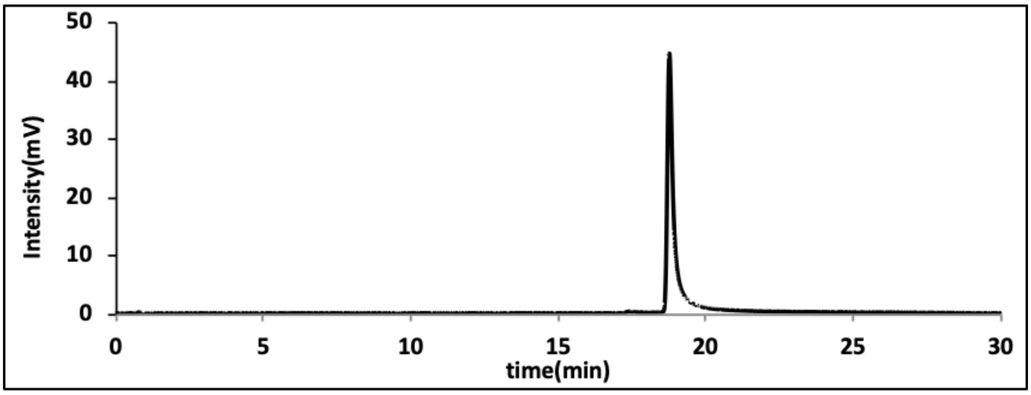

Linear gradient of acetonitrile (5 to 10% over 30 min) in 0.1 M TEAA buffer (pH 7.0) at 50 ºC.

ODN4 5'-d(GCG TTT TTT GCT)-3', T = GuNA[Me,Me]-T

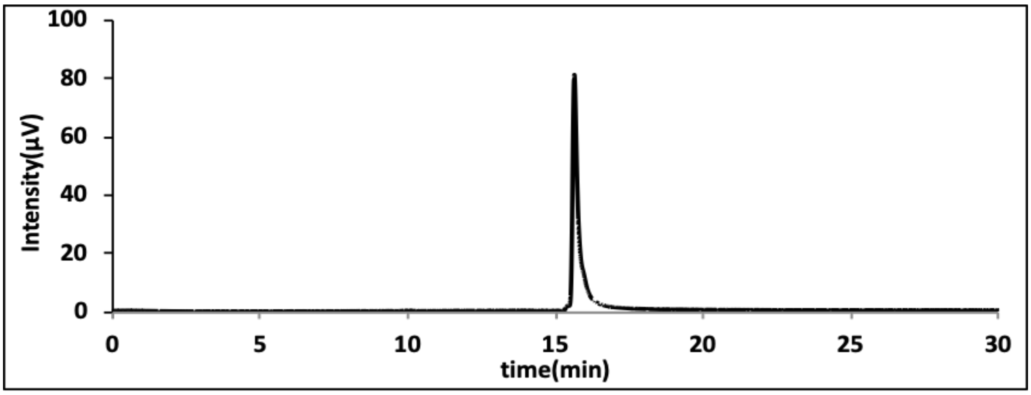

Linear gradient of acetonitrile (10 to 20% over 30 min) in 0.1 M TEAA buffer (pH 7.0) at 50 ºC.

ODN5 5'-d(GCG TTT TTT GCT)-3', T = GuNA[Me,^{t}Bu]-T

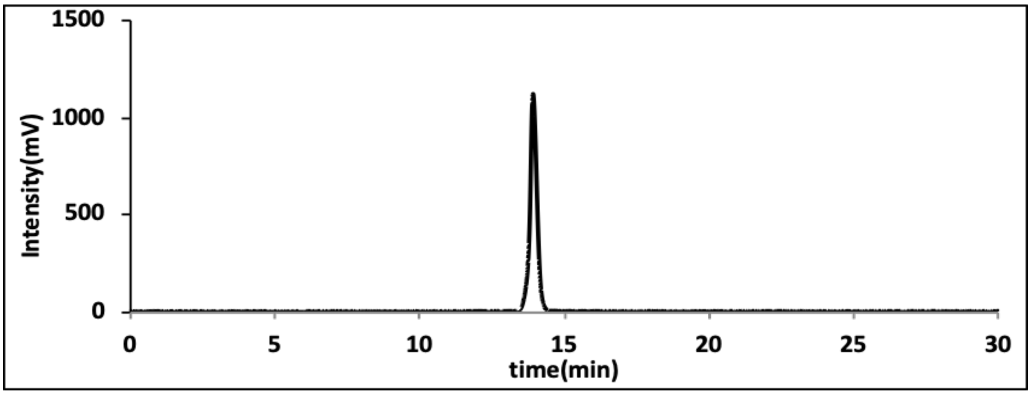

Linear gradient of methanol (5 to 20% over 30 min) in HFIP/triethylamine/H_2O (1/0.1/100 v/v) solution at 70 ºC.

ODN6 5'-d(GCG TTT TTT GCT)-3', T = GuNA[Me,Me]-T

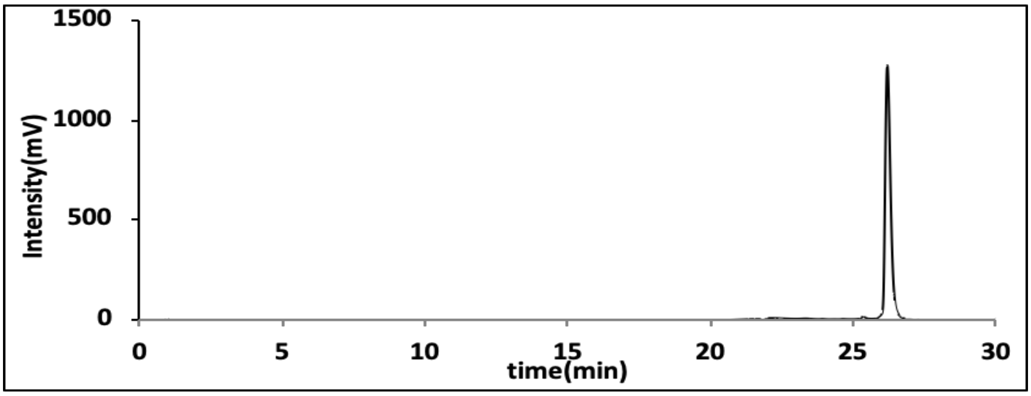

Linear gradient of acetonitrile (0 to 10% over 30 min) in 0.1 M TEAA buffer (pH 7.0) at 60 ºC.

ODN7 5'-d(GCG TTT TTT GCT)-3', T = GuNA[Me,^{i}Bu]-T

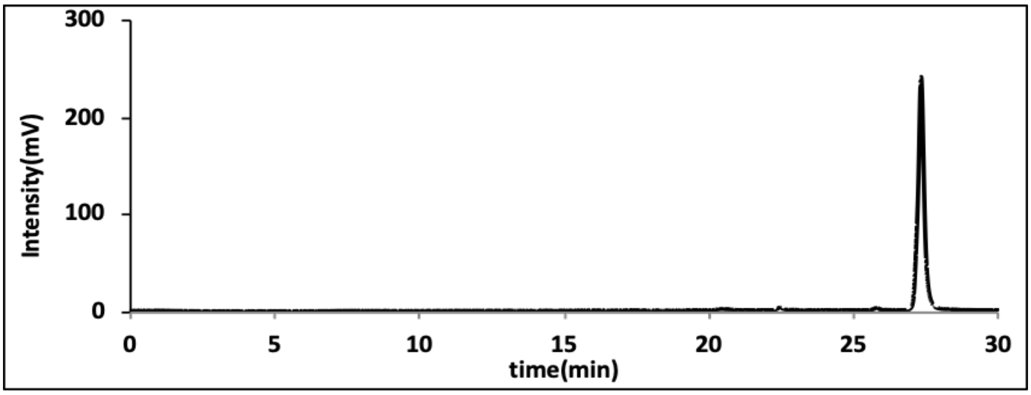

Linear gradient of acetonitrile (0 to 10% over 30 min) in 0.1 M TEAA buffer (pH 7.0) at 60 ºC.

ODN8 5'-d(TTT TTT TTT T)-3', T = GuNA[Et]-T

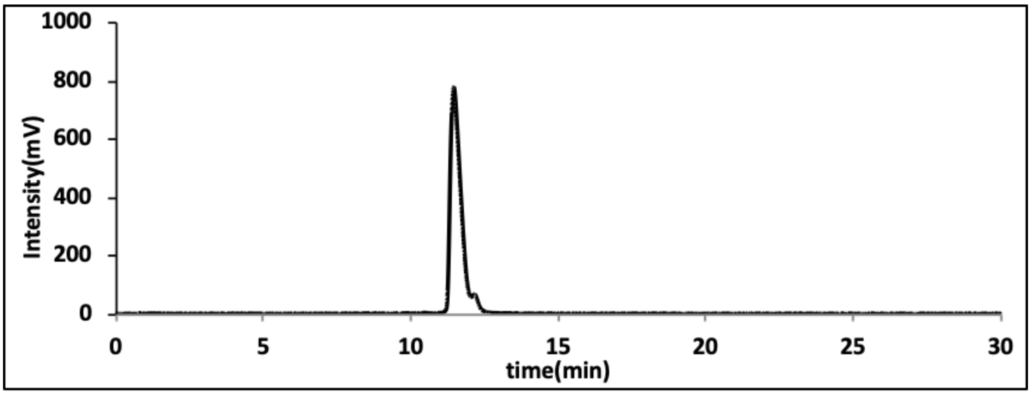

Linear gradient of acetonitrile (7.5 to 12.5% over 30 min) in 0.1 M TEAA buffer (pH 7.0) at 50 ºC.

ODN9 5'-d(TTT TTT TTT T)-3', T = GuNA[^{i}Pr]-T

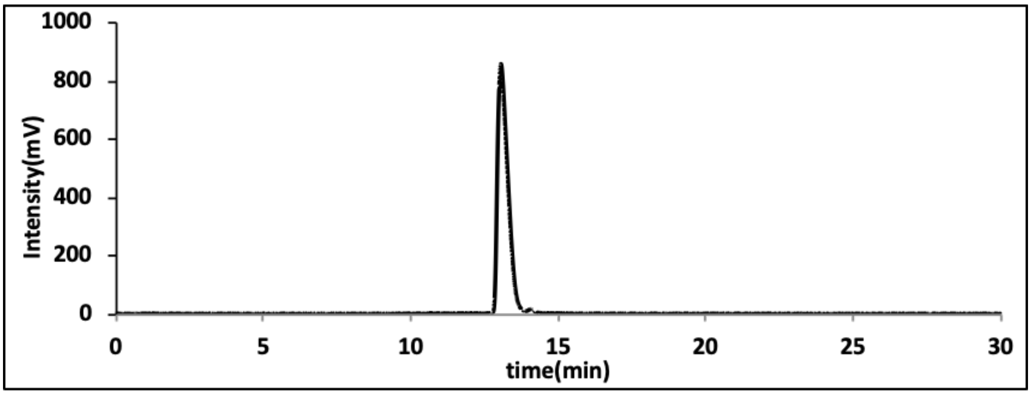

Linear gradient of acetonitrile (7.5 to 12.5% over 30 min) in 0.1 M TEAA buffer (pH 7.0) at 50 ºC.

ODN10 5'-d(TTT TTT TT<u>T</u> T)-3', <u>T</u> = GuNA[^{t}Bu]-T

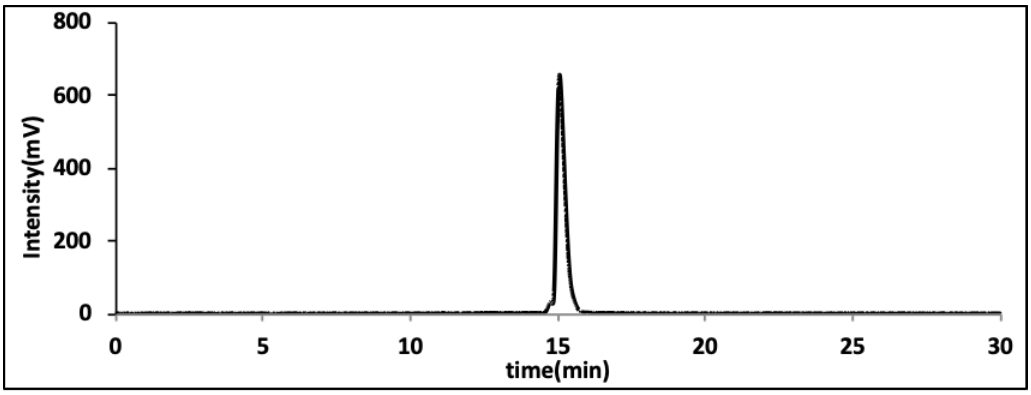

Linear gradient of acetonitrile (7.5 to 12.5% over 30 min) in 0.1 M TEAA buffer (pH 7.0) at 50 ºC.

ODN11 5'-d(TTT TTT TT<u>T</u> T)-3', <u>T</u> = GuNA[Me,Me]-T

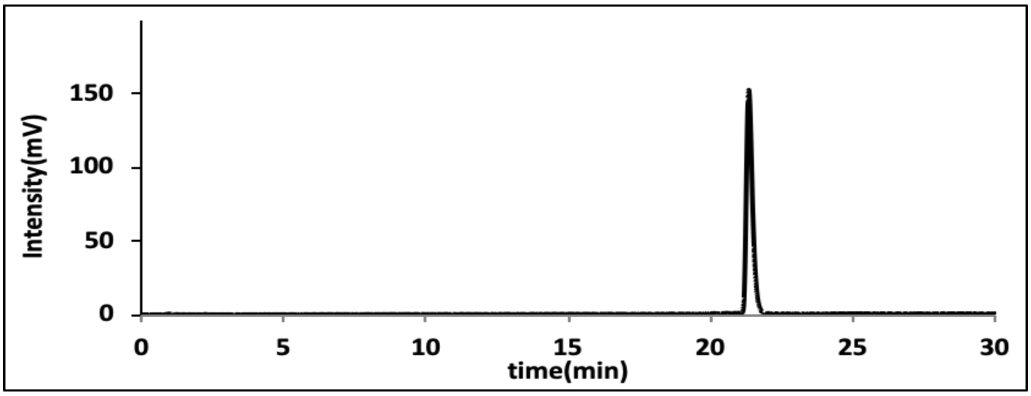

Linear gradient of acetonitrile (7.5 to 12.5% over 30 min) in 0.1 M TEAA buffer (pH 7.0) at 50 ºC.

ODN12 5'-d(TTT TTT TT<u>T</u> T)-3', <u>T</u> = GuNA[Me,^{t}Bu]-T

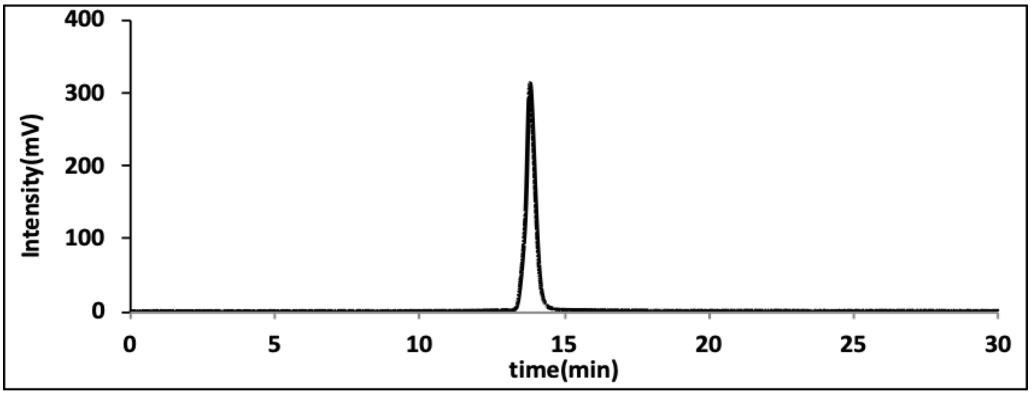

Linear gradient of acetonitrile (7.5 to 12.5% over 30 min) in 0.1 M TEAA buffer (pH 7.0) at 50 ºC.

ODN13 5'-d(GCG TTA TTT GCT)-3', A = GuNA[Me,Me]-A

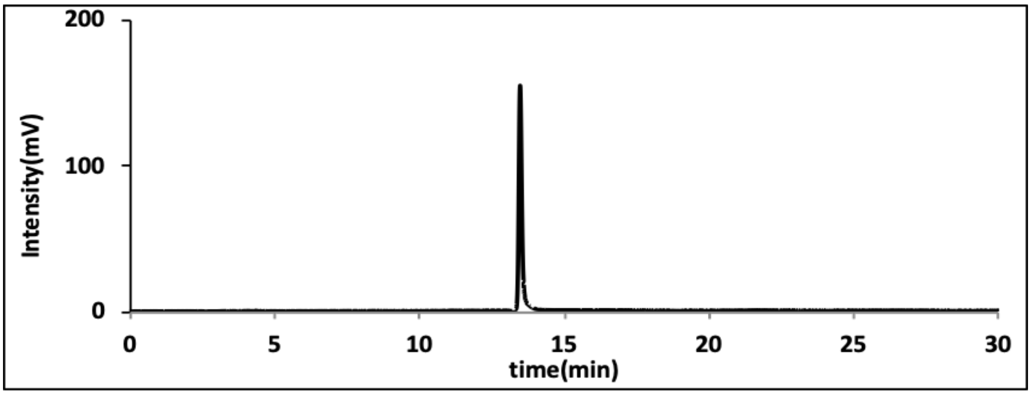

Linear gradient of acetonitrile (5 to 15% over 30 min) in 0.1 M TEAA buffer (pH 7.0) at 50 ºC.

ODN14 5'-d(GCG TTA TTT GCT)-3', A = GuNA[Me,^{t}Bu]-A

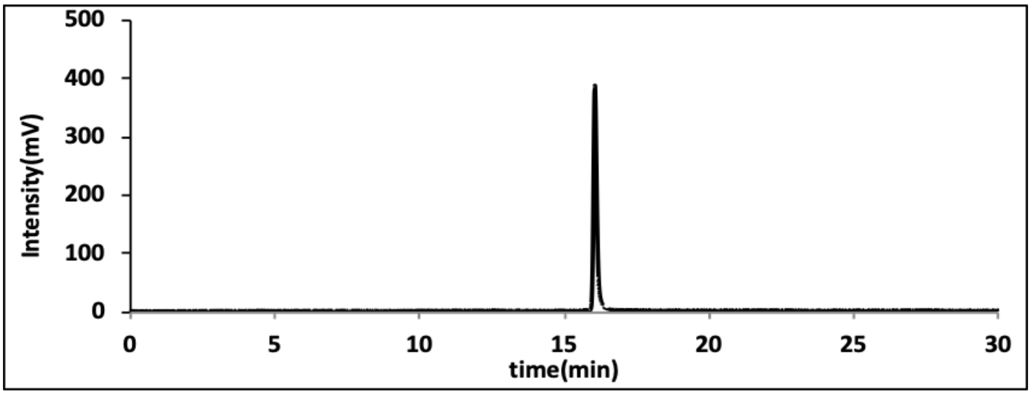

Linear gradient of acetonitrile (5 to 15% over 30 min) in 0.1 M TEAA buffer (pH 7.0) at 50 ºC.

ODN15 5'-d(GCG TTG TTT GCT)-3', G = GuNA[Me,Me]-G

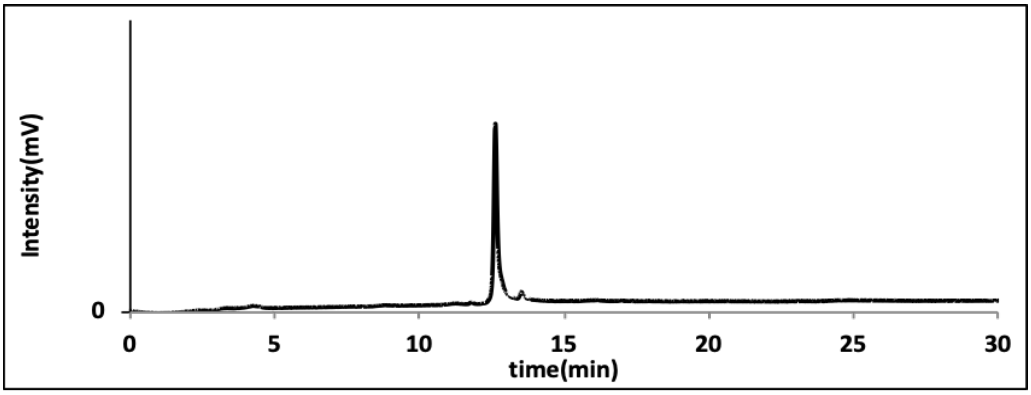

Linear gradient of acetonitrile (5 to 15% over 30 min) in 0.1 M TEAA buffer (pH 7.0) at 50 ºC.

ODN16 5'-d(GCG TTG TTT GCT)-3', G = GuNA[Me,^{t}Bu]-G

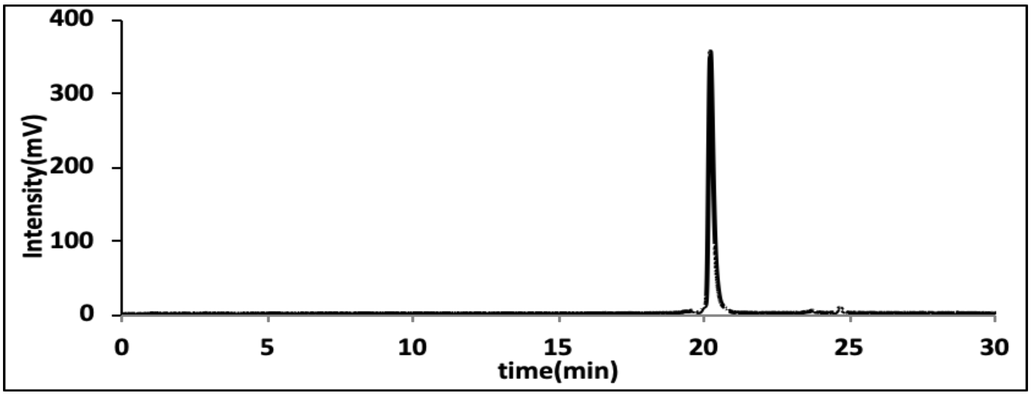

Linear gradient of acetonitrile (5 to 15% over 30 min) in 0.1 M TEAA buffer (pH 7.0) at 50 ºC.

ODN17 5'-d(GCG TT^{m}C TTT GCT)-3', ^{m}C = GuNA[Me,Me]-^{m}C

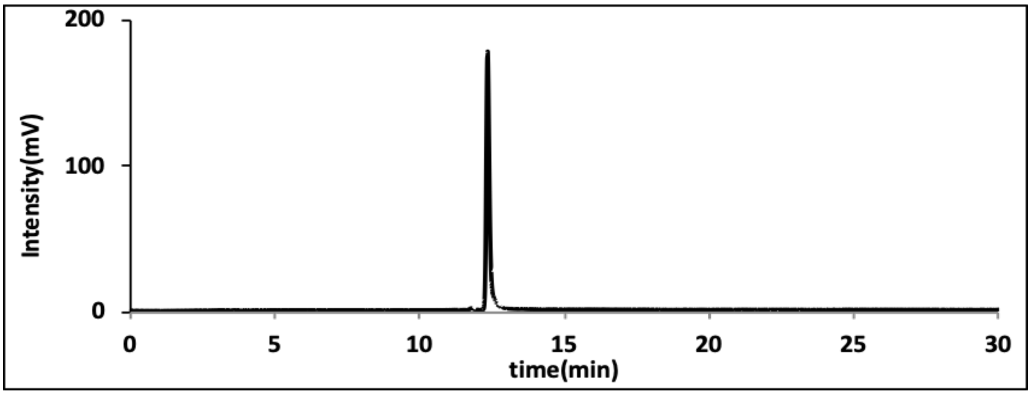

Linear gradient of acetonitrile (5 to 15% over 30 min) in 0.1 M TEAA buffer (pH 7.0) at 50 ºC.

ODN18 5'-d(GCG TT^{m}C TTT GCT)-3', ^{m}C = GuNA[Me,^{t}Bu]-^{m}C

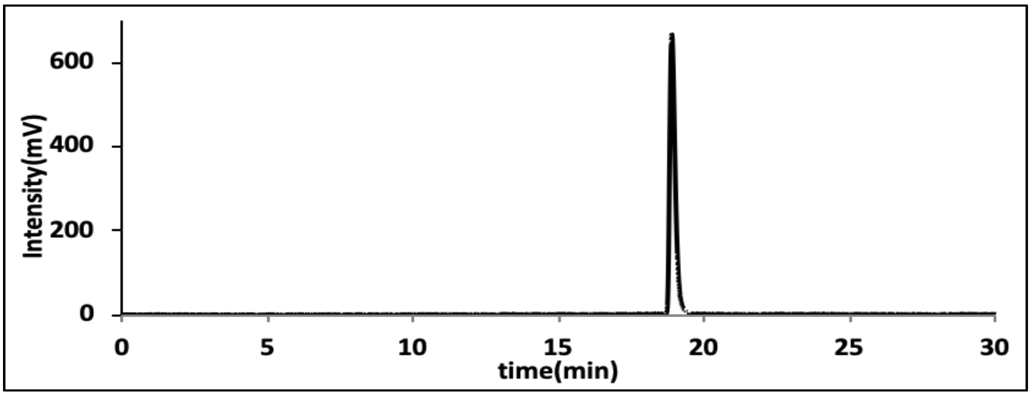

Linear gradient of acetonitrile (5 to 15% over 30 min) in 0.1 M TEAA buffer (pH 7.0) at 50 ºC.

ODN24 5'-d(GCG TA<u>T</u> ACG C)-3', <u>T</u> = GuNA[Me,Me]-T

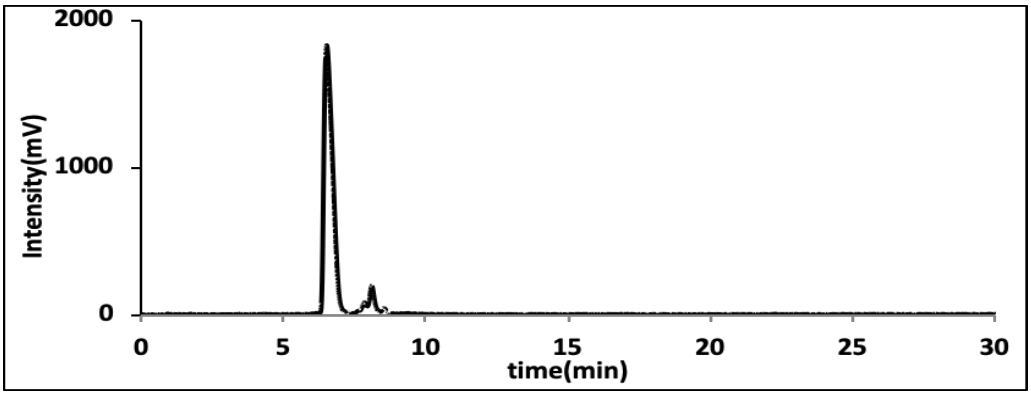

Linear gradient of methanol (5 to 20% over 30 min) in HFIP/triethylamine/H_2O (1/0.1/100 v/v) solution at 70 ºC.

ODN25 5'-d(GCG TA<u>T</u> ACG C)-3', <u>T</u> = GuNA[Me,^{t}Bu]-T

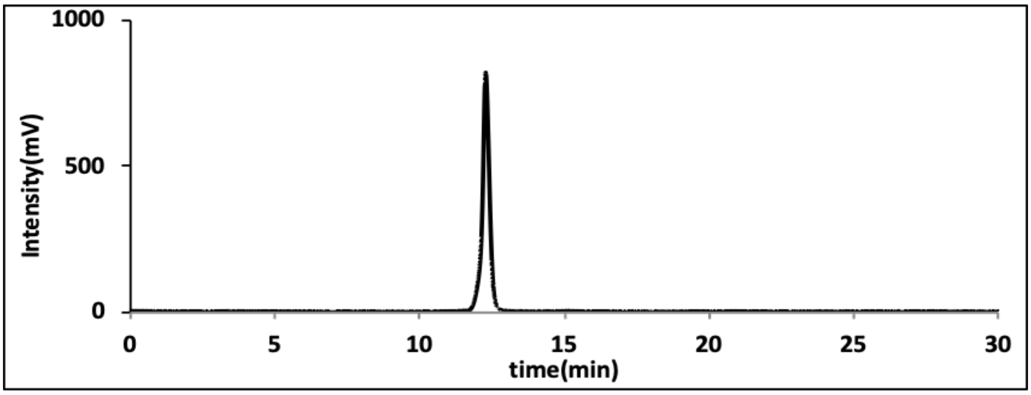

Linear gradient of methanol (5 to 20% over 30 min) in HFIP/triethylamine/H_2O (1/0.1/100 v/v) solution at 70 ºC.

ODN26 5'-d(GCG <u>T</u>AT ACG C)-3', <u>T</u> = GuNA[Me,Me]-T

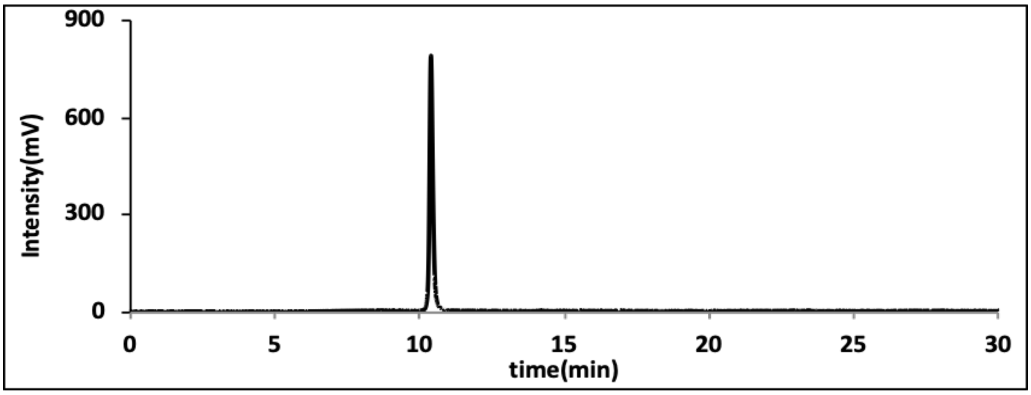

Linear gradient of acetonitrile (0 to 10% over 30 min) in HFIP/triethylamine/H_2O (1/0.1/100 v/v) solution at 70 ºC.

ODN27 5'-d(GCG T̲AT ACG C)-3', T̲ = GuNA[Me,^{t}Bu]-T

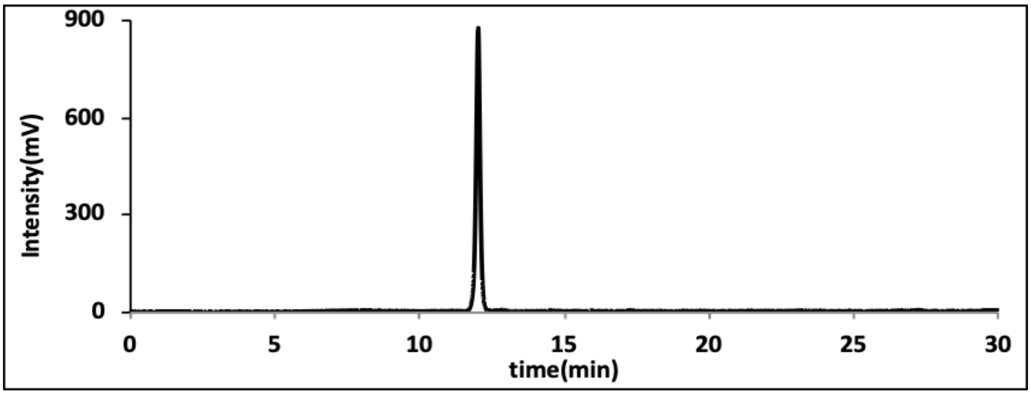

Linear gradient of acetonitrile (0 to 10% over 30 min) in HFIP/triethylamine/H_2O (1/0.1/100 v/v) solution at 70 ºC.

ODN28 5'-d(GT̲G U^{Br}AC AC)-3', T̲ = GuNA[Me,Me]-T

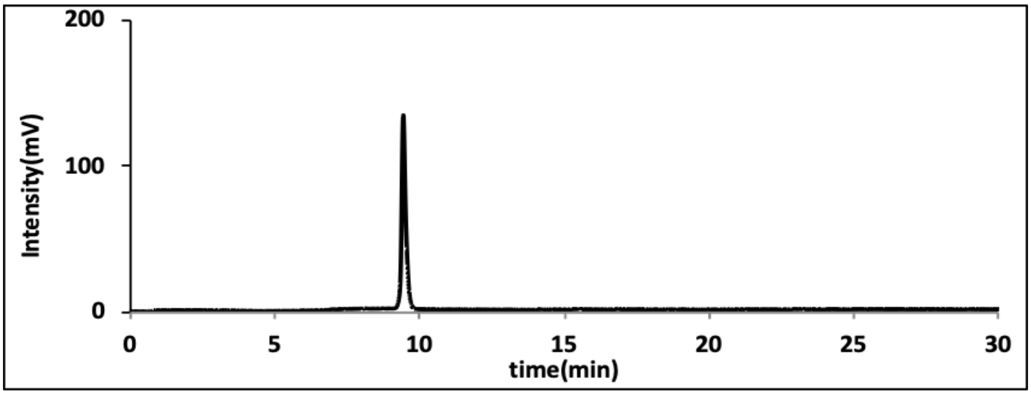

Linear gradient of acetonitrile (0 to 10% over 30 min) in HFIP/triethylamine/H_2O (1/0.1/100 v/v) solution at 70 ºC.

ODN29 5'-d(GT̲G U^{Br}AC AC)-3', T̲ = GuNA[Me,^{t}Bu]-T

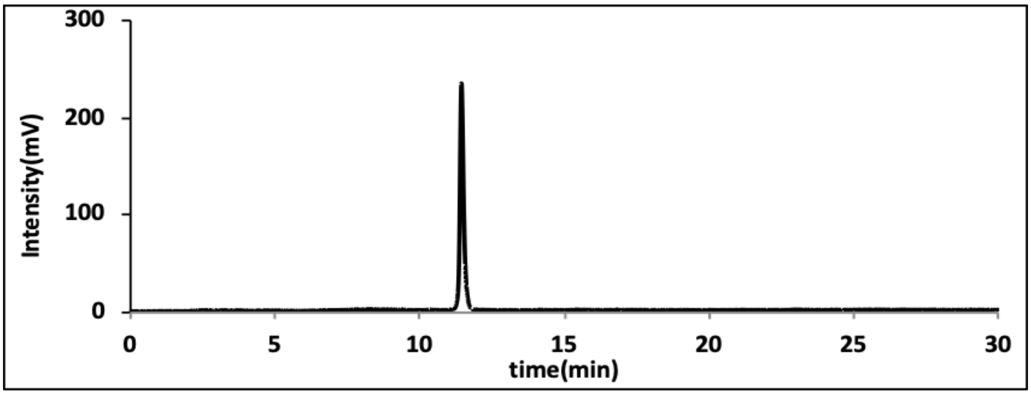

Linear gradient of acetonitrile (0 to 10% over 30 min) in HFIP/triethylamine/H_2O (1/0.1/100 v/v) solution at 70 ºC.

ODN30 5'-d(GTG $U^{Br}A\underline{^{m}C}$ AC)-3', $\underline{^{m}C}$ = GuNA[Me,Me]-^{m}C

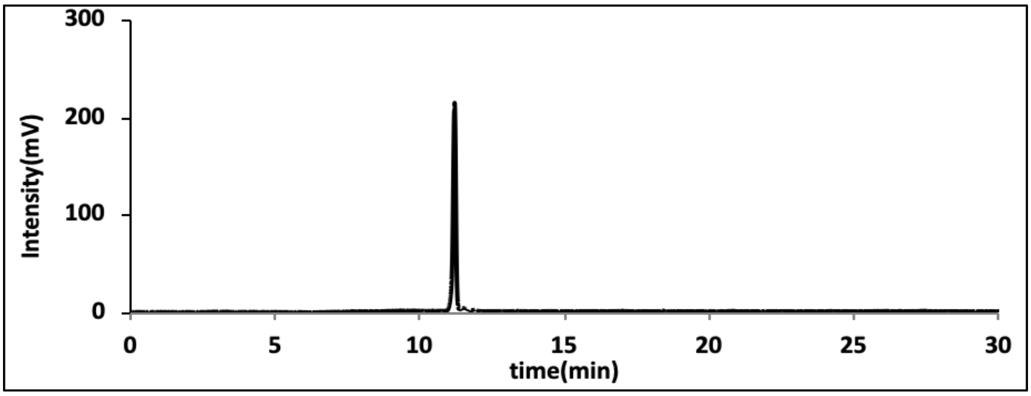

Linear gradient of acetonitrile (0 to 10% over 30 min) in HFIP/triethylamine/H_2O (1/0.1/100 v/v) solution at 70 ºC.

ODN31 5'-d(GTG $U^{Br}A\underline{^{m}C}$ AC)-3', $\underline{^{m}C}$ = GuNA[Me,^{t}Bu]-^{m}C

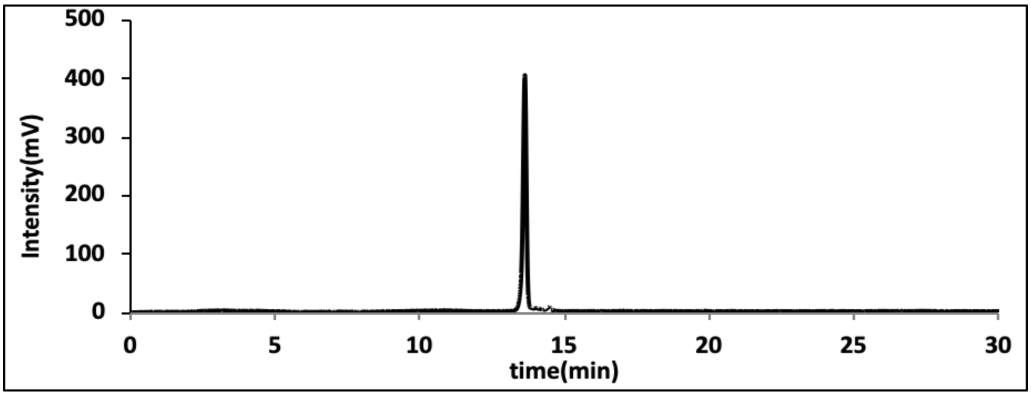

Linear gradient of acetonitrile (0 to 10% over 30 min) in HFIP/triethylamine/H_2O (1/0.1/100 v/v) solution at 70 ºC.

ODN32 5'-d(GTG U^{Br}AC $\underline{A}$C)-3', $\underline{A}$ = GuNA[Me,Me]-A

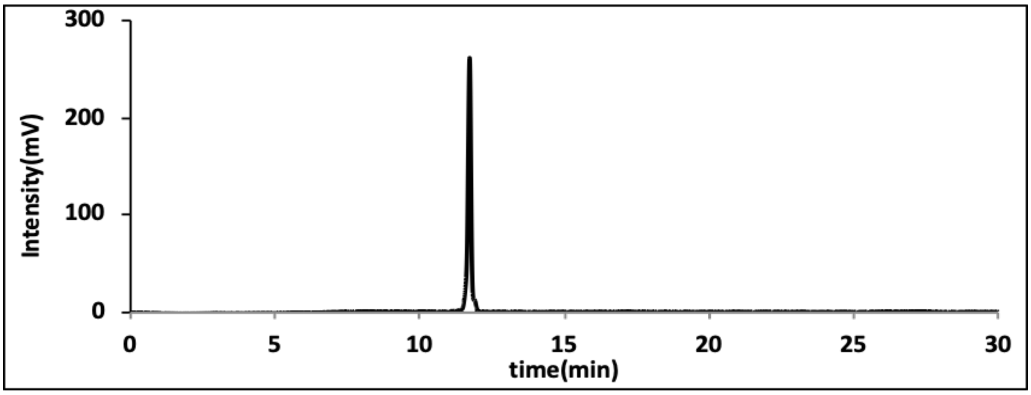

Linear gradient of acetonitrile (0 to 10% over 30 min) in HFIP/triethylamine/H_2O (1/0.1/100 v/v) solution at 70 ºC.

ODN33 5'-d(GTG U^{Br}AC A̲C)-3', A̲ = GuNA[Me,^{t}Bu]-A

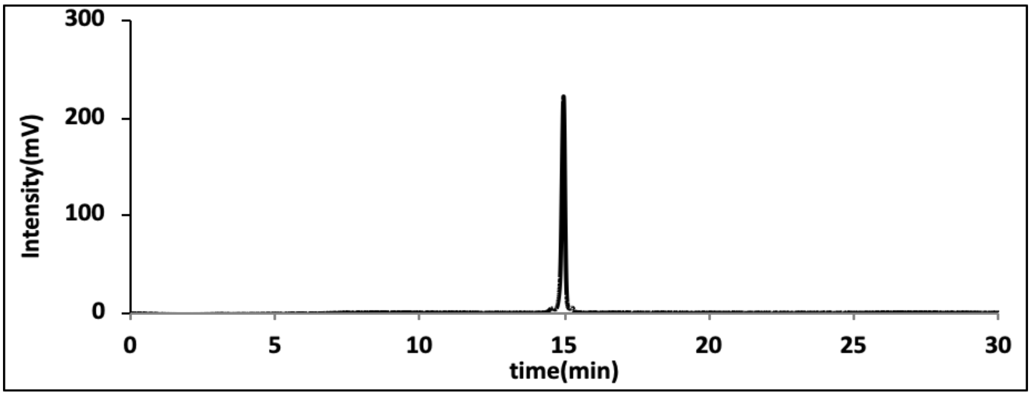

Linear gradient of acetonitrile (0 to 10% over 30 min) in HFIP/triethylamine/H_2O (1/0.1/100 v/v) solution at 70 ºC.

Mass data

ODN1 5'-d(GCG TT<u>T</u> TTT GCT)-3', <u>T</u> = GuNA[Et]-T

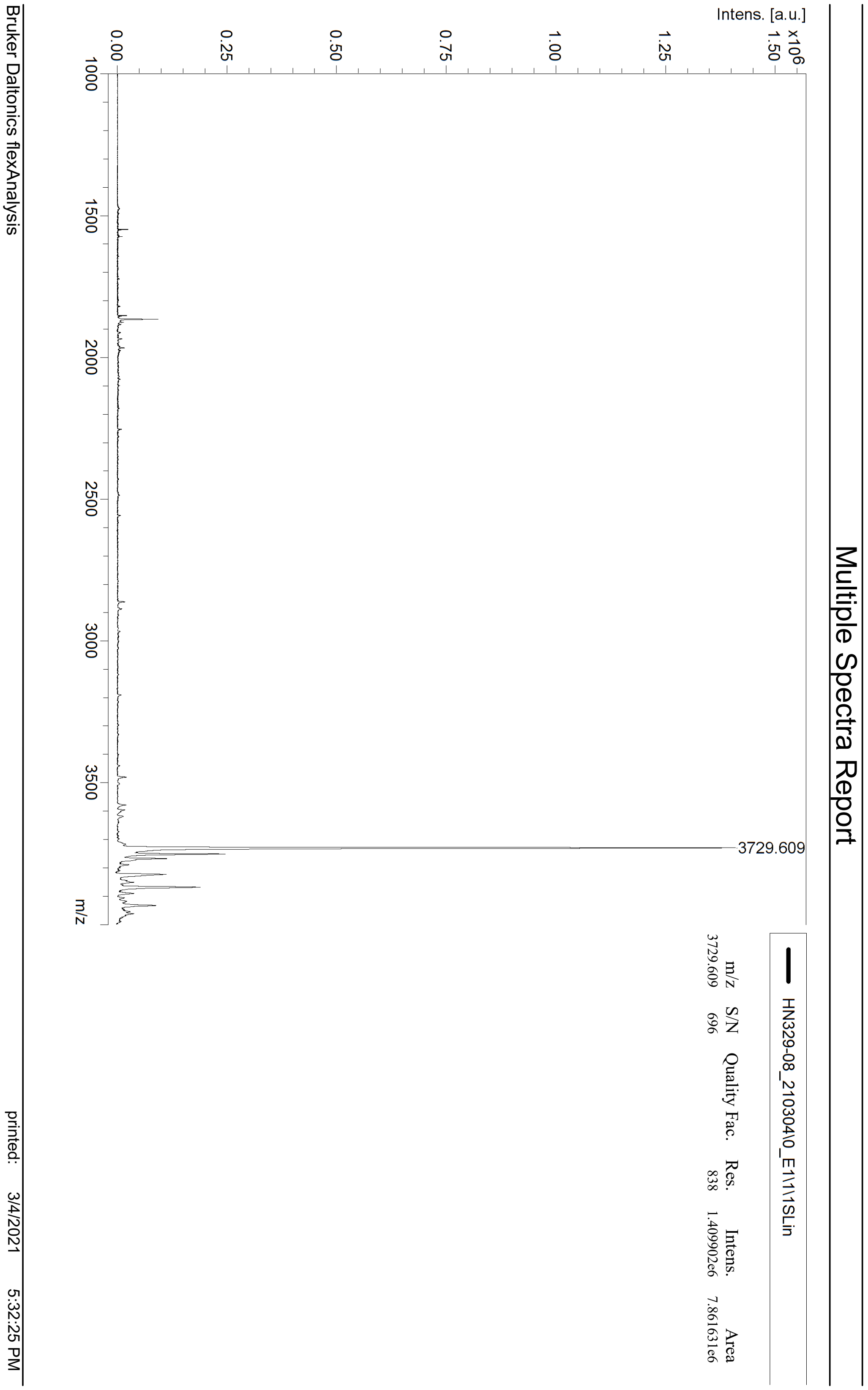

ODN2 5'-d(GCG TT$\underline{T}$ TTT GCT)-3', $\underline{T}$ = GuNA[^{i}Pr]-T

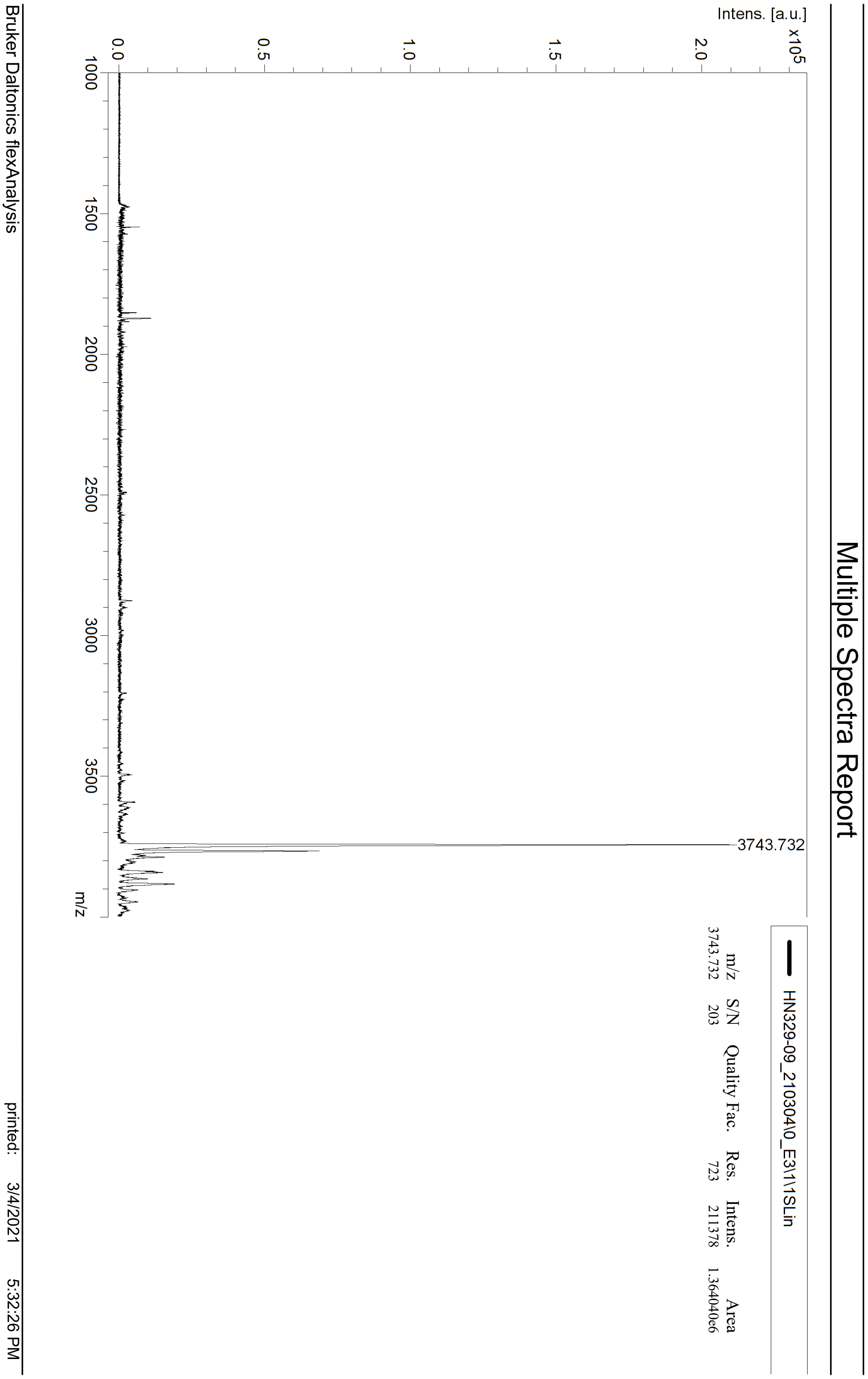

ODN3 5'-d(GCG TT<u>T</u> TTT GCT)-3', <u>T</u> = GuNA[^{t}Bu]-T

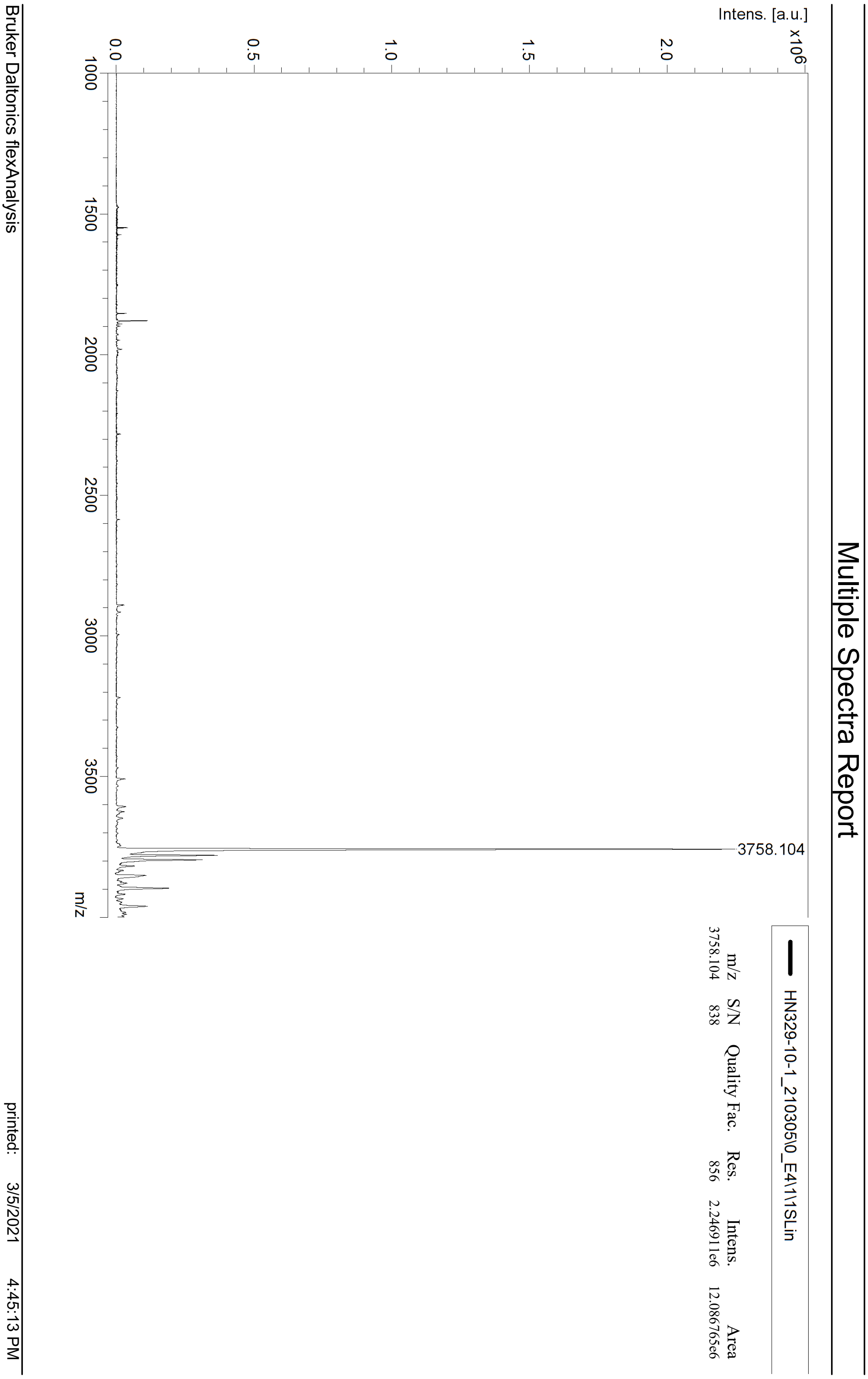

ODN4 5'-d(GCG TT<u>T</u> TTT GCT)-3', <u>T</u> = GuNA[Me,Me]-T

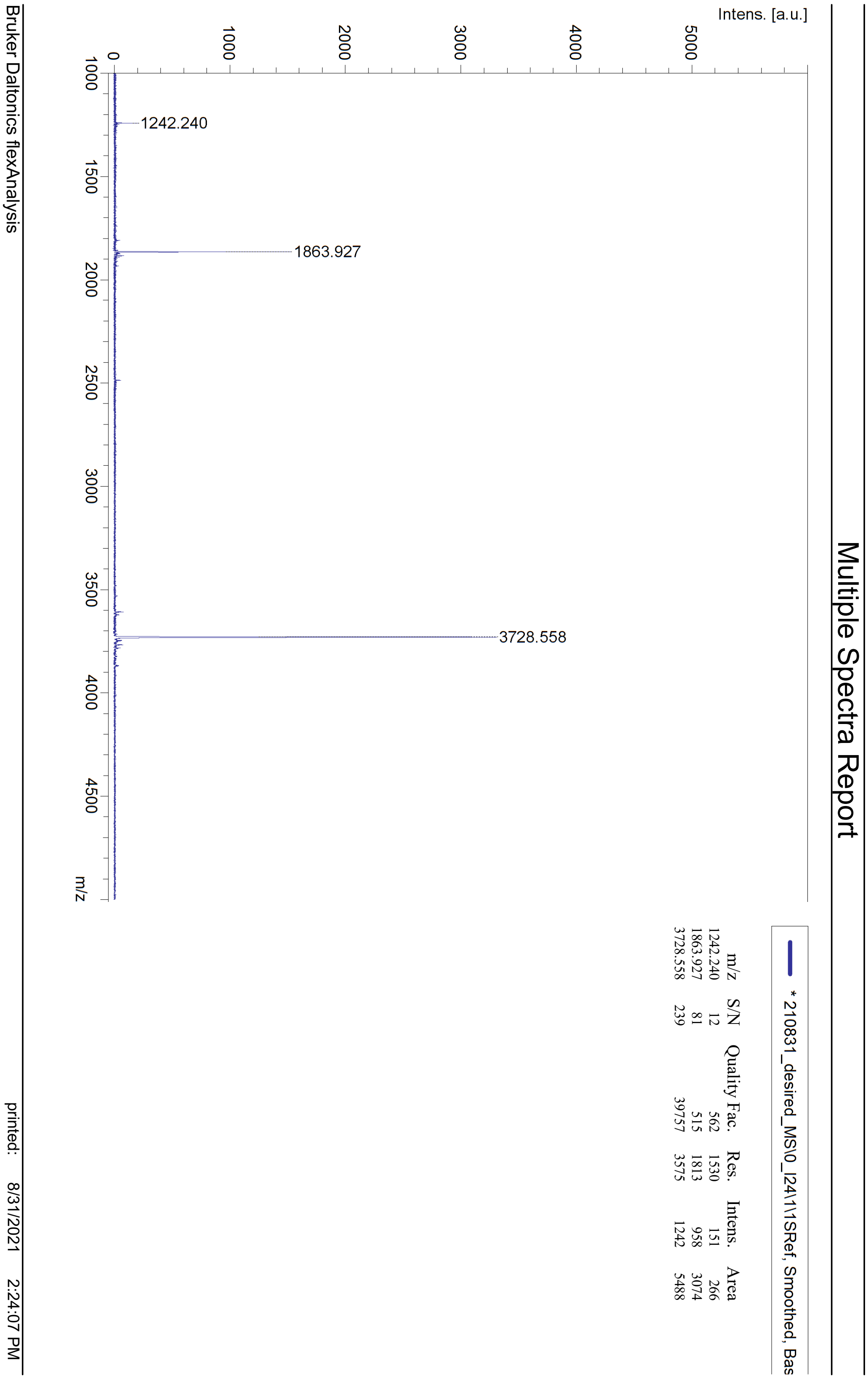

ODN5 5'-d(GCG TTT TTT GCT)-3', T = GuNA[Me,^{t}Bu]-T

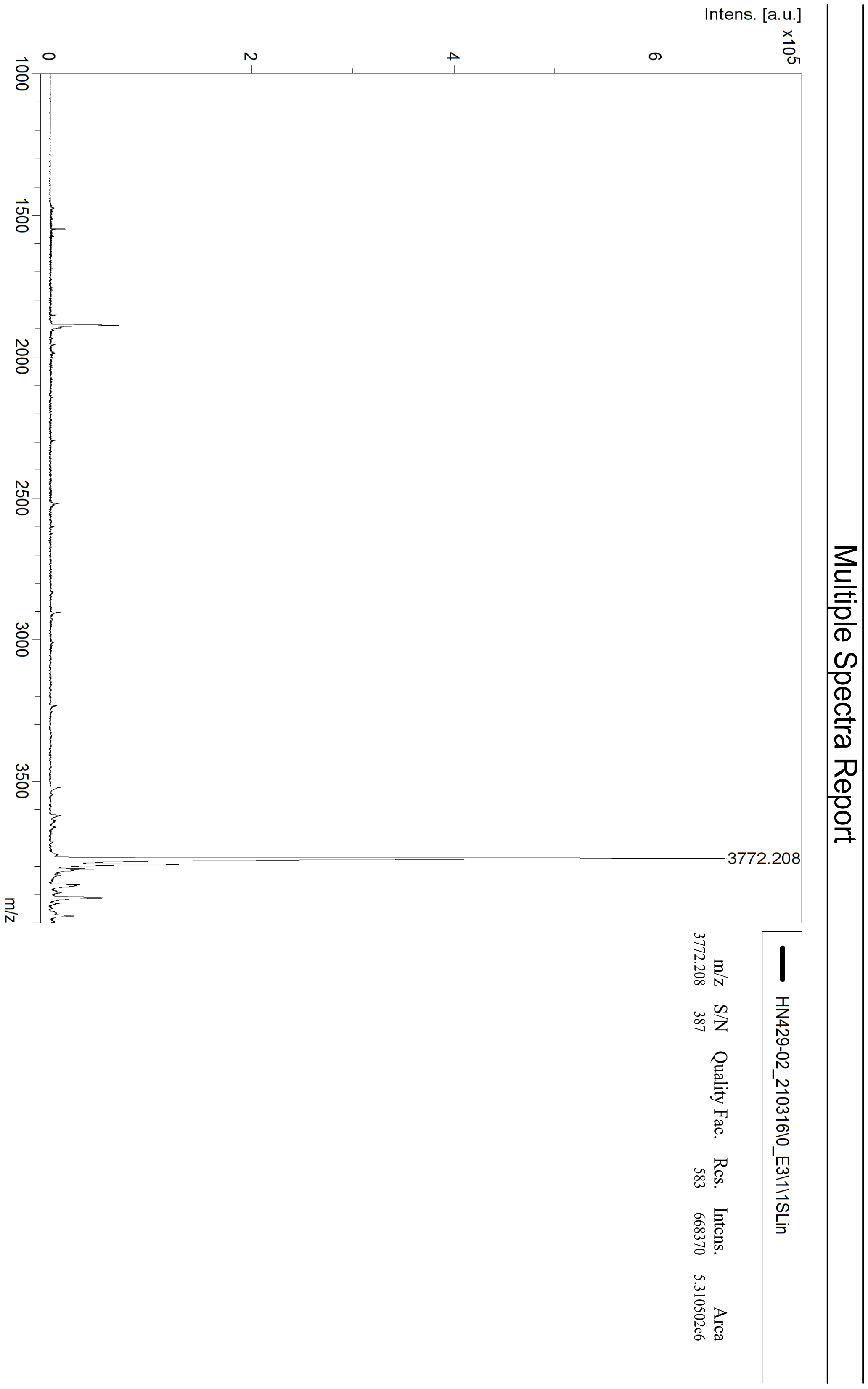

ODN6 5'-d(GCG T<u>TT</u> <u>T</u>TT GCT)-3', <u>T</u> = GuNA[Me,Me]-T

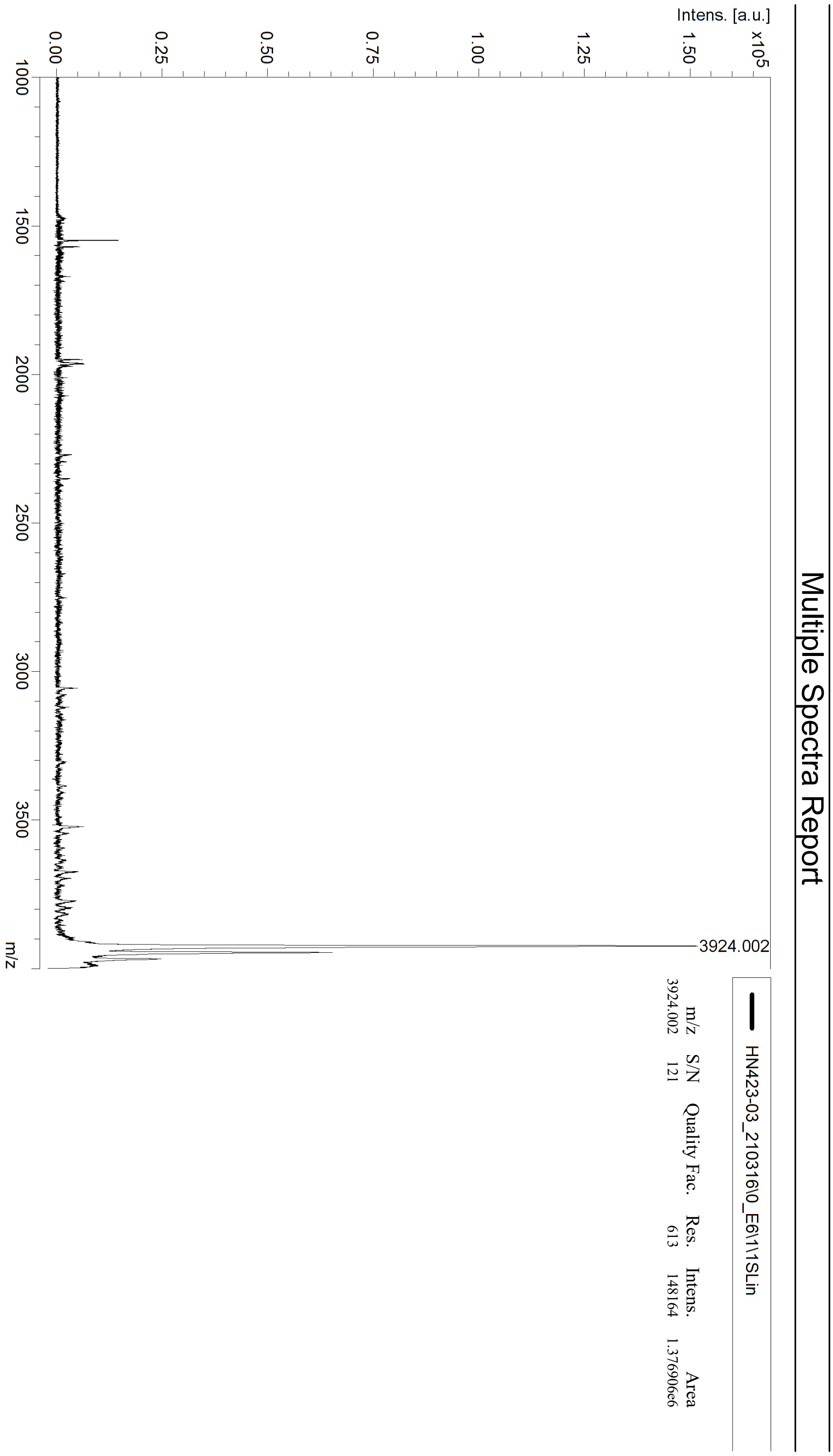

ODN7 5'-d(GCG TTT TTT GCT)-3', T = GuNA[Me,^{t}Bu]-T

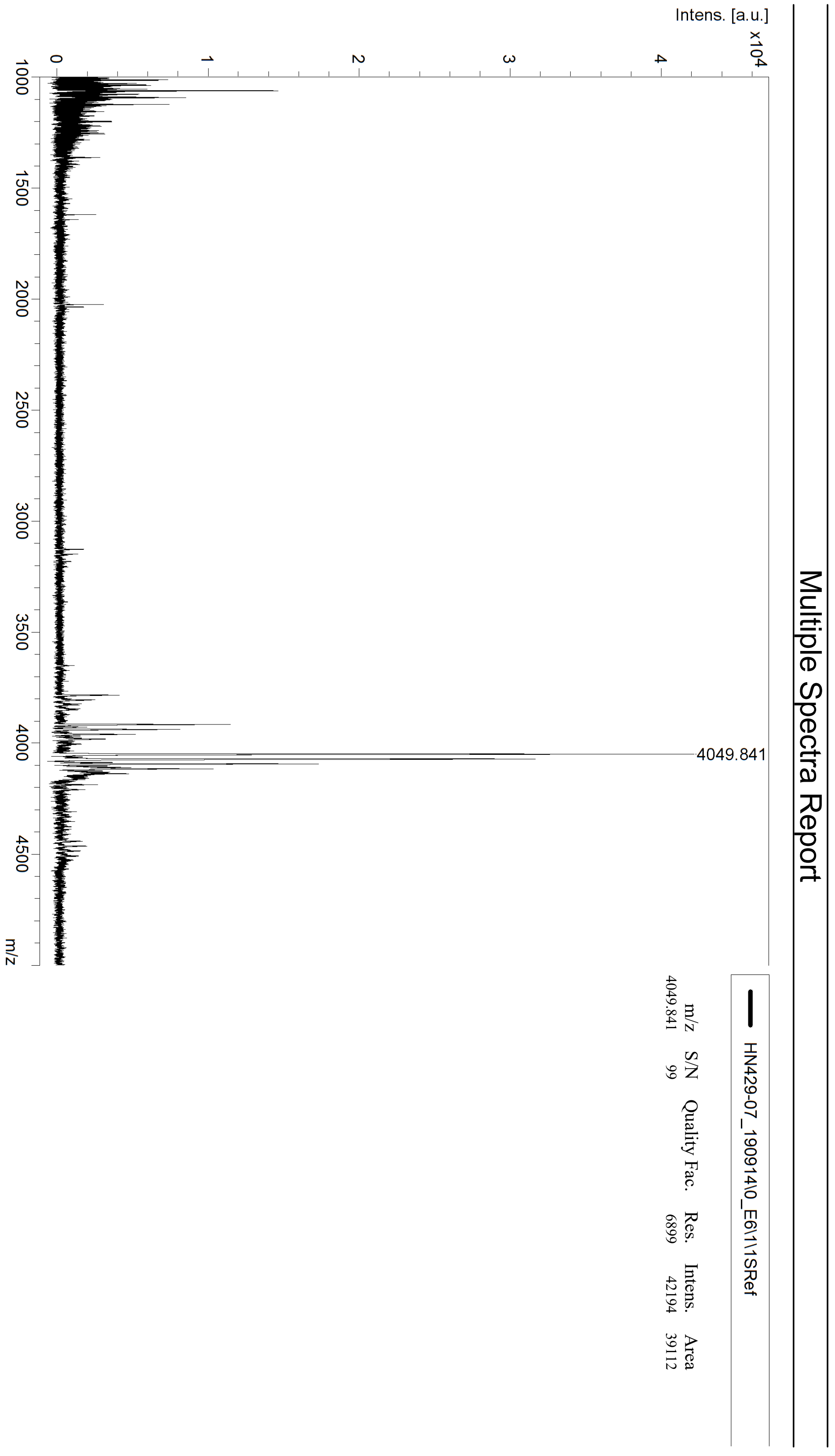

ODN8 5'-d(TTT TTT TT<u>T</u> T)-3', <u>T</u> = GuNA[Et]-T

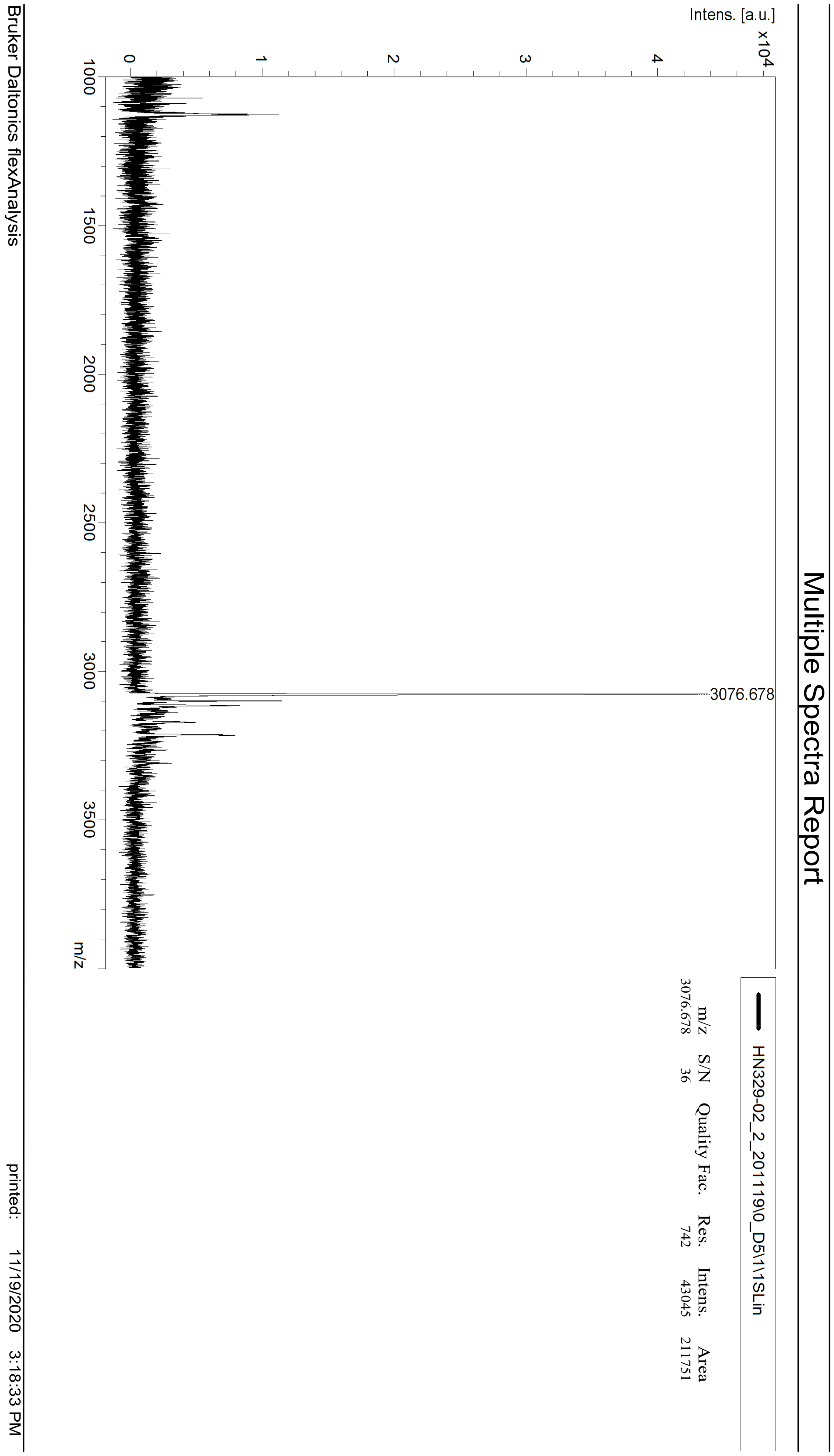

ODN9 5'-d(TTT TTT TT<u>T</u> T)-3', <u>T</u> = GuNA[^{i}Pr]-T

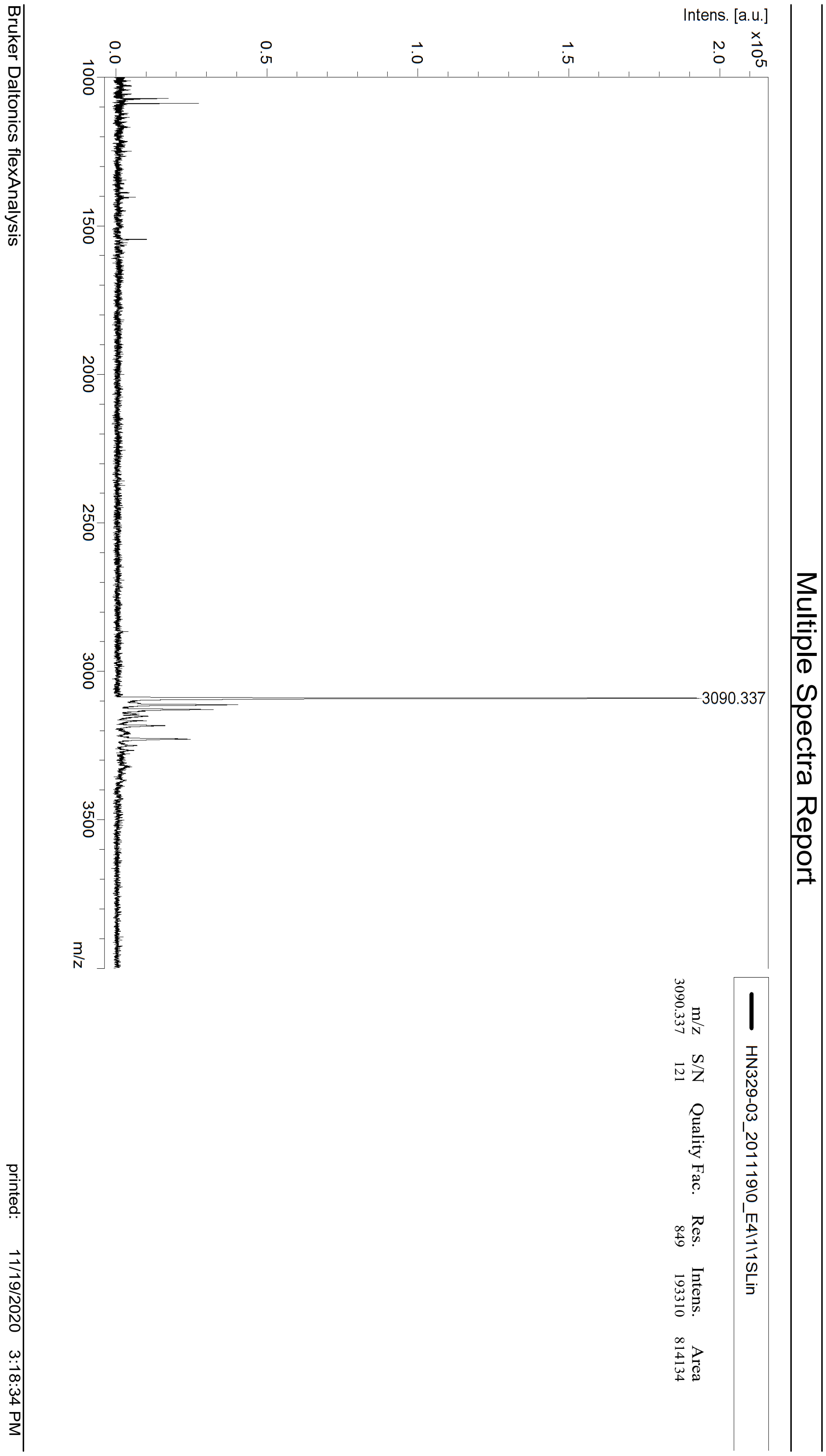

ODN10 5'-d(TTT TTT TT<u>T</u> T)-3', <u>T</u> = GuNA[^{t}Bu]-T

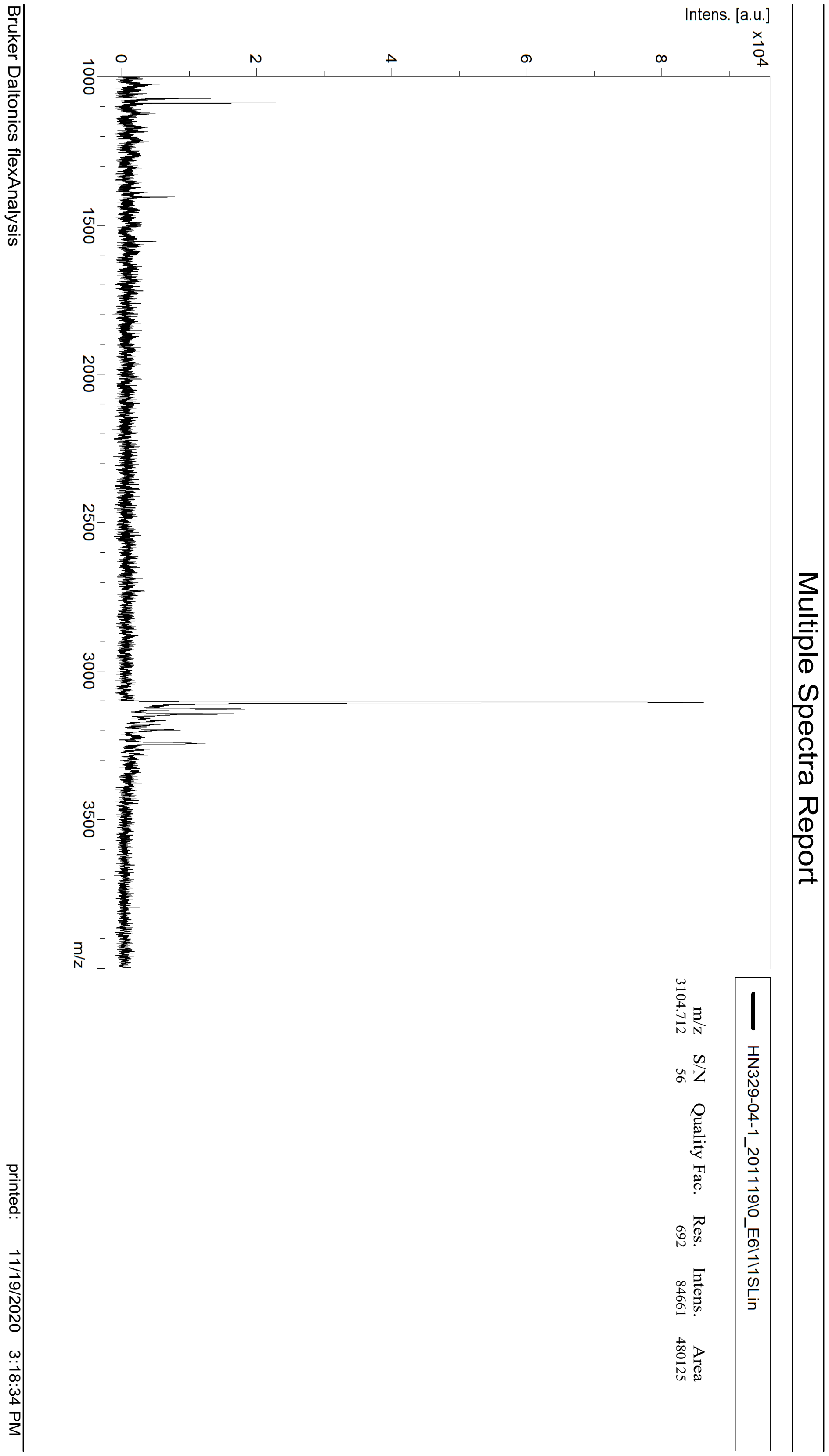

ODN11 5'-d(TTT TTT TT<u>T</u> T)-3', <u>T</u> = GuNA[Me,Me]-T

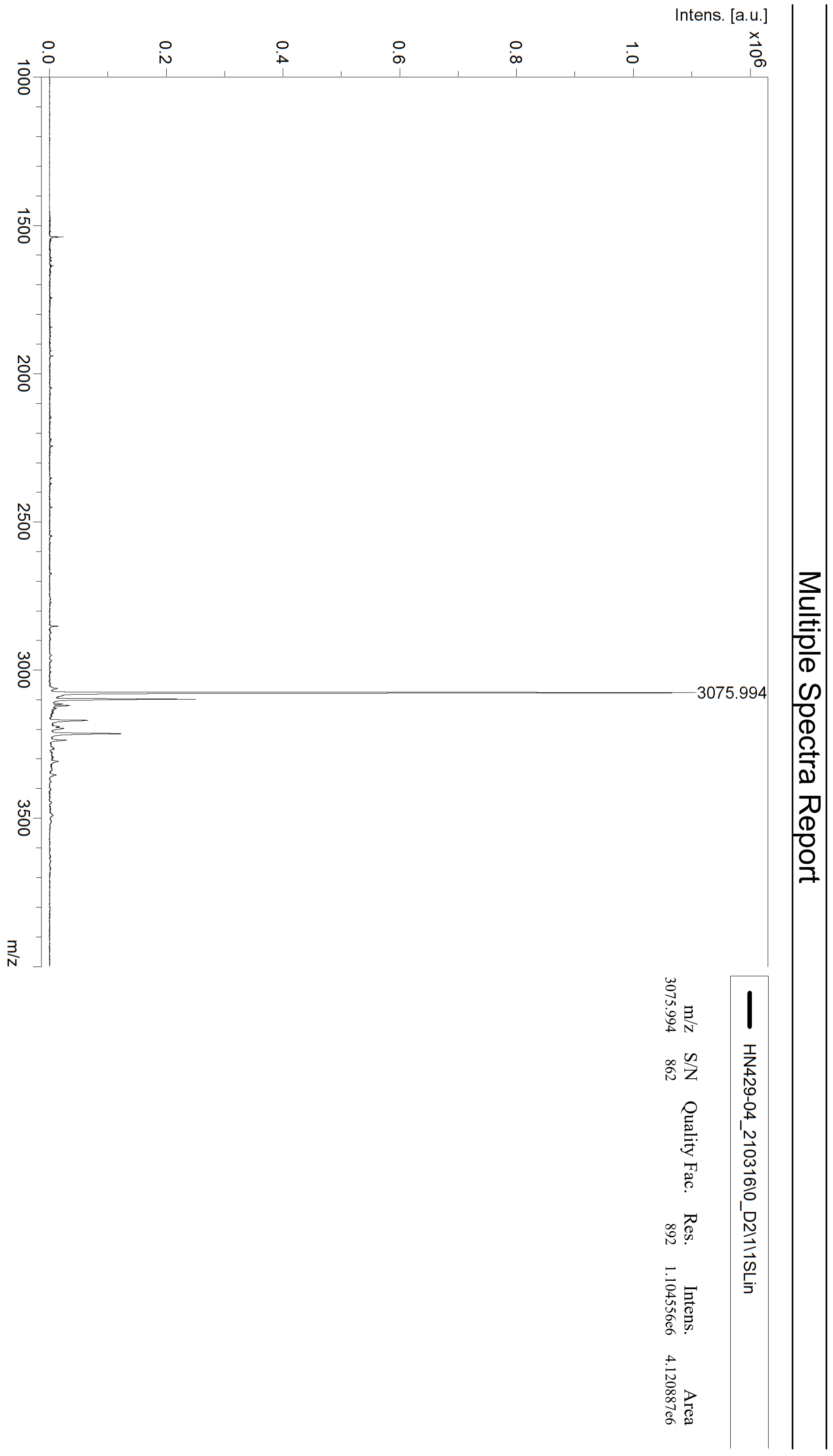

ODN12 5'-d(TTT TTT TT<u>T</u> T)-3', <u>T</u> = GuNA[Me,^{t}Bu]-T

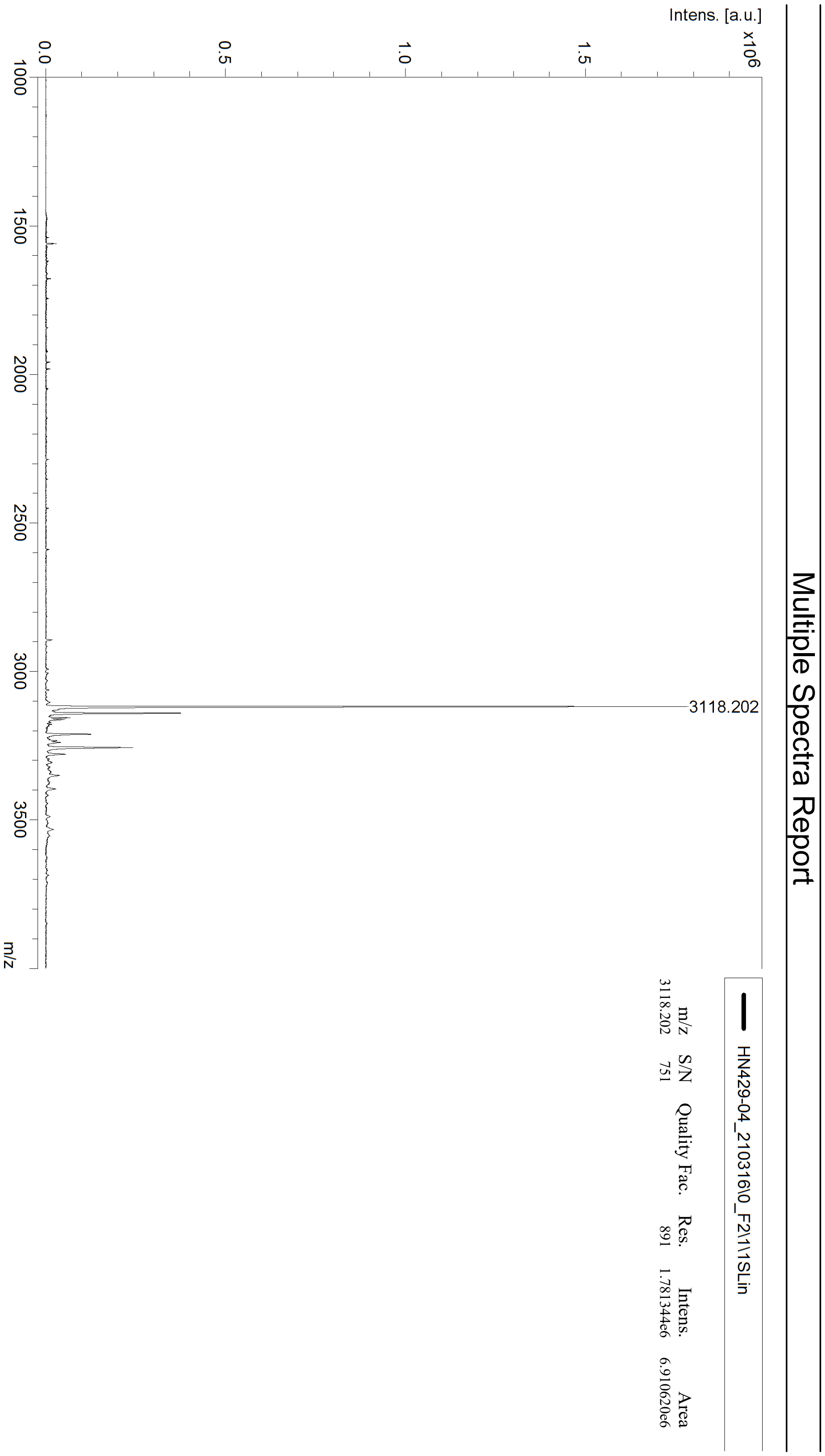

ODN13 5'-d(GCG TTA TTT GCT)-3', A = GuNA[Me,Me]-A

Multiple Spectra Report

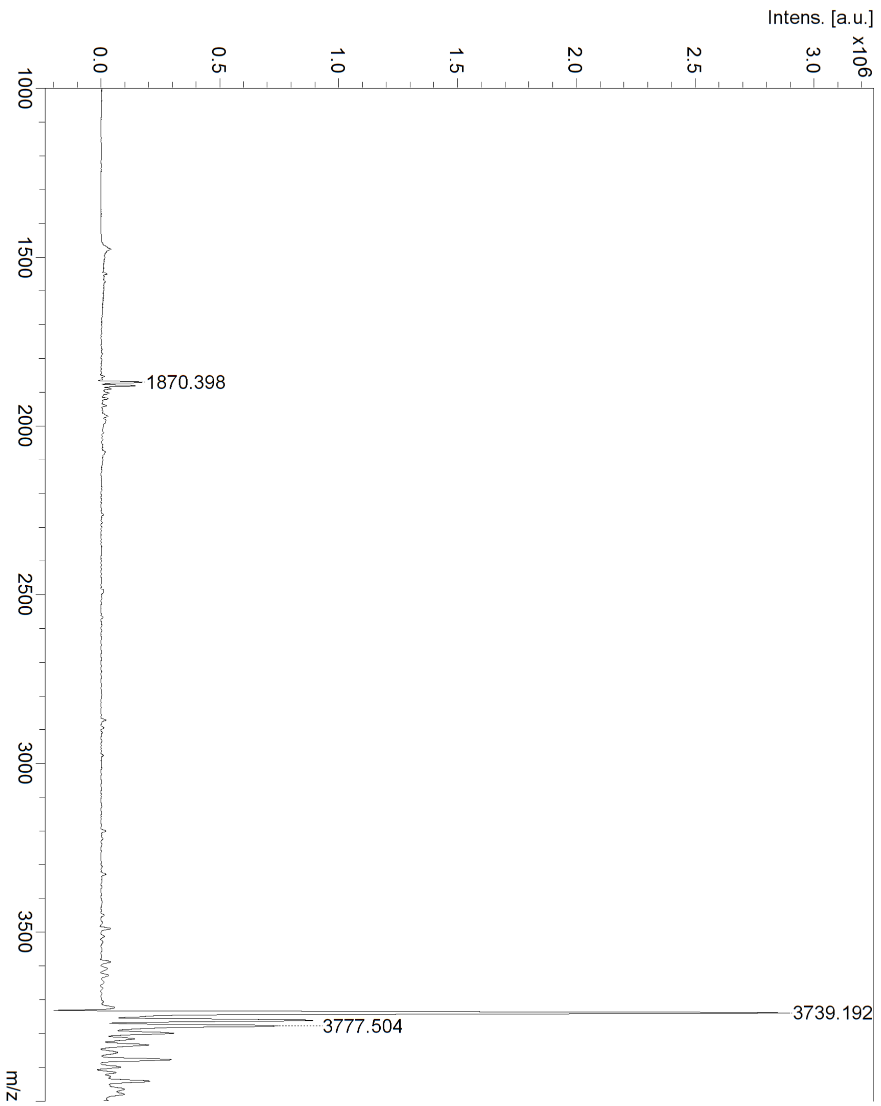

HN553-01\0_A10\1\1SLin

m/z	S/N	Quality Fac.	Res.	Intens.	Area
1870.398	46		360	176642	920845
1881.355	38		373	145309	792846
3739.192	579		502	2.89990e6	22.61724e6
3761.183	178		501	891780	7.419769e6
3777.504	149		460	745751	7.37127e6

ODN14 5'-d(GCG TTA TTT GCT)-3', A = GuNA[Me,^{t}Bu]-A

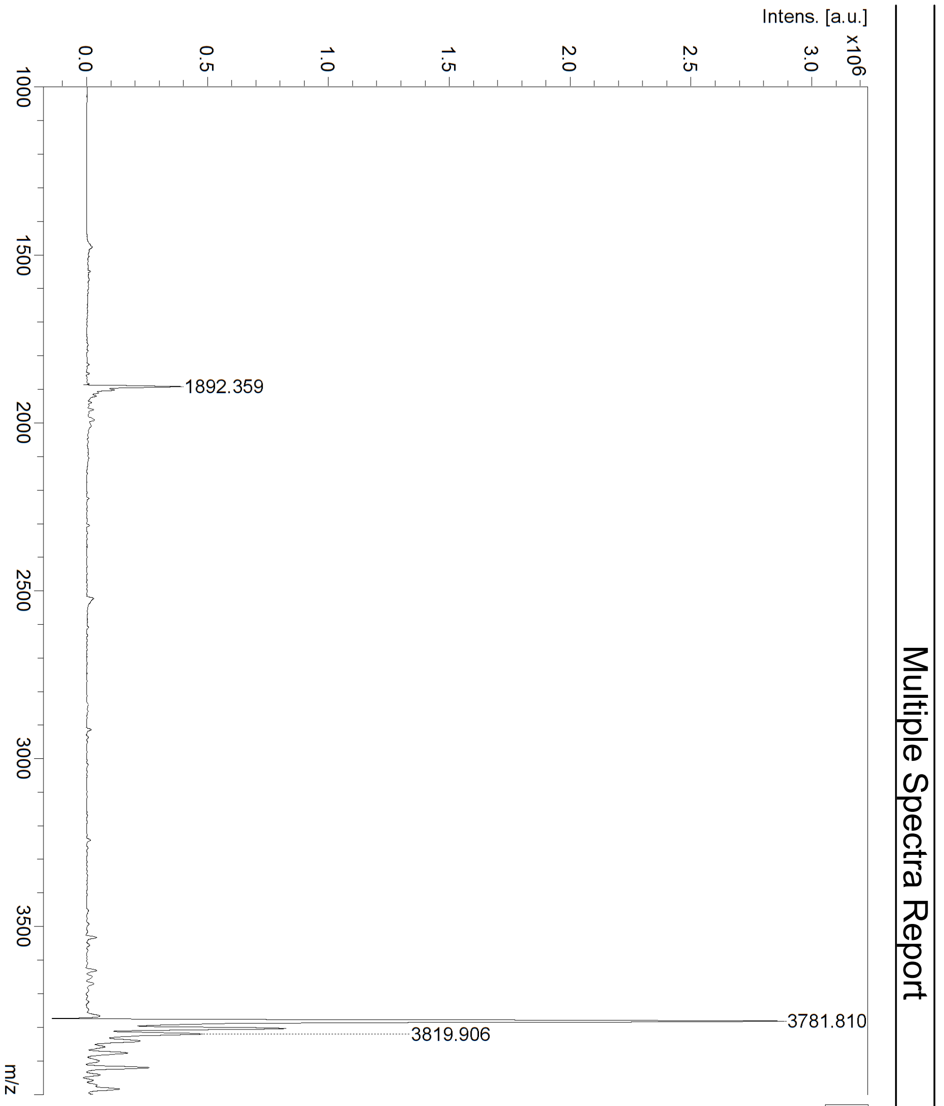

m/z	S/N	Quality Fac.	Res.	Intens.	Area
1892.359	127		361	392544	2.550677e6
3781.810	766		486	2.886274e6	25.683989e6
3803.573	219		501	825678	7.853429e6
3819.906	127		461	479003	5.298789e6

ODN15 5'-d(GCG TTG TTT GCT)-3', G = GuNA[Me,Me]-G

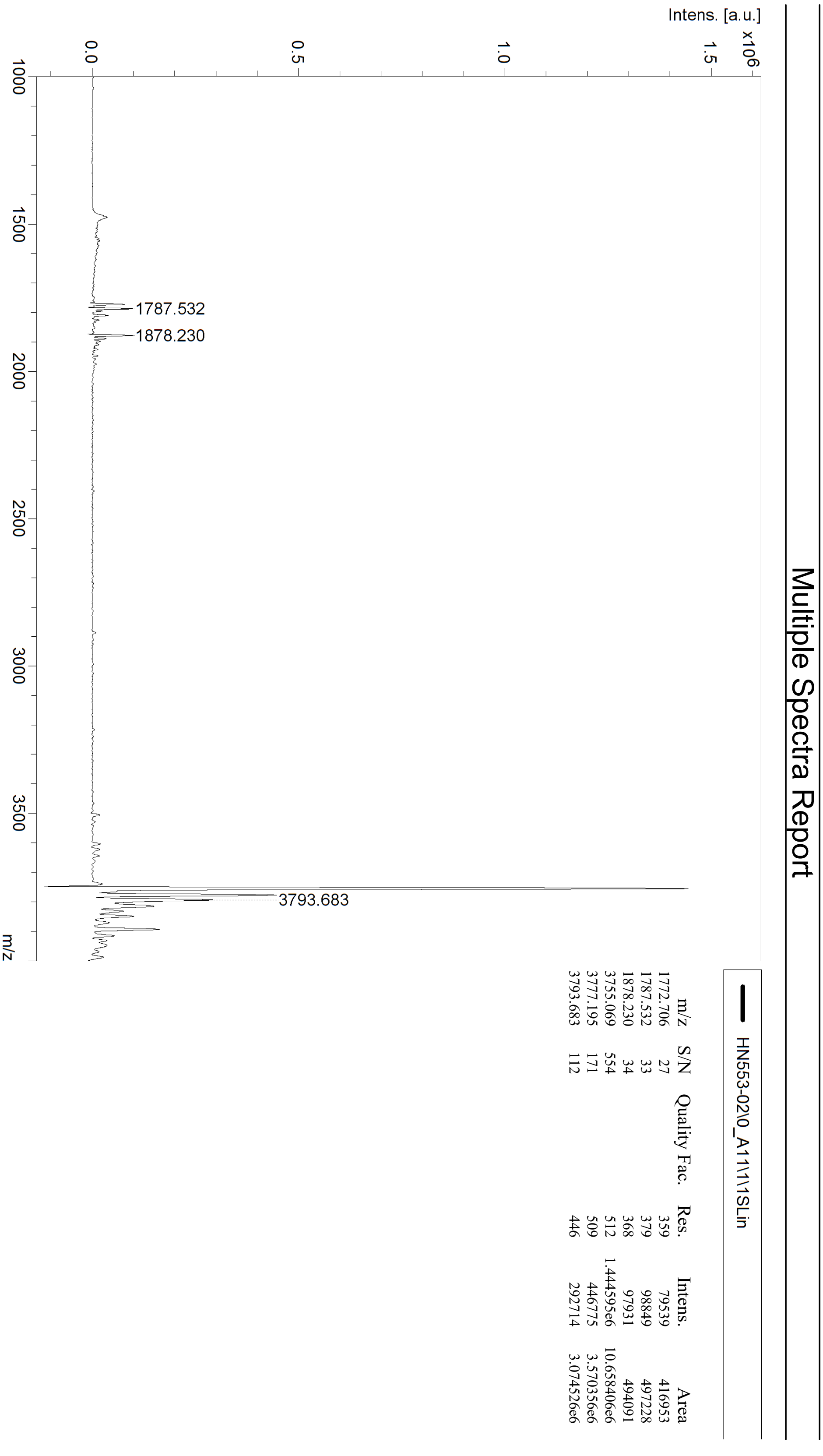

ODN16 5'-d(GCG TT<u>G</u> TTT GCT)-3', <u>G</u> = GuNA[Me,^{t}Bu]-G

Multiple Spectra Report

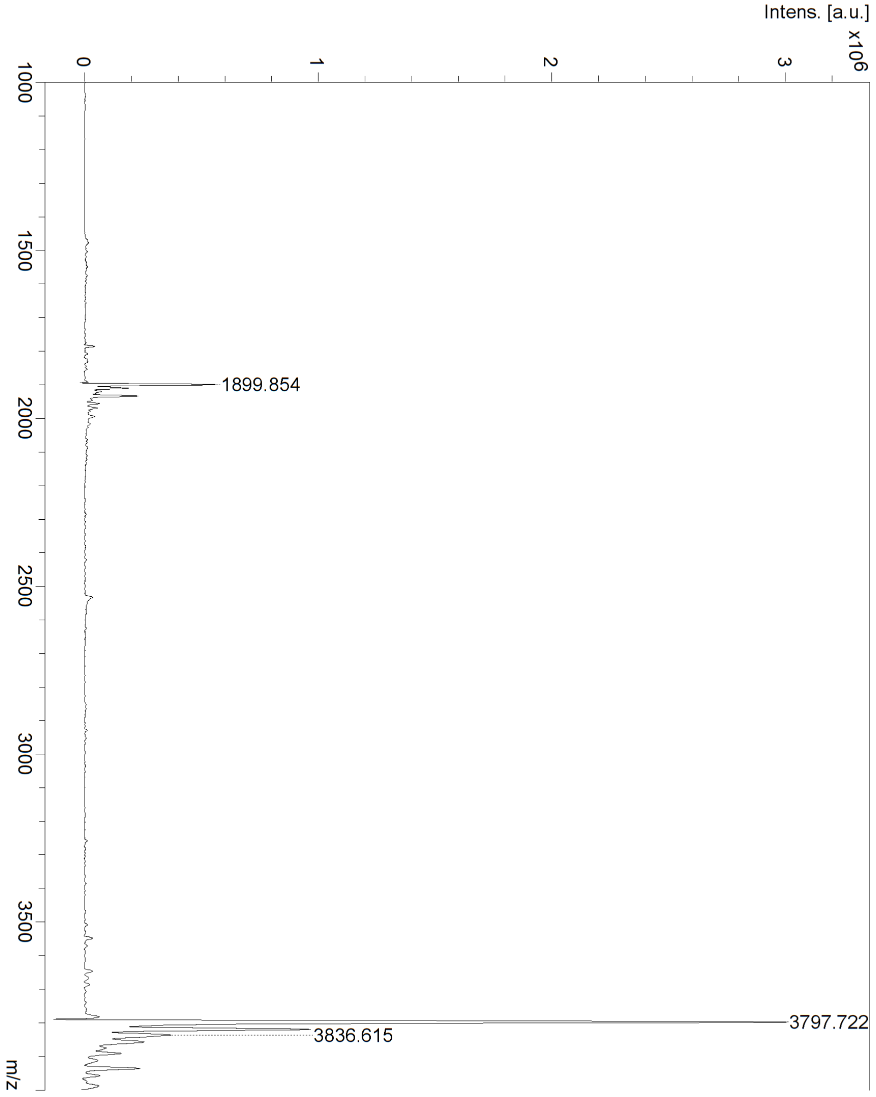

HN553-05\0_B12\1\1SLin

m/z	S/N	Quality Fac.	Res.	Intens.	Area
1899.854	99		368	571049	3.180600e6
3797.722	673		483	3.004384e6	26.384714e6
3819.541	217		498	968630	9.204290e6
3836.615	83		340	369009	4.956042e6

ODN17 5'-d(GCG TT$\underline{^{m}C}$ TTT GCT)-3', $\underline{^{m}C}$ = GuNA[Me,Me]-^{m}C

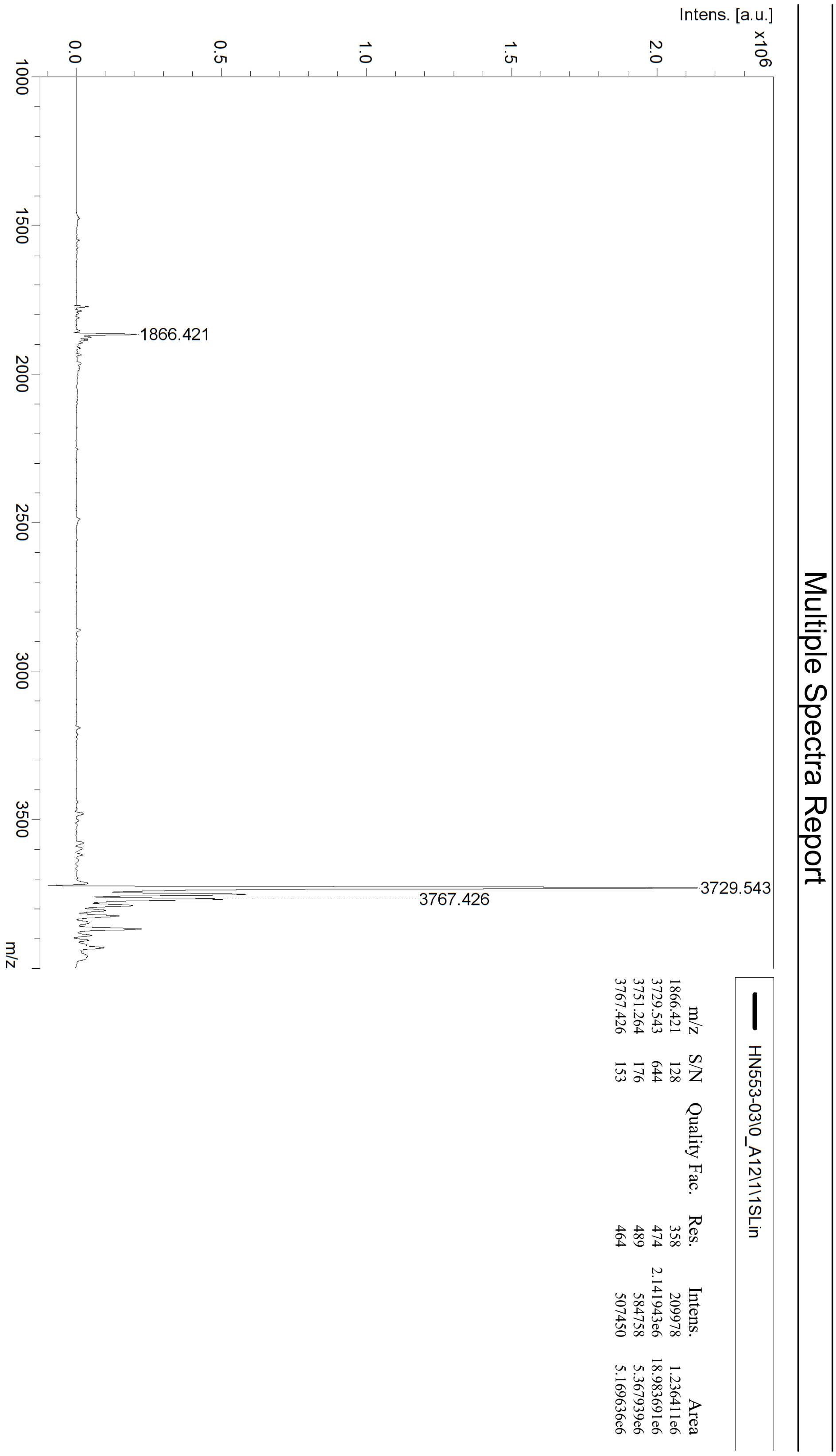

ODN18 5'-d(GCG TT$\underline{^{m}C}$ TTT GCT)-3', $\underline{^{m}C}$ = GuNA[Me,^{t}Bu]-^{m}C

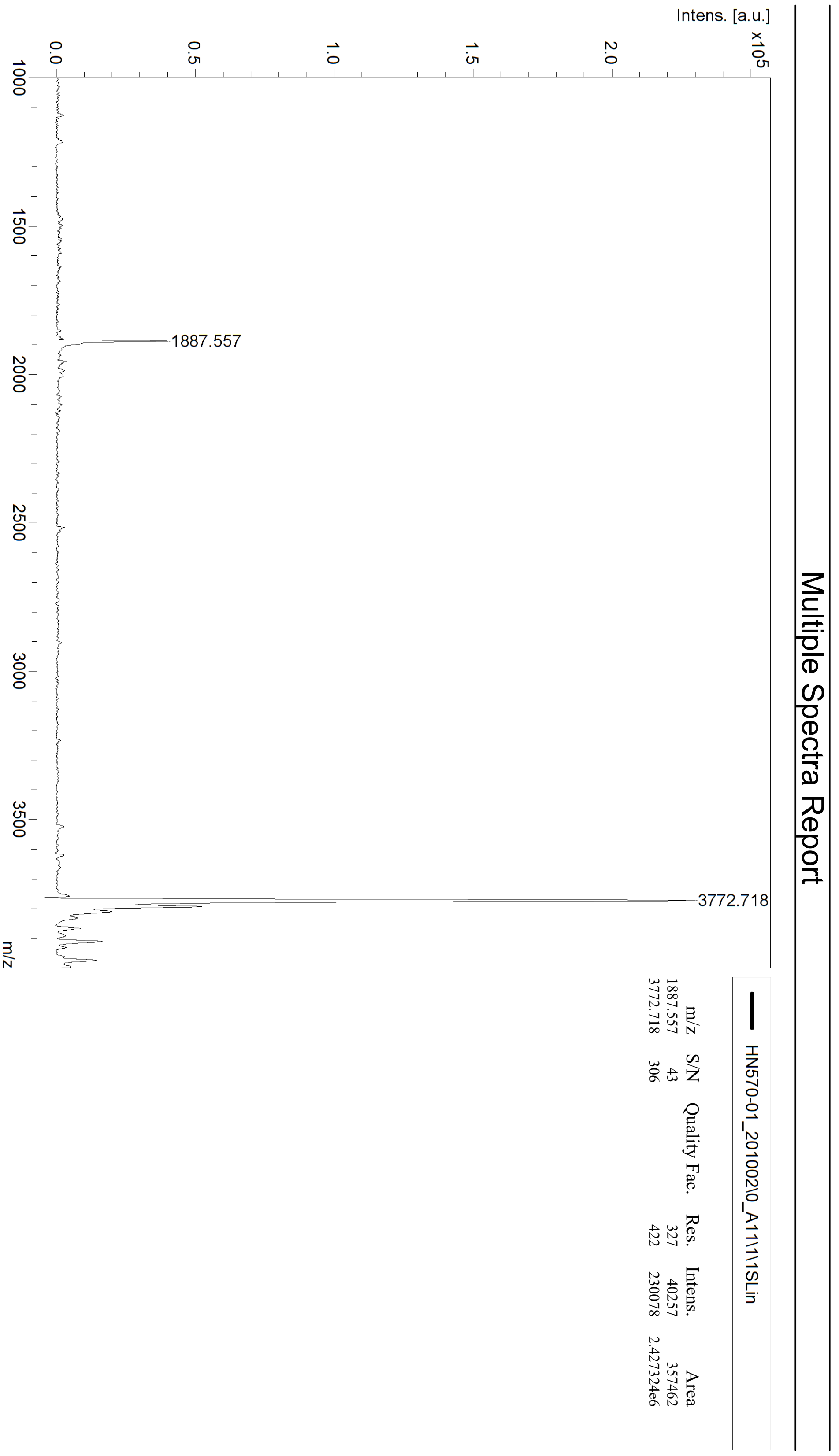

ODN24 5'-d(GCG TA<u>T</u> ACG C)-3', <u>T</u> = GuNA[Me,Me]-T

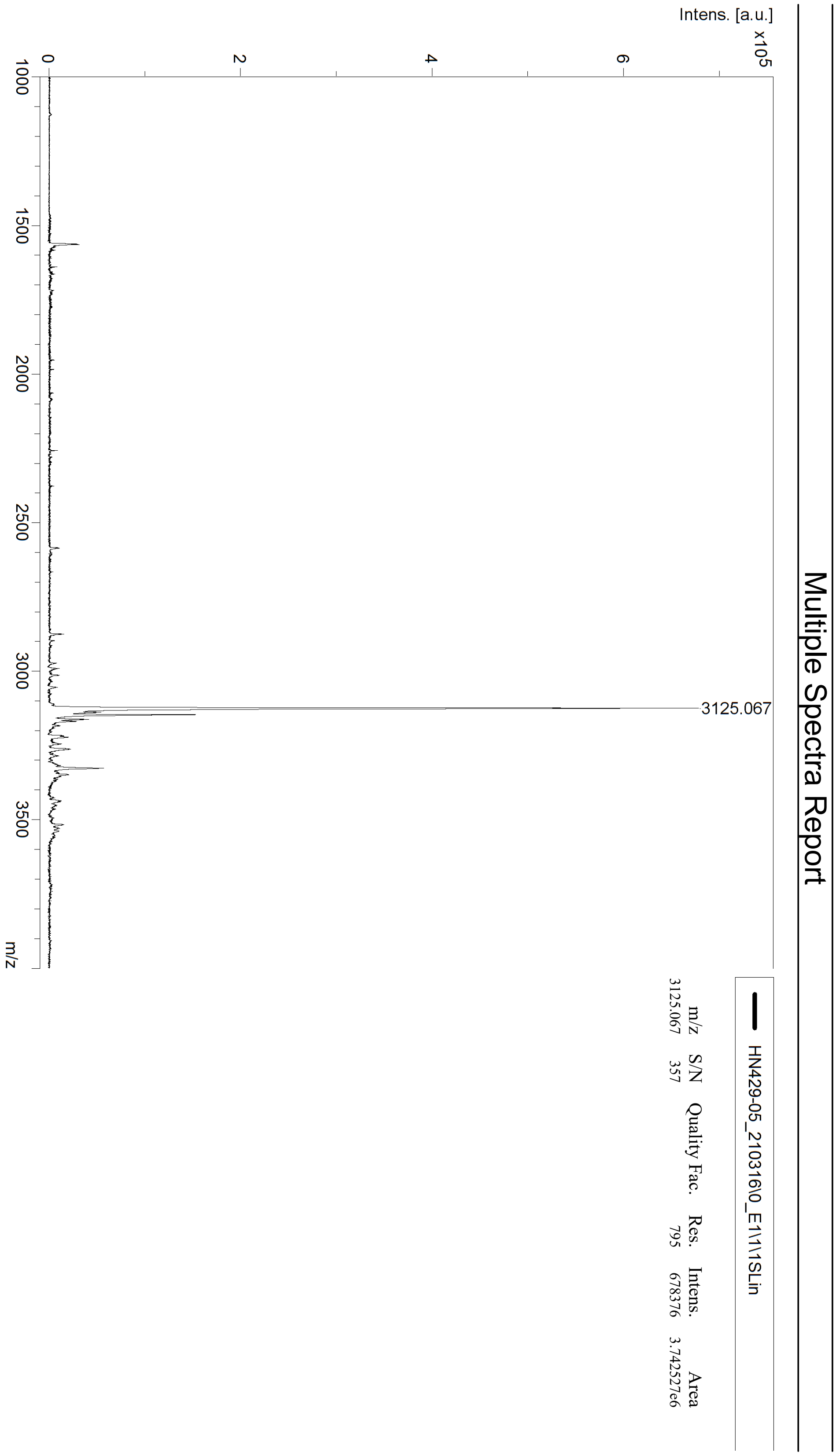

ODN25 5'-d(GCG TAT ACG C)-3', T = GuNA[Me,^{t}Bu]-T

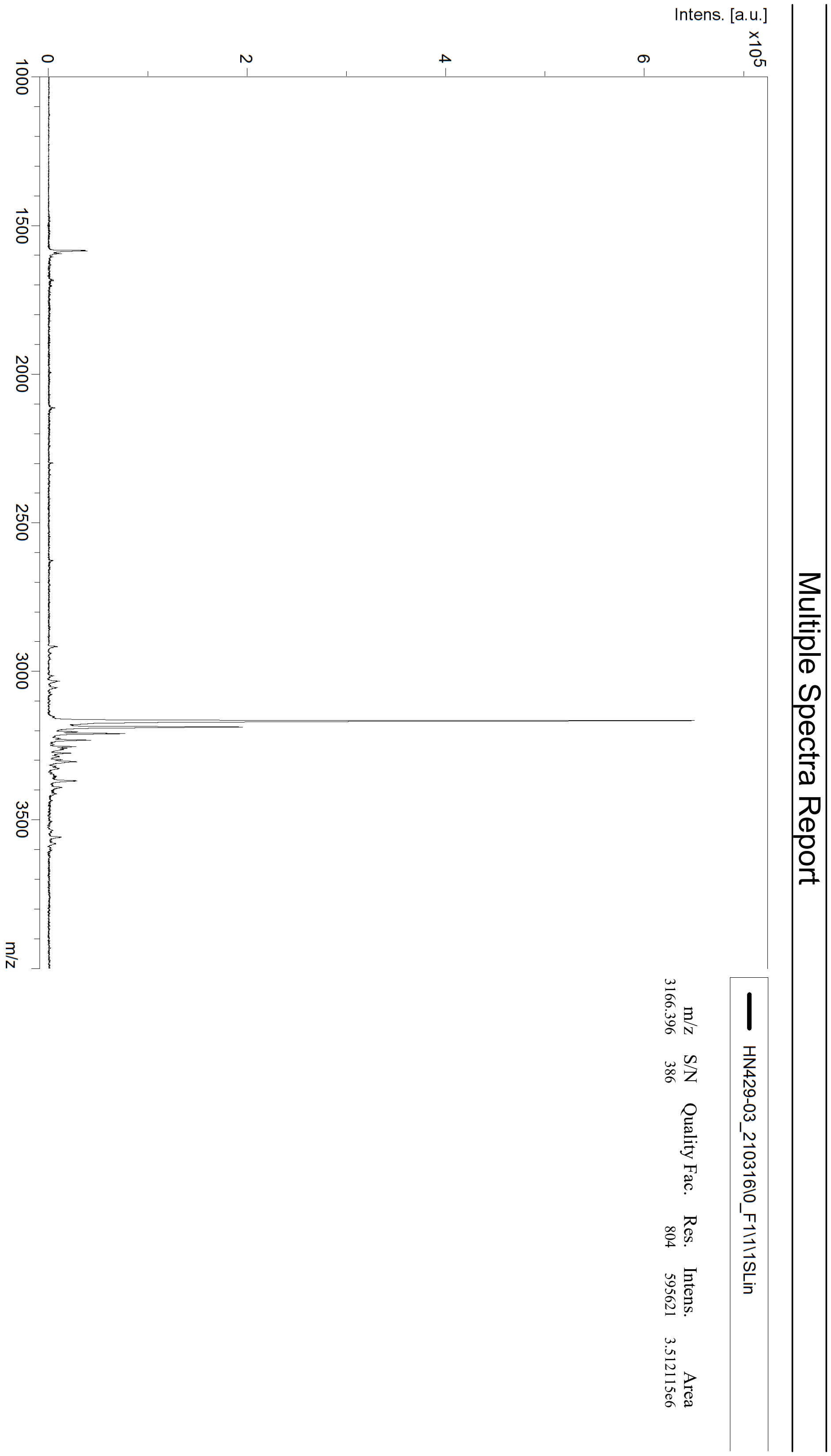

ODN26 5'-d(GCG T̲AT ACG C)-3', T̲ = GuNA[Me,Me]-T

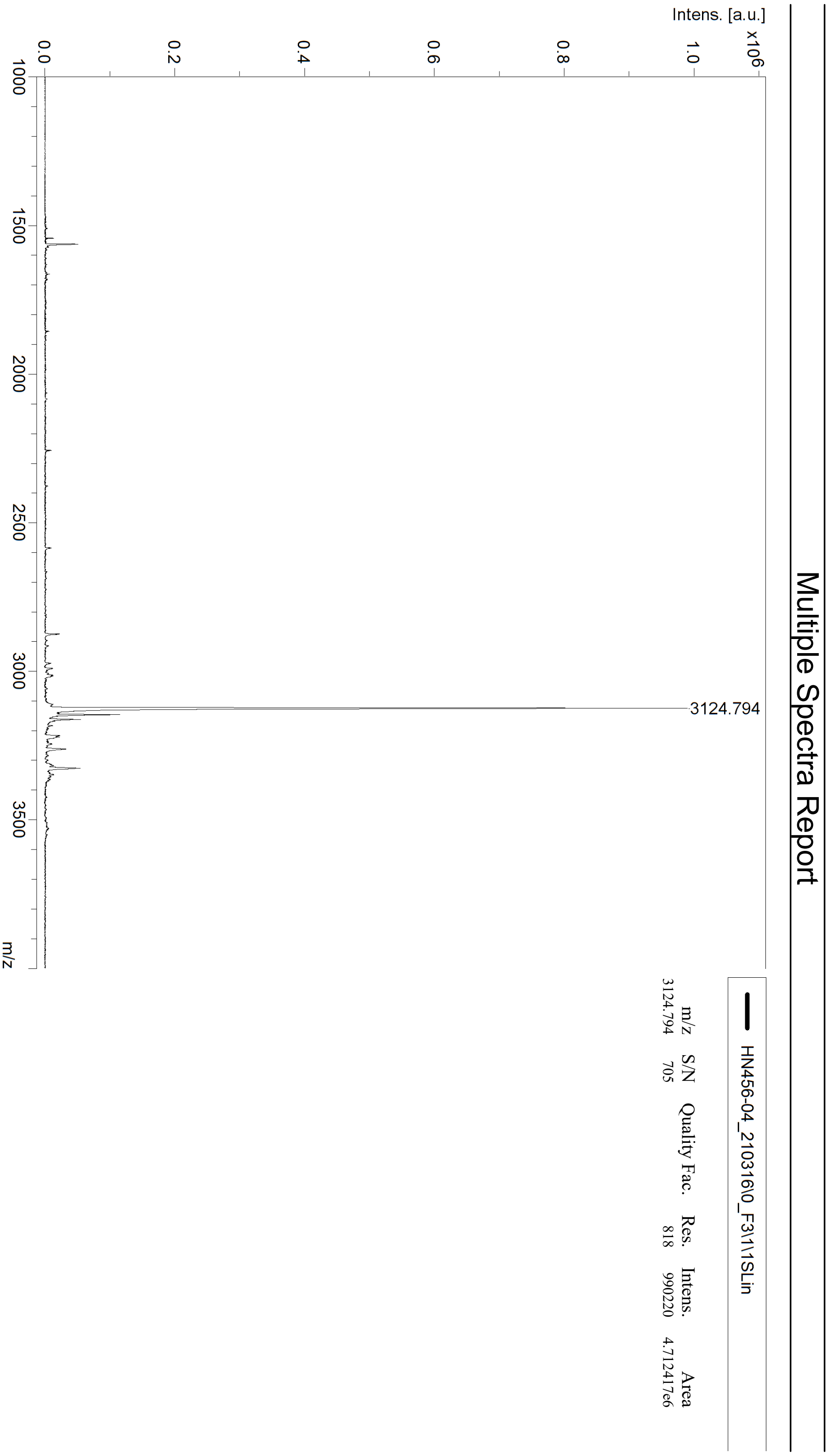

ODN27 5'-d(GCG $\underline{T}$AT ACG C)-3', $\underline{T}$ = GuNA[Me,^{t}Bu]-T

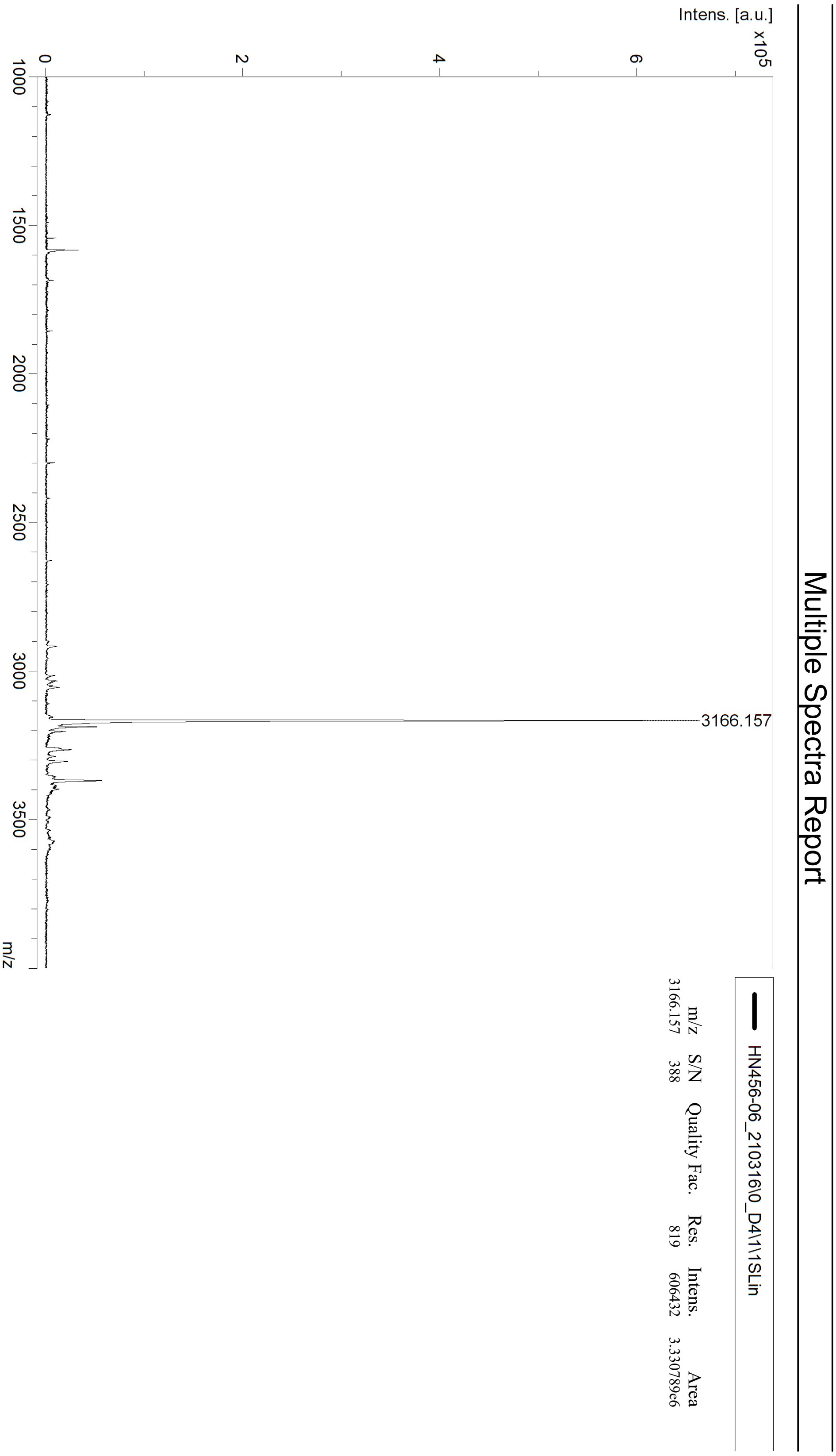

ODN28 5'-d(G<u>T</u>G ^{Br}UAC AC)-3', <u>T</u> = GuNA[Me,Me]-T

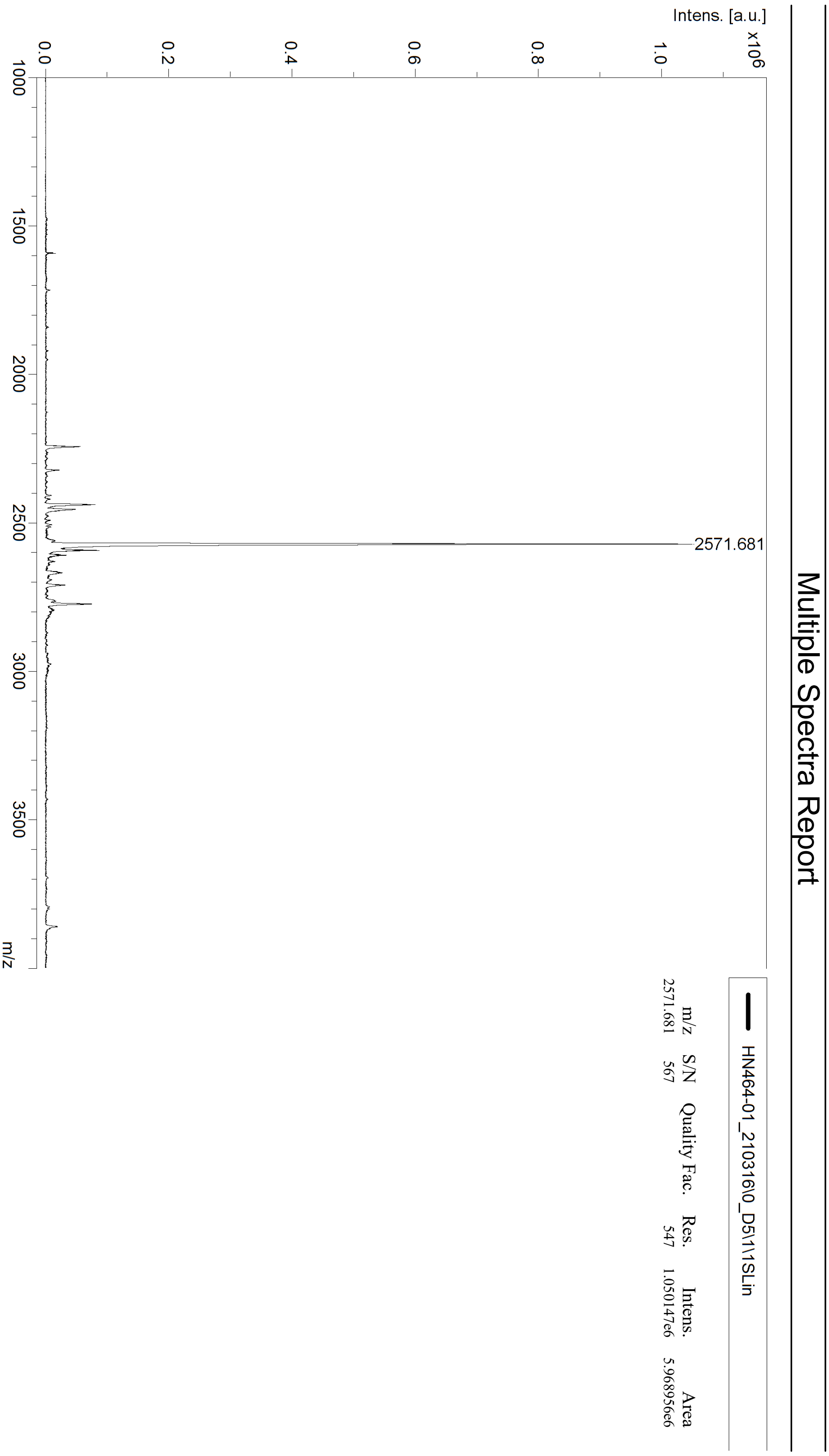

ODN29 5'-d(G<u>T</u>G ^{Br}UAC AC)-3', <u>T</u> = GuNA[Me,^{t}Bu]-T

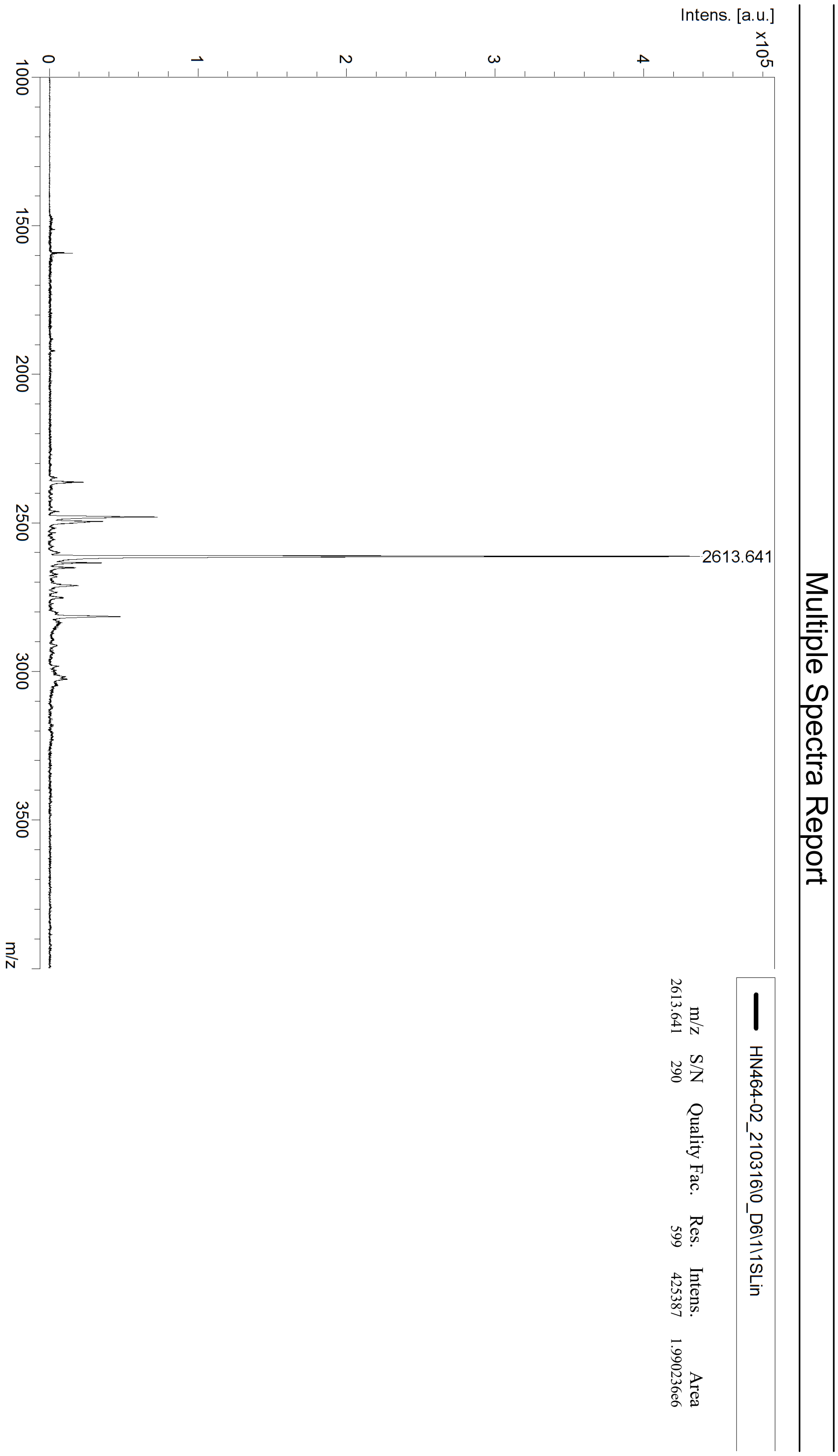

ODN30 5'-d(GTG ^{Br}UA^{m}<u>C</u> AC)-3', ^{m}<u>C</u> = GuNA[Me,Me]-^{m}C

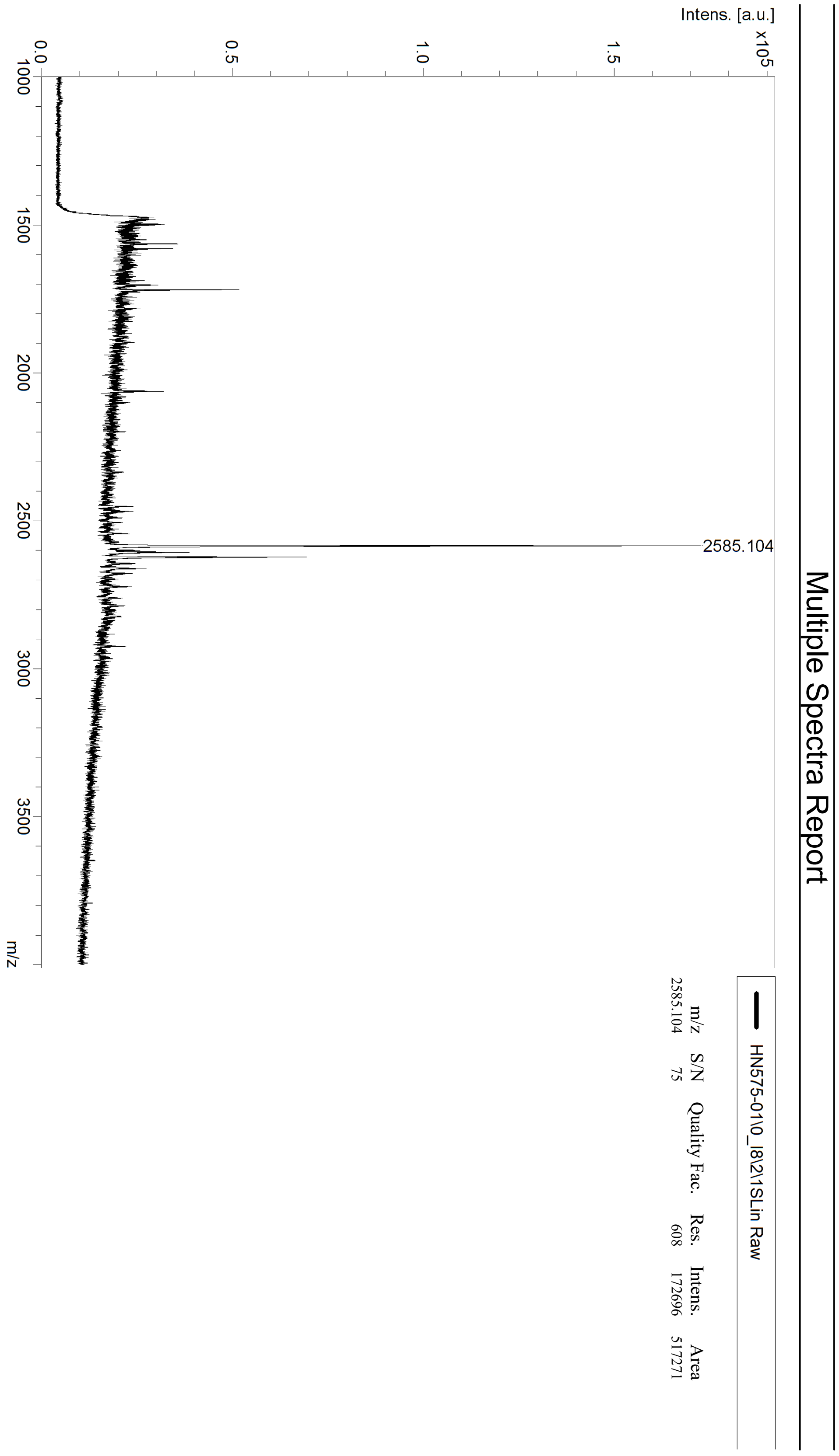

ODN31 5'-d(GTG ^{Br}UA$\underline{^{m}C}$ AC)-3', $\underline{^{m}C}$ = GuNA[Me,^{t}Bu]-^{m}C

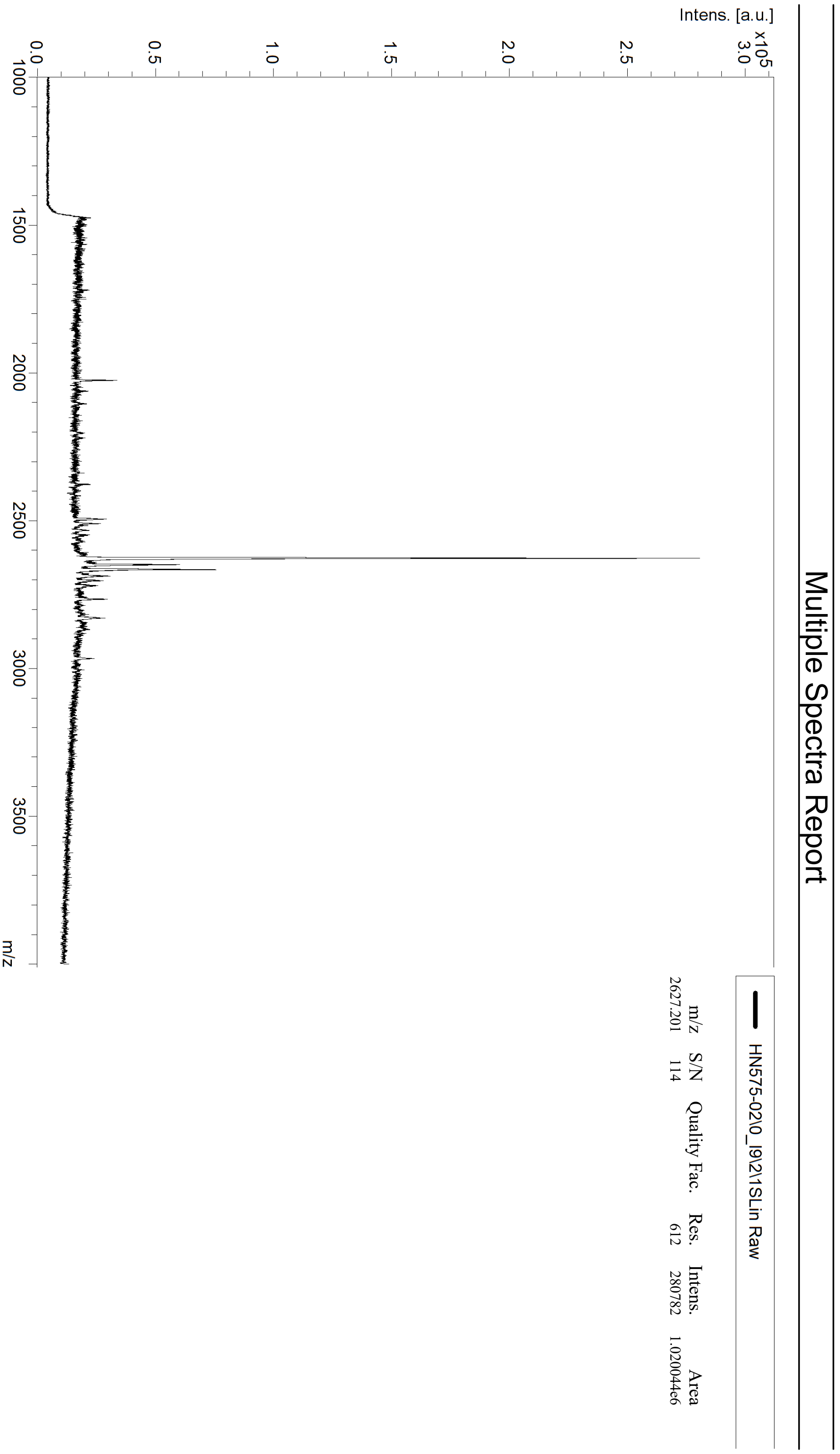

ODN32 5'-d(GTG ^{Br}UAC A̲C)-3', A̲ = GuNA[Me,Me]-A

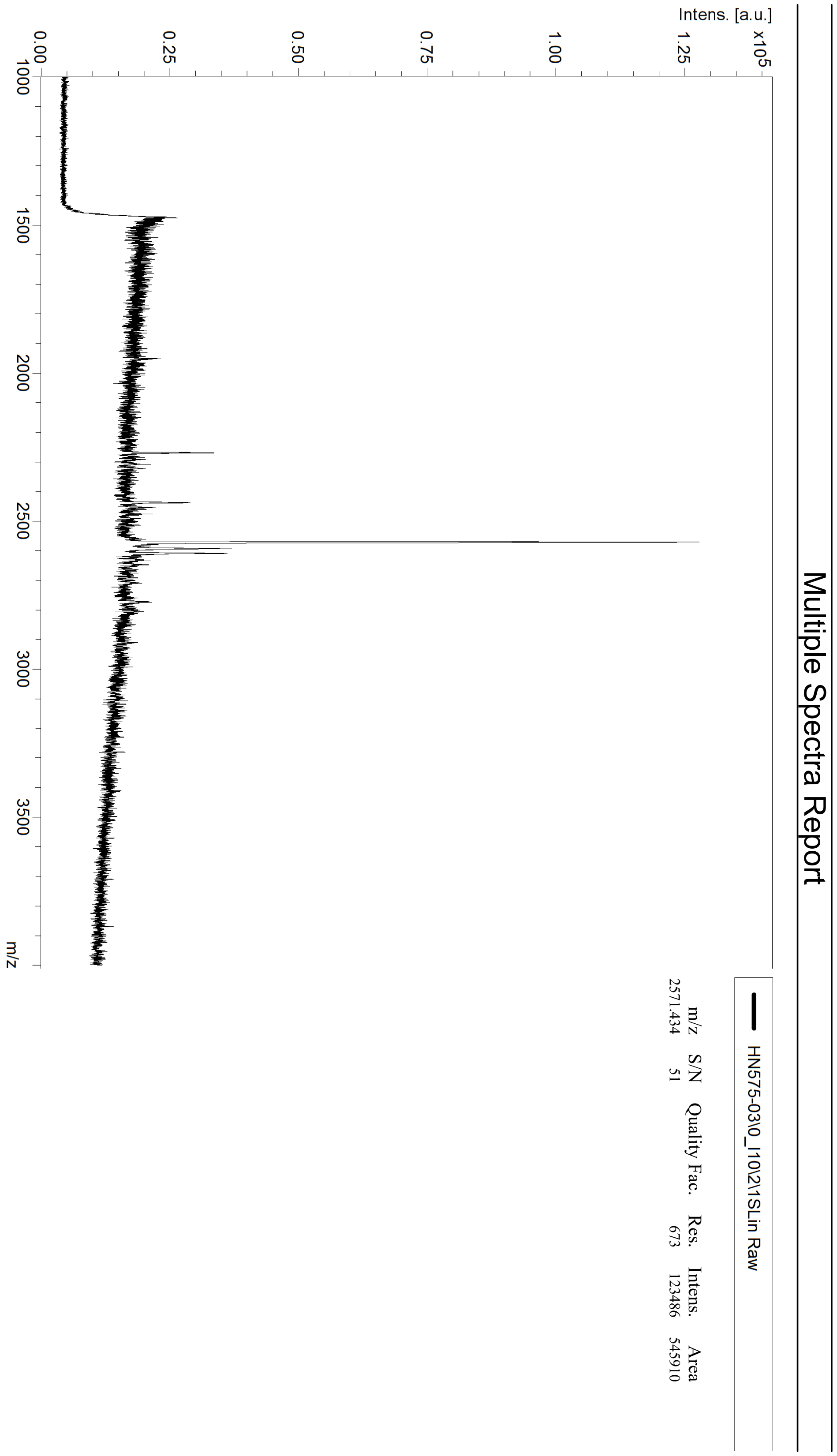

ODN33 5'-d(GTG ^{Br}UAC A̲C)-3', A̲ = GuNA[Me,^{t}Bu]-A

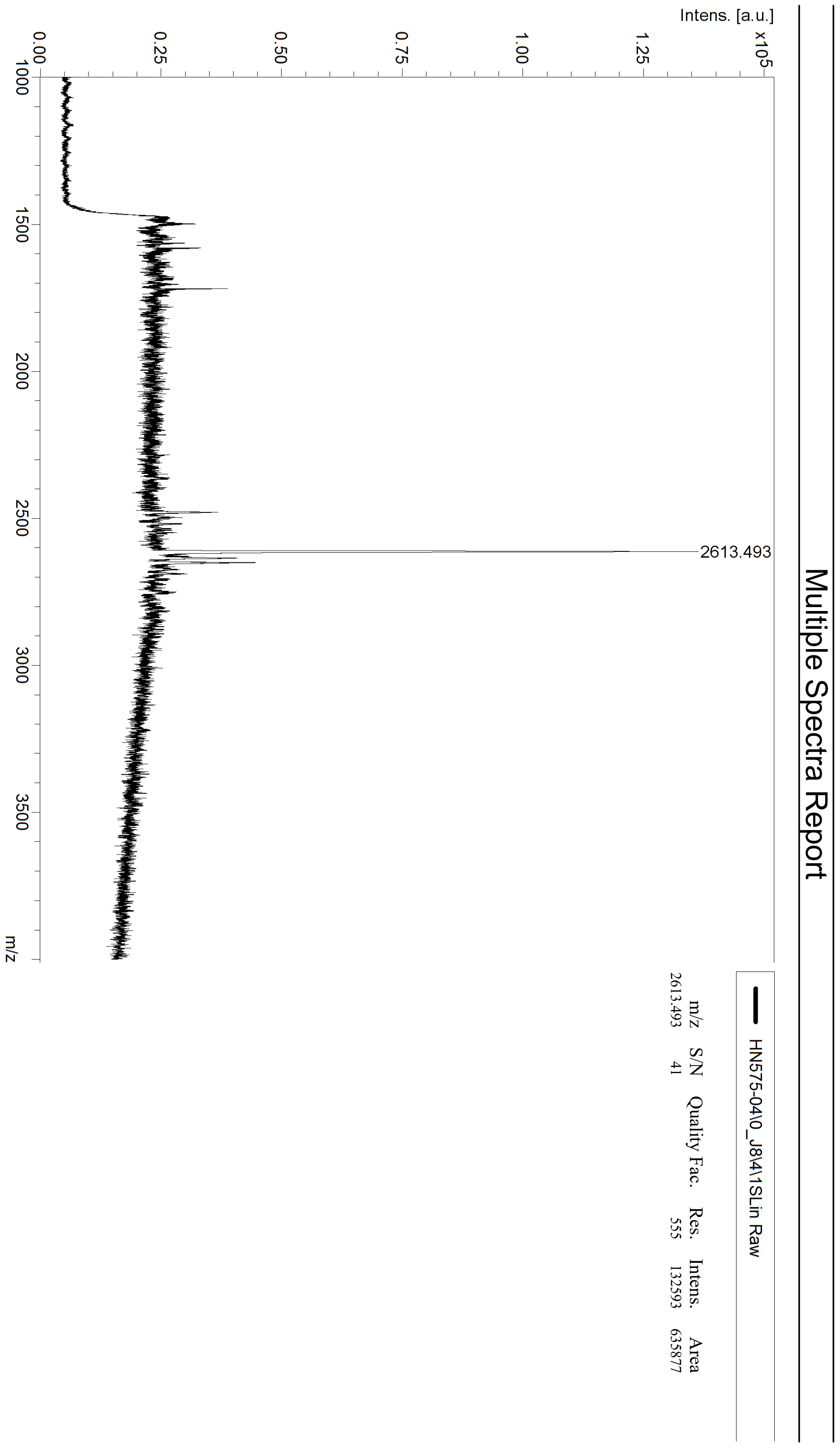

5. UV-melting curves

Normalized UV melting curves for the duplexes formed between GuNA[R]-modified oligonucleotides and the complementally RNA strand. The sequences are 5'-d(GCG TTT TTT GCT)-3' and 5'-r(AGC AAA AAA CGC)-3'. T indicates the position of modifications.

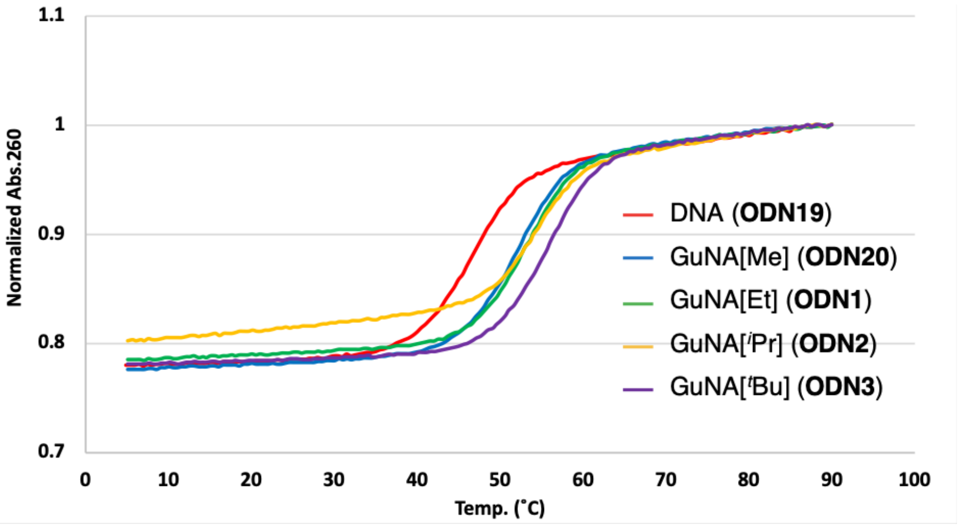

Normalized UV melting curves for the duplexes formed between GuNA[R,R]-modified oligonucleotides and the complementally RNA strand. The sequences are 5'-d(GCG TTT TTT GCT)-3' and 5'-r(AGC AAA AAA CGC)-3'. T indicates the position of modifications.

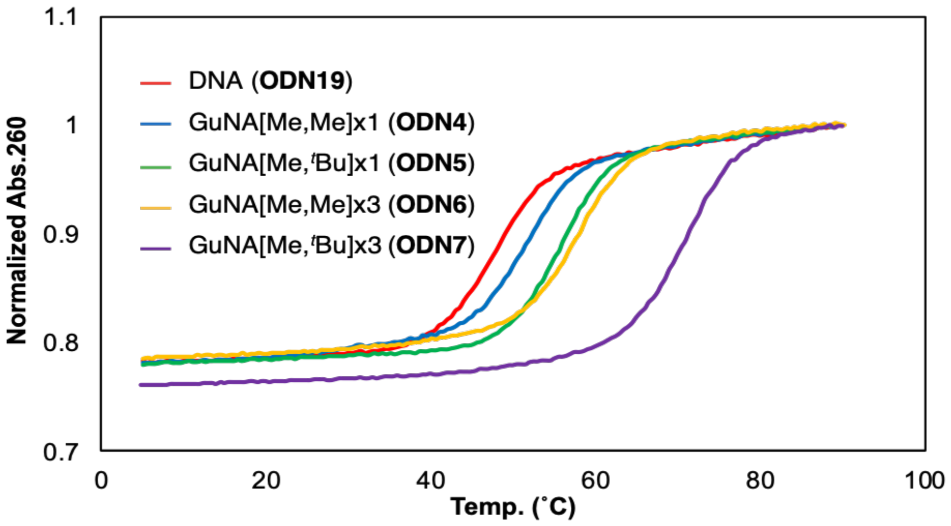

Normalized UV melting curves for the duplexes formed between GuNA[R]- modified oligonucleotides and the complementally DNA strand. The sequences are 5'-d(GCG TTT TTT GCT)-3' and 5'-d(AGC AAA AAA CGC)-3'. T indicates the position of modifications.

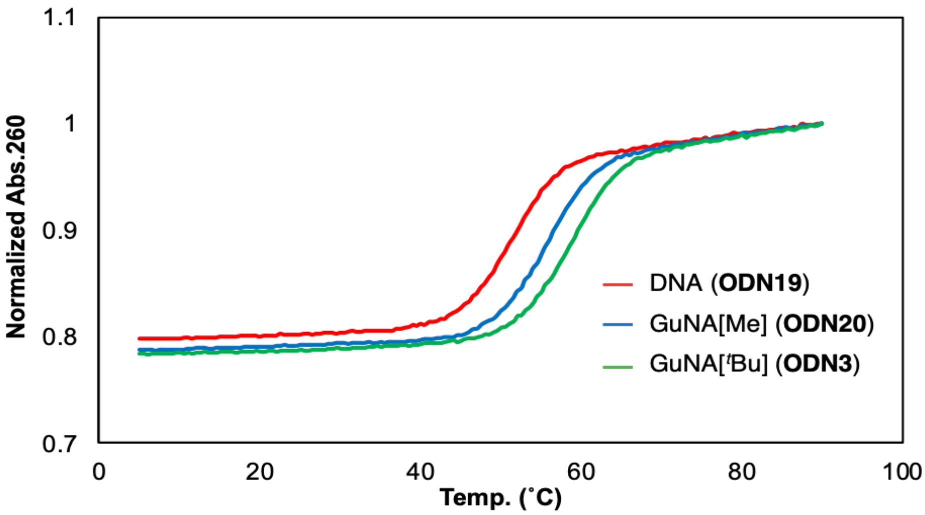

Normalized UV melting curves for the duplexes formed between GuNA[R,R]-modified oligonucleotides and the complementally DNA strand. The sequences are 5'-d(GCG TTT TTT GCT)-3' and 5'-d(AGC AAA AAA CGC)-3'. T indicates the position of modifications.

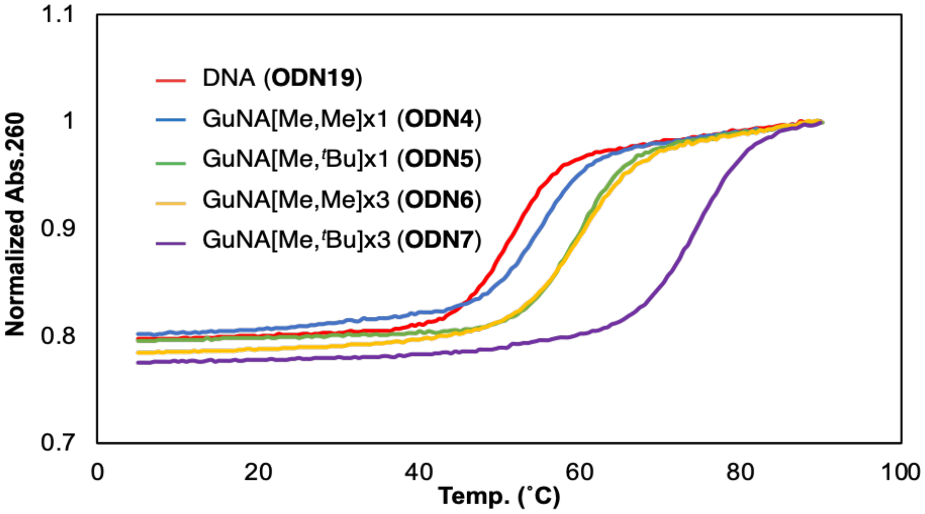

Normalized UV melting curves for the duplexes formed between GuNA[Me]-modified oligonucleotides (**ODN20**) and the complementally and mismatch RNA strands. The sequences are 5'-d(GCG TTT TTT GCT)-3' and 5'-r(AGC AAA YAA CGC)-3'. T indicates the position of modifications. Y = A, G, C, U.

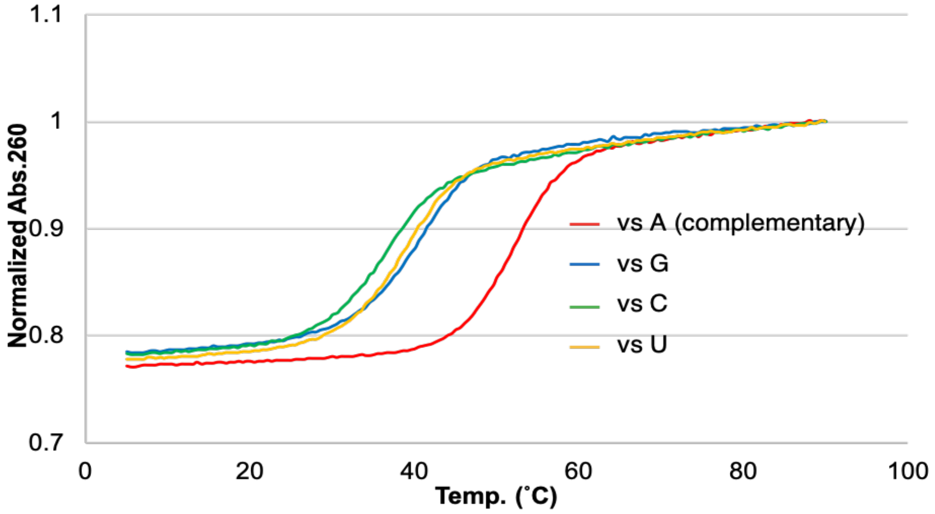

Normalized UV melting curves for the duplexes formed between GuNA[^{t}Bu]-modified oligonucleotides (**ODN3**) and the complementally and mismatch RNA strands. The sequences are 5'-d(GCG TTT TTT GCT)-3' and 5'-r(AGC AAA YAA CGC)-3'. T indicates the position of modifications. Y = A, G, C, U.

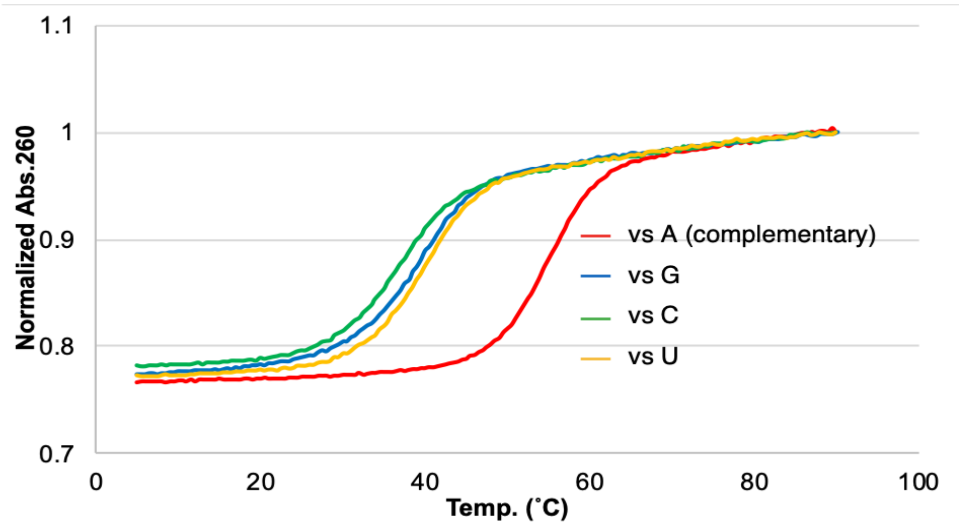

Normalized UV melting curves for the duplexes formed between GuNA[Me,^{t}Bu]-modified oligonucleotides (**ODN5**) and the complementally and mismatch RNA strands. The sequences are 5'-d(GCG TT<u>T</u> TTT GCT)-3' and 5'-r(AGC AAA <u>Y</u>AA CGC)-3'. <u>T</u> indicates the position of modifications. <u>Y</u> = A, G, C, U.

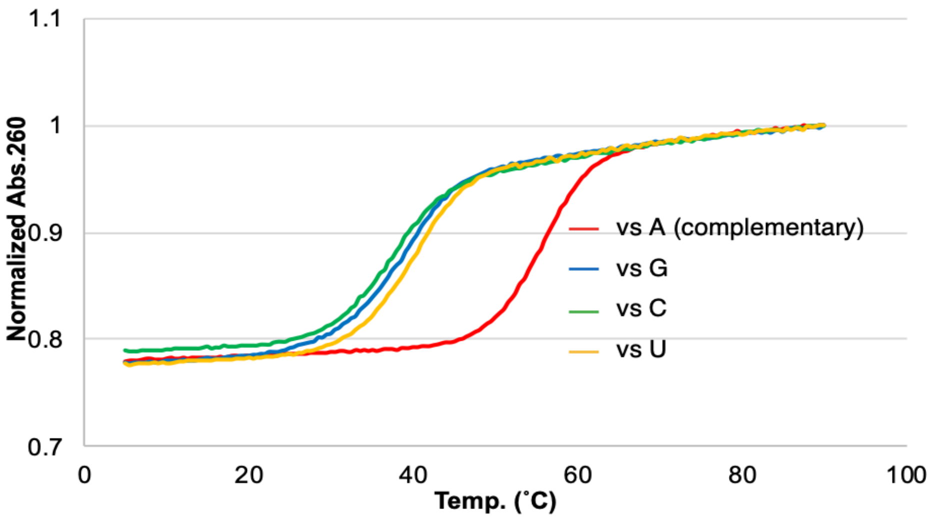

Normalized UV melting curves for the duplexes formed between GuNA[Me]-modified oligonucleotides (**ODN20**) and the complementally and mismatch DNA strands. The sequences are 5'-d(GCG TT<u>T</u> TTT GCT)-3' and 5'-d(AGC AAA <u>Y</u>AA CGC)-3'. <u>T</u> indicates the position of modifications. <u>Y</u> = A, G, C, T.

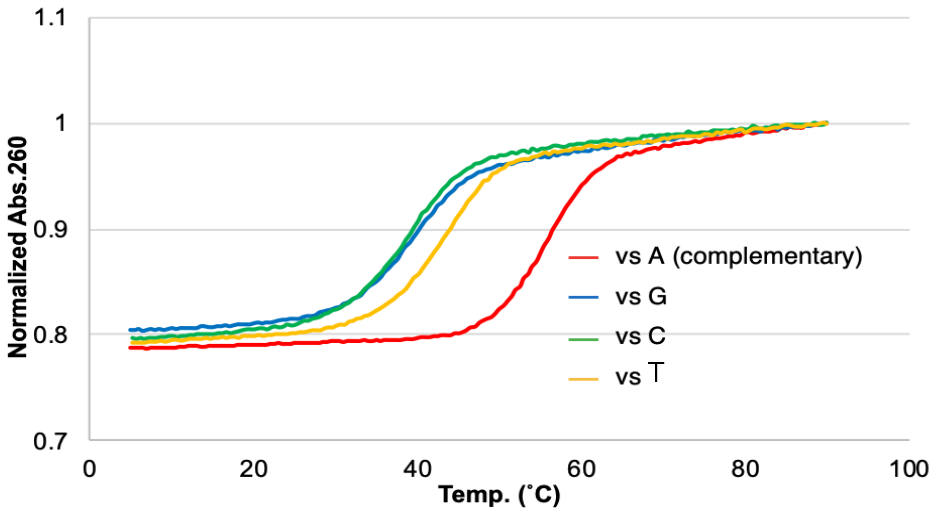

Normalized UV melting curves for the duplexes formed between GuNA[^{t}Bu]-modified oligonucleotides (**ODN3**) and the complementally and mismatch DNA strands. The sequences are 5'-d(GCG TT<u>T</u> TTT GCT)-3' and 5'-d(AGC AAA <u>Y</u>AA CGC)-3'. <u>T</u> indicates the position of modifications. <u>Y</u> = A, G, C, T.

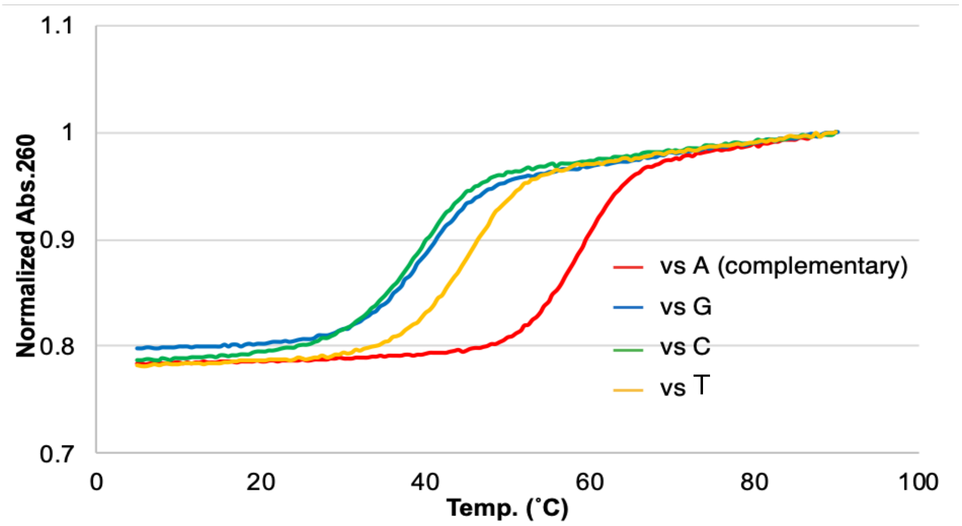

Normalized UV melting curves for the duplexes formed between GuNA[Me,^{t}Bu]-modified oligonucleotides (**ODN5**) and the complementally and mismatch DNA strands. The sequences are 5'-d(GCG TT<u>T</u> TTT GCT)-3' and 5'-d(AGC AAA <u>Y</u>AA CGC)-3'. <u>T</u> indicates the position of modifications. <u>Y</u> = A, G, C, T.

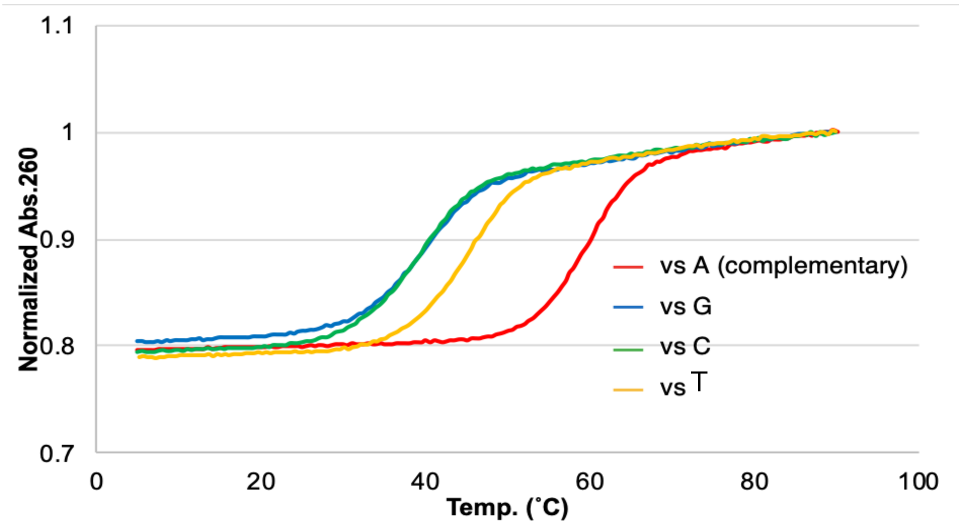

Normalized UV melting curves for the duplexes formed between GuNA[R,R]-modified oligonucleotides and the complementally RNA strand. The sequences are 5'-d(GCG TTA TTT GCT)-3' and 5'-r(AGC AAA UAA CGC)-3'. A indicates the position of modifications.

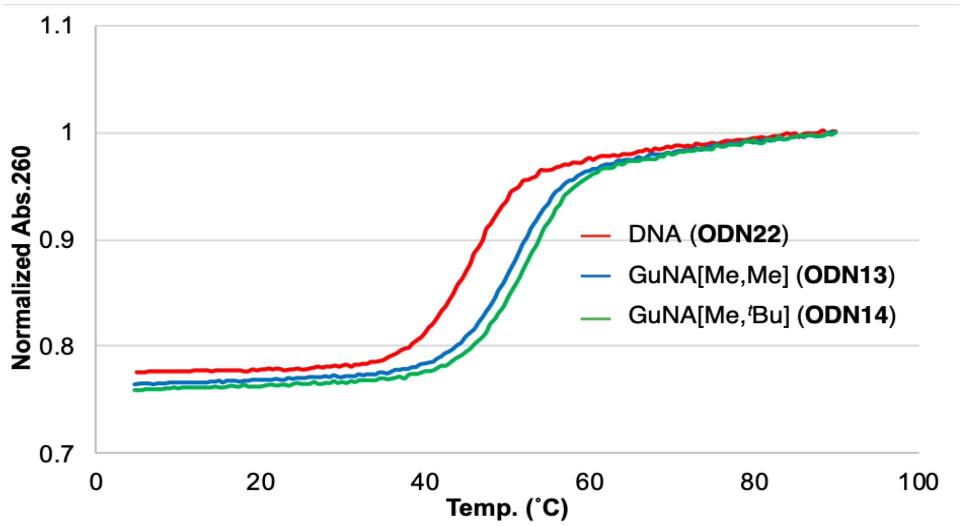

Normalized UV melting curves for the duplexes formed between GuNA[R,R]-modified oligonucleotides and the complementally DNA strand. The sequences are 5'-d(GCG TTA TTT GCT)-3' and 5'-d(AGC AAA TAA CGC)-3'. A indicates the position of modifications.

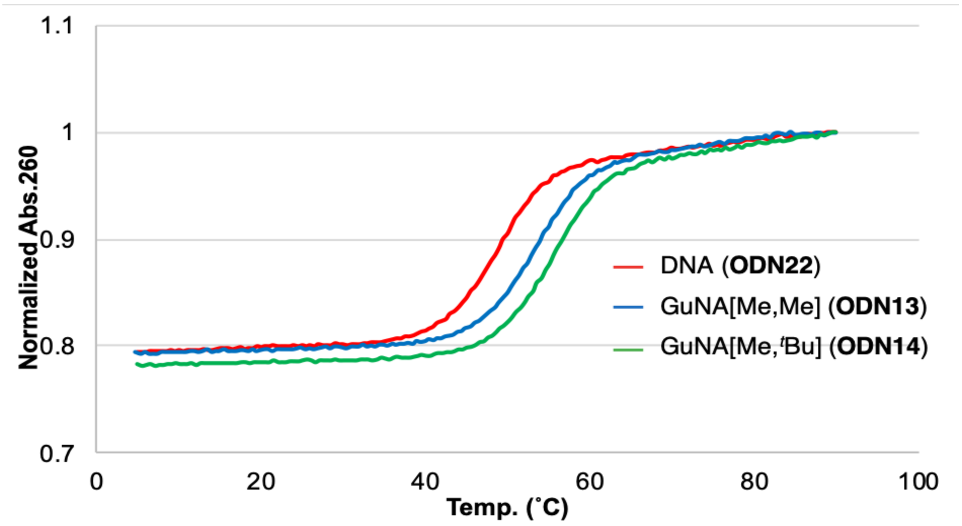

Normalized UV melting curves for the duplexes formed between GuNA[R,R]-modified oligonucleotides and the complementally RNA strand. The sequences are 5'-d(GCG TT$^{m}\underline{C}$ TTT GCT)-3' and 5'-r(AGC AAA GAA CGC)-3'. $^{m}\underline{C}$ indicates the position of modifications.

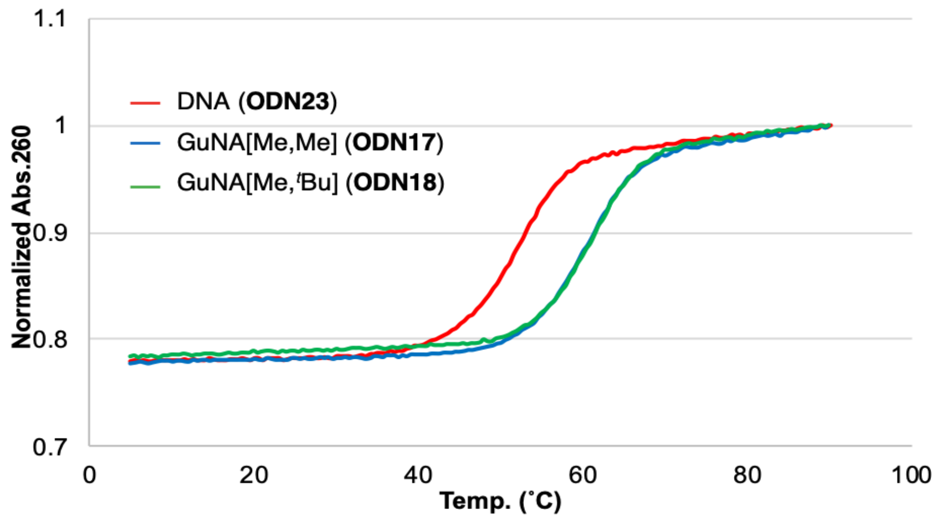

Normalized UV melting curves for the duplexes formed between GuNA[R,R]-modified oligonucleotides and the complementally DNA strand. The sequences are 5'-d(GCG TT$^{m}\underline{C}$ TTT GCT)-3' and 5'-d(AGC AAA GAA CGC)-3'. $^{m}\underline{C}$ indicates the position of modifications.

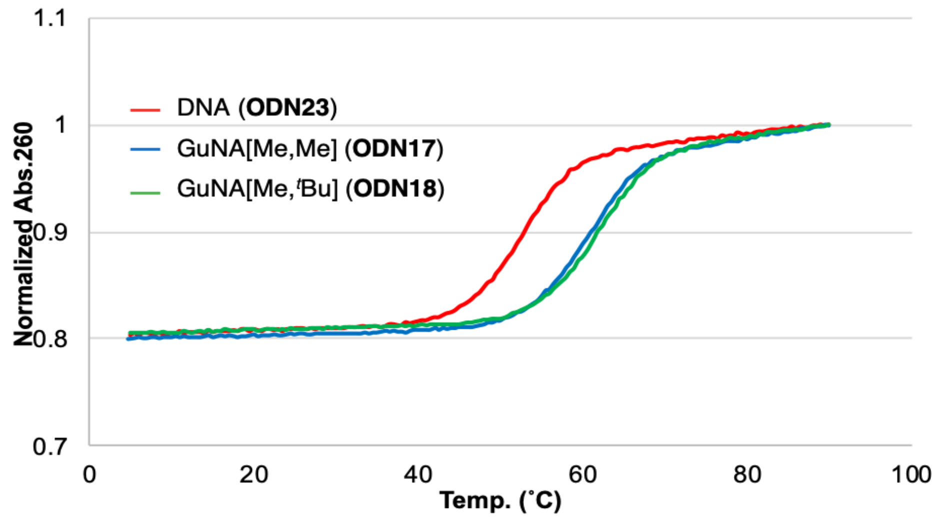

Normalized UV melting curves for the duplexes formed by self-complementary 10-mer oligonucleotides (**ODN24–ODN27**, **ODN34**). The sequence is 5'-d(GCG TA<u>T</u> ACG C)-3'. <u>T</u> indicates the position of modifications.

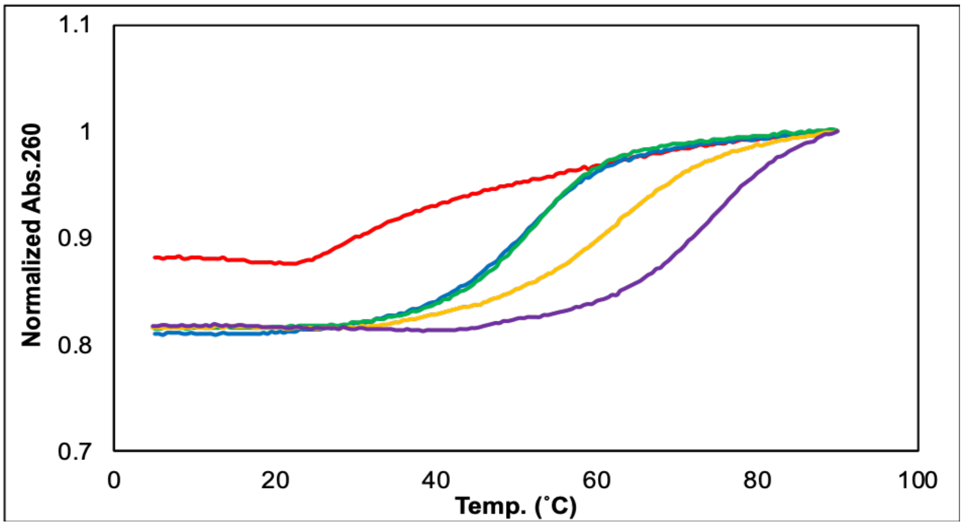

Normalized UV melting curves for the duplexes formed by self-complementary 8-mer oligonucleotides having GuNA[R,R]-T (**ODN28**, **ODN29**).

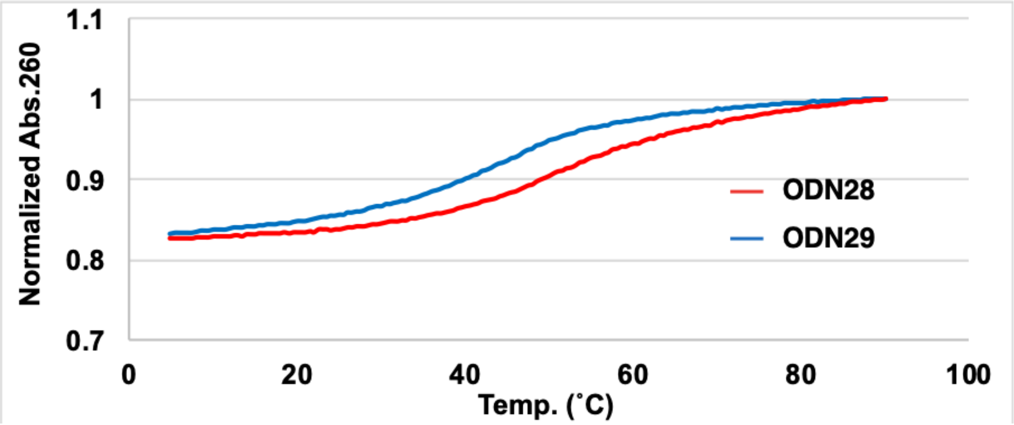

6. X-ray crystallography data

Table S6. X-ray data collection and structure refinement statistics for **ODN25**.

**ODN25**	5'-d(GCG TA<u>T</u> ACG C)-3', <u>T</u> = GuNA[Me,^{t}Bu]
PDB ID	8HIS
Crystal data	
Space group	$P2_12_12_1$
Lattice constants, Å	a = 25.07, b = 44.36, c = 45.19
Lattice angles, °	α=β=γ=90
Data collection	
X-ray source	Rigaku MicroMax-007HF
Wavelength, Å	1.54178
Resolution [outer shell], Å	20.13–2.01 (2.07–2.01)
Unique reflections	3,652 (294)
Completeness [outer shell], %	99.7 (99.4)
R_{merge} [outer shell], %	4.2 (5.5)
I/σ(I) [outer shell]	55.1 (43.8)
Redundancy [outer shell]	6.5 (6.4)
Structure refinement	
R_{work}, %	17.8
R_{free}, %	22.8
R.m.s.d. bond length, Å	0.005
R.m.s.d. bond angles, º	1.911

Table S7. X-ray data collection and structure refinement statistics for **ODN28**.

**ODN28**	5'-d(GTG ^{Br}UAC AC)-3', T = GuNA[Me,Me]
PDB ID	8I50
Crystal data	
Space group	$P6_122$
Lattice constants, Å	$a = b = 32.27$, $c = 80.67$
Lattice angles, °	α=β= 90, γ=120
Data collection	
Beamline	BL44XU of SPring-8
Wavelength, Å	0.9199
Resolution [outer shell], Å	40.34–0.95 (0.97–0.95)
Unique reflections	16,392 (720)
Completeness [outer shell], %	99.5 (94.3)
R_{merge} [outer shell], %	4.2 (1.2)
I/σ(I) [outer shell]	34.6 (1.6)
Redundancy [outer shell]	18.3 (8.1)
Structure refinement	
R_{work}, %	16.8
R_{free}, %	19.4
R.m.s.d. bond length, Å	0.006
R.m.s.d. bond angles, º	2.134

Table S8. X-ray data collection and structure refinement statistics for **ODN29**.

**ODN29**	5'-d(G<u>T</u>G ^{Br}UAC AC)-3', <u>T</u> = GuNA[Me,^{t}Bu]
PDB ID	8HU5
Crystal data	
Space group	$P4_32_12$
Lattice constants, Å	*a* = *b* = 42.49, *c* = 24.32
Lattice angles, °	α=β=γ=90
Data collection	
Beamline	BL44XU of SPring-8
Wavelength, Å	0.9199
Resolution [outer shell], Å	21.10–0.93 (0.99–0.93)
Unique reflections	27,813 (3,969)
Completeness [outer shell], %	97.7 (86.6)
R_{merge} [outer shell], %	8.3 (23.7)
I/σ(I) [outer shell]	16.0 (3.9)
Redundancy [outer shell]	6.6 (3.1)
Structure refinement	
R_{work}, %	19.2
R_{free}, %	21.3
R.m.s.d. bond length, Å	0.008
R.m.s.d. bond angles, º	2.399
